# HIV Pre-exposure Prophylaxis Education for Clinicians Caring for Spanish-Speaking Men Who Have Sex With Men (MSM)

**DOI:** 10.15766/mep_2374-8265.11110

**Published:** 2021-03-18

**Authors:** Luis Alzate-Duque, John P. Sánchez, Sebastian R. Mendez Marti, Dwindally Rosado-Rivera, Nelson F. Sánchez

**Affiliations:** 1 Assistant Professor of Medicine, Department of Medicine, Rutgers New Jersey Medical School; 2 President, Building the Next Generation of Academic Physicians; 3 Resident, Inova Fairfax Internal Medicine Residency Program; 4 Instructor, Department of Medicine, Weill Cornell Medicine; 5 Associate Professor of Medicine, Department of Medicine, Weill Cornell Medicine

**Keywords:** Medical Spanish, PrEP, Pre-exposure Prophylaxis, MSM, HIV, LGBTQ, Sexual and Gender Minorities, Diversity, Inclusion, Health Equity

## Abstract

**Introduction:**

A growing number of Liaison Committee on Medical Education–accredited allopathic medical schools offer formal bilingual (English and Spanish) medical education, and numerous other schools offer medical Spanish through elective workshops as part of their curricula. One significant health disparity in the Hispanic community is the incidence of HIV among Spanish-speaking men who have sex with men (MSM). Pre-exposure prophylaxis (PrEP) has emerged as an effective strategy to reduce the risk of HIV transmission.

**Methods:**

We developed an education module to train clinicians to discuss PrEP with Spanish-speaking MSM. Our module is adapted from an English module on PrEP education. It includes a Spanish-language PowerPoint slide deck with information about PrEP as well as a Spanish-language videotaped scripted clinical encounter.

**Results:**

The module was implemented on three occasions with 18 participants, and learners reported increased comfort in discussing and confidence in prescribing PrEP with Spanish-speaking patients.

**Discussion:**

This workshop can be incorporated within medical Spanish curriculums offered at health professional schools and community-based organizations dedicated to reducing the HIV burden in the Spanish-speaking Hispanic community.

## Educational Objectives

By the end of this session, learners will be able to meet the following health objectives with Spanish-speaking patients:

1. Describe the indications for pre-exposure prophylaxis (PrEP) prescription.
2. Describe appropriate testing and medical care for PrEP initiation, maintenance, adherence, and discontinuation.
3. Demonstrate culturally informed communication skills needed for PrEP screening and patient education.

## Introduction

An estimated 1.7 million new HIV infections occur each year worldwide.^1^ In 2018, 37,968 individuals were newly diagnosed with HIV in the United States; men who have sex with men (MSM) accounted for 69% of those,^2^ whereas 20% were Hispanic (for the purposes of this module, Hispanic encompasses the terms *Latino, Hispanic,* and *Latinx*). Additionally, from 2010 to 2016, the number of HIV diagnoses among Hispanic MSM increased 21%; this subpopulation of new HIV diagnoses was the second largest in the United States, following Black or African American MSM.^3^ The CDC does not collect data specifically on Spanish-speaking MSM, but we do know that the majority of United States Hispanics are Spanish proficient.^4^ Hispanics are less likely than their ethno-racial counterparts to access HIV testing and treatment.^3^ Pre-exposure prophylaxis (PrEP) is an evidence-based preventative treatment that prevents HIV infection for at-risk populations, such as MSM.^5–7^ Studies have shown that PrEP can reduce the risk of HIV transmission by more than 90%.^8–11^ Despite the well-known benefits of PrEP, it is not widely prescribed, and its uptake among Hispanic MSM has trailed their White peers.^12,13^ Improving clinicians' culturally informed communication skills with Hispanic MSM could improve Hispanic MSM PrEP uptake and reduce HIV transmission.

Clinicians' poor recognition of at-risk populations has negatively impacted patient access to PrEP,^14^ prompting the need to reinforce indications for PrEP prescriptions, which is our first objective. Providers' lack of comfort with prescribing PrEP has emerged as a factor that consequently affects access to its use.^14^ Educating clinicians on the appropriate testing prior to initiation of the medication, as well as monitoring parameters including laboratory data, adherence, and discontinuation parameters, is an essential objective of this module. Another barrier to PrEP access may be clinicians' limited Spanish proficiency and the challenges of caring for a growing Spanish-speaking population that now surpasses 50 million people in the United States.^15–17^ Additional barriers include the stigmatization of high-risk populations, limited access to PrEP-informed providers, and mistrust of the medical community among Hispanic MSM.^18^ In light of these, the main objectives of this module are to build provider awareness of at-risk populations who could benefit from PrEP, enhance provider comfort in prescribing PrEP, and develop cultural humility to improve PrEP uptake.

The CDC, through the Act Against AIDS initiative, provides culturally informed and language appropriate prevention campaigns using mass media to deliver HIV prevention messages. Current programs in the CDC compendium include interventions and strategies to reduce sexual risk in HIV-negative individuals, including the use of PrEP and post-exposure prophylaxis. Currently, there are limited strategies and interventions specifically tailored for Hispanic MSM.^18,19^ Furthermore, there exists a need for the development and implementation of educational programs for clinicians to promote PrEP use and adherence.^20^ With a paucity of research in PrEP access, existing barriers contributing to disparities in HIV outcomes, and existing need for culturally informed interventions, an educational instruction to better prepare trainees in caring for Spanish-speaking Hispanic MSM populations is needed. Uniquely, this module provides standardized medical Spanish curricular content related to HIV and PrEP education.

There are five *MedEdPORTAL* publications that address medical Spanish education.^21–25^ The published medical Spanish modules focus on the proper use of medical interpreters in clinical encounters,^21^ the use of Spanish-language vignettes to teach medical Spanish,^25^ and the use of role-play scenarios to teach medical Spanish.^23^ Given the scarcity of data addressing the specific challenges that providers face when attending to the needs of special at-risk populations, such as Spanish-speaking Hispanic MSM, whether it is providers' level of comfort with Spanish language, with prescribing PrEP, or with establishing a dialogue about sexual practices in the setting of HIV risk, we consider the inclusion of this topic of utmost importance. Therefore, to support the development of the current module, we have adapted components of an English-language module on PrEP education.^26^

Our innovations for this module include the creation of new content and instructional materials. New content includes HIV incidence and prevalence in the United States and Latin America and PrEP access among Hispanic MSM living in the United States and Latin America. Knowledge of the unique health disparities among Hispanic MSM and their access to PrEP will inform the learners' capacity to prescribe PrEP and tailor HIV prevention strategies. Our instructional materials to promote medical Spanish innovate by providing content via audio-recorded PowerPoint (PPT) slides, in both Spanish and English, and a Spanish-language videotaped scripted clinical encounter with Spanish- and English-language transcripts. Additionally, our content and instructional materials address medical Spanish core competencies and corresponding performance objectives designated by health professionals with expertise in medical Spanish.^16^

A team of Spanish-proficient faculty, fellows, and medical students with knowledge of PrEP and of caring for members of the LGBTQ community developed, implemented, and/or evaluated the workshop. Each team member was a first-generation Hispanic American or native of Latin America. An associate professor of internal medicine with clinical PrEP prescribing experience and research expertise, an assistant professor of internal medicine with teaching and research experience, and an internal medicine instructor with clinical PrEP prescribing experience served as workshop facilitators. Additionally, three authors identified as gay.

The six-step Kern model^27^ was applied as a framework for the design, implementation, and evaluation of this educational innovation as indicated below:

1. *Problem identification and general needs assessment:* These were performed via literature review and input from medical students, residents, fellows, and medical school faculty. Lack of awareness, comfort, and familiarity prescribing PrEP; lack of culturally informed practices and need for medical Spanish; and lack of standardized educational instructional material were identified as issues.
2. *Targeted needs assessment:* To better assess PrEP education needs across a subset of medical schools, we conducted a needs assessment to evaluate PrEP-related education. The 12-item needs assessment evaluated 112 participants' knowledge and comfort regarding PrEP, as well as their recommendations for topic inclusions. The assessment was disseminated by email to students at three Liaison Committee on Medical Education–accredited schools: Ponce Health Sciences University, Ponce, Puerto Rico; San Juan Bautista School of Medicine, Caguas, Puerto Rico; and Rutgers New Jersey Medical School, Newark, New Jersey. Medical students at Ponce Health Sciences University and San Juan Bautista School of Medicine received a bilingual medical education in English and Spanish. Almost 85% of the responders to our needs assessment self-reported knowledge about the risk factors for HIV transmission in sexually active individuals; however, only 22% self-reported knowledge about prescribing PrEP, and 42% reported comfort assessing HIV risk behaviors. Additionally, almost half the respondents were not knowledgeable about tests needed prior to prescribing PrEP, and more than half were not knowledgeable about PrEP follow-up testing or PrEP side effects. Given these results, there was a clear need for PrEP education for clinicians in English and Spanish.
3. *Goals and objectives:* Based on the literature review and our previously published module on PrEP,^26^ the goal of the medical Spanish educational workshop was to raise awareness and knowledge of PrEP and improve competence in caring for Spanish-speaking Hispanic MSM patients. Objectives included understanding the indications for PrEP prescription; describing appropriate testing and medical care for PrEP initiation, maintenance, adherence, and discontinuation; and learning culturally informed communication skills needed for PrEP screening and initiation. Content was also developed to meet Spanish-language core competencies and performance objectives.^16^ We organized the instruction of our goals and objectives in Spanish- and English-language PPT presentations (Appendices A and B).
4. *Educational strategies:* The material was presented via an in-person presentation at three sites: Rutgers New Jersey Medical School (*n* = 6); Universidad Central del Caribe School of Medicine, Bayamon, Puerto Rico (*n* = 6); and Weill Cornell Medicine, New York City, New York (*n* = 6). The 18 participants included nine medical students, five academic faculty, one resident, one nurse, one staff member of a community-based organization, and one researcher. Each session was offered as a presentation at an academic medicine professional development conference sponsored by Building the Next Generation of Academic Physicians. At these conferences, participants received information on best practices in preparing for an academic medicine career, including the development of medical education portfolios. Additionally, the three sites allowed access to participants who included native Spanish speakers and native English speakers. Presenters utilized a discussion guide to teach the appropriate content (Appendix E). A video demonstrating Spanish-language communication skills utilized during PrEP counseling was shown during the presentation (Appendix F).
5. *Implementation:* The 1-hour presentation was administered in the summer of 2019 and was offered to medical students, residents, fellows, and faculty.
6. *Evaluation and feedback:* Each participant was given the opportunity to complete a pre- and postpresentation form to evaluate the workshop design and content (Appendices I and J). Memorial Sloan Kettering Cancer Center (Protocol X19–024: May 29, 2019) and Rutgers New Jersey Medical School (Pro2018001609: February 28, 2019) Institutional Review Boards approved this study.

## Methods

### Workshop Content by Appendix

• *Appendix A—Spanish PPT slide deck presentation:* The content of the Spanish-language workshop was provided in the PPT presentation, which outlined the core content for learners, including key terms, the epidemiology of HIV in the United States and Latin America, a brief history of HIV prevention, best-practice guidelines for PrEP prescribing, and barriers to uptake of PrEP in Hispanic MSM.
• *Appendix B—English PPT slide deck presentation:* The content of the English-language PPT presentation was a direct translation of Appendix A. The presentation outlined the core content for learners, including key terms, the epidemiology of HIV in the United States and Latin America, a brief history of HIV prevention, best-practice guidelines for PrEP prescribing, and barriers to uptake of PrEP in Hispanic MSM.
• *Appendix C—Spanish audio-guided PPT slide deck presentation:* The flow and content of the workshop were featured in this video presentation. The Spanish-language presentation outlined the core content for learners, including key terms, the epidemiology of HIV in Latin America, a brief history of HIV prevention, best-practice guidelines for PrEP prescribing, and barriers to uptake of PrEP in Hispanic MSM.
• *Appendix D—English audio-guided PPT slide deck presentation:* The flow and content of the workshop were featured in this video presentation. The English-language presentation was a direct translation of Appendix C, and it outlined the core content for learners, including key terms, the epidemiology of HIV in Latin America, a brief history of HIV prevention, best-practice guidelines for PrEP prescribing, and barriers to uptake of PrEP in Hispanic MSM.
• *Appendix E—discussion guide:* This discussion guide was used to facilitate an in-person presentation of the provided PPT slides.
• *Appendix F—patient-physician video:* This videotaped scripted clinical encounter allowed learners to view Spanish-language communication skills used in PrEP counseling.
• Appendix G—Spanish transcript of patient-physician video.
• Appendix H—English transcript of patient-physician video.
• *Appendices I and J—pre- and postworkshop evaluation forms:* Among the three sites at which the workshop was evaluated, there were some similar and some different questions. At all three sites, participant demographics were collected on the presurvey. On the postsurvey, at all three sites, participants were asked to use a scale (1 = *poor*, 2 = *fair*, 3 = *good*, 4 = *very good*, 5 = *excellent*) to rate quality of the lecture and scripted clinical encounter. The postsurvey also asked participants to “Please comment on the strengths of the workshop” and “Please include your suggestions for the workshop.”

The following medical Spanish core competencies were addressed in the workshop^16^:

1. Medical Spanish knowledge regarding organ systems and medical interviewing, including a complete medical interview script.
2. Medical Spanish knowledge regarding common disease entities, including comprehension of information provide in Spanish by the patient and synthesis of information gathered into a working assessment/plan.
3. Patient-centered explanation of medical diagnoses/assessment, including ability to explain medical diagnoses in colloquially understandable language.
4. Patient-centered explanation of treatment/evaluation plan, including testing (blood, imaging, urine, etc.), medication management/instructions, therapeutic interventions and procedural discussions, and referrals/follow-up care.

The following medical Spanish performance objectives were addressed in the workshop^16^:

1. Obtaining an accurate, age-appropriate, focused medical history.
2. Successfully conducting the medical interview by asking appropriate questions that demonstrate evaluation of pertinent positives/negatives that sufficiently address differential diagnostic considerations for a given chief complaint.
3. Assessing patient comprehension of the information provided and addressing gaps in the patient's knowledge.
4. Orally communicating the treatment plan to the patient, adjusted for cultural, emotional, and literacy needs.
5. Assessing patient comprehension of the information provided and addressing gaps in the patient's knowledge.

The workshop featured two primary educational strategies: (1) a didactic component offered as a PPT slide deck instructing participants about HIV prevention methods and best practices in PrEP prescribing (Appendices A and B) and (2) a video-based scripted MSM patient encounter to teach appropriate communication skills for PrEP counseling (Appendix F). Workshops were not limited to a specific number of participants.

This workshop could serve as a stand-alone resource or be implemented in the context of a larger curriculum on LGBTQ health or HIV prevention for Spanish-speaking patients. Participants experienced transformative learning by acquiring knowledge about PrEP and appropriate clinical communication skills for its prescription among at-risk Spanish-speaking Hispanic MSM.

### Learners and Facilitators

This workshop was designed for clinicians and physicians-in-training and as such could be presented to medical students, residents, and/or faculty. Participants could be native Spanish speakers who had completed high school or non-native Spanish speakers who had passed a high-school advanced placement Spanish-language exam or college-equivalent course. The ideal facilitators were clinicians with an MD, DO, PA, or NP degree and with Spanish proficiency, experience in HIV prevention, and LGBTQ health care. The entire presentation could be completed within a 1-hour block.

In preparation for the workshop, facilitators reviewed the video presentation and evaluation forms. Reviewing the material took approximately 3 hours. Should future facilitators choose, they can give an oral presentation using the PPT slides provided. The content in the PPT slides is an exact replica of the audio-recorded PPT presentation.

### Materials

Additional materials needed to administer this workshop included pens, audiovisual equipment to show the PPT or video presentation, and printed copies of the evaluation forms.

### Time Line

The suggested length of the workshop was approximately 60 minutes, with a proposed time line below:

• Preworkshop evaluation (5 minutes).
• Slides 1–44: in-person PPT presentation (25 minutes) or audio-guided PPT presentation (17 minutes).
• Videotaped scripted clinical encounter (15 minutes).
• Postworkshop evaluation (5 minutes).
• Question-and-answer period (10 minutes).

The module can be altered for different presenters and learners:

• Option 1: Presenter plays Spanish- or English-language audio-guided PPT presentation (Appendix C or D) and scripted clinical encounter (Appendix F). This option is recommended for presenters with no or limited experience prescribing PrEP to MSM populations.
• Option 2: Presenter performs in-person presentation using supplied Spanish- or English-language PPT slides (Appendix A or B). A discussion guide is supplied (Appendix E). The session can then conclude by viewing the videotaped scripted clinical encounter (Appendix F). This option is recommended for presenters with experience prescribing PrEP to MSM populations.
• Option 3: Trainees are emailed the audio-guided PPT video presentation (Appendix C or D) and evaluation forms (Appendices I and J). We recommend that the instructor follows up with trainees via an in-person small-group discussion to discuss their comfort and knowledge as they pertain to PrEP prescribing.

## Results

This workshop was implemented at three sites: Universidad Central del Caribe School of Medicine (*n* = 6), Weill Cornell Medicine (*n* = 6), and Rutgers New Jersey Medical School (*n* = 6).

### Rutgers New Jersey Medical School

Participants included six medical students who self-identified as native Spanish-speaking. Participants were asked to rate their level of agreement (1 = *strongly disagree,* 5 = *strongly agree*) with seven statements. The Mann-Whitney *U* test was used to assess a statistical difference in pre- and posttest responses. The results of these assessments are described in Table 1.

**Table 1.**
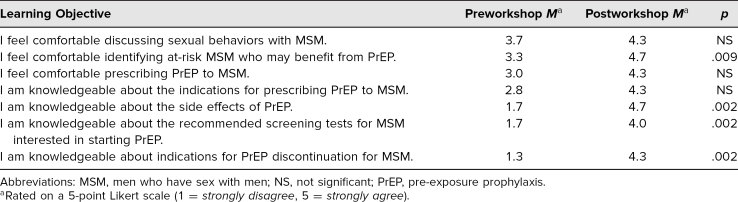
Assessment of Workshop Learning Objectives Among Rutgers New Jersey Medical School Participants (*n* = 6)

### Universidad Central del Caribe School of Medicine and Weill Cornell Medicine

Participants included five academic faculty, three medical students, one resident, one nurse, one staff member of a community-based organization, and one researcher. Participants were asked to rate their level of confidence regarding the learning objectives. The Mann-Whitney *U* test did not show a statistical difference in pre- and posttest responses per learning objective. Additionally, participants were asked to rate quality of the lecture and scripted clinical encounter. The results of these assessments are described in Table 2.

**Table 2.**
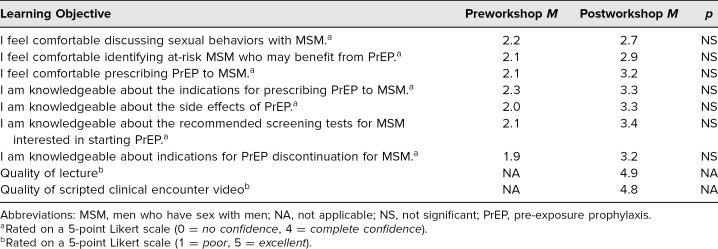
Assessment of Workshop Learning Objectives Among Universidad Central del Caribe School of Medicine and Weill Cornell Medicine Participants (*n* = 12)

In qualitative feedback, learners reported that the workshop was “well-organized” and “provided new information.” Participants recommended “correcting grammatical issues” and “providing alternatives for prevention.” The video was valued for “showing application of the material used in the PowerPoint” and was “a good tutorial” and “an excellent example on how to discuss PrEP with patients.”

## Discussion

This workshop to educate future clinicians on best practices in prescribing PrEP to Spanish-speaking Hispanic MSM is a successful educational innovation.

The module aligns with the National Hispanic Health Foundation and University of Illinois College of Medicine multidisciplinary expert panel consensus recommendations for integrating medical Spanish educational initiatives and training efforts as a strategy to improve Hispanic health and assess medical student learner skills through standardized patient encounters.^16^ The workshop addresses four core competencies and corresponding performance objectives recommended by the medical Spanish panel of experts. In alignment with multidisciplinary expert panel consensus recommendation 4, “provide a consensus core curriculum as a recommended structure for medical Spanish courses in U.S. medical schools,”^16^ our workshop seeks to build on appropriate usage of terminology (through the use of culturally accepted term use) and culturally appropriate health explanations, such as the exploration of barriers to PrEP uptake. Additionally, our videotaped scripted clinical encounter can be used as a standardized patient encounter instructional strategy, which aligns with consensus recommendation 5, “assessment of medical Spanish learner skills through standardized patient (SP) encounters that incorporate interactive communication and interpersonal skills.”^16^

We learned some lessons in the development of this 1-hour workshop. Completing a needs assessment prior to developing the workshop informed the content of our presentation as it related to the health concerns of Hispanics living in the United States and Latin America. We also learned that clinicians benefited from real-time demonstrations of Spanish-language clinical communication skills used to assess HIV risk and preventive care. Finally, we recognized that some educators might not have Spanish-language proficiency skills or might have minimal clinical experience with Hispanic MSM patients. We created audio-guided PPT slide deck presentations in both Spanish and English (Appendices C and D) so that the educational materials could be utilized by educators with poor Spanish-language skills or limited clinical experience with Hispanic MSM patients.

The data from our evaluation forms showed significant improvement in participants' comfort identifying high-risk MSM, knowledge of the side effects of PrEP, knowledge of recommend screening tests for MSM interested in starting PrEP, and knowledge of indications for PrEP discontinuation. In written feedback, participants expressed satisfaction with the workshop's subject matter and the videotaped scripted clinical encounter reviewing clinical communication skills used when prescribing PrEP to MSM populations. The videotaped encounter is unique in that it provides trainees with an opportunity to learn medical Spanish that is respectful and inclusive of sexual minorities.

While this presentation focuses on the Hispanic MSM population, it is important to remember that people of all sexual, ethno-racial, and gender identities can benefit from PrEP. High-risk populations for HIV transmission include individuals with multiple sexual partners and/or intravenous drug users. Recommendations for testing before prescribing PrEP and testing during PrEP maintenance are the same for all populations, with the addition of hepatitis C screening for intravenous drug users. The CDC website maintains up-to-date information on best practices in prescribing PrEP for all at-risk populations https://www.cdc.gov/hiv/risk/prep/index.html.^28^ Specific resources for other at-risk populations include webpages catering to cisgender women,^29^ intravenous drug users,^30^ and transwomen.^31^

### Limitations

Evaluation of this project was limited to three academic medical centers. The effectiveness of this education may also vary with audience. For example, senior health professionals who are uncomfortable with discussing sexual orientation or LGBTQ health may be more uncomfortable prescribing PrEP and may need additional training. This innovation also does not address skills acquisition. The workshop did not assess participants' ability to apply the material when engaging with Spanish-speaking patients, for example, via an observed standardized clinical examination. However, future presentations could potentially do so if role-plays with learner feedback are included. Additionally, PrEP prescribing recommendations may vary as new medications become available. We recommend that educators and participants review the CDC's PrEP website^32^ for up-to-date clinical recommendations. Finally, the impact of this workshop is limited in that we have assessed its immediate influence on participants' comfort and confidence prescribing PrEP. Sustained comfort and confidence may require repeated training and reinforcement via observed clinical encounters and continuing medical education activities.

### Conclusion

As U.S. medical schools develop, implement, and evaluate medical Spanish modules for their medical students, this module can serve as a seminal workshop for delivering medical Spanish training related to PrEP, HIV prevention, or sexual history taking. The module was developed by native Spanish speakers and reviewed by faculty who have been involved in implementing bilingual medical education for the past 10 years. Spanish is the second most common language spoken in the U.S., and the Hispanic population is expected to be one in four residents by 2050. Thus, medical Spanish modules on topics of a disparate burden to Hispanics are critical to providing equitable care for patients in the United States.

## Appendices

Spanish PPT Presentation.pptxEnglish PPT Presentation.pptxSpanish Audio-Guided PPT Video Presentation.pptxEnglish Audio-Guided PPT Video Presentation.pptxDiscussion Guide.docxPatient-Physician Video.mp4Spanish Transcript of Patient-Physician Video.docxEnglish Transcript of Patient-Physician Video.docxPreworkshop Evaluation Form.docxPostworkshop Evaluation Form.docx
All appendices are peer reviewed as integral parts of the Original Publication.

## References

[1] Global statistics. HIV.gov. 2018. Accessed October 16, 2020. https://www.hiv.gov/hiv-basics/overview/data-and-trends/global-statistics

[2] U.S. statistics. HIV.gov. 2018. Updated June 30, 2020. Accessed October 16, 2020. https://www.hiv.gov/hiv-basics/overview/data-and-trends/statistics

[3] HIV and Hispanics/Latinos. National Prevention Information Network. October 2018. Updated November 19, 2018. Accessed October 16, 2020. https://npin.cdc.gov/publication/hiv-and-hispanicslatinos

[4] Krogstad JM, Lopez MH. Use of Spanish declines among Latinos in major U.S. metros. Pew Research Center. October 31, 2017. Accessed October 16, 2020. https://www.pewresearch.org/fact-tank/2017/10/31/use-of-spanish-declines-among-latinos-in-major-u-s-metros/

[5] Petroll AE, Walsh JL, Owczarzak JL, McAuliffe TL, Bogart LM, Kelly JA. PrEP awareness, familiarity, comfort, and prescribing experience among US primary care providers and HIV specialists. AIDS Behav. 2017;21(5):1256–1267. 10.1007/s10461-016-1625-1 27885552 PMC5500978

[6] Maartens G, Celum C, Lewin SR. HIV infection: epidemiology, pathogenesis, treatment, and prevention. Lancet. 2014;384(9939):258–271. 10.1016/S0140-6736(14)60164-1 24907868

[7] Jones A, Cremin I, Abdullah F, et al. Transformation of HIV from pandemic to low-endemic levels: a public health approach to combination prevention. Lancet. 2014;384(9939):272–279. 10.1016/S0140-6736(13)62230-8 24740087

[8] Markowitz M, Grossman H, Anderson PL, et al. Newly acquired infection with multidrug-resistant HIV-1 in a patient adherent to preexposure prophylaxis. J Acquir Immune Defic Syndr. 2017;76(4):e104–e106. 10.1097/QAI.0000000000001534 29076941 PMC5792163

[9] Hoornenborg E, de Bree GJ. Acute infection with a wild-type HIV-1 virus in a PrEP user with high TDF levels. Presented at: Conference on Retroviruses and Opportunistic Infections; February 13–16, 2017; Seattle WA.

[10] Knox DC, Anderson PL, Harrigan PR, Tan DHS. Multidrug-resistant HIV-1 infection despite preexposure prophylaxis. N Engl J Med. 2017;376(5):501–502. 10.1056/NEJMc1611639 28146652

[11] Grossman H, Anderson P, Grant R, Gandhi M, Mohri H, Markowitz M. Newly acquired HIV-1 infection with multi-drug resistant (MDR) HIV-1 in a patient on TDF/FTC-based PrEP. Presented at: Conference on HIV Research for Prevention; October 17–21, 2016; Chicago, IL.

[12] Mapping PrEP: first ever data on PrEP users across the U.S. AIDSVu. March 6, 2018. Accessed October 16, 2020. https://aidsvu.org/prep/

[13] Brooks RA, Nieto O, Landrian A, Donohoe TJ. Persistent stigmatizing and negative perceptions of pre-exposure prophylaxis (PrEP) users: implications for PrEP adoption among Latino men who have sex with men. AIDS Care. 2019;31(4):427–435. 10.1080/09540121.2018.1499864 30021456 PMC6338523

[14] Krakower DS, Mayer KH. The role of healthcare providers in the roll out of preexposure prophylaxis. Curr Opin HIV AIDS. 2016;11(1):41–48. 10.1097/COH.0000000000000206 26417953 PMC4676563

[15] del Rio C. Latinos and HIV care in the Southeastern United States: new challenges complicating longstanding problems. Clin Infect Dis. 2011;53(5):488–489. 10.1093/cid/cir440 21844032

[16] Ortega P, Diamond L, Alemán MA, et al.; Medical Spanish Summit. Medical Spanish standardization in U.S. medical schools: consensus statement from a multidisciplinary expert panel. Acad Med. 2020;95(1):22–31. 10.1097/ACM.0000000000002917 31365394

[17] HIV and Hispanics/Latinos. Centers for Disease Control and Prevention. 2019. Updated October 13, 2020. Accessed October 16, 2020. https://www.cdc.gov/hiv/group/racialethnic/hispaniclatinos/index.html

[18] García M, Harris AL. PrEP awareness and decision-making for Latino MSM in San Antonio, Texas. PLoS One. 2017;12(9):e0184014. 10.1371/journal.pone.0184014 28953905 PMC5617149

[19] McCree DH, Walker T, DiNenno E, et al. A programmatic approach to address increasing HIV diagnoses among Hispanic/Latino MSM, 2010–2014. Prev Med. 2018;114:64–71. 10.1016/j.ypmed.2018.06.007 29908762

[20] Silapaswan A, Krakower D, Mayer KH. Pre-exposure prophylaxis: a narrative review of provider behavior and interventions to increase PrEP implementation in primary care. J Gen Intern Med. 2017;32(2):192–198. 10.1007/s11606-016-3899-4 27761767 PMC5264683

[21] Callahan E, Garcia E, Rehm J. Talk louder? Communicating with your Spanish speaking patients. MedEdPORTAL. 2011;7:8427. 10.15766/mep_2374-8265.8427

[22] Cesari WA, Brescia WF, Singh KH, et al. Medical Spanish. MedEdPORTAL. 2012;8:9171. 10.15766/mep_2374-8265.9171

[23] Ortega P, López-Hinojosa I, Park YS, Girotti JA. Medical Spanish musculoskeletal and dermatologic educational module. MedEdPORTAL. 2021;17:11071. 10.15766/mep_2374-8265.11071 33473381 PMC7809932

[24] O'Rourke K, Gruener G, Quinones D, Stratta E, Howell J. Spanish bilingual medical student certification. MedEdPORTAL. 2013;9:9400. 10.15766/mep_2374-8265.9400

[25] Rampal A, Wang C, Kalisvaart J. Pediatric medical Spanish vignettes. MedEdPORTAL. 2009;5:5110. 10.15766/mep_2374-8265.5110

[26] Perucho J, Alzate-Duque L, Bhuiyan A, Sánchez JP, Sánchez NF. PrEP (pre-exposure prophylaxis) education for clinicians: caring for an MSM patient. MedEdPORTAL. 2020;16:10908. 10.15766/mep_2374-8265.10908 32656329 PMC7336890

[27] Thomas PA, Kern DE, Hughes MT, Chen BY, eds. Curriculum Development for Medical Education: A Six-Step Approach. 3rd ed. Johns Hopkins University Press; 2016.

[28] Pre-exposure prophylaxis (PrEP). Centers for Disease Control and Prevention. Updated May 13, 2020. https://www.cdc.gov/hiv/risk/prep/index.html

[29] PrEP for women. The Well Project. 2020. https://www.thewellproject.org/hiv-information/prep-women

[30] HIV and injection drug use. Centers for Disease Control and Prevention. Updated November 3, 2020. https://www.cdc.gov/hiv/risk/idu.html

[31] HIV prevention and care for the transgender population. Centers for Disease Control and Prevention. 2020. https://www.cdc.gov/hiv/clinicians/transforming-health/transgender-patients/prep-pep.html

[32] PrEP (pre-exposure prophylaxis). Centers for Disease Control and Prevention. Updated November 3, 2020. https://www.cdc.gov/hiv/basics/prep.html

