# Supplementary material for: HIV Pre-exposure Prophylaxis Education for Clinicians Caring for Spanish-Speaking Men Who Have Sex With Men (MSM)
Source: MedEdPORTAL. 2021 Mar 18;17:11110. doi: 10.15766/mep_2374-8265.11110 (PMC8015640; doi:10.15766/mep_2374-8265.11110)
Supplement: Supplementary file 1 — Spanish PPT Presentation.pptxEnglish PPT Presentation.pptxSpanish Audio-Guided PPT Video Presentation.pptxEnglish Audio-Guided PPT Video Presentation.pptxDiscussion Guide.docxPatient-Physician Video.mp4Spanish Transcript of Patient-Physician Video.docxEnglish Transcript of Patient-Physician Video.docxPreworkshop Evaluation Form.docxPostworkshop Evaluation Form.docx [file mep_2374-8265.11110-s001.zip › A. Spanish PPT Presentation.pptx]

## Slide 1
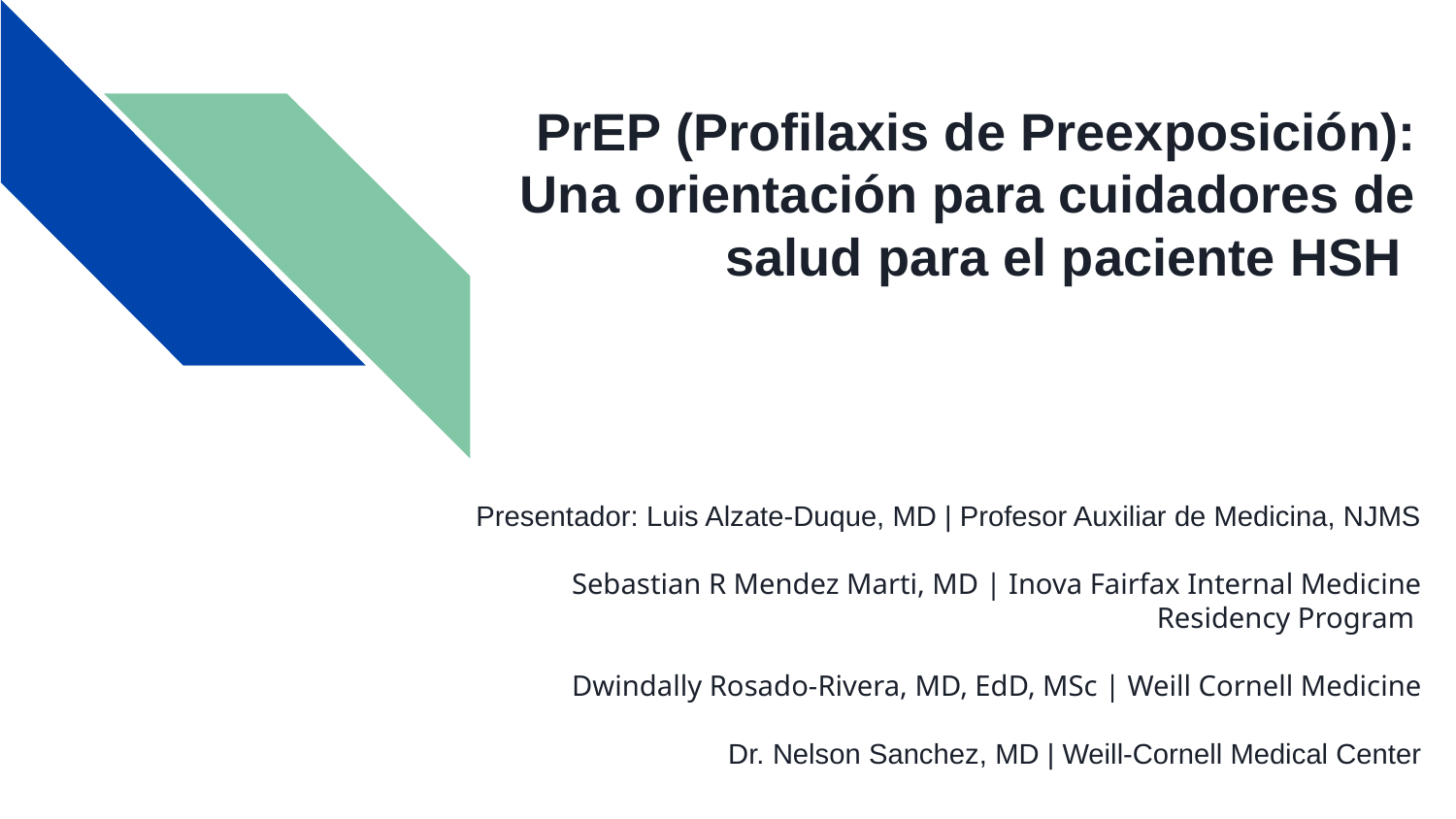

# PrEP (Profilaxis de Preexposición): Una orientación para cuidadores de salud para el paciente HSH
Presentador: Luis Alzate-Duque, MD | Profesor Auxiliar de Medicina, NJMS
Sebastian R Mendez Marti, MD | Inova Fairfax Internal Medicine Residency Program
Dwindally Rosado-Rivera, MD, EdD, MSc | Weill Cornell Medicine
Dr. Nelson Sanchez, MD | Weill-Cornell Medical Center
1

## Slide 2
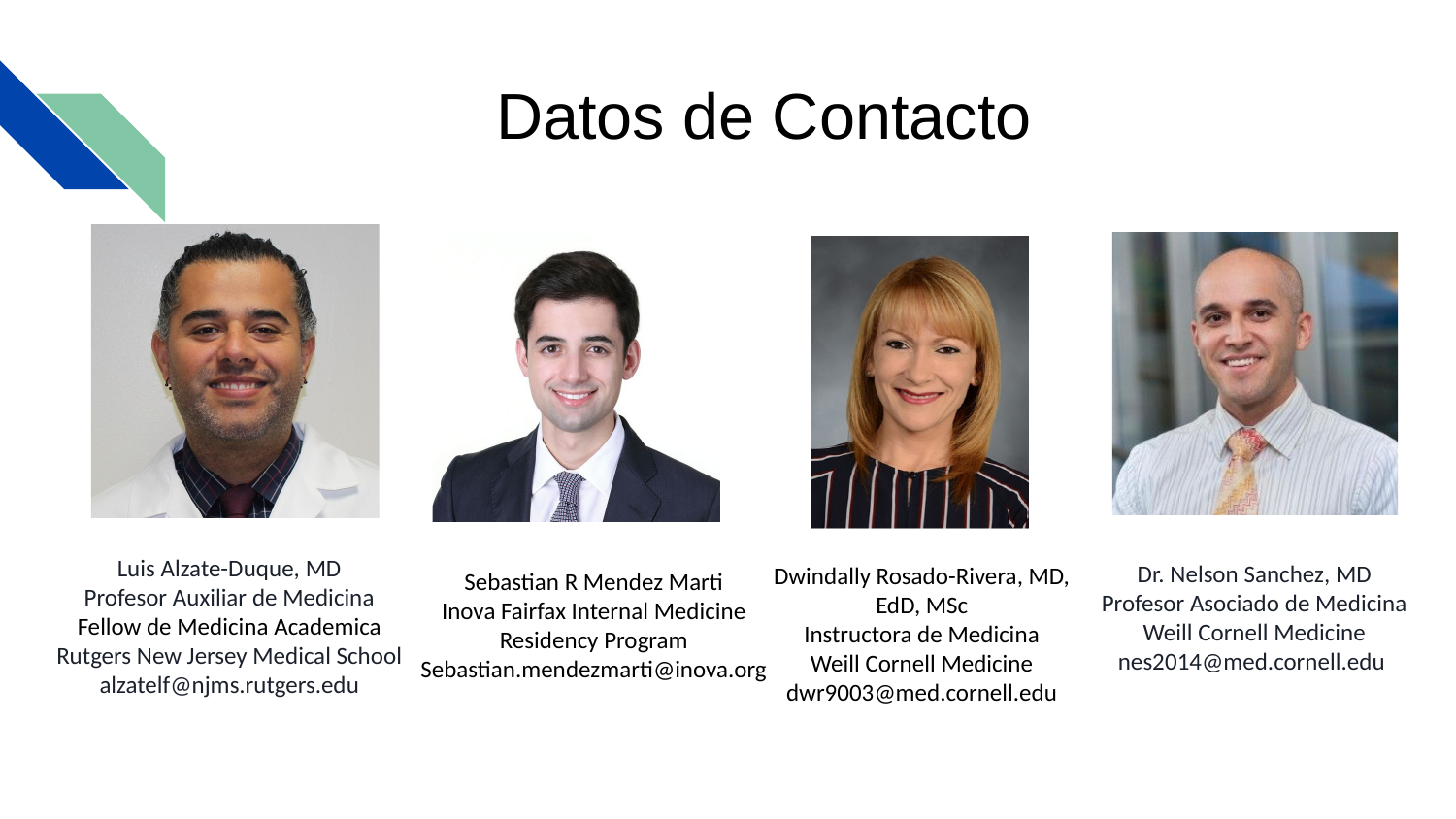

Datos de Contacto
Dr. Nelson Sanchez, MD
Profesor Asociado de Medicina
Weill Cornell Medicine
nes2014@med.cornell.edu
Sebastian R Mendez Marti
Inova Fairfax Internal Medicine Residency Program
Sebastian.mendezmarti@inova.org
Luis Alzate-Duque, MD
Profesor Auxiliar de Medicina
Fellow de Medicina Academica
Rutgers New Jersey Medical School
alzatelf@njms.rutgers.edu
Dwindally Rosado-Rivera, MD,
EdD, MSc
Instructora de Medicina
Weill Cornell Medicine
dwr9003@med.cornell.edu
2

## Slide 3
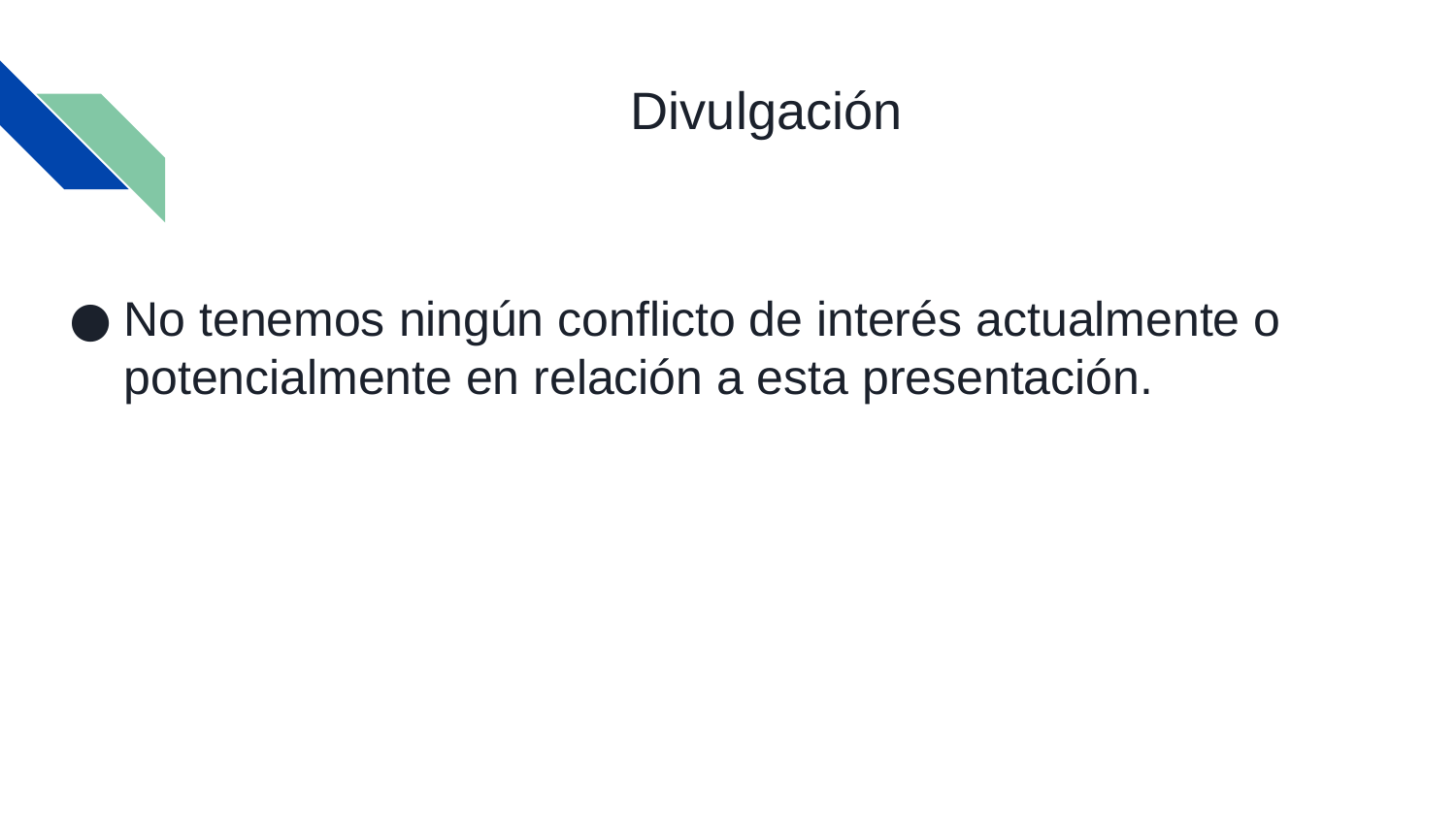

# Divulgación
No tenemos ningún conflicto de interés actualmente o potencialmente en relación a esta presentación.
3

## Slide 4
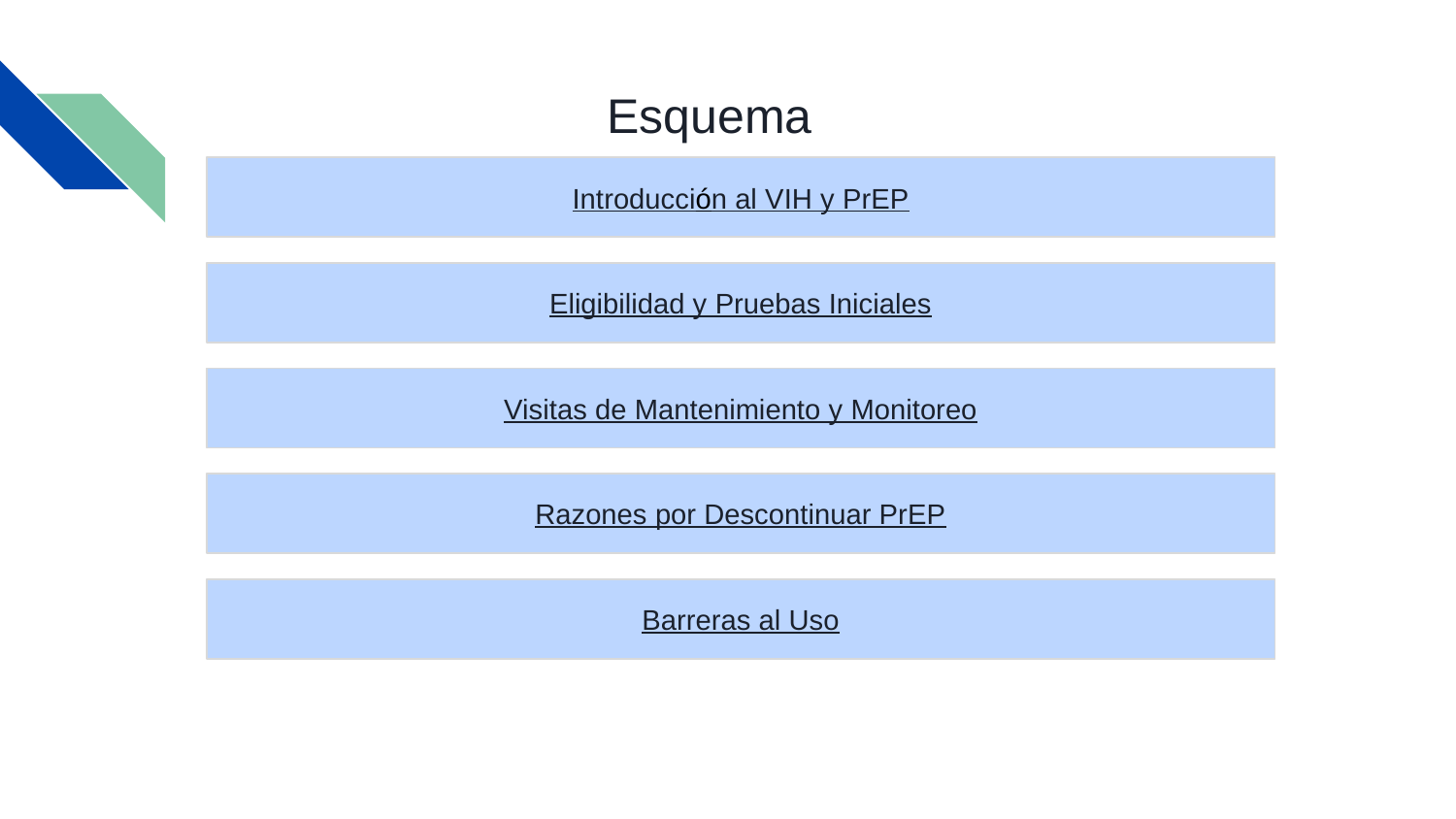

# Esquema
Introducción al VIH y PrEP
Eligibilidad y Pruebas Iniciales
Visitas de Mantenimiento y Monitoreo
Razones por Descontinuar PrEP
Barreras al Uso
4

## Slide 5
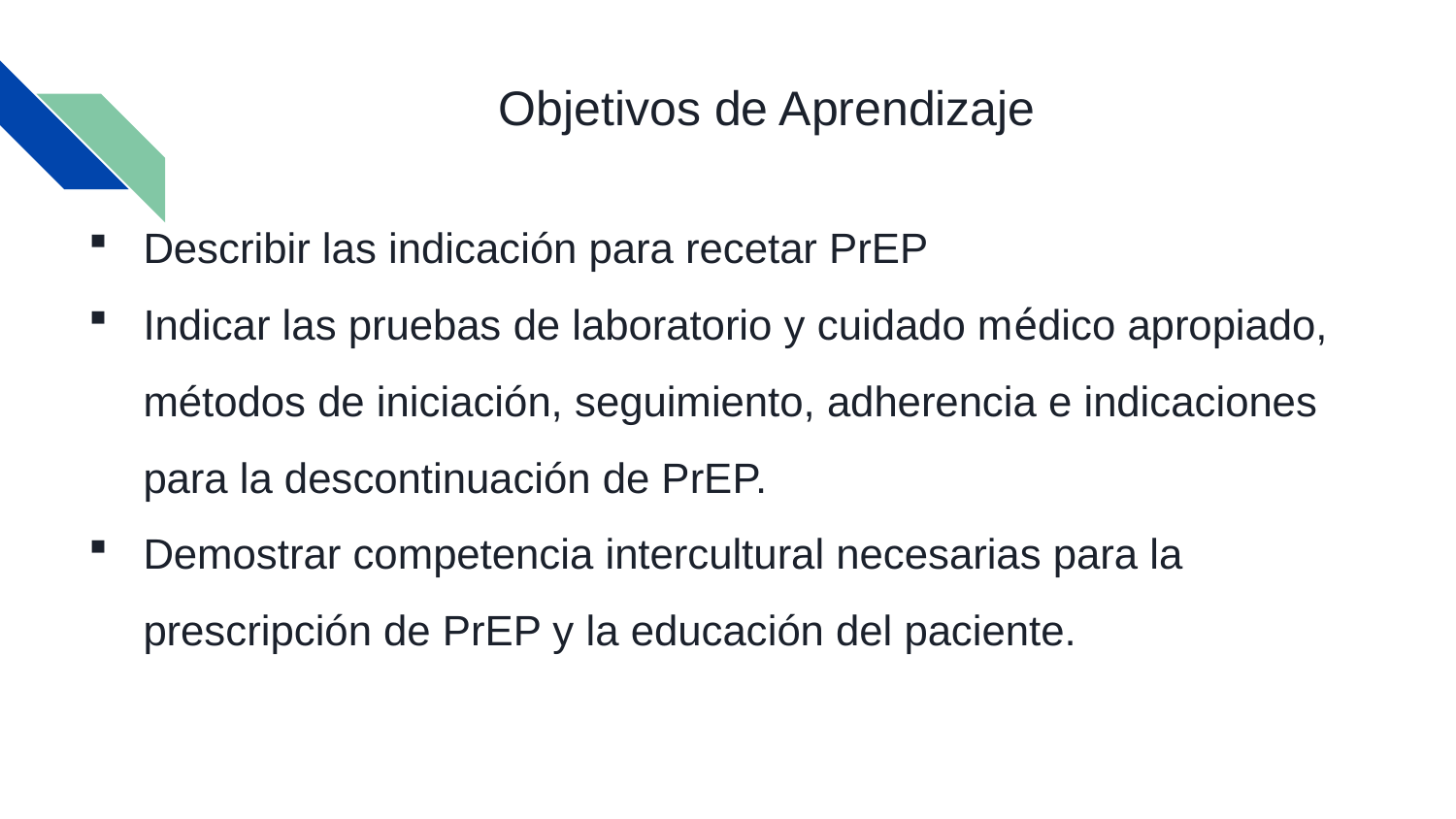

# Objetivos de Aprendizaje
Describir las indicación para recetar PrEP
Indicar las pruebas de laboratorio y cuidado médico apropiado, métodos de iniciación, seguimiento, adherencia e indicaciones para la descontinuación de PrEP.
Demostrar competencia intercultural necesarias para la prescripción de PrEP y la educación del paciente.
5

## Slide 6
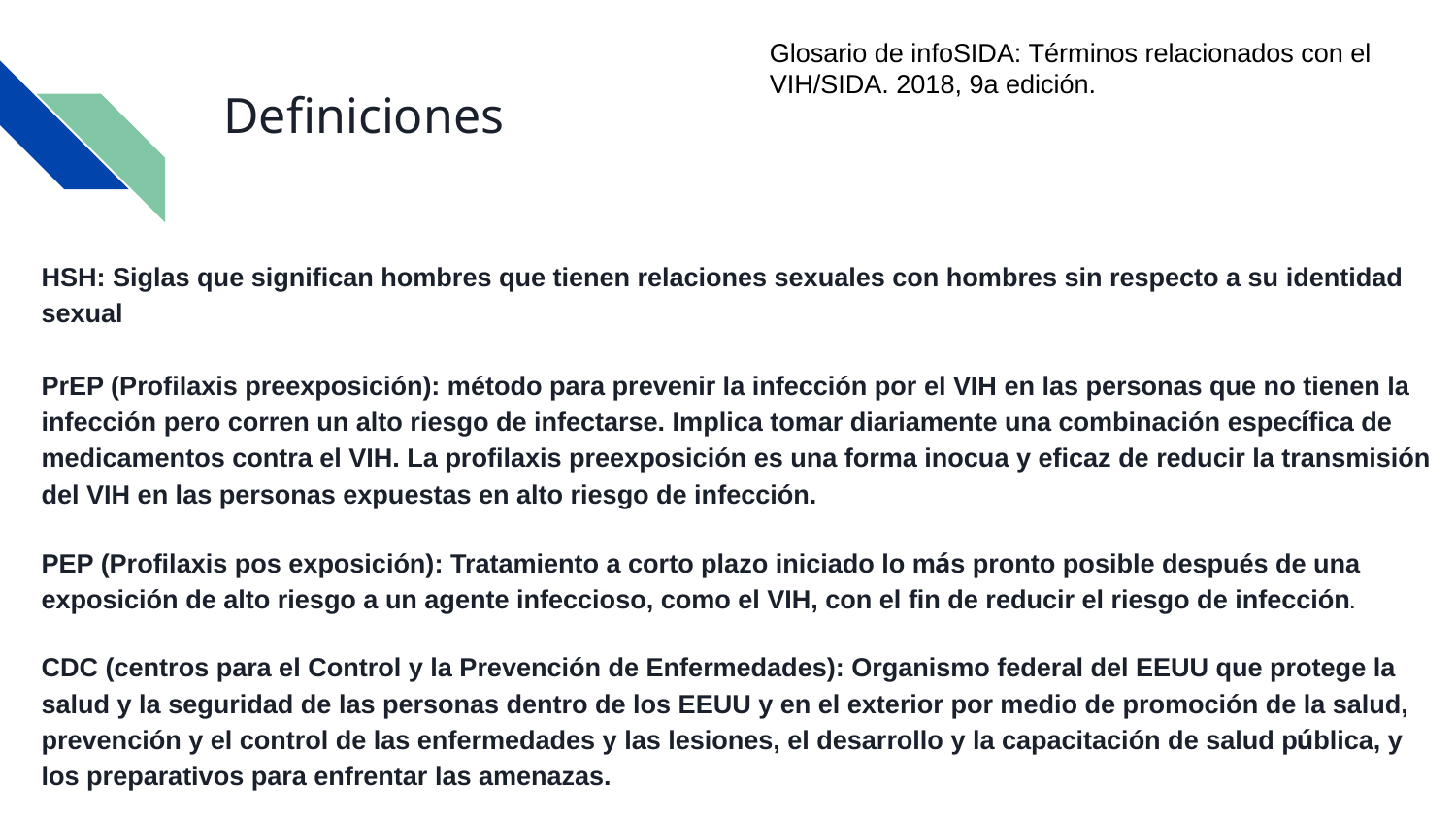

Glosario de infoSIDA: Términos relacionados con el VIH/SIDA. 2018, 9a edición.
# Definiciones
HSH: Siglas que significan hombres que tienen relaciones sexuales con hombres sin respecto a su identidad sexual
PrEP (Profilaxis preexposición): método para prevenir la infección por el VIH en las personas que no tienen la infección pero corren un alto riesgo de infectarse. Implica tomar diariamente una combinación específica de medicamentos contra el VIH. La profilaxis preexposición es una forma inocua y eficaz de reducir la transmisión del VIH en las personas expuestas en alto riesgo de infección.
PEP (Profilaxis pos exposición): Tratamiento a corto plazo iniciado lo más pronto posible después de una exposición de alto riesgo a un agente infeccioso, como el VIH, con el fin de reducir el riesgo de infección.
CDC (centros para el Control y la Prevención de Enfermedades): Organismo federal del EEUU que protege la salud y la seguridad de las personas dentro de los EEUU y en el exterior por medio de promoción de la salud, prevención y el control de las enfermedades y las lesiones, el desarrollo y la capacitación de salud pública, y los preparativos para enfrentar las amenazas.
6

## Slide 7
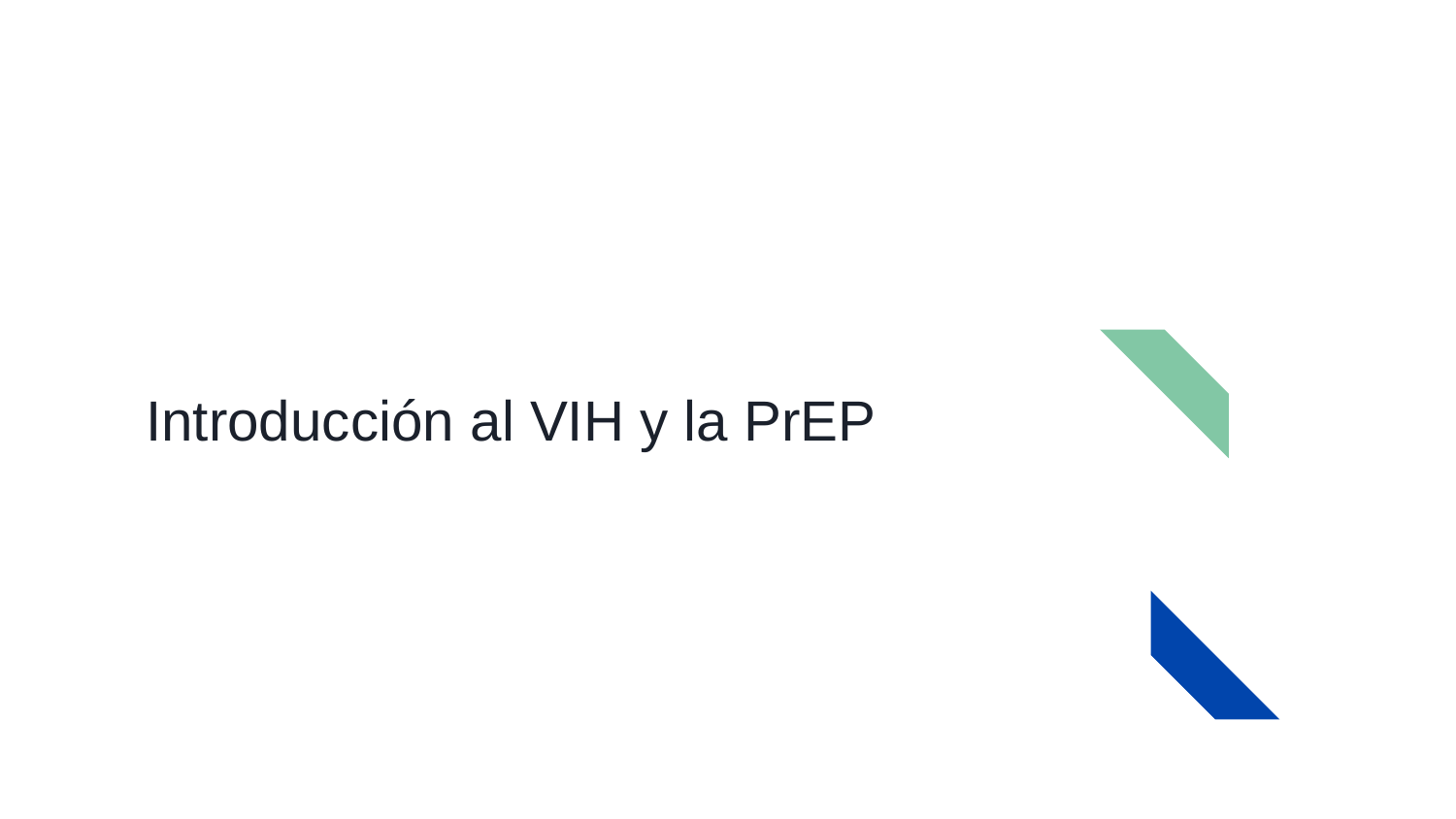

# Introducción al VIH y la PrEP
7

## Slide 8
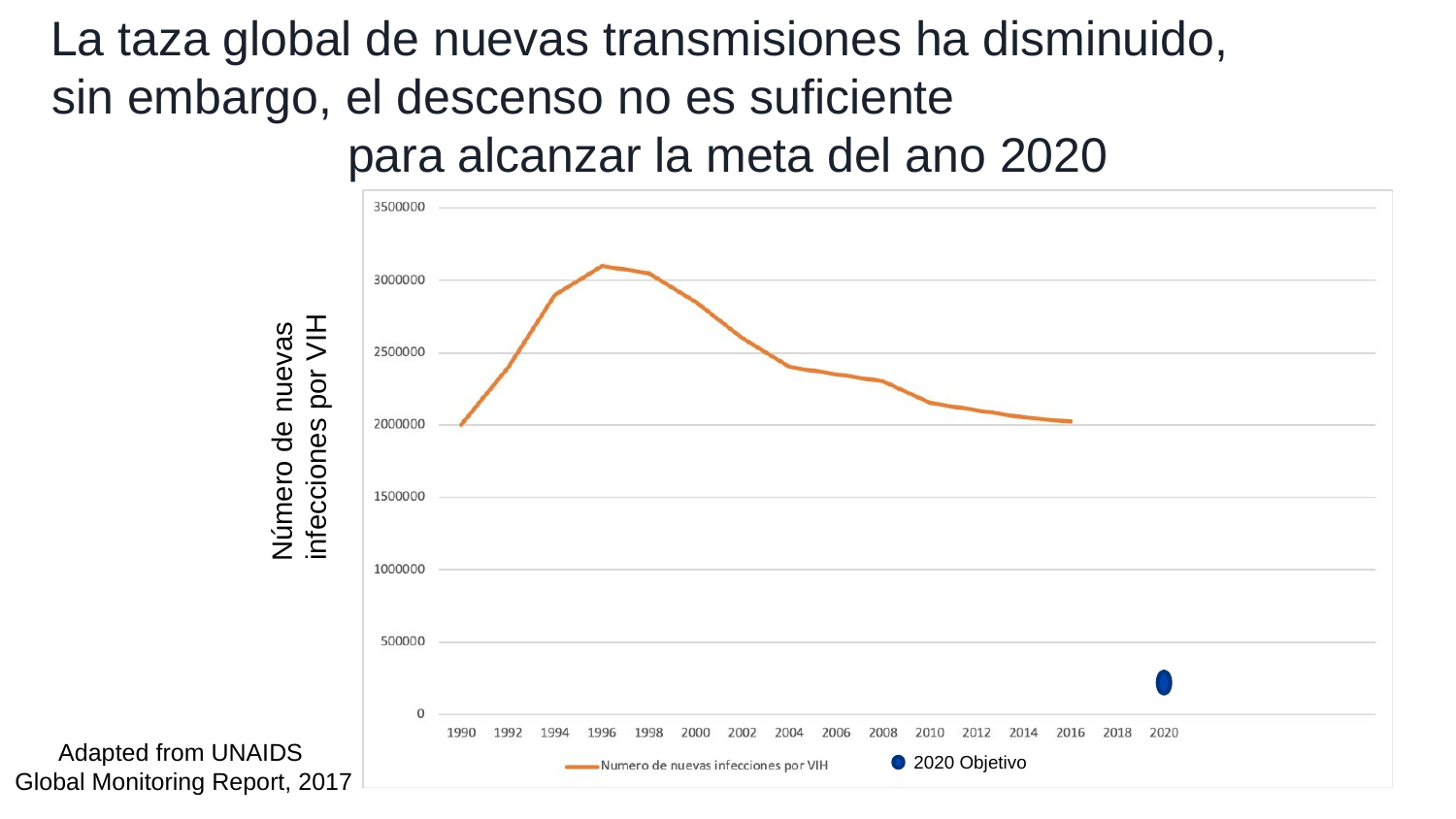

# La taza global de nuevas transmisiones ha disminuido, sin embargo, el descenso no es suficiente para alcanzar la meta del ano 2020
Número de nuevas
 infecciones por VIH
Adapted from UNAIDS
Global Monitoring Report, 2017
8
2020 Objetivo

## Slide 9
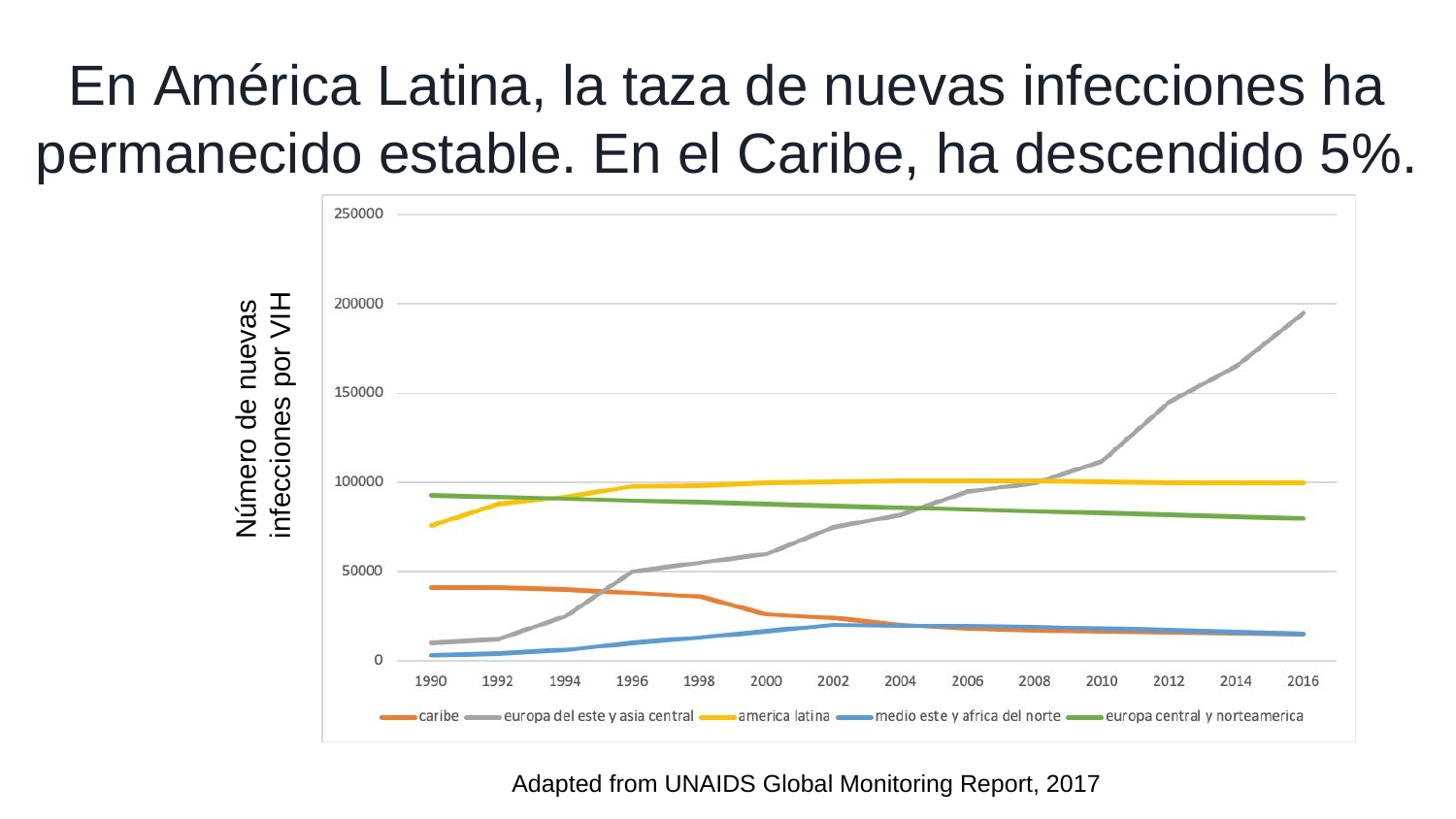

# En América Latina, la taza de nuevas infecciones ha permanecido estable. En el Caribe, ha descendido 5%.
Número de nuevas
infecciones por VIH
9
Adapted from UNAIDS Global Monitoring Report, 2017

## Slide 10
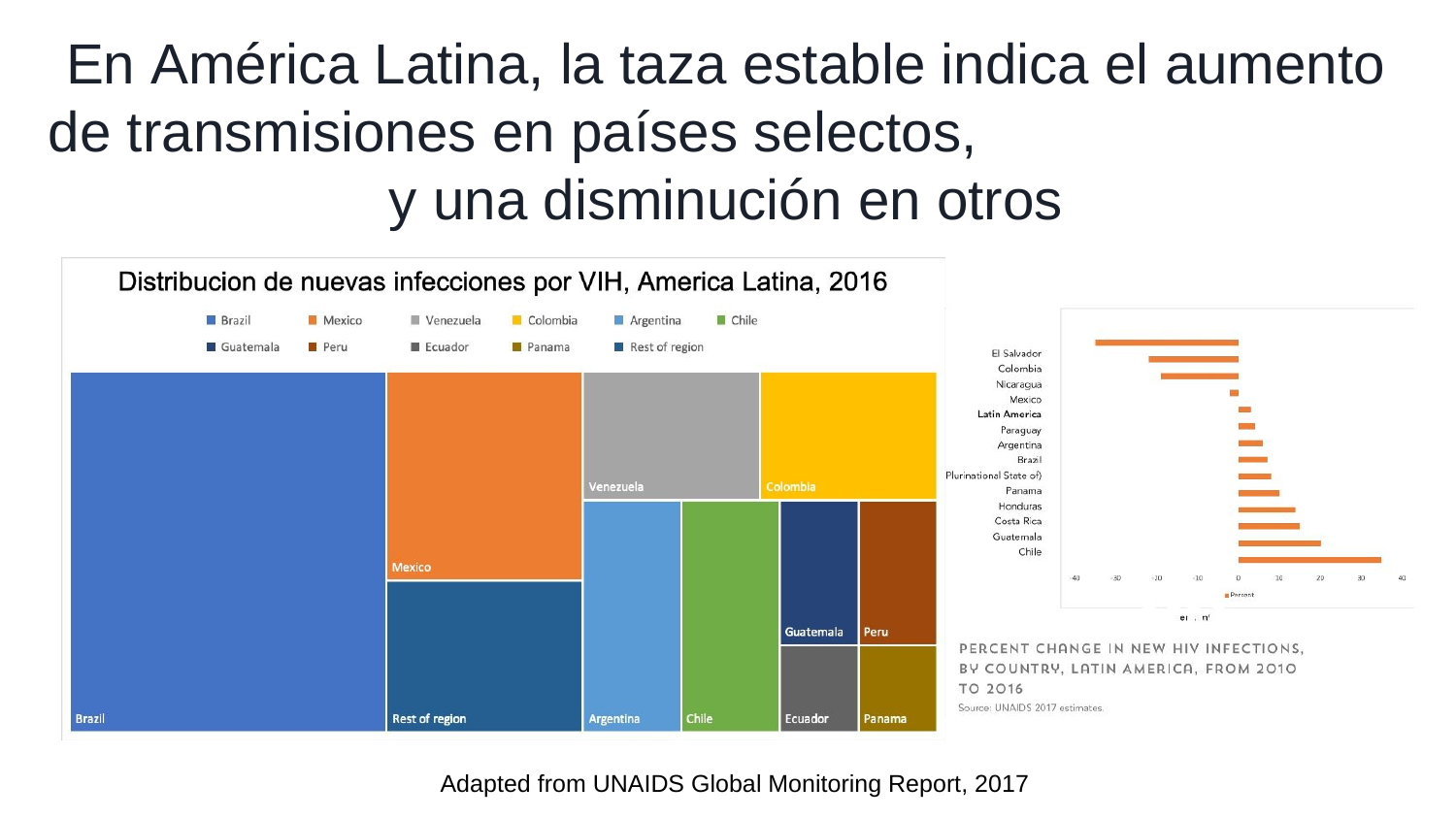

# En América Latina, la taza estable indica el aumento de transmisiones en países selectos, y una disminución en otros
10
Adapted from UNAIDS Global Monitoring Report, 2017

## Slide 11
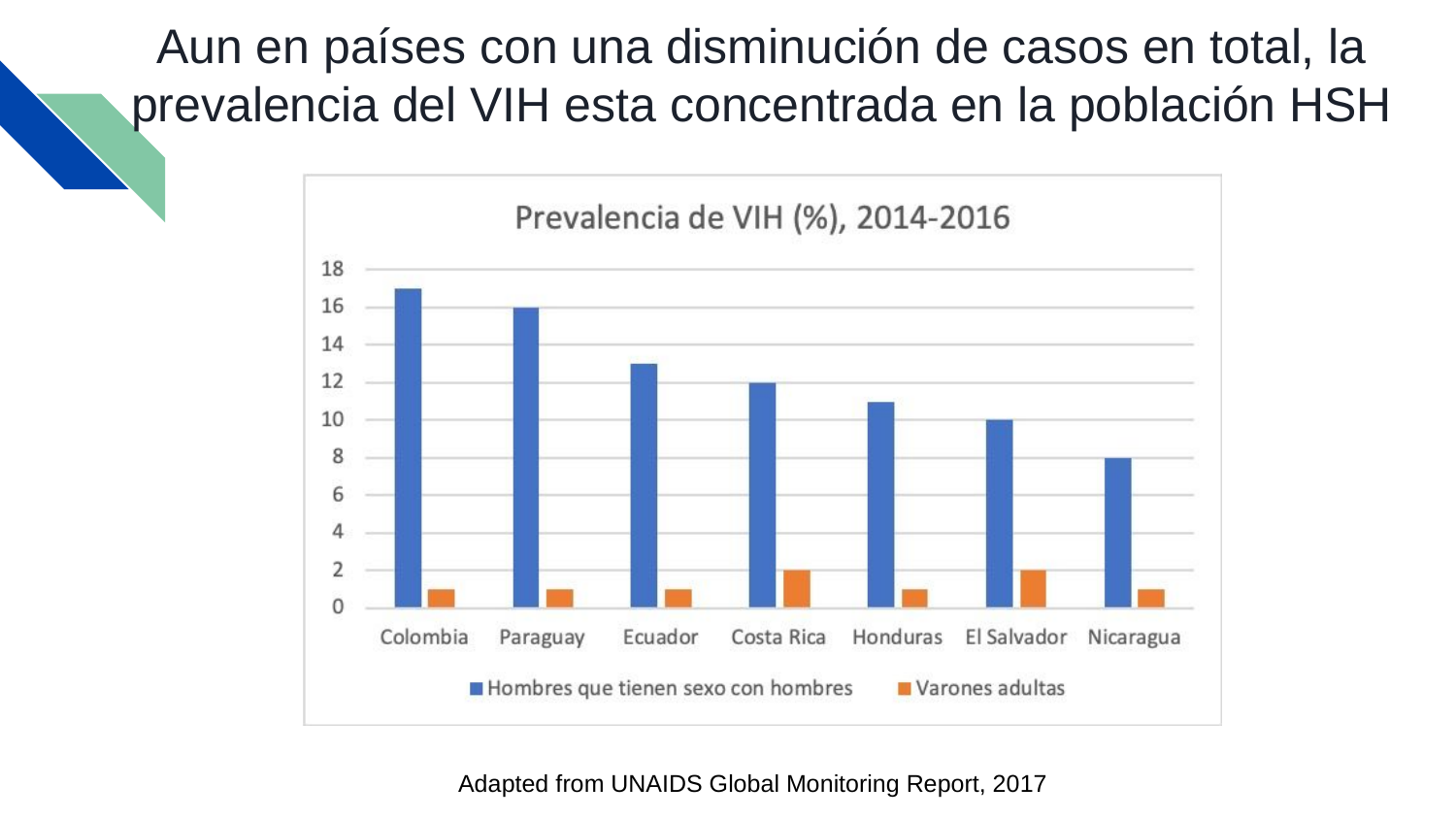

# Aun en países con una disminución de casos en total, la prevalencia del VIH esta concentrada en la población HSH
7/10
11
Adapted from UNAIDS Global Monitoring Report, 2017

## Slide 12
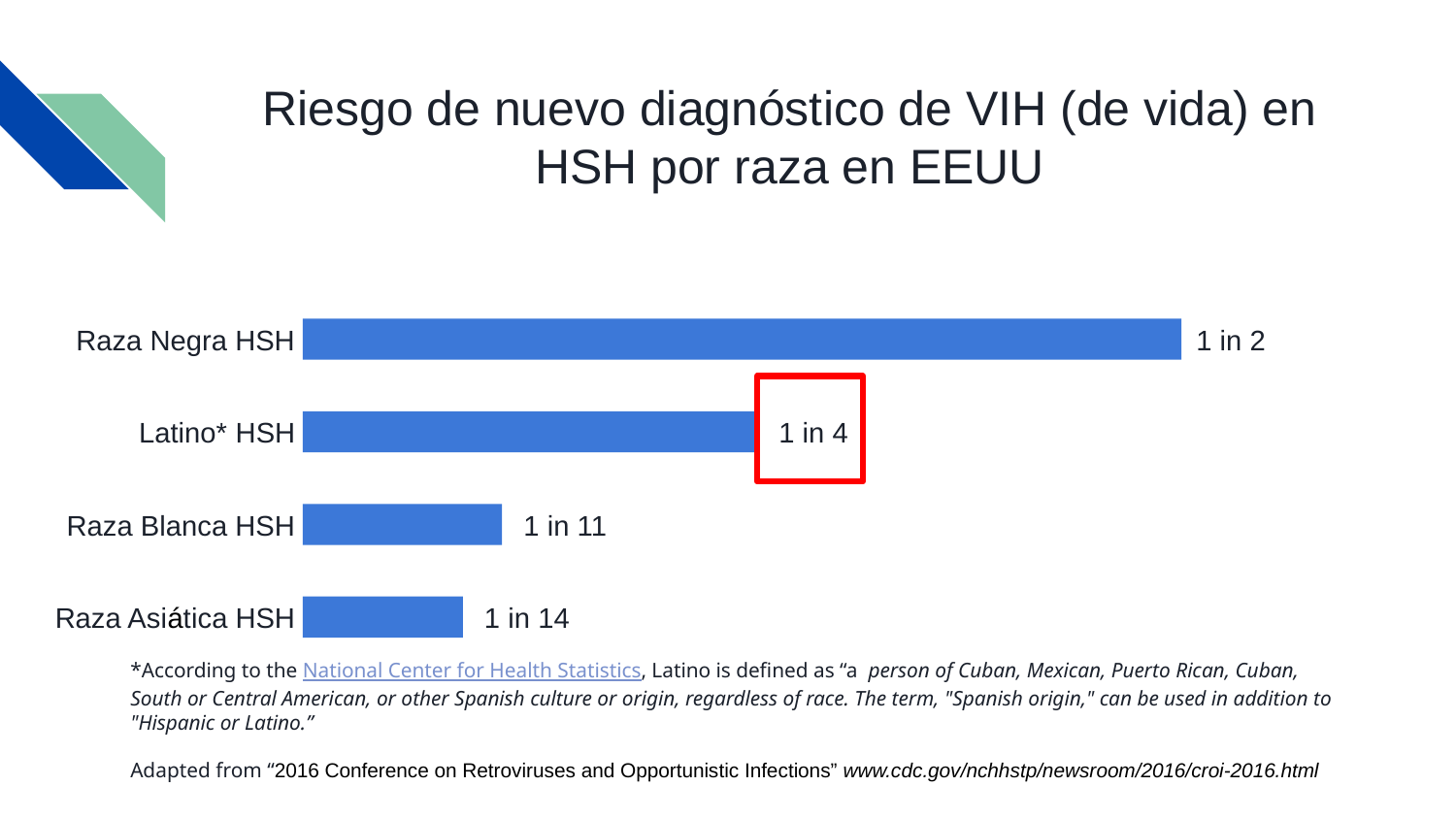

# Riesgo de nuevo diagnóstico de VIH (de vida) en HSH por raza en EEUU
Raza Negra HSH
1 in 2
Latino* HSH
1 in 4
Raza Blanca HSH
1 in 11
Raza Asiática HSH
1 in 14
*According to the National Center for Health Statistics, Latino is defined as “a person of Cuban, Mexican, Puerto Rican, Cuban, South or Central American, or other Spanish culture or origin, regardless of race. The term, "Spanish origin," can be used in addition to "Hispanic or Latino.”
Adapted from “2016 Conference on Retroviruses and Opportunistic Infections” www.cdc.gov/nchhstp/newsroom/2016/croi-2016.html
12

## Slide 13
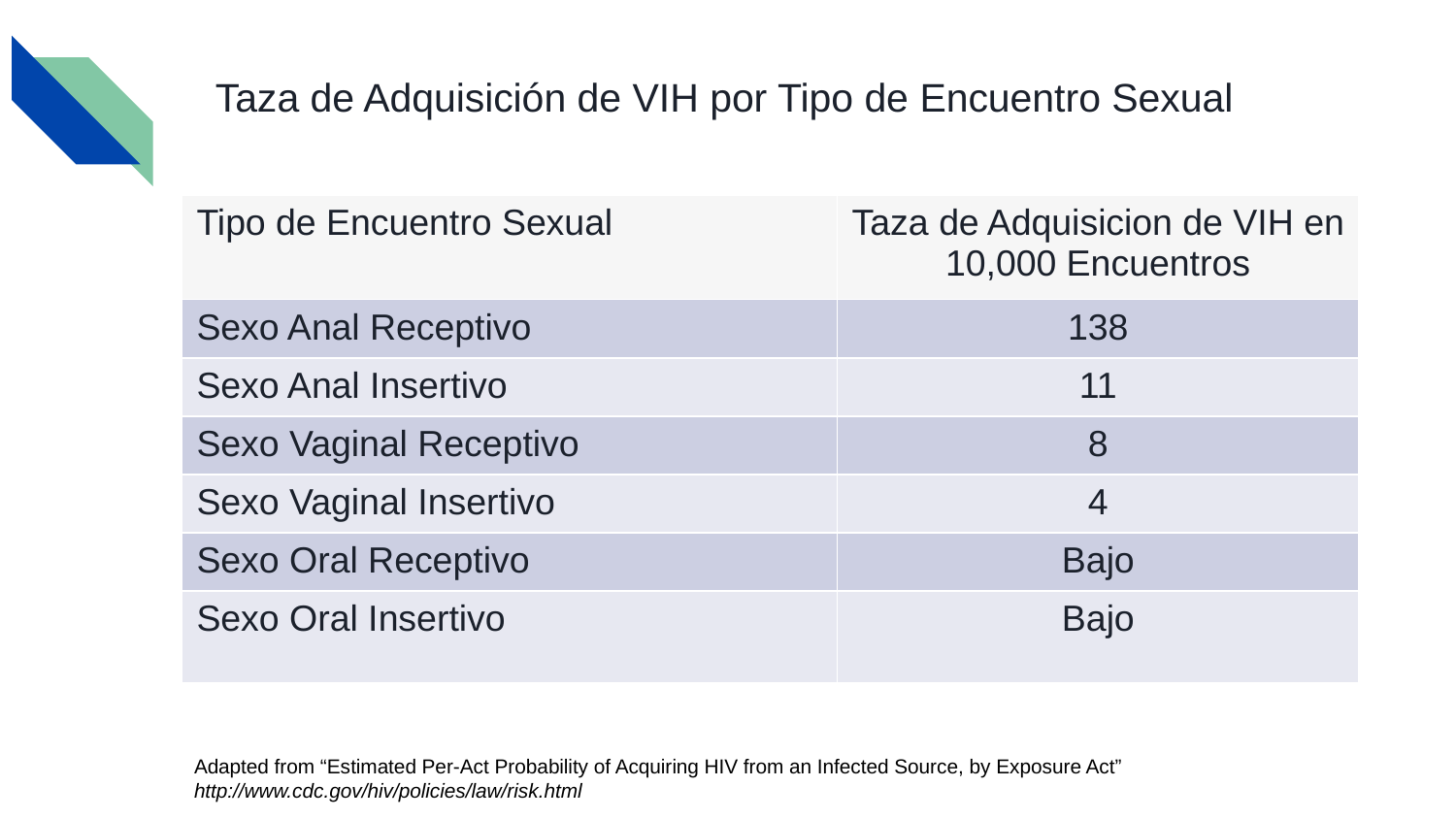

# Taza de Adquisición de VIH por Tipo de Encuentro Sexual
| Tipo de Encuentro Sexual | Taza de Adquisicion de VIH en 10,000 Encuentros |
| --- | --- |
| Sexo Anal Receptivo | 138 |
| Sexo Anal Insertivo | 11 |
| Sexo Vaginal Receptivo | 8 |
| Sexo Vaginal Insertivo | 4 |
| Sexo Oral Receptivo | Bajo |
| Sexo Oral Insertivo | Bajo |
Adapted from “Estimated Per-Act Probability of Acquiring HIV from an Infected Source, by Exposure Act” http://www.cdc.gov/hiv/policies/law/risk.html

## Slide 14
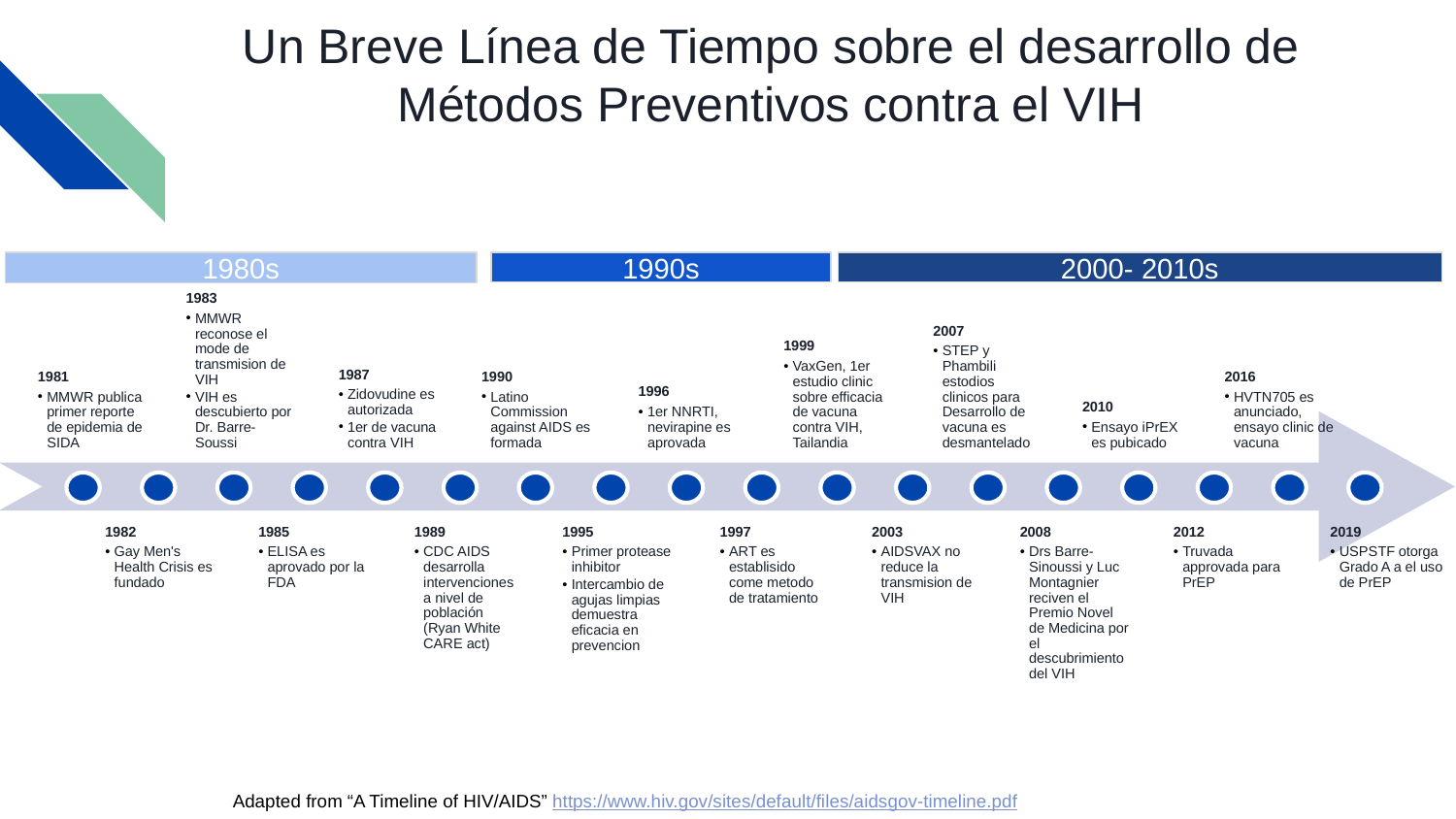

# Un Breve Línea de Tiempo sobre el desarrollo de Métodos Preventivos contra el VIH
1981
MMWR publica primer reporte de epidemia de SIDA
1983
MMWR reconose el mode de transmision de VIH
VIH es descubierto por Dr. Barre-Soussi
1987
Zidovudine es autorizada
1er de vacuna contra VIH
1990
Latino Commission against AIDS es formada
1996
1er NNRTI, nevirapine es aprovada
1999
VaxGen, 1er estudio clinic sobre efficacia de vacuna contra VIH, Tailandia
2007
STEP y Phambili estodios clinicos para Desarrollo de vacuna es desmantelado
2010
Ensayo iPrEX es pubicado
2016
HVTN705 es anunciado, ensayo clinic de vacuna
1982
Gay Men's Health Crisis es fundado
1985
ELISA es aprovado por la FDA
1989
CDC AIDS desarrolla intervenciones a nivel de población (Ryan White CARE act)
1995
Primer protease inhibitor
Intercambio de agujas limpias demuestra eficacia en prevencion
1997
ART es establisido come metodo de tratamiento
2003
AIDSVAX no reduce la transmision de VIH
2008
Drs Barre-Sinoussi y Luc Montagnier reciven el Premio Novel de Medicina por el descubrimiento del VIH
2012
Truvada approvada para PrEP
2019
USPSTF otorga Grado A a el uso de PrEP
1980s
1990s
2000- 2010s
14
Adapted from “A Timeline of HIV/AIDS” https://www.hiv.gov/sites/default/files/aidsgov-timeline.pdf

## Slide 15
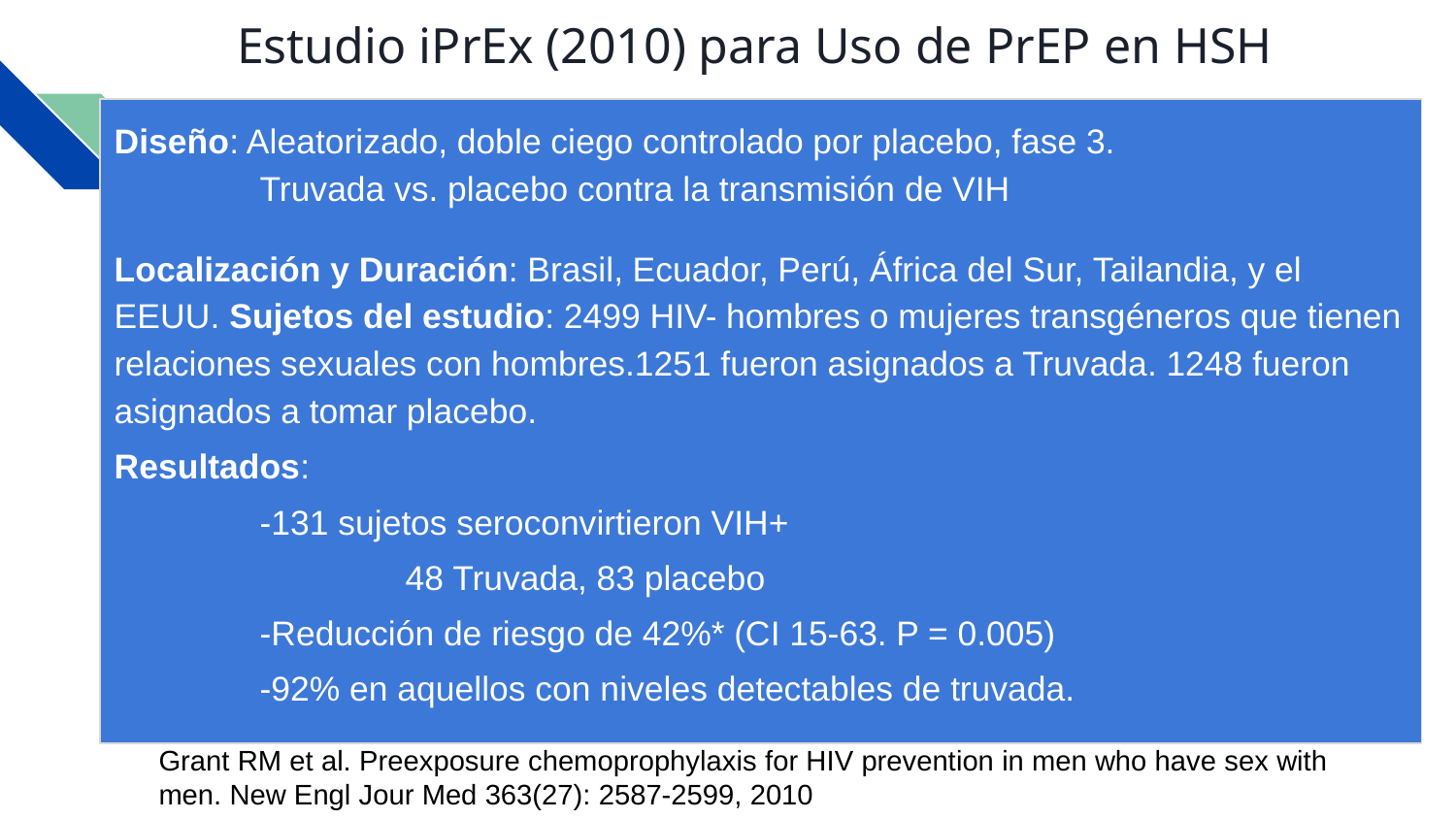

# Estudio iPrEx (2010) para Uso de PrEP en HSH
Diseño: Aleatorizado, doble ciego controlado por placebo, fase 3.
	Truvada vs. placebo contra la transmisión de VIH
Localización y Duración: Brasil, Ecuador, Perú, África del Sur, Tailandia, y el EEUU. Sujetos del estudio: 2499 HIV- hombres o mujeres transgéneros que tienen relaciones sexuales con hombres.1251 fueron asignados a Truvada. 1248 fueron asignados a tomar placebo.
Resultados:
	-131 sujetos seroconvirtieron VIH+
		48 Truvada, 83 placebo
	-Reducción de riesgo de 42%* (CI 15-63. P = 0.005)
	-92% en aquellos con niveles detectables de truvada.
Grant RM et al. Preexposure chemoprophylaxis for HIV prevention in men who have sex with men. New Engl Jour Med 363(27): 2587-2599, 2010
15

## Slide 16
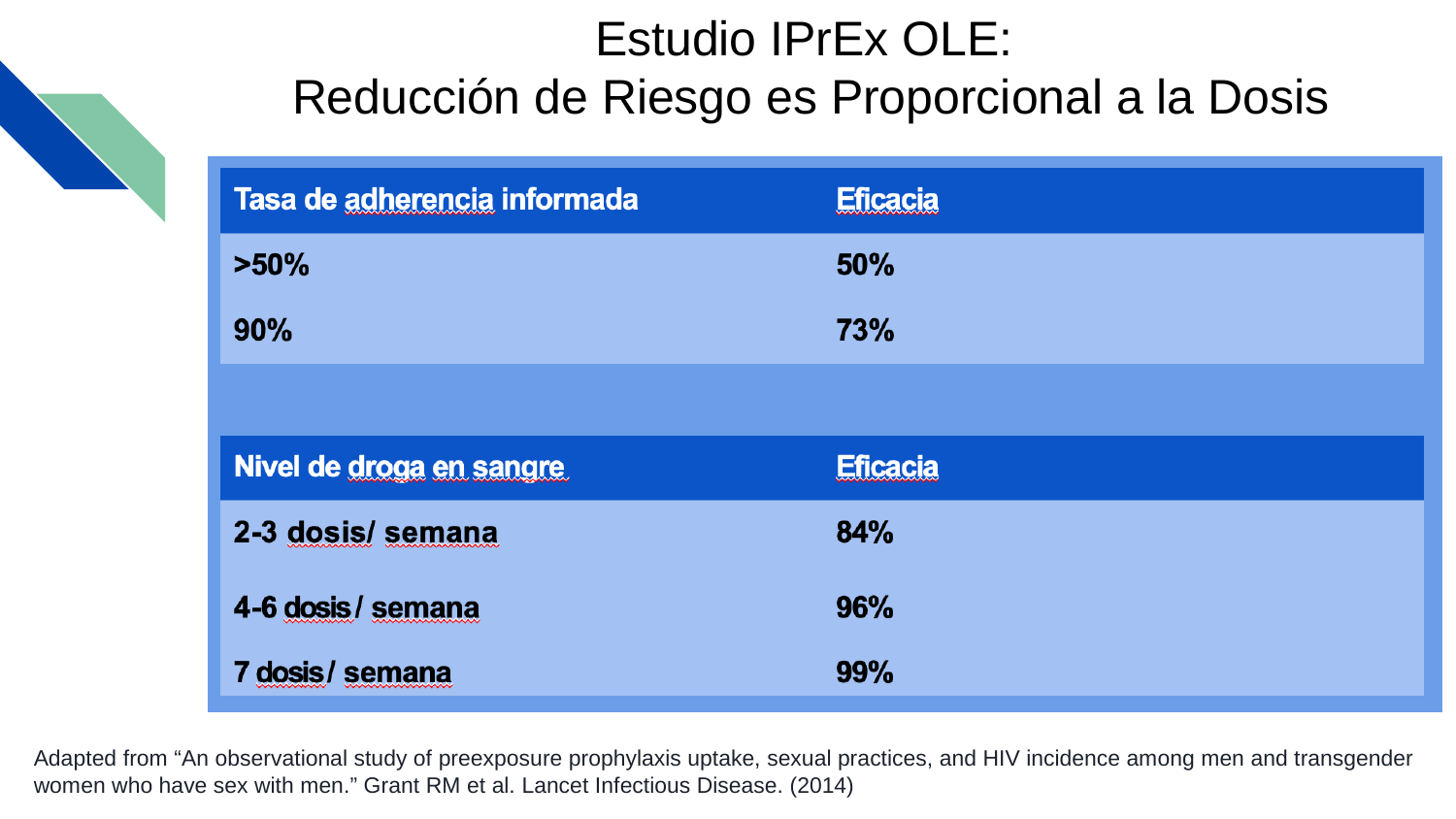

Estudio IPrEx OLE:
Reducción de Riesgo es Proporcional a la Dosis
# iPrEx Open Label Extension Study
Adapted from “An observational study of preexposure prophylaxis uptake, sexual practices, and HIV incidence among men and transgender women who have sex with men.” Grant RM et al. Lancet Infectious Disease. (2014)
16

## Slide 17
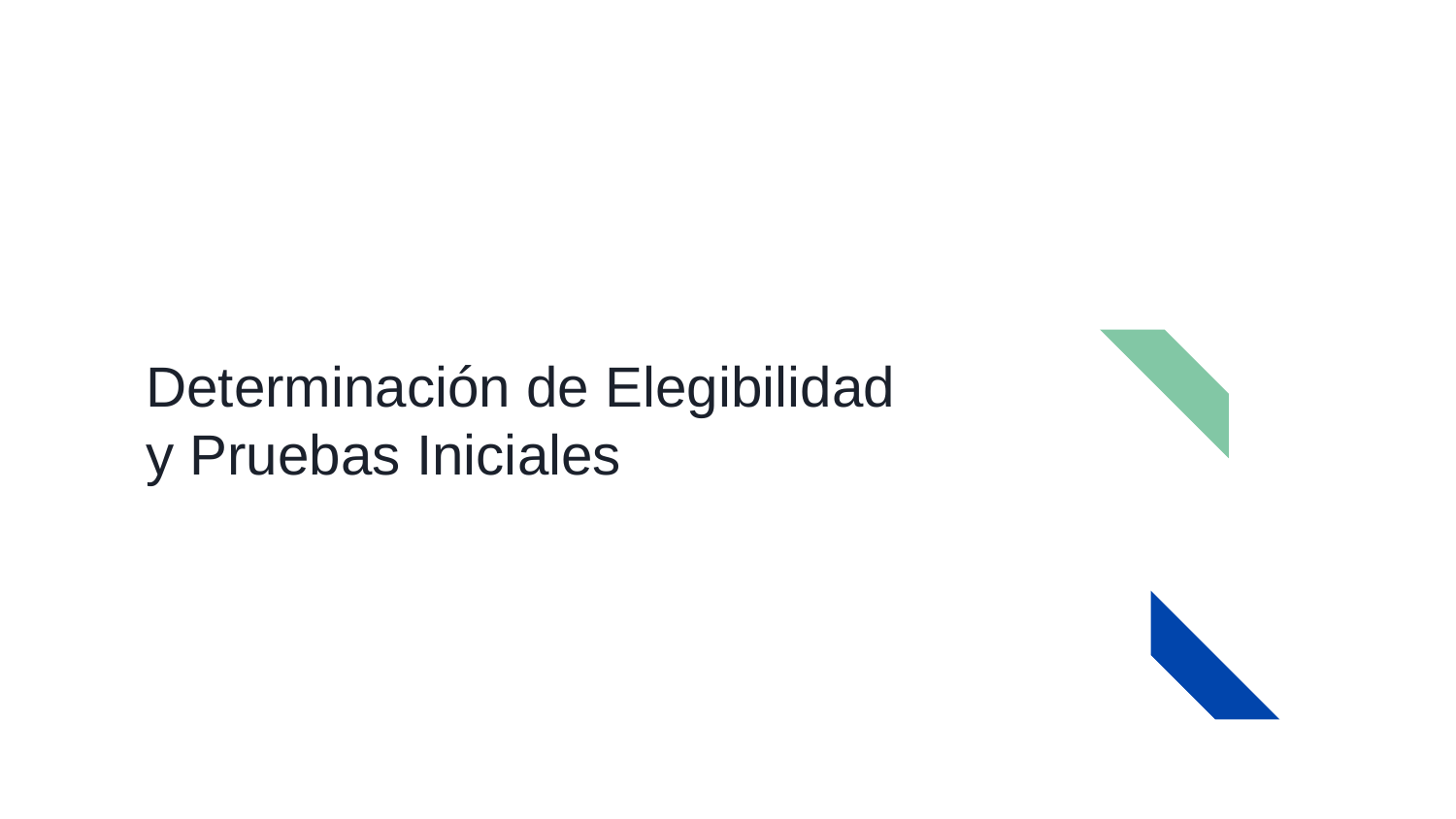

# Determinación de Elegibilidad y Pruebas Iniciales
17

## Slide 18
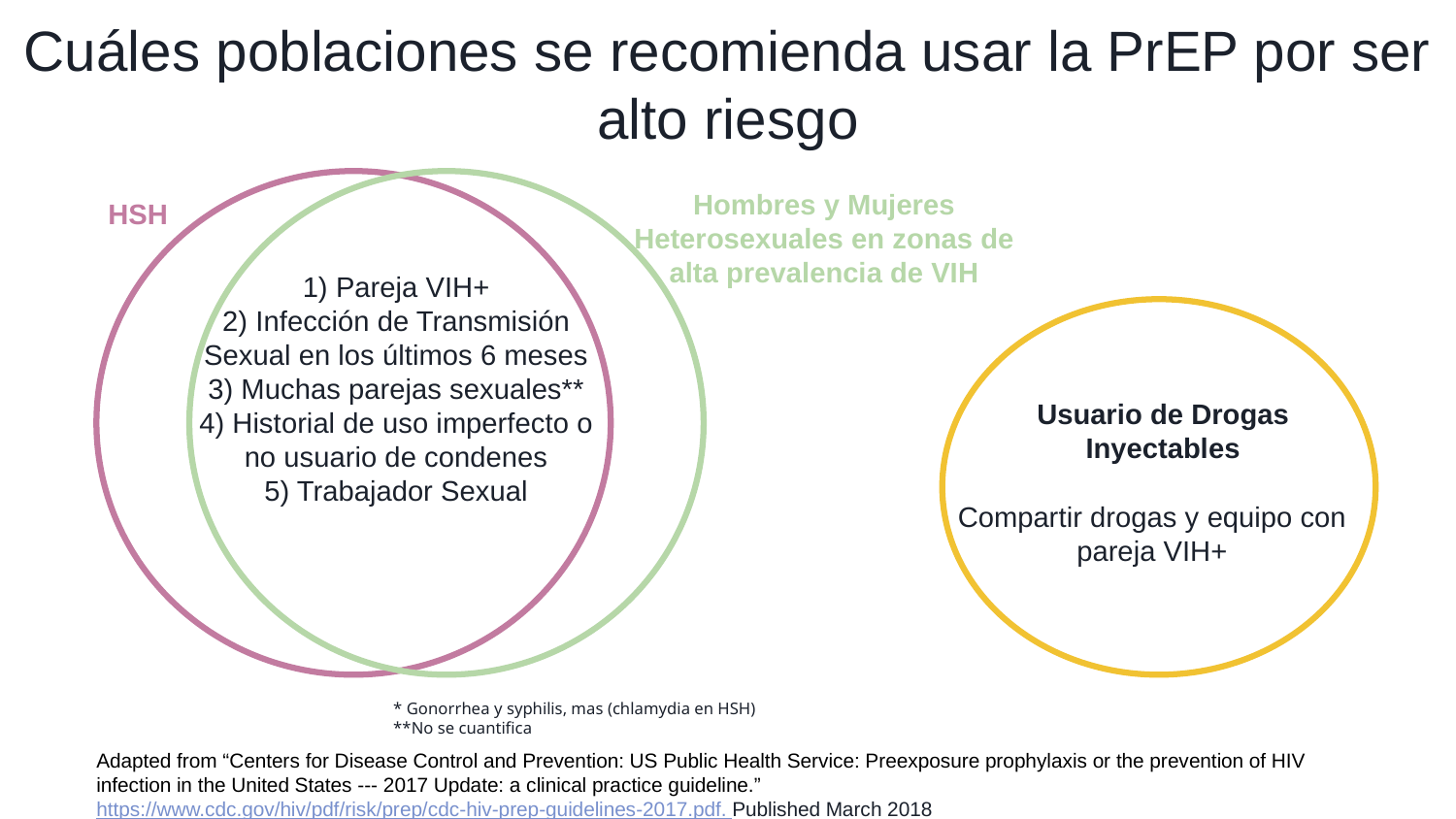

Cuáles poblaciones se recomienda usar la PrEP por ser alto riesgo
Hombres y Mujeres Heterosexuales en zonas de alta prevalencia de VIH
HSH
1) Pareja VIH+
2) Infección de Transmisión Sexual en los últimos 6 meses
3) Muchas parejas sexuales**
4) Historial de uso imperfecto o no usuario de condenes
5) Trabajador Sexual
Usuario de Drogas Inyectables
Compartir drogas y equipo con pareja VIH+
* Gonorrhea y syphilis, mas (chlamydia en HSH)
**No se cuantifica
Adapted from “Centers for Disease Control and Prevention: US Public Health Service: Preexposure prophylaxis or the prevention of HIV infection in the United States --- 2017 Update: a clinical practice guideline.” https://www.cdc.gov/hiv/pdf/risk/prep/cdc-hiv-prep-guidelines-2017.pdf. Published March 2018
18

## Slide 19
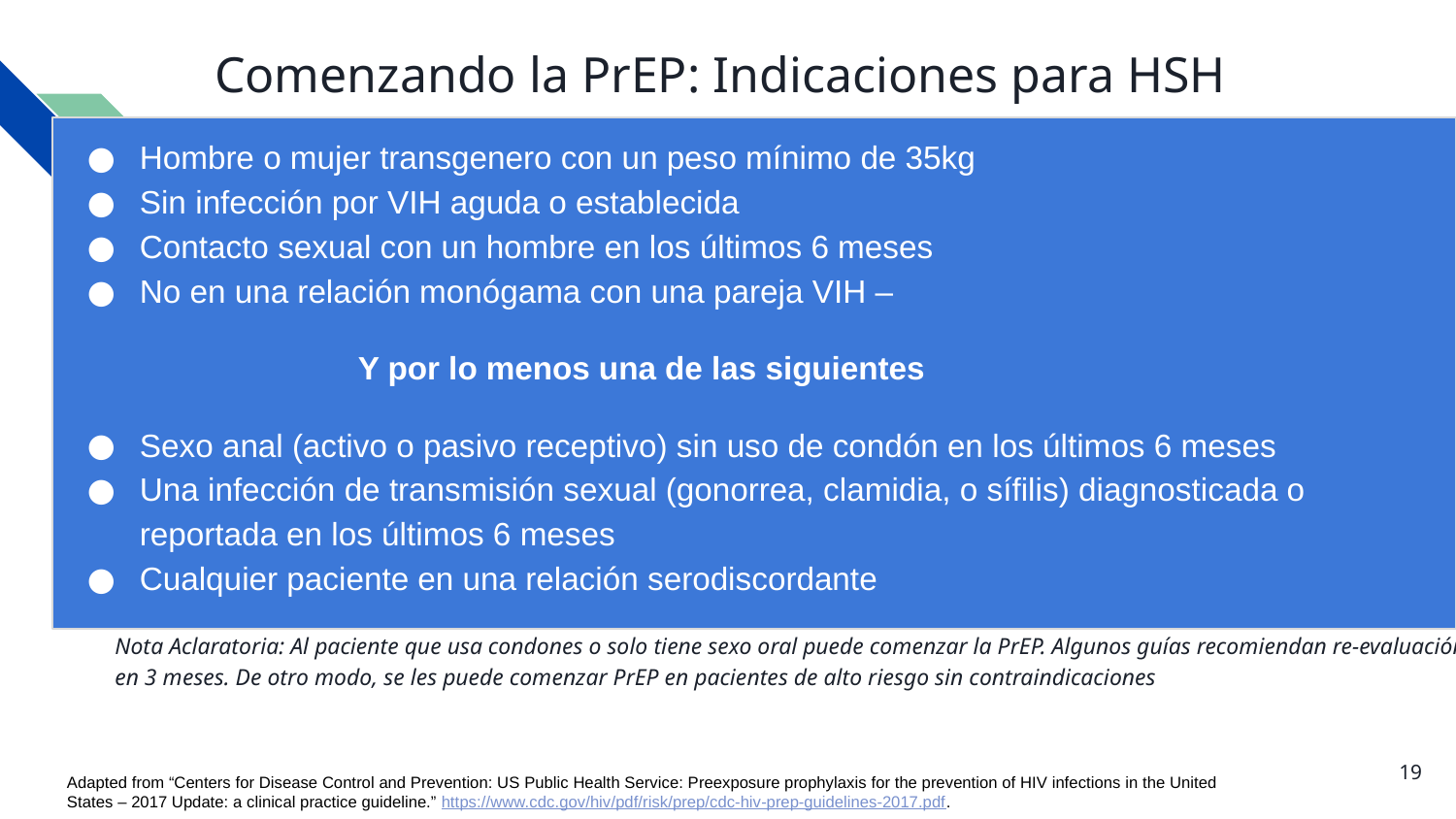

# Comenzando la PrEP: Indicaciones para HSH
Hombre o mujer transgenero con un peso mínimo de 35kg
Sin infección por VIH aguda o establecida
Contacto sexual con un hombre en los últimos 6 meses
No en una relación monógama con una pareja VIH –
Y por lo menos una de las siguientes
Sexo anal (activo o pasivo receptivo) sin uso de condón en los últimos 6 meses
Una infección de transmisión sexual (gonorrea, clamidia, o sífilis) diagnosticada o reportada en los últimos 6 meses
Cualquier paciente en una relación serodiscordante
Nota Aclaratoria: Al paciente que usa condones o solo tiene sexo oral puede comenzar la PrEP. Algunos guías recomiendan re-evaluación en 3 meses. De otro modo, se les puede comenzar PrEP en pacientes de alto riesgo sin contraindicaciones
19
Adapted from “Centers for Disease Control and Prevention: US Public Health Service: Preexposure prophylaxis for the prevention of HIV infections in the United States – 2017 Update: a clinical practice guideline.” https://www.cdc.gov/hiv/pdf/risk/prep/cdc-hiv-prep-guidelines-2017.pdf.

## Slide 20
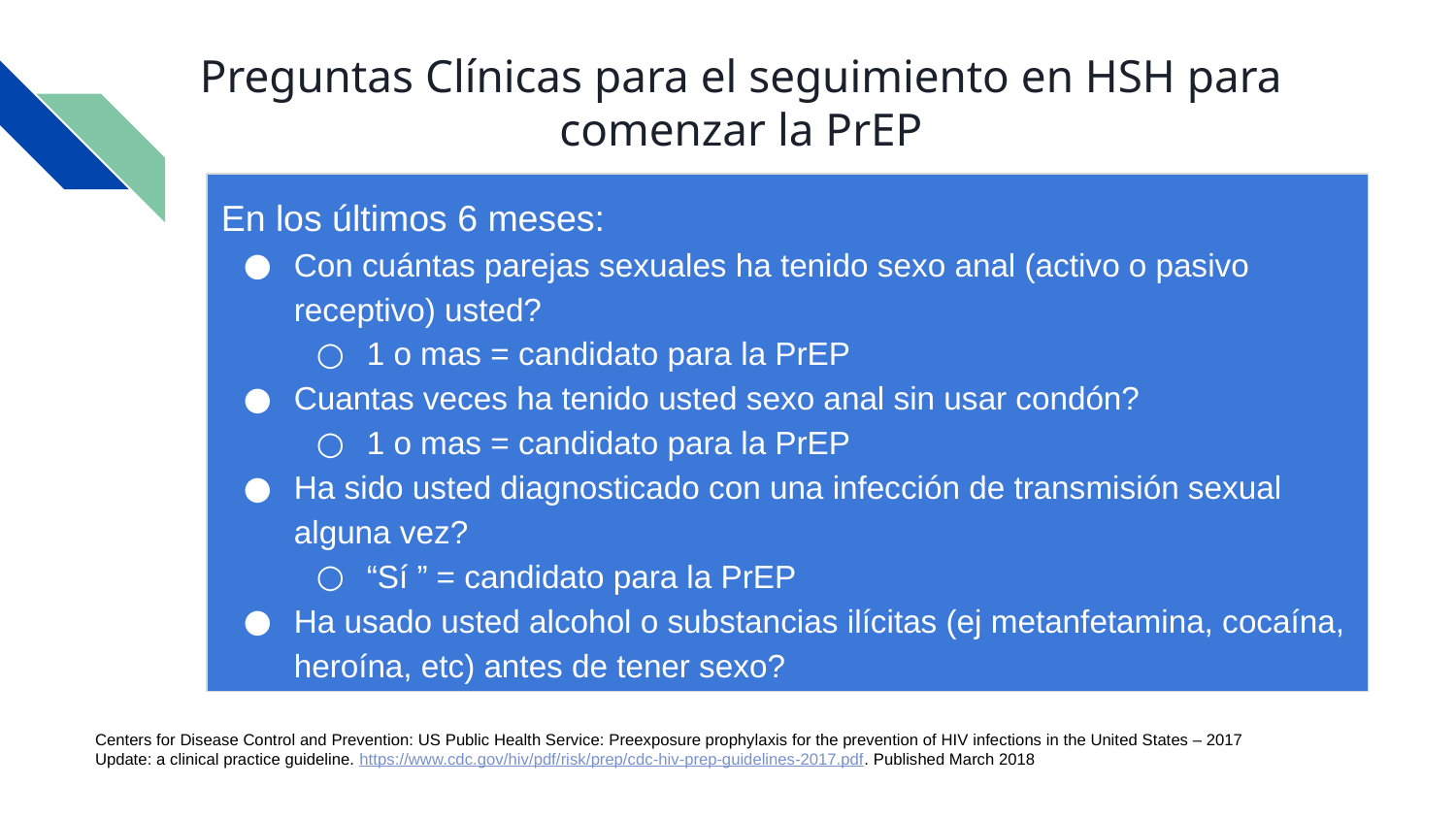

# Preguntas Clínicas para el seguimiento en HSH para comenzar la PrEP
En los últimos 6 meses:
Con cuántas parejas sexuales ha tenido sexo anal (activo o pasivo receptivo) usted?
1 o mas = candidato para la PrEP
Cuantas veces ha tenido usted sexo anal sin usar condón?
1 o mas = candidato para la PrEP
Ha sido usted diagnosticado con una infección de transmisión sexual alguna vez?
“Sí ” = candidato para la PrEP
Ha usado usted alcohol o substancias ilícitas (ej metanfetamina, cocaína, heroína, etc) antes de tener sexo?
 ““Si” = candidato para la PrEP
Centers for Disease Control and Prevention: US Public Health Service: Preexposure prophylaxis for the prevention of HIV infections in the United States – 2017 Update: a clinical practice guideline. https://www.cdc.gov/hiv/pdf/risk/prep/cdc-hiv-prep-guidelines-2017.pdf. Published March 2018
20

## Slide 21
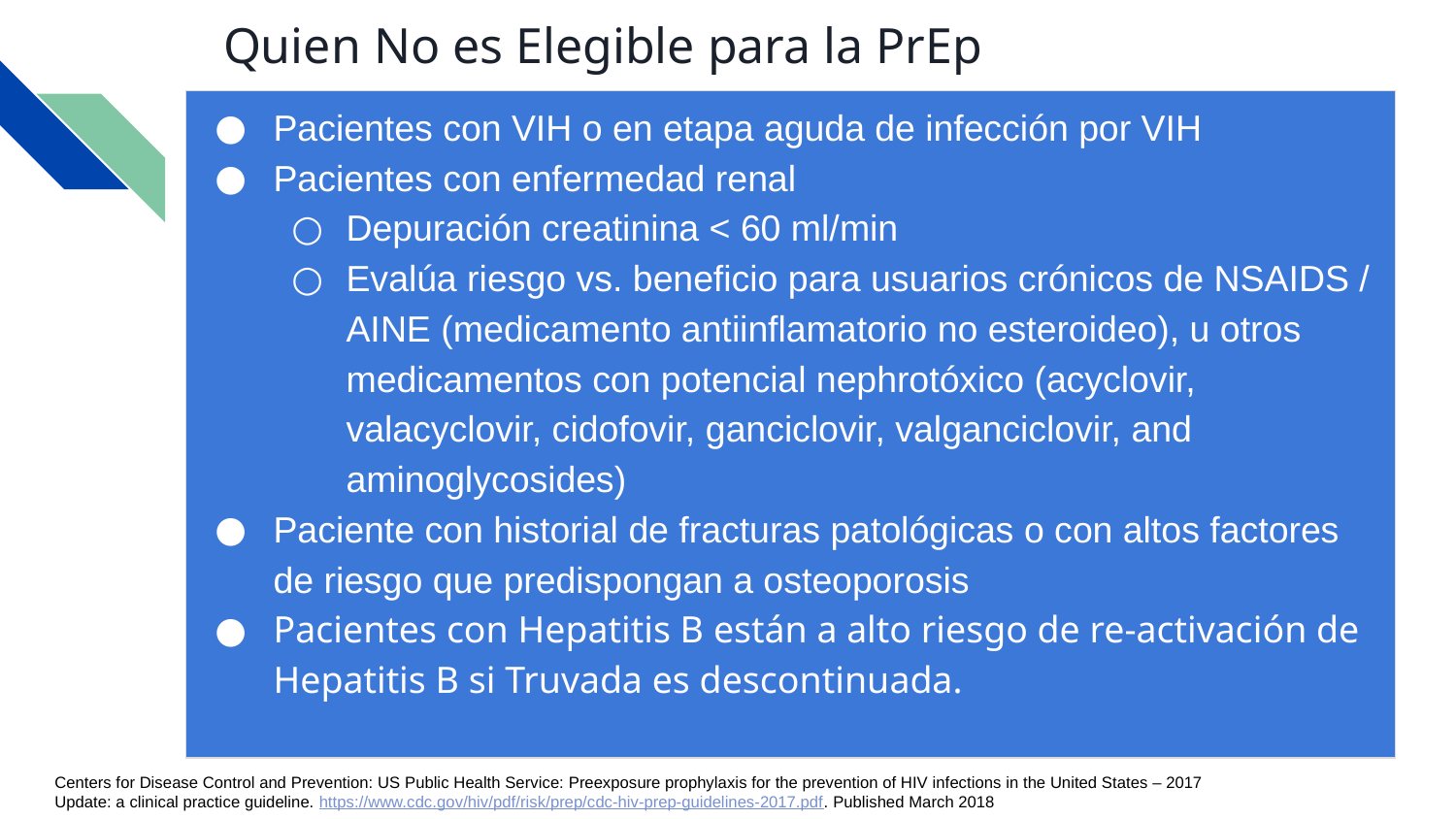

# Quien No es Elegible para la PrEp
Pacientes con VIH o en etapa aguda de infección por VIH
Pacientes con enfermedad renal
Depuración creatinina < 60 ml/min
Evalúa riesgo vs. beneficio para usuarios crónicos de NSAIDS / AINE (medicamento antiinflamatorio no esteroideo), u otros medicamentos con potencial nephrotóxico (acyclovir, valacyclovir, cidofovir, ganciclovir, valganciclovir, and aminoglycosides)
Paciente con historial de fracturas patológicas o con altos factores de riesgo que predispongan a osteoporosis
Pacientes con Hepatitis B están a alto riesgo de re-activación de Hepatitis B si Truvada es descontinuada.
21
Centers for Disease Control and Prevention: US Public Health Service: Preexposure prophylaxis for the prevention of HIV infections in the United States – 2017 Update: a clinical practice guideline. https://www.cdc.gov/hiv/pdf/risk/prep/cdc-hiv-prep-guidelines-2017.pdf. Published March 2018

## Slide 22
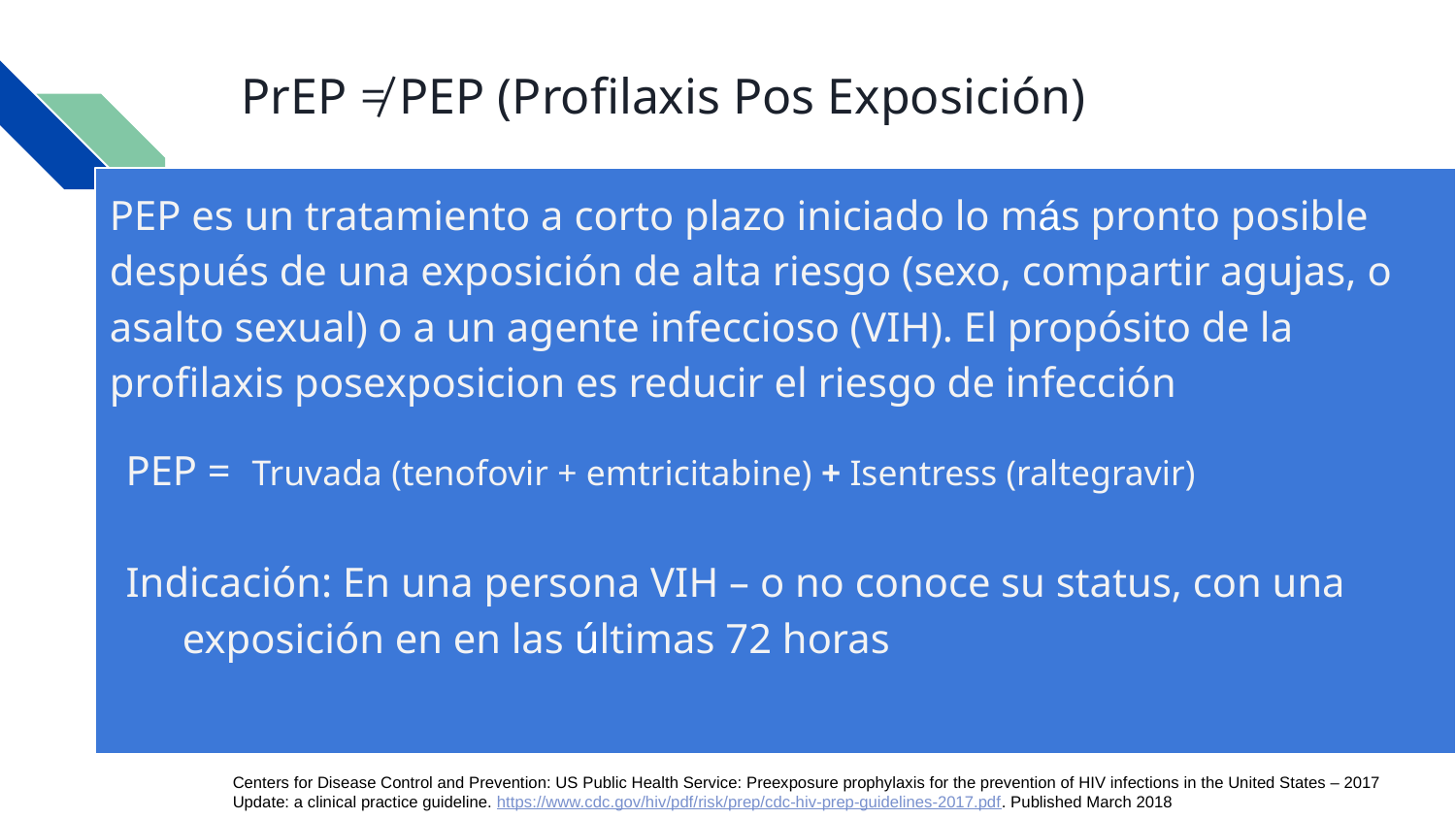

# PrEP ≠ PEP (Profilaxis Pos Exposición)
PEP es un tratamiento a corto plazo iniciado lo más pronto posible después de una exposición de alta riesgo (sexo, compartir agujas, o asalto sexual) o a un agente infeccioso (VIH). El propósito de la profilaxis posexposicion es reducir el riesgo de infección
PEP = Truvada (tenofovir + emtricitabine) + Isentress (raltegravir)
Indicación: En una persona VIH – o no conoce su status, con una exposición en en las últimas 72 horas
22
Centers for Disease Control and Prevention: US Public Health Service: Preexposure prophylaxis for the prevention of HIV infections in the United States – 2017 Update: a clinical practice guideline. https://www.cdc.gov/hiv/pdf/risk/prep/cdc-hiv-prep-guidelines-2017.pdf. Published March 2018

## Slide 23
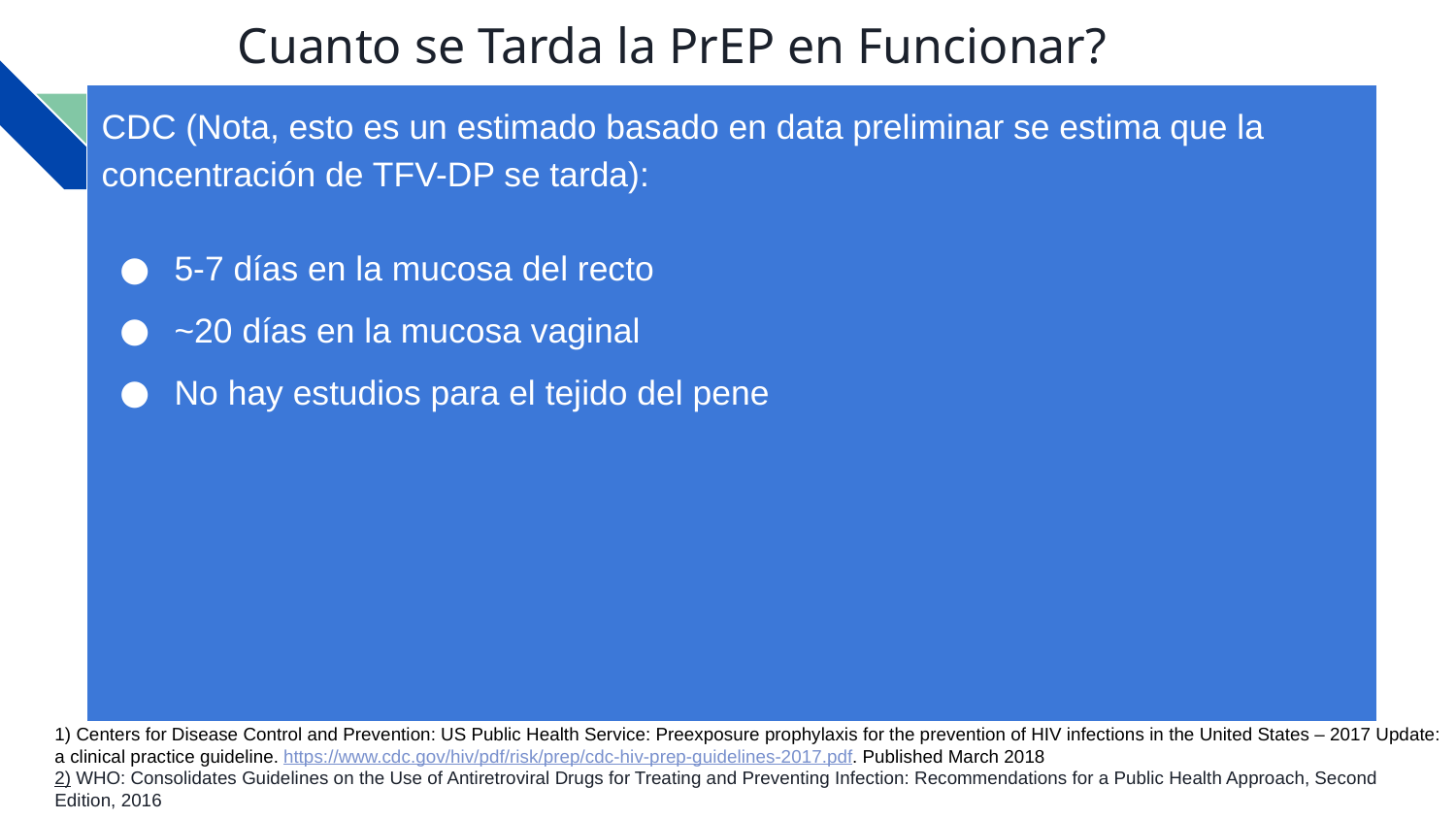

# Cuanto se Tarda la PrEP en Funcionar?
CDC (Nota, esto es un estimado basado en data preliminar se estima que la concentración de TFV-DP se tarda):
5-7 días en la mucosa del recto
~20 días en la mucosa vaginal
No hay estudios para el tejido del pene
1) Centers for Disease Control and Prevention: US Public Health Service: Preexposure prophylaxis for the prevention of HIV infections in the United States – 2017 Update: a clinical practice guideline. https://www.cdc.gov/hiv/pdf/risk/prep/cdc-hiv-prep-guidelines-2017.pdf. Published March 2018
2) WHO: Consolidates Guidelines on the Use of Antiretroviral Drugs for Treating and Preventing Infection: Recommendations for a Public Health Approach, Second Edition, 2016
23

## Slide 24
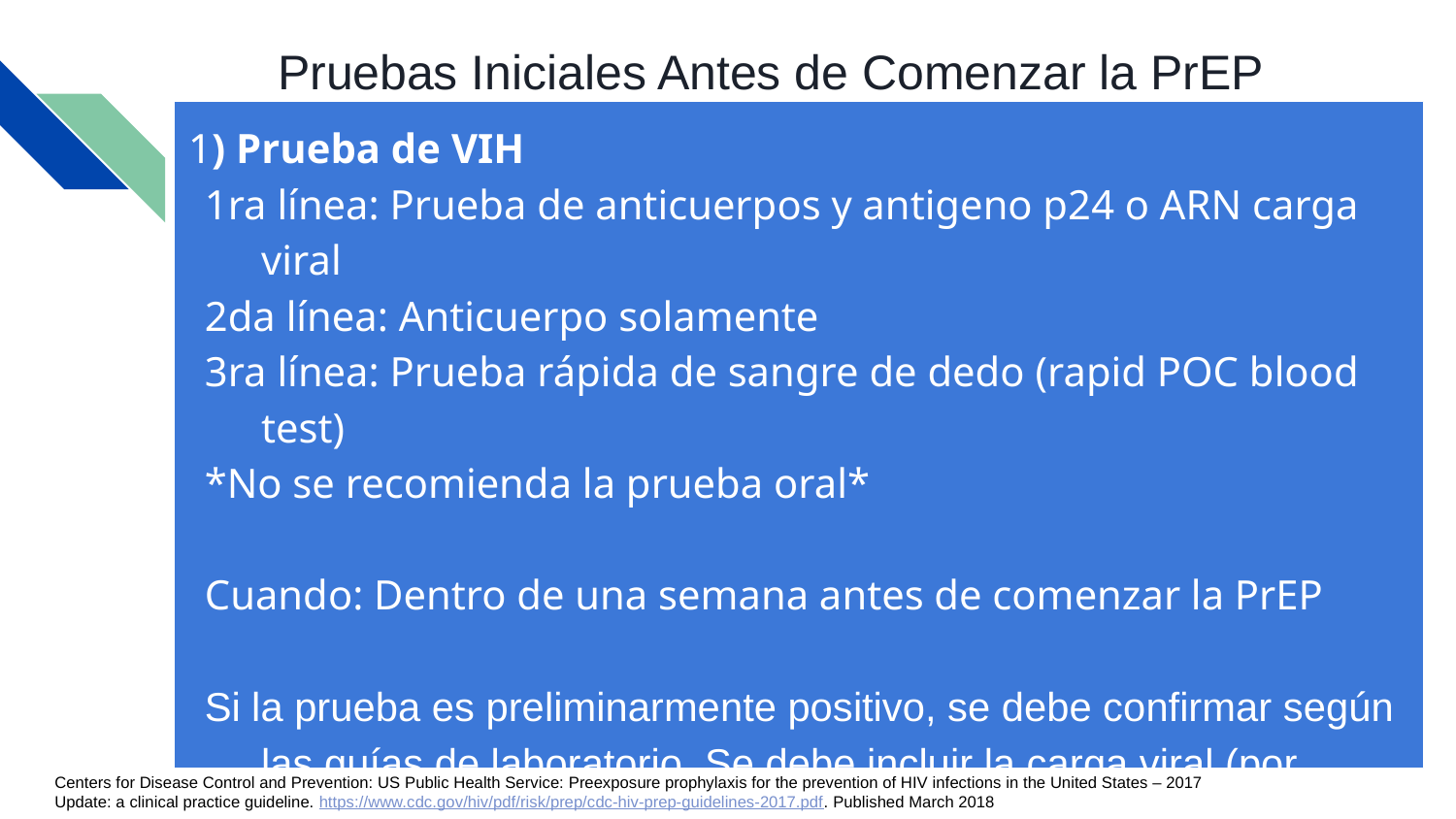

# Pruebas Iniciales Antes de Comenzar la PrEP
1) Prueba de VIH
1ra línea: Prueba de anticuerpos y antigeno p24 o ARN carga viral
2da línea: Anticuerpo solamente
3ra línea: Prueba rápida de sangre de dedo (rapid POC blood test)
*No se recomienda la prueba oral*
Cuando: Dentro de una semana antes de comenzar la PrEP
Si la prueba es preliminarmente positivo, se debe confirmar según las guías de laboratorio. Se debe incluir la carga viral (por pruebas de acido nucleico (NAT)) y conteo de linfocitos CD4+
24
Centers for Disease Control and Prevention: US Public Health Service: Preexposure prophylaxis for the prevention of HIV infections in the United States – 2017 Update: a clinical practice guideline. https://www.cdc.gov/hiv/pdf/risk/prep/cdc-hiv-prep-guidelines-2017.pdf. Published March 2018

## Slide 25
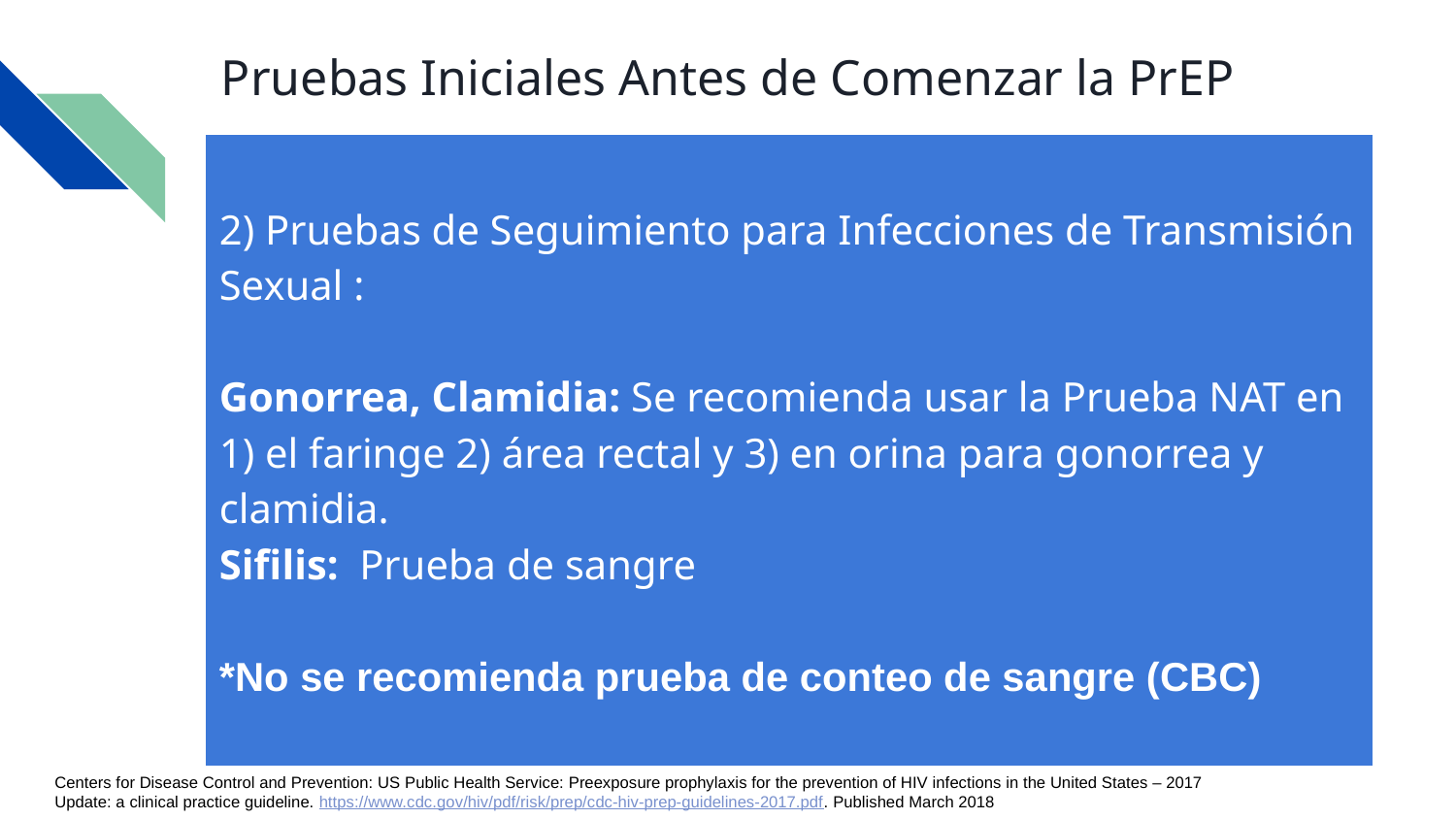

# Pruebas Iniciales Antes de Comenzar la PrEP
2) Pruebas de Seguimiento para Infecciones de Transmisión Sexual :
Gonorrea, Clamidia: Se recomienda usar la Prueba NAT en 1) el faringe 2) área rectal y 3) en orina para gonorrea y clamidia.
Sifilis: Prueba de sangre
*No se recomienda prueba de conteo de sangre (CBC)
25
Centers for Disease Control and Prevention: US Public Health Service: Preexposure prophylaxis for the prevention of HIV infections in the United States – 2017 Update: a clinical practice guideline. https://www.cdc.gov/hiv/pdf/risk/prep/cdc-hiv-prep-guidelines-2017.pdf. Published March 2018

## Slide 26
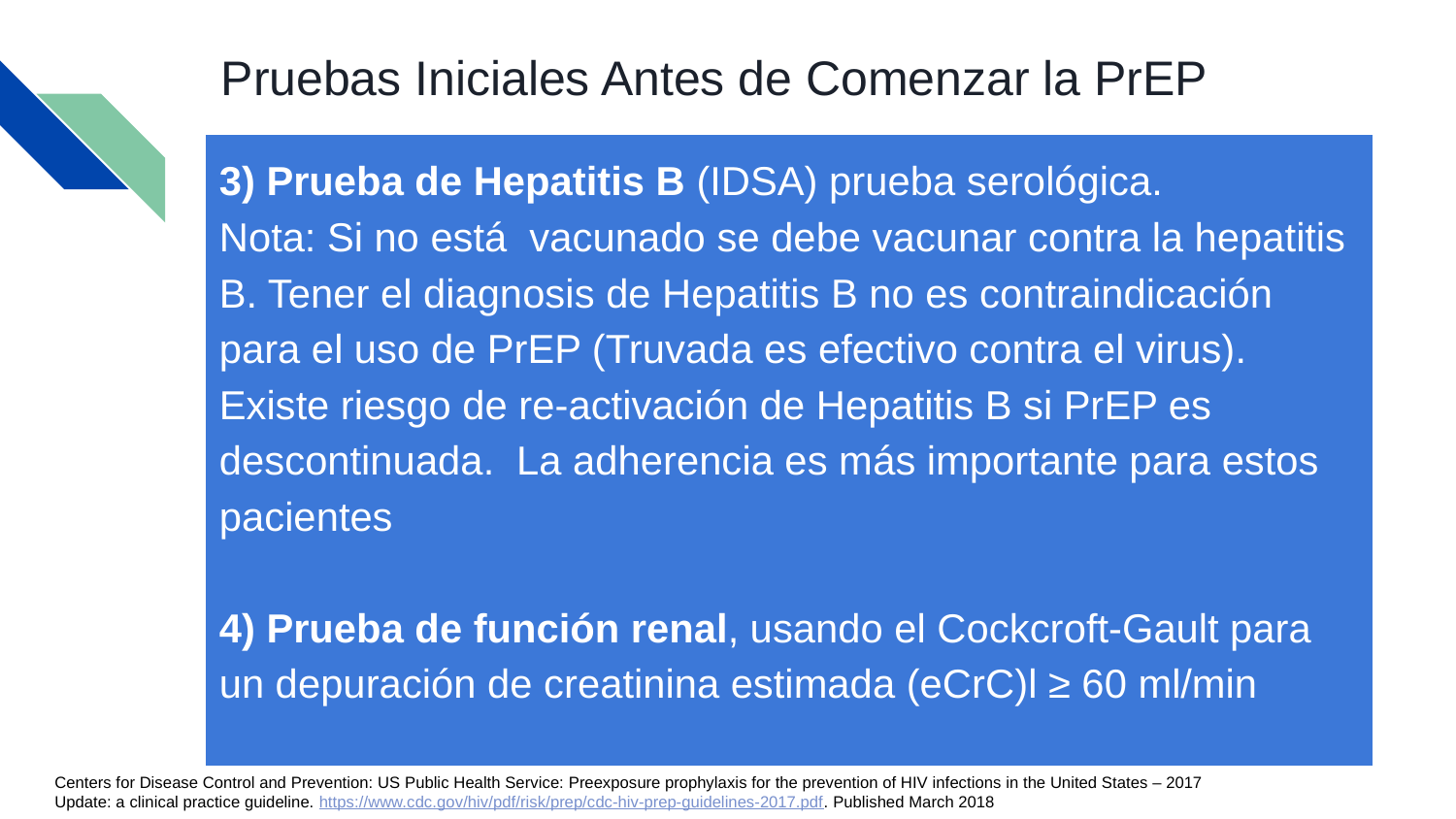

# Pruebas Iniciales Antes de Comenzar la PrEP
3) Prueba de Hepatitis B (IDSA) prueba serológica.
Nota: Si no está vacunado se debe vacunar contra la hepatitis B. Tener el diagnosis de Hepatitis B no es contraindicación para el uso de PrEP (Truvada es efectivo contra el virus). Existe riesgo de re-activación de Hepatitis B si PrEP es descontinuada. La adherencia es más importante para estos pacientes
4) Prueba de función renal, usando el Cockcroft-Gault para un depuración de creatinina estimada (eCrC)l ≥ 60 ml/min
26
Centers for Disease Control and Prevention: US Public Health Service: Preexposure prophylaxis for the prevention of HIV infections in the United States – 2017 Update: a clinical practice guideline. https://www.cdc.gov/hiv/pdf/risk/prep/cdc-hiv-prep-guidelines-2017.pdf. Published March 2018

## Slide 27
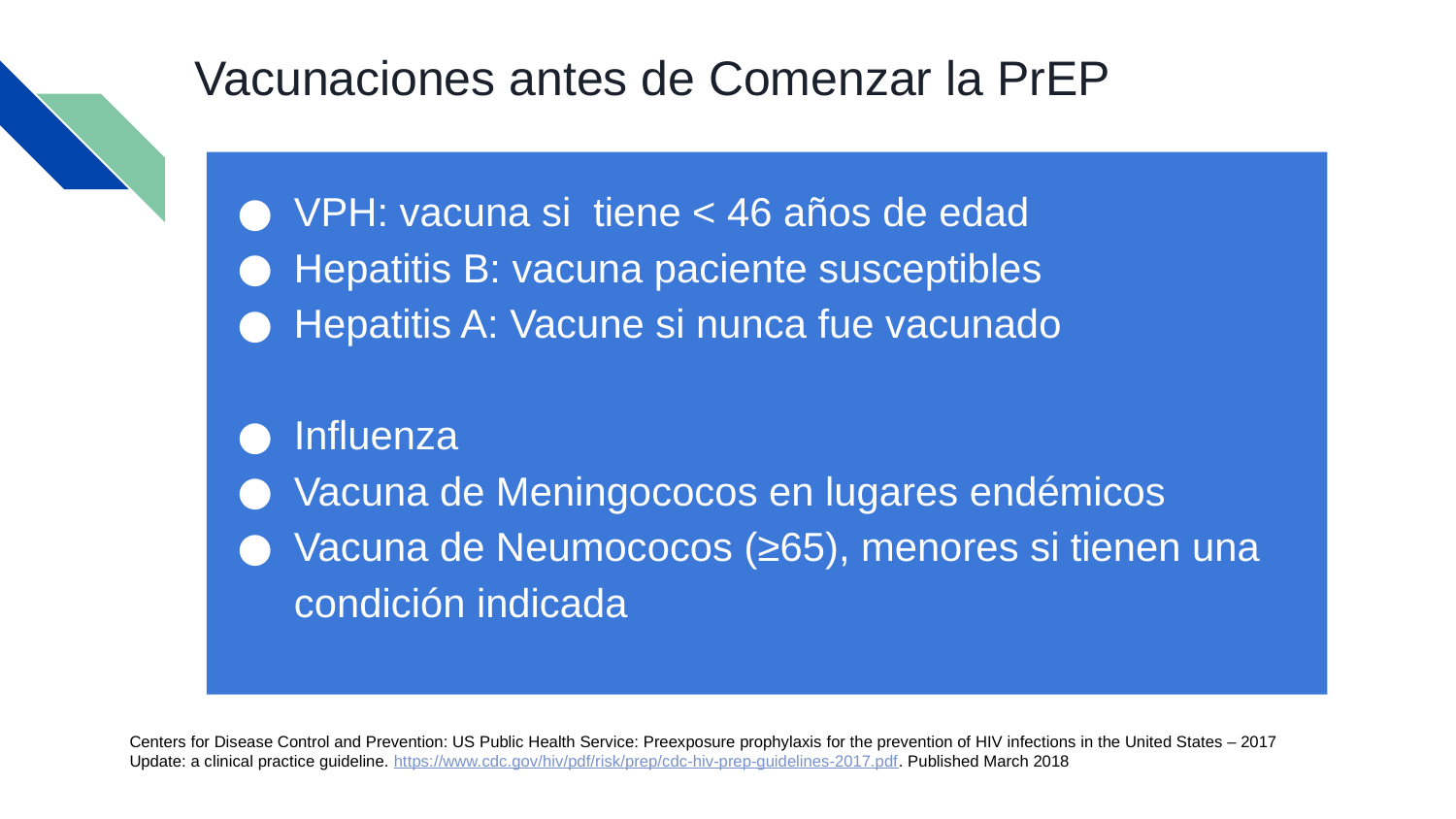

# Vacunaciones antes de Comenzar la PrEP
VPH: vacuna si tiene < 46 años de edad
Hepatitis B: vacuna paciente susceptibles
Hepatitis A: Vacune si nunca fue vacunado
Influenza
Vacuna de Meningococos en lugares endémicos
Vacuna de Neumococos (≥65), menores si tienen una condición indicada
Centers for Disease Control and Prevention: US Public Health Service: Preexposure prophylaxis for the prevention of HIV infections in the United States – 2017 Update: a clinical practice guideline. https://www.cdc.gov/hiv/pdf/risk/prep/cdc-hiv-prep-guidelines-2017.pdf. Published March 2018
27

## Slide 28
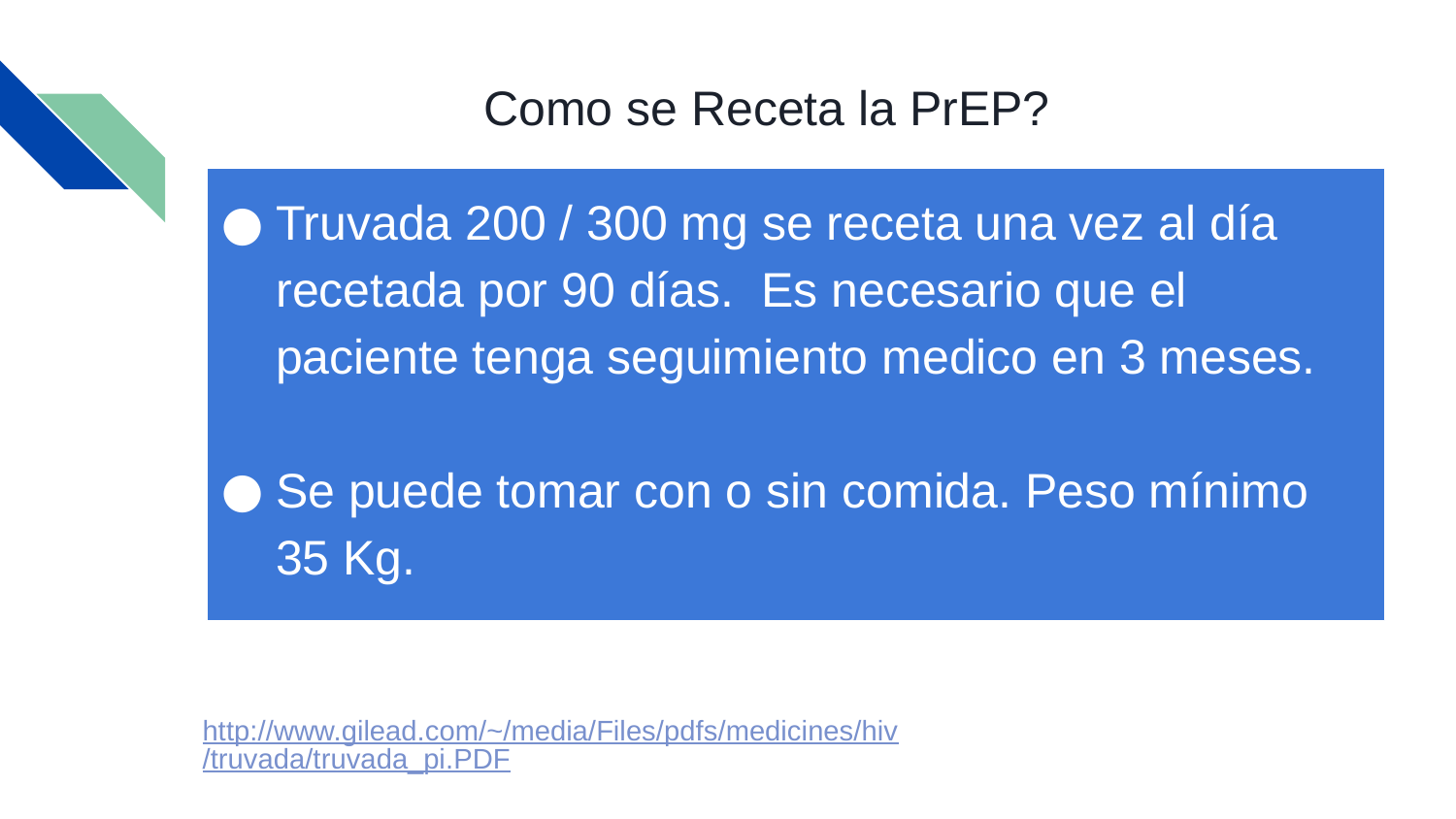

# Como se Receta la PrEP?
Truvada 200 / 300 mg se receta una vez al día recetada por 90 días. Es necesario que el paciente tenga seguimiento medico en 3 meses.
Se puede tomar con o sin comida. Peso mínimo 35 Kg.
http://www.gilead.com/~/media/Files/pdfs/medicines/hiv/truvada/truvada_pi.PDF
28

## Slide 29
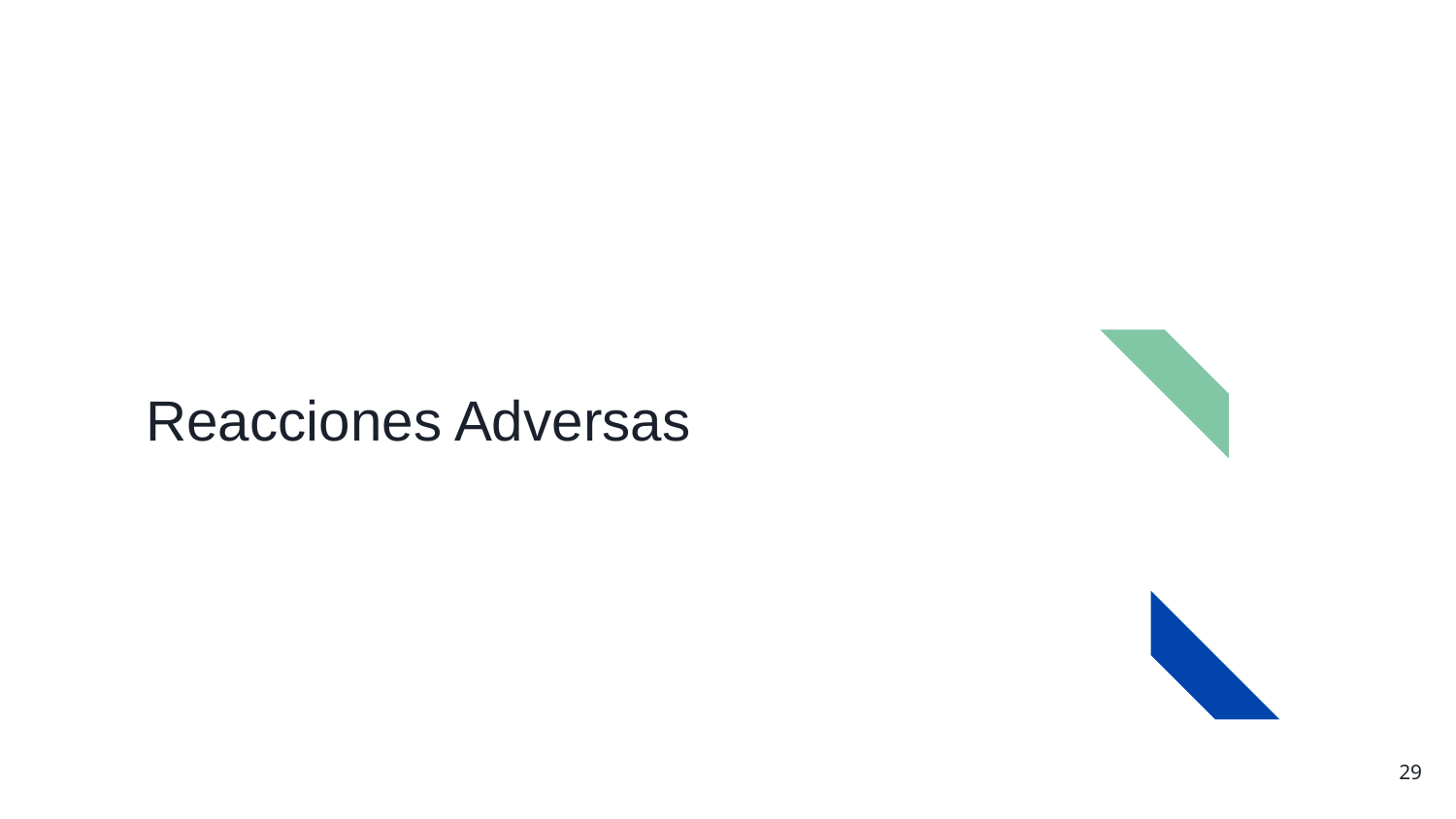

# Reacciones Adversas
29

## Slide 30
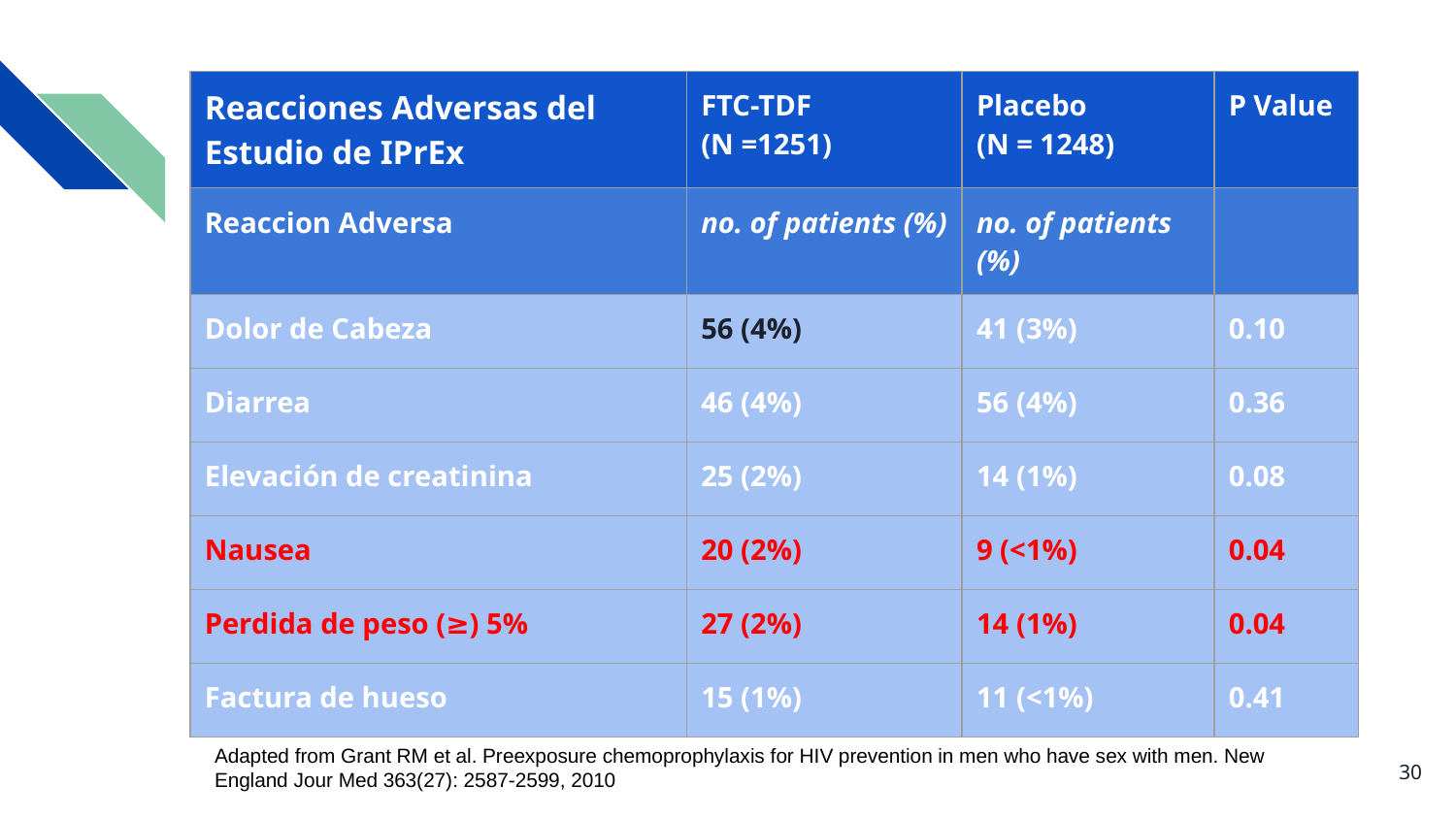

# PrEP Side Effects and Adverse Reactions
| Reacciones Adversas del Estudio de IPrEx | FTC-TDF (N =1251) | Placebo (N = 1248) | P Value |
| --- | --- | --- | --- |
| Reaccion Adversa | no. of patients (%) | no. of patients (%) | |
| Dolor de Cabeza | 56 (4%) | 41 (3%) | 0.10 |
| Diarrea | 46 (4%) | 56 (4%) | 0.36 |
| Elevación de creatinina | 25 (2%) | 14 (1%) | 0.08 |
| Nausea | 20 (2%) | 9 (<1%) | 0.04 |
| Perdida de peso (≥) 5% | 27 (2%) | 14 (1%) | 0.04 |
| Factura de hueso | 15 (1%) | 11 (<1%) | 0.41 |
Adapted from Grant RM et al. Preexposure chemoprophylaxis for HIV prevention in men who have sex with men. New England Jour Med 363(27): 2587-2599, 2010
30

## Slide 31
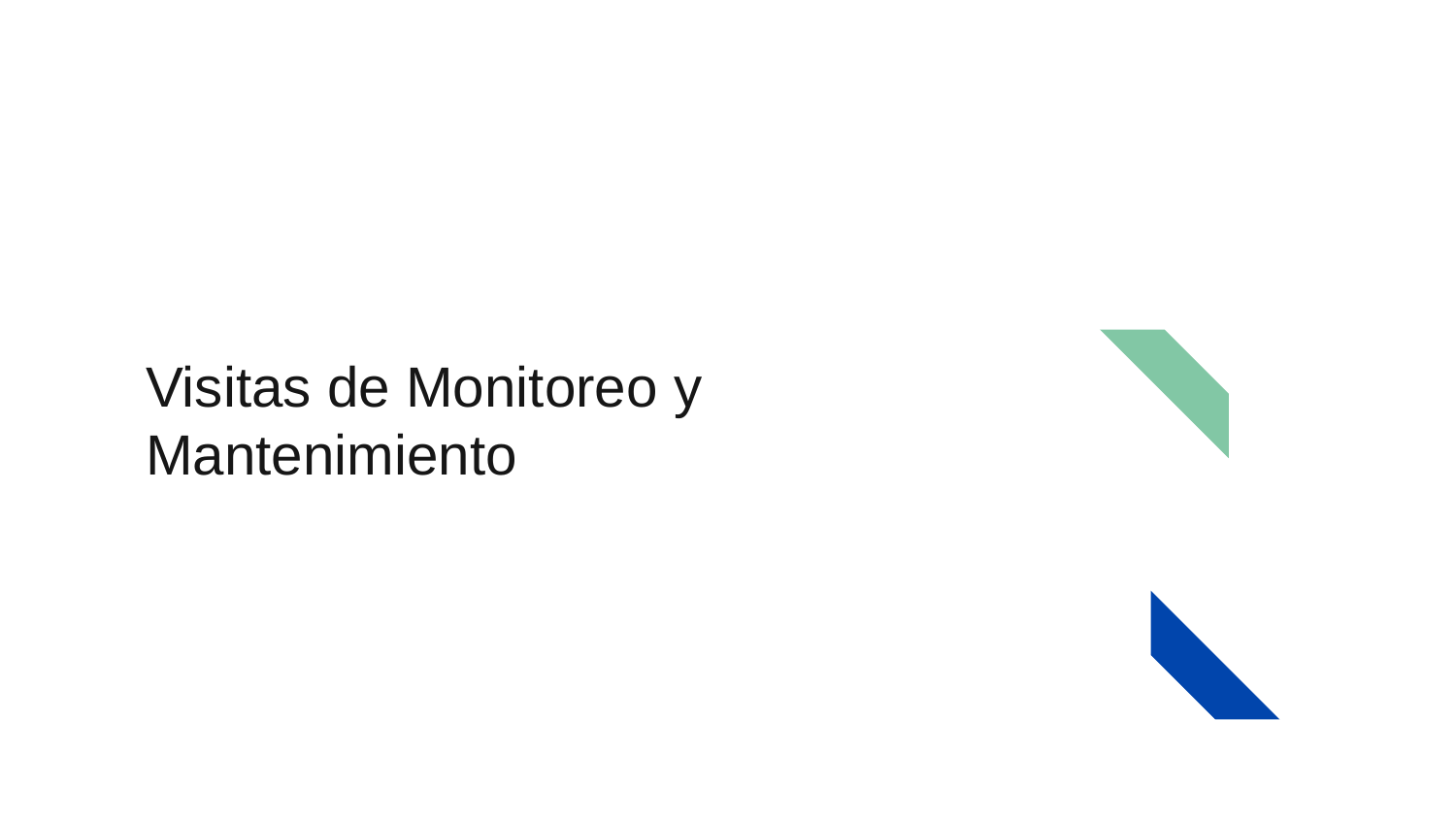

# Visitas de Monitoreo y Mantenimiento
31

## Slide 32
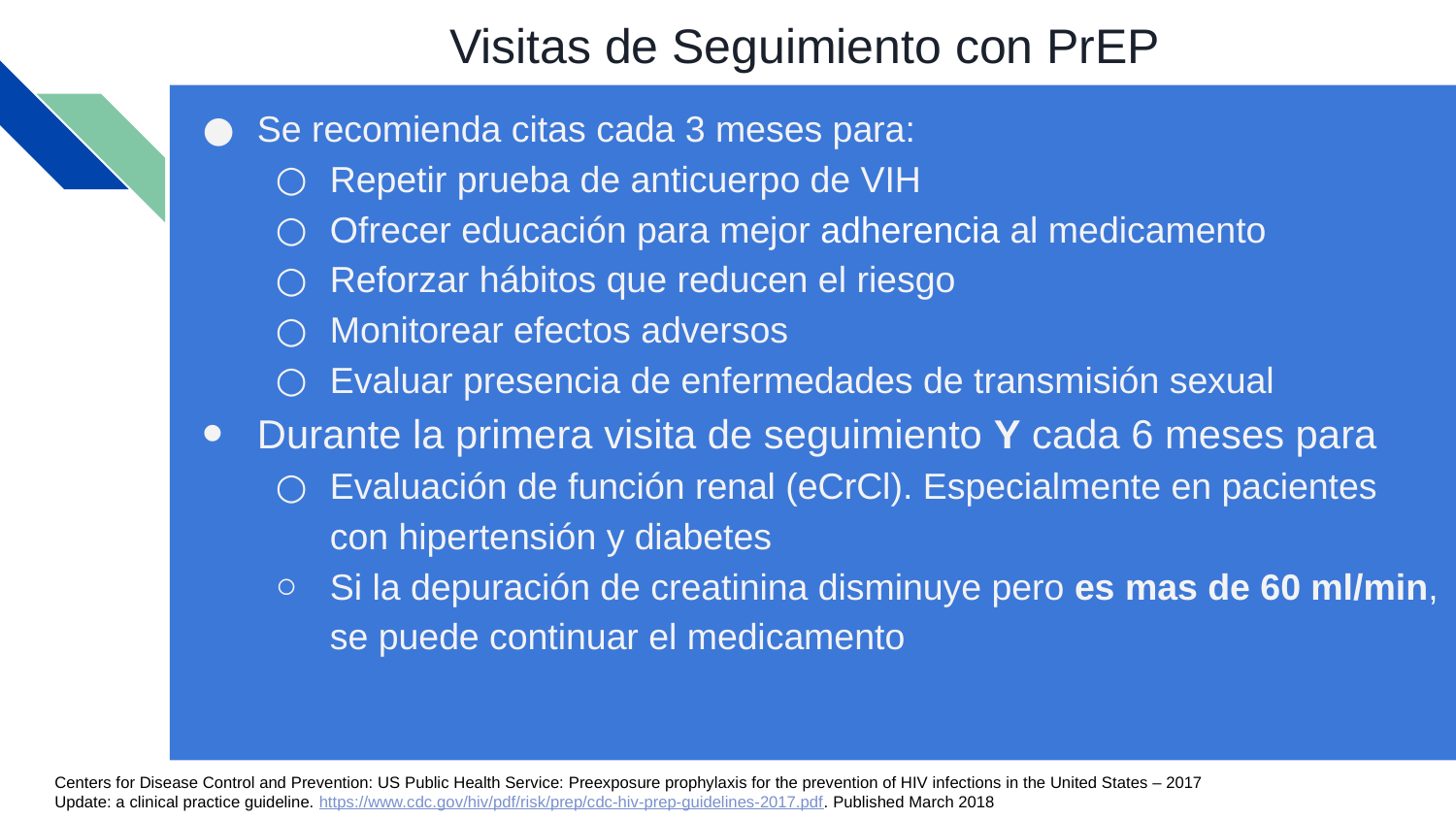

# Visitas de Seguimiento con PrEP
Se recomienda citas cada 3 meses para:
Repetir prueba de anticuerpo de VIH
Ofrecer educación para mejor adherencia al medicamento
Reforzar hábitos que reducen el riesgo
Monitorear efectos adversos
Evaluar presencia de enfermedades de transmisión sexual
Durante la primera visita de seguimiento Y cada 6 meses para
Evaluación de función renal (eCrCl). Especialmente en pacientes con hipertensión y diabetes
Si la depuración de creatinina disminuye pero es mas de 60 ml/min, se puede continuar el medicamento
32
Centers for Disease Control and Prevention: US Public Health Service: Preexposure prophylaxis for the prevention of HIV infections in the United States – 2017 Update: a clinical practice guideline. https://www.cdc.gov/hiv/pdf/risk/prep/cdc-hiv-prep-guidelines-2017.pdf. Published March 2018

## Slide 33
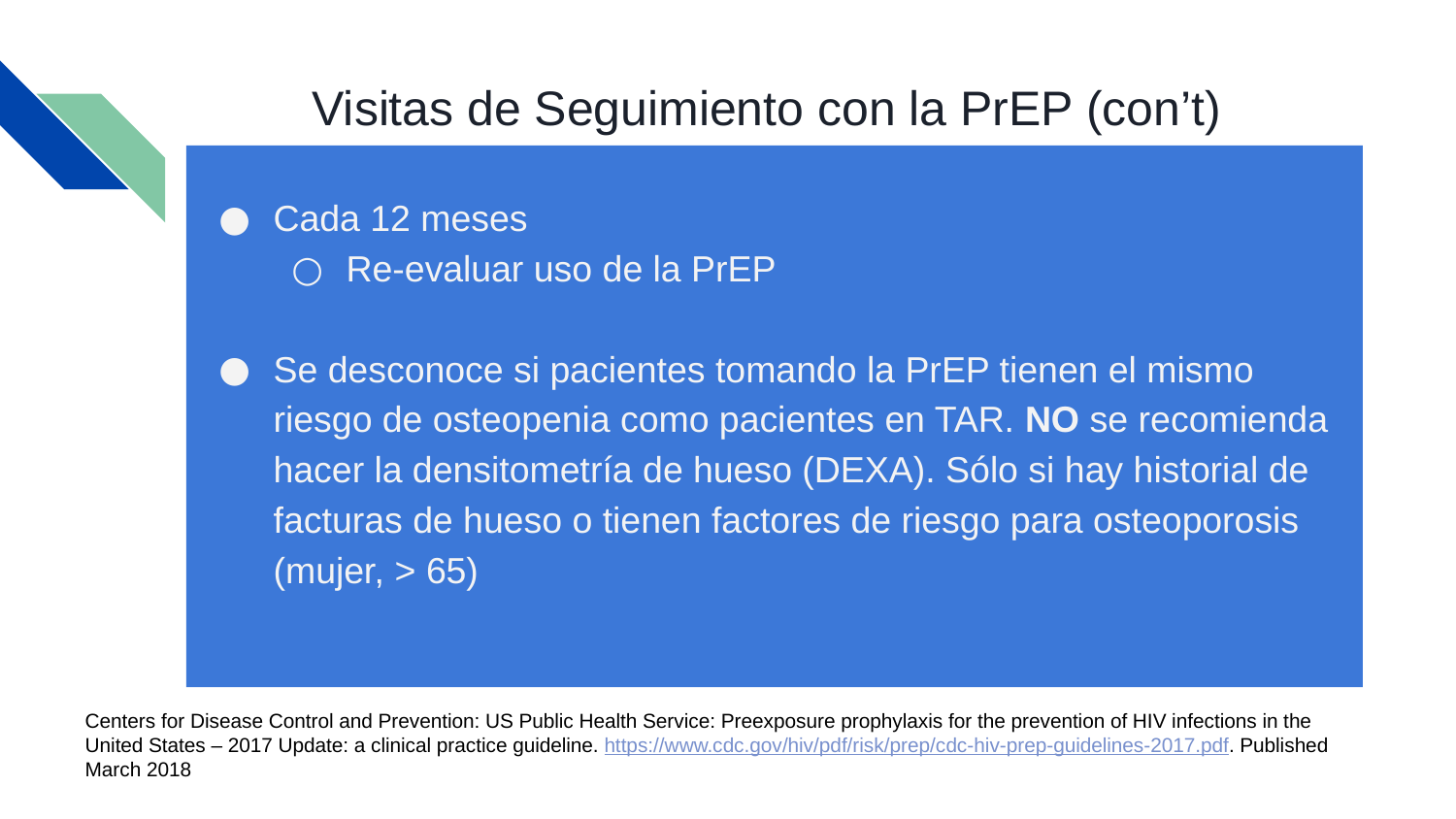

# Visitas de Seguimiento con la PrEP (con’t)
Cada 12 meses
Re-evaluar uso de la PrEP
Se desconoce si pacientes tomando la PrEP tienen el mismo riesgo de osteopenia como pacientes en TAR. NO se recomienda hacer la densitometría de hueso (DEXA). Sólo si hay historial de facturas de hueso o tienen factores de riesgo para osteoporosis (mujer, > 65)
Centers for Disease Control and Prevention: US Public Health Service: Preexposure prophylaxis for the prevention of HIV infections in the United States – 2017 Update: a clinical practice guideline. https://www.cdc.gov/hiv/pdf/risk/prep/cdc-hiv-prep-guidelines-2017.pdf. Published March 2018
33

## Slide 34
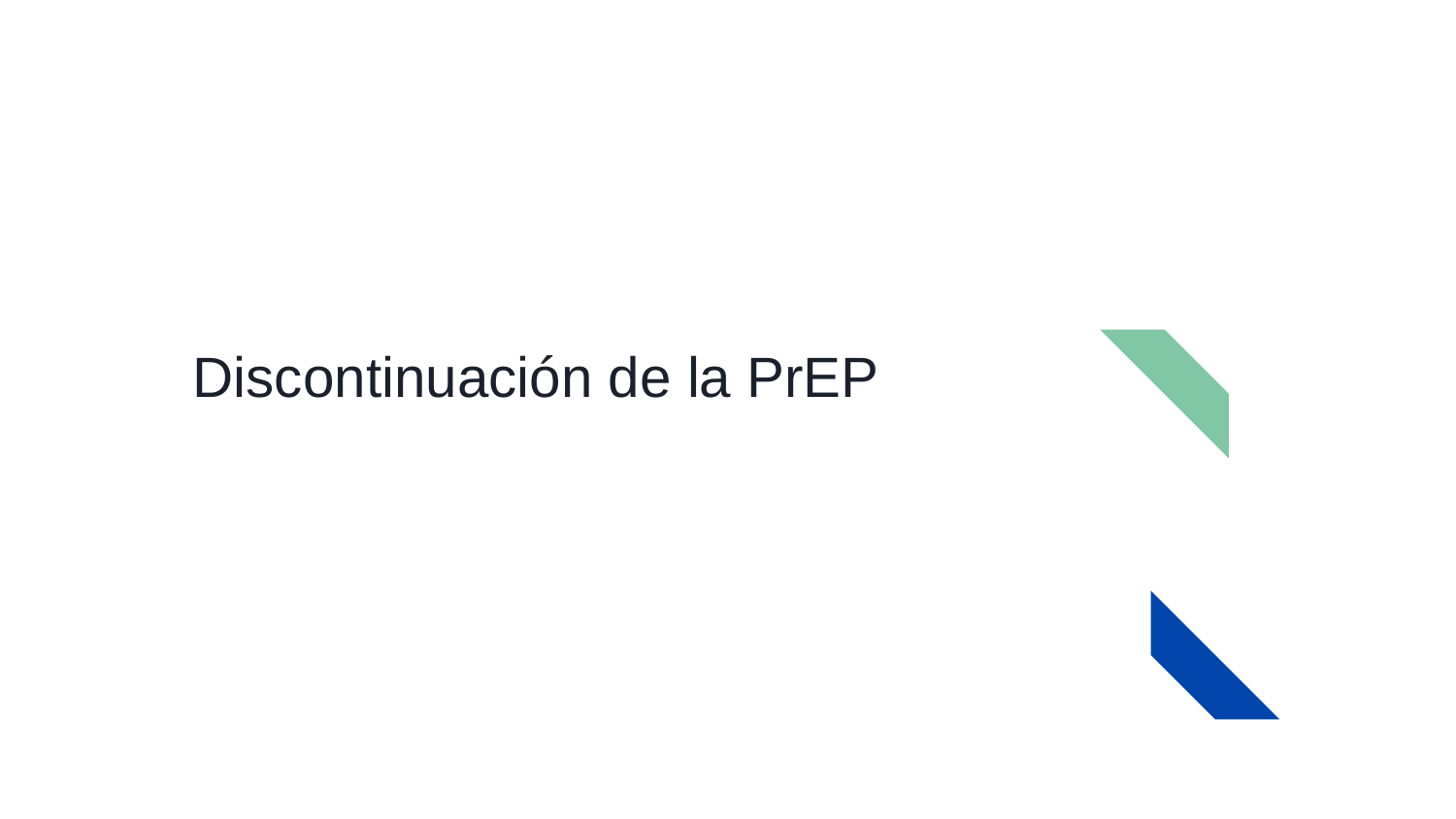

# Discontinuación de la PrEP
34

## Slide 35
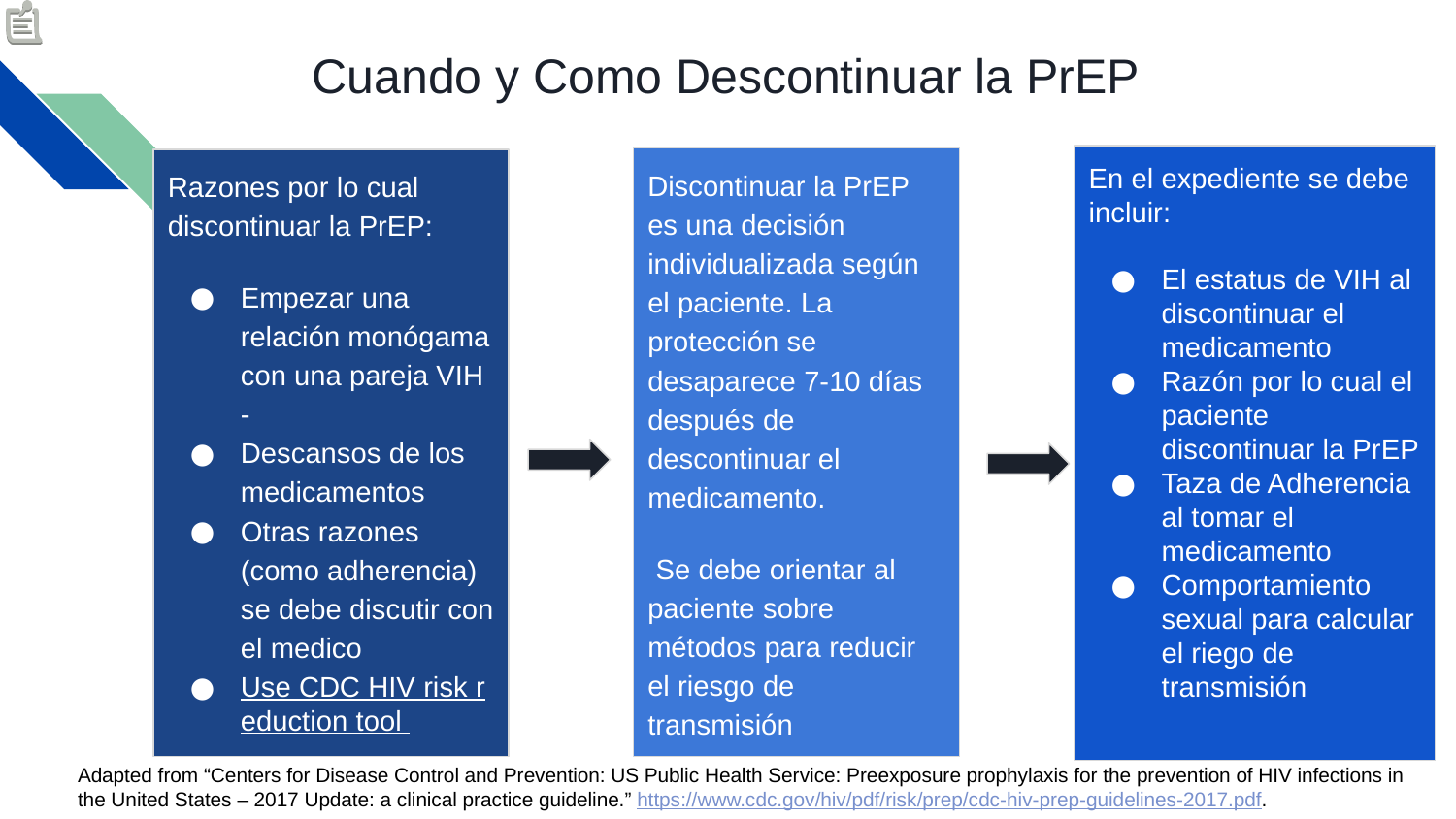

# Cuando y Como Descontinuar la PrEP
En el expediente se debe incluir:
El estatus de VIH al discontinuar el medicamento
Razón por lo cual el paciente discontinuar la PrEP
Taza de Adherencia al tomar el medicamento
Comportamiento sexual para calcular el riego de transmisión
Discontinuar la PrEP es una decisión individualizada según el paciente. La protección se desaparece 7-10 días después de descontinuar el medicamento.
 Se debe orientar al paciente sobre métodos para reducir el riesgo de transmisión
Razones por lo cual discontinuar la PrEP:
Empezar una relación monógama con una pareja VIH -
Descansos de los medicamentos
Otras razones (como adherencia) se debe discutir con el medico
Use CDC HIV risk reduction tool
35
Adapted from “Centers for Disease Control and Prevention: US Public Health Service: Preexposure prophylaxis for the prevention of HIV infections in the United States – 2017 Update: a clinical practice guideline.” https://www.cdc.gov/hiv/pdf/risk/prep/cdc-hiv-prep-guidelines-2017.pdf.

## Slide 36
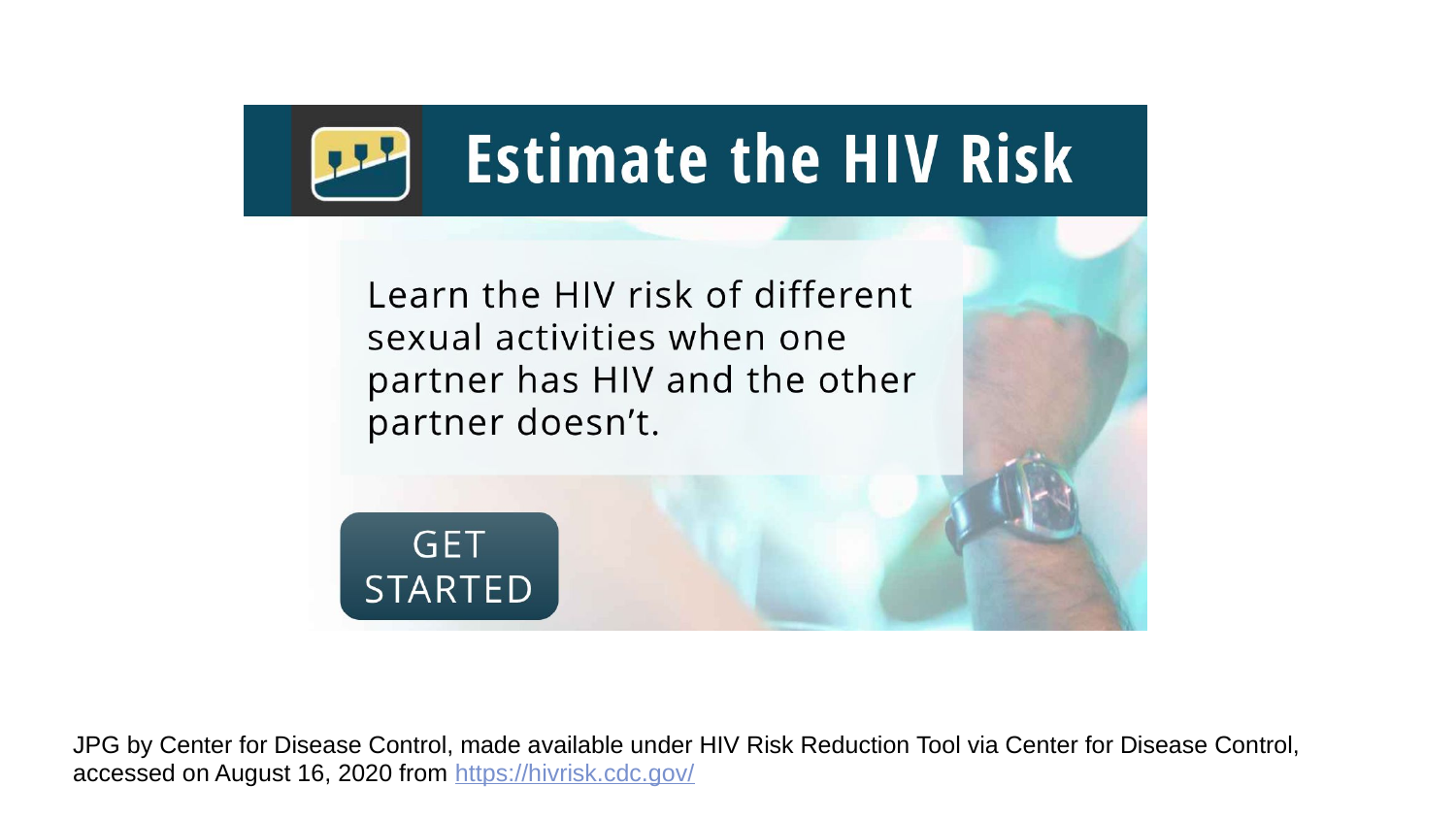

Haga Clic Aqui
JPG by Center for Disease Control, made available under HIV Risk Reduction Tool via Center for Disease Control, accessed on August 16, 2020 from https://hivrisk.cdc.gov/
36

## Slide 37
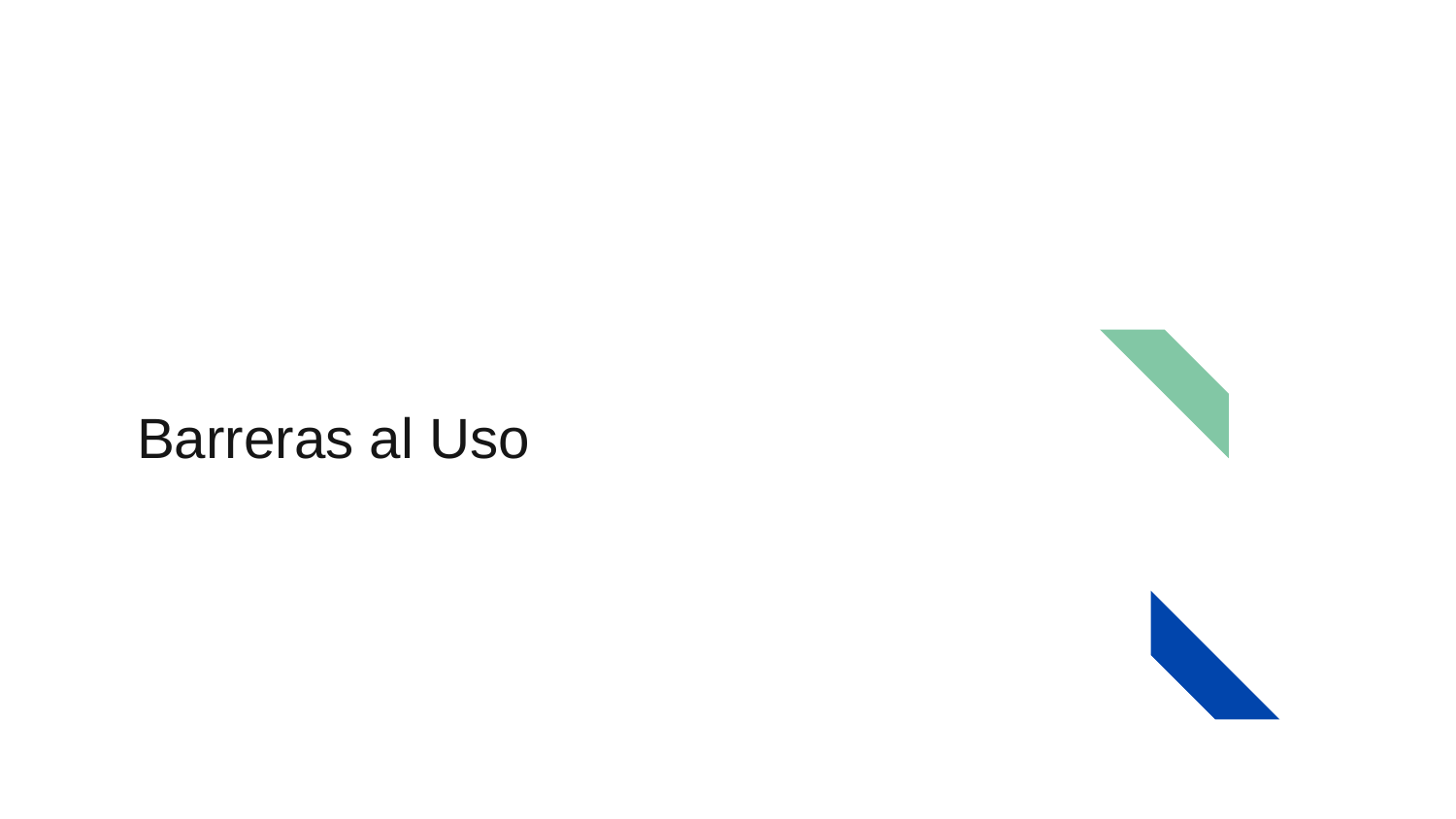

# Barreras al Uso
37

## Slide 38
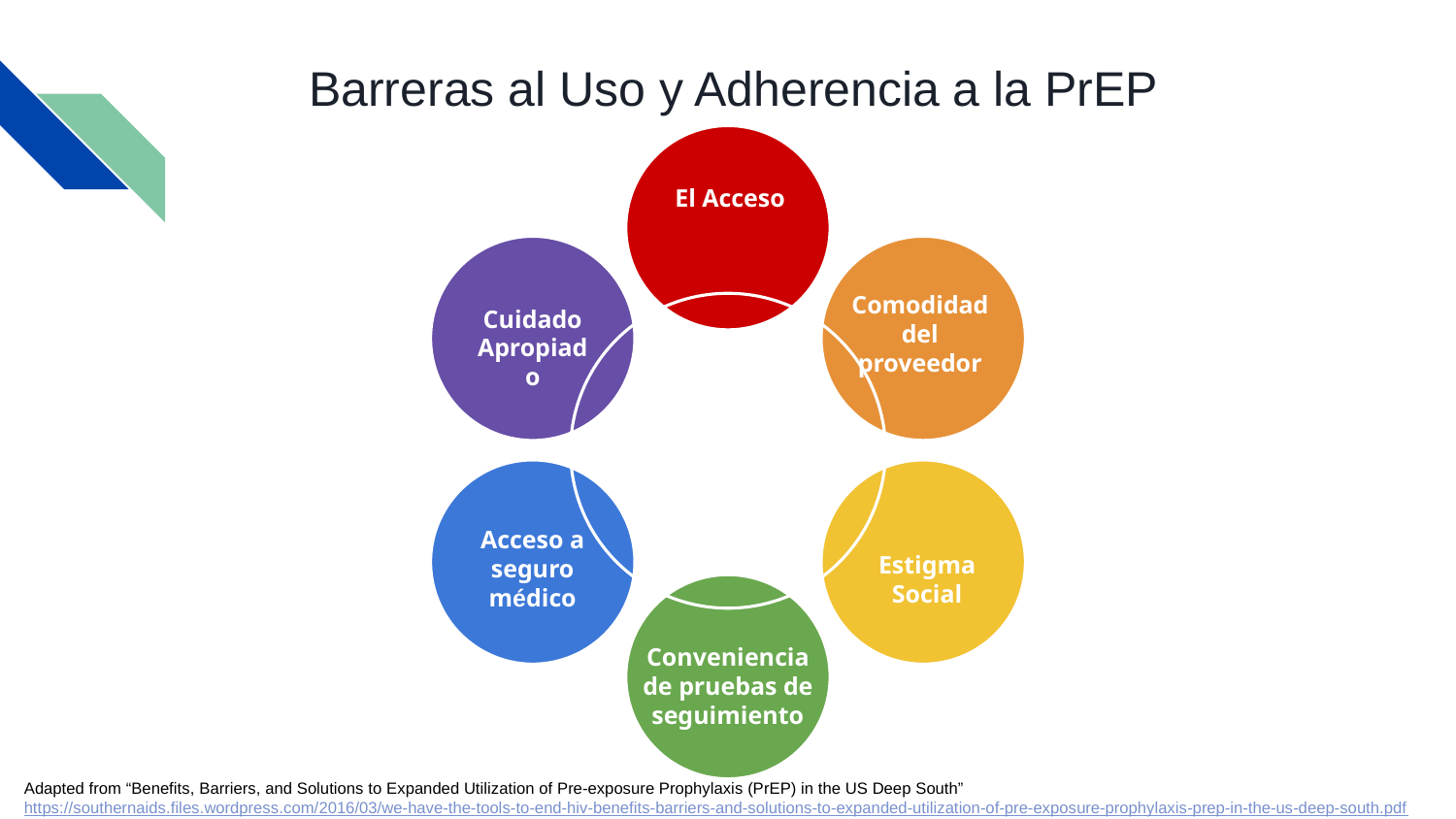

# Barreras al Uso y Adherencia a la PrEP
El Acceso
Comodidad del
proveedor
Cuidado Apropiado
Uptake and Adherence to PrEP
Estigma Social
Acceso a seguro médico
Conveniencia de pruebas de seguimiento
38
Adapted from “Benefits, Barriers, and Solutions to Expanded Utilization of Pre-exposure Prophylaxis (PrEP) in the US Deep South” https://southernaids.files.wordpress.com/2016/03/we-have-the-tools-to-end-hiv-benefits-barriers-and-solutions-to-expanded-utilization-of-pre-exposure-prophylaxis-prep-in-the-us-deep-south.pdf

## Slide 39
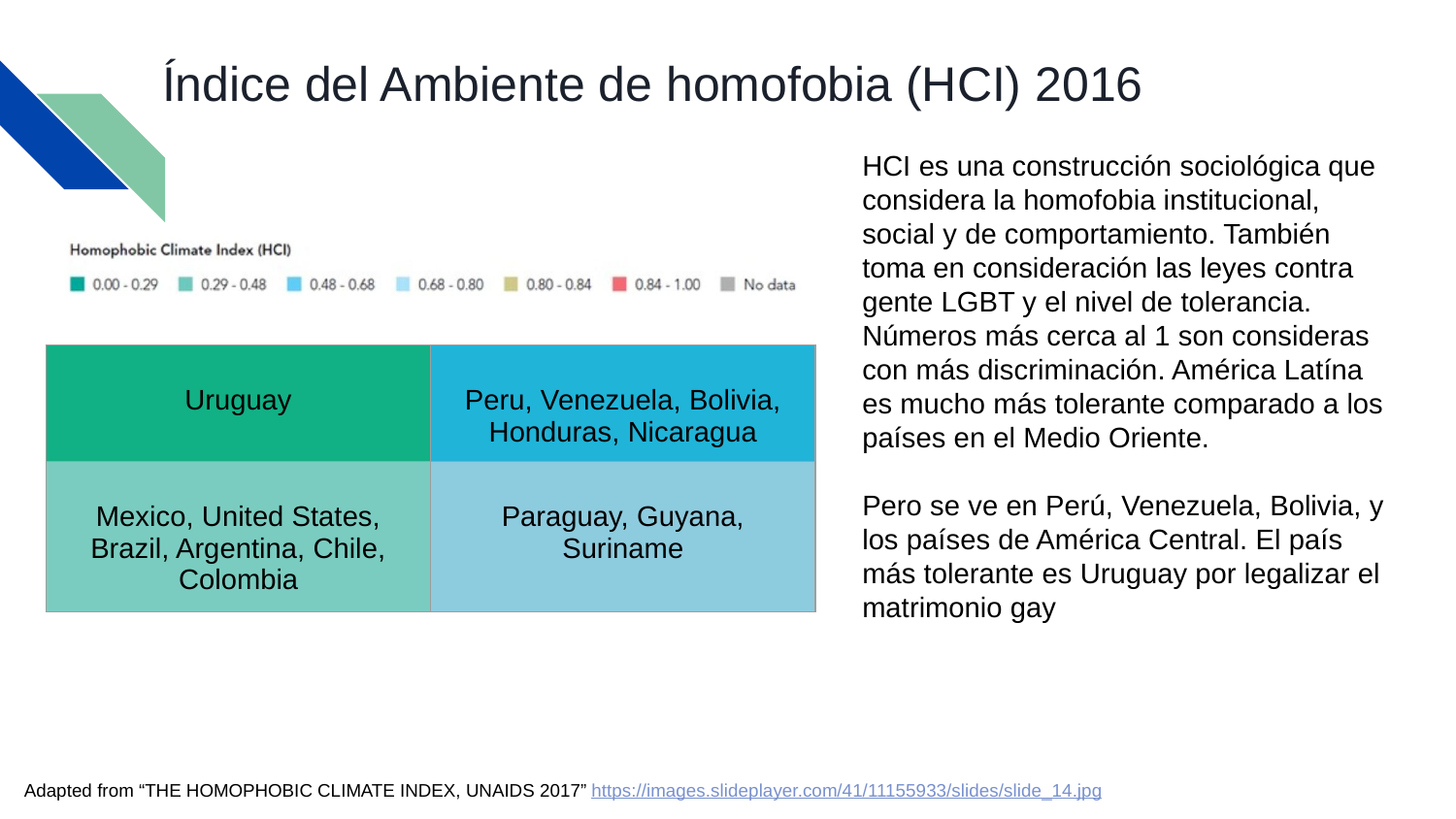

# Índice del Ambiente de homofobia (HCI) 2016
HCI es una construcción sociológica que considera la homofobia institucional, social y de comportamiento. También toma en consideración las leyes contra gente LGBT y el nivel de tolerancia. Números más cerca al 1 son consideras con más discriminación. América Latína es mucho más tolerante comparado a los países en el Medio Oriente.
Pero se ve en Perú, Venezuela, Bolivia, y los países de América Central. El país más tolerante es Uruguay por legalizar el matrimonio gay
| Uruguay | Peru, Venezuela, Bolivia, Honduras, Nicaragua |
| --- | --- |
| Mexico, United States, Brazil, Argentina, Chile, Colombia | Paraguay, Guyana, Suriname |
39
Adapted from “THE HOMOPHOBIC CLIMATE INDEX, UNAIDS 2017” https://images.slideplayer.com/41/11155933/slides/slide_14.jpg

## Slide 40
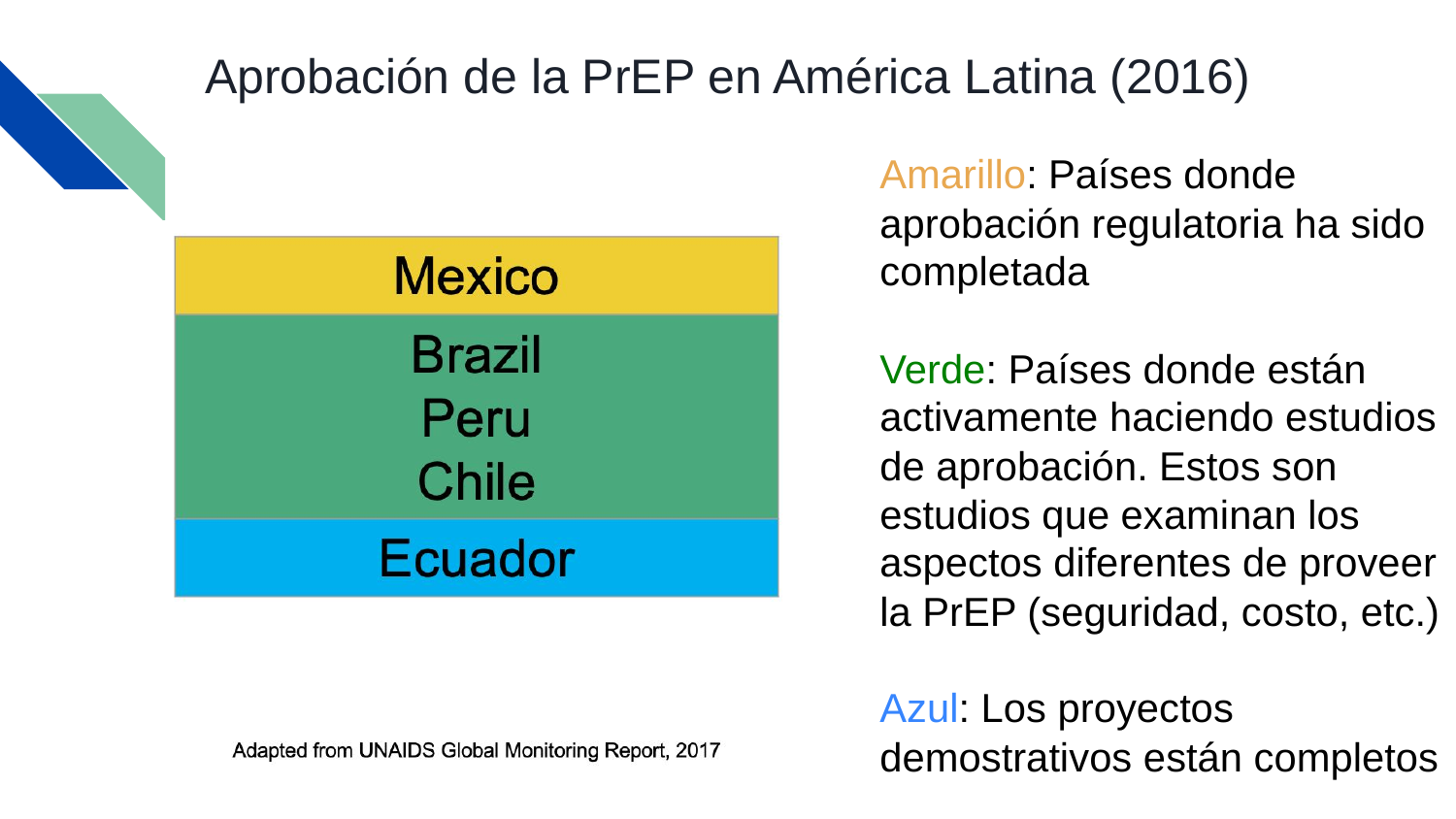

# Aprobación de la PrEP en América Latina (2016)
Amarillo: Países donde aprobación regulatoria ha sido completada
Verde: Países donde están activamente haciendo estudios de aprobación. Estos son estudios que examinan los aspectos diferentes de proveer la PrEP (seguridad, costo, etc.)
Azul: Los proyectos demostrativos están completos
40

## Slide 41
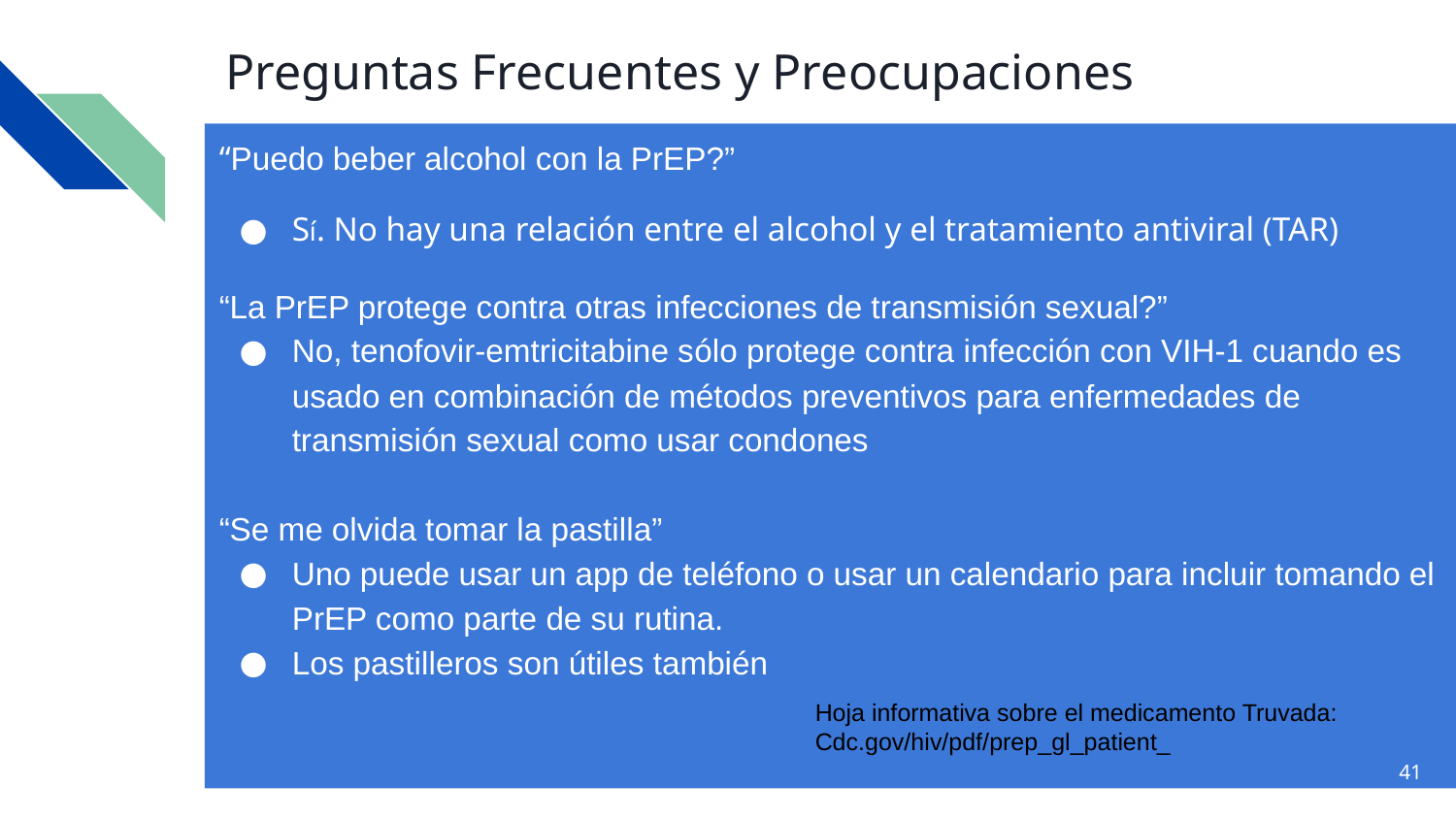

# Preguntas Frecuentes y Preocupaciones
“Puedo beber alcohol con la PrEP?”
Sí. No hay una relación entre el alcohol y el tratamiento antiviral (TAR)
“La PrEP protege contra otras infecciones de transmisión sexual?”
No, tenofovir-emtricitabine sólo protege contra infección con VIH-1 cuando es usado en combinación de métodos preventivos para enfermedades de transmisión sexual como usar condones
“Se me olvida tomar la pastilla”
Uno puede usar un app de teléfono o usar un calendario para incluir tomando el PrEP como parte de su rutina.
Los pastilleros son útiles también
Hoja informativa sobre el medicamento Truvada: Cdc.gov/hiv/pdf/prep_gl_patient_
41

## Slide 42
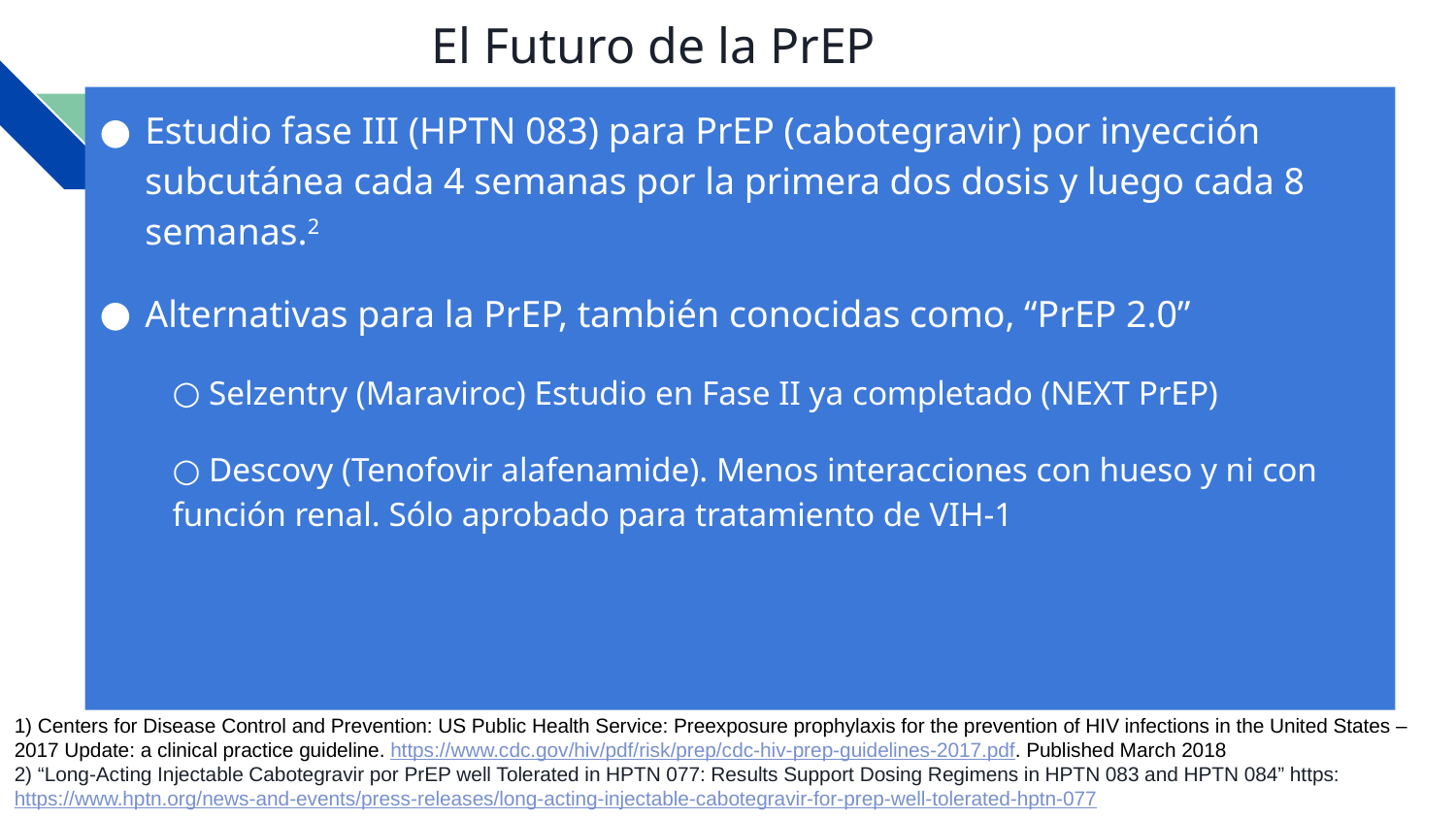

# El Futuro de la PrEP
Estudio fase III (HPTN 083) para PrEP (cabotegravir) por inyección subcutánea cada 4 semanas por la primera dos dosis y luego cada 8 semanas.2
Alternativas para la PrEP, también conocidas como, “PrEP 2.0”
 Selzentry (Maraviroc) Estudio en Fase II ya completado (NEXT PrEP)
 Descovy (Tenofovir alafenamide). Menos interacciones con hueso y ni con función renal. Sólo aprobado para tratamiento de VIH-1
1) Centers for Disease Control and Prevention: US Public Health Service: Preexposure prophylaxis for the prevention of HIV infections in the United States – 2017 Update: a clinical practice guideline. https://www.cdc.gov/hiv/pdf/risk/prep/cdc-hiv-prep-guidelines-2017.pdf. Published March 2018
2) “Long-Acting Injectable Cabotegravir por PrEP well Tolerated in HPTN 077: Results Support Dosing Regimens in HPTN 083 and HPTN 084” https:https://www.hptn.org/news-and-events/press-releases/long-acting-injectable-cabotegravir-for-prep-well-tolerated-hptn-077
42

## Slide 43
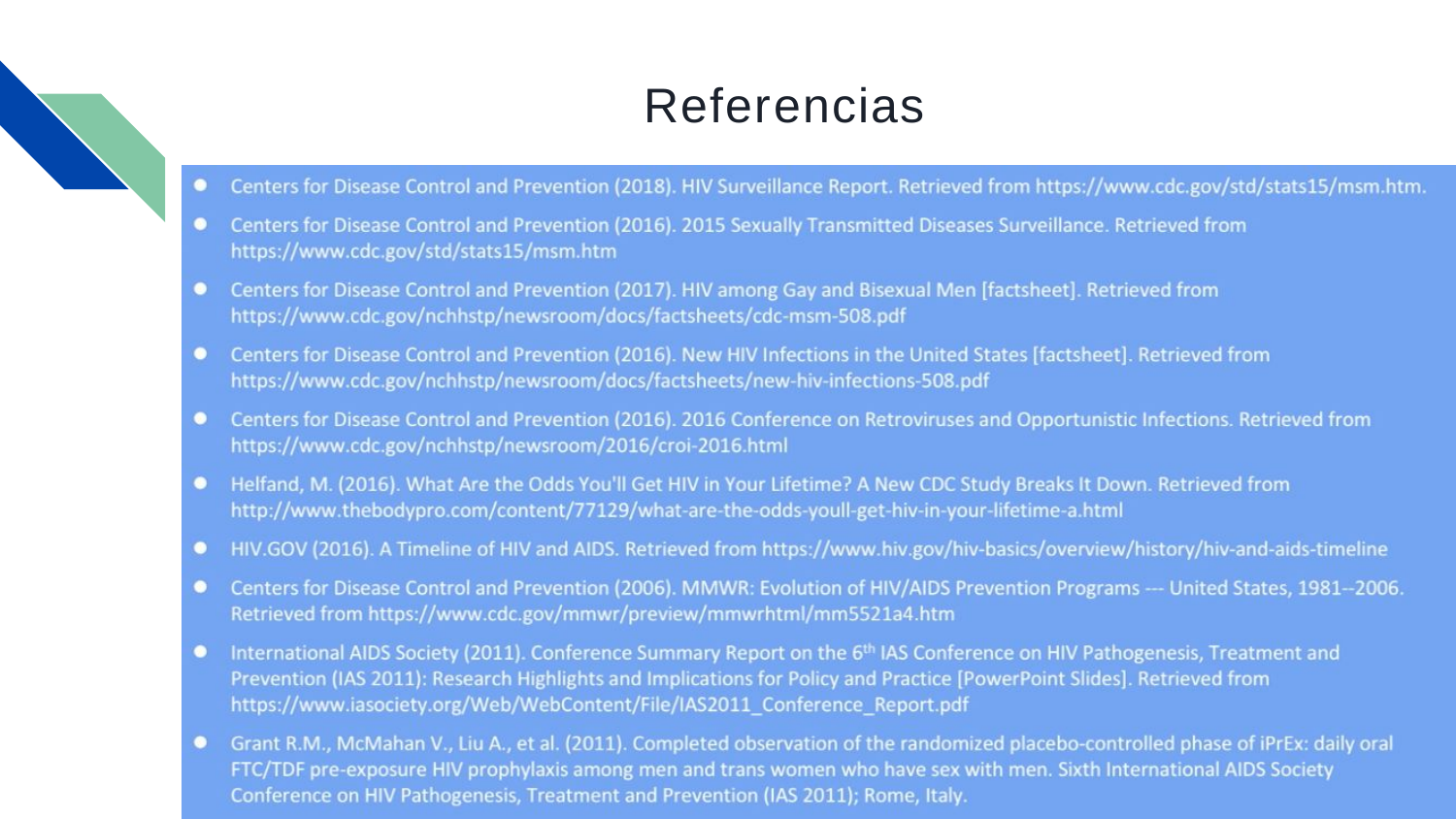

# Referencias
43

## Slide 44
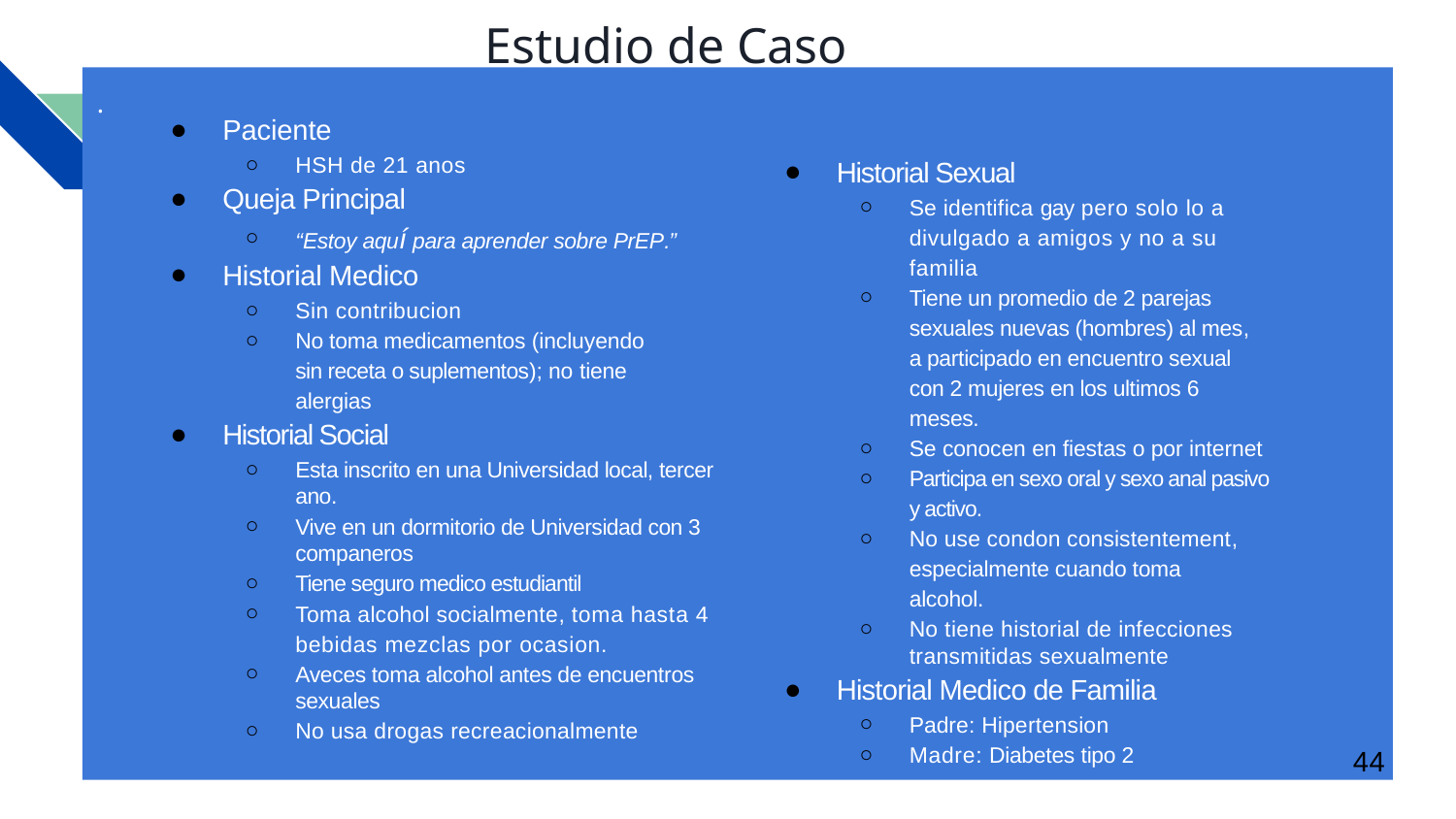

# Estudio de Caso
.
Paciente
HSH de 21 anos
Queja Principal
“Estoy aquí para aprender sobre PrEP.”
Historial Medico
Sin contribucion
No toma medicamentos (incluyendo sin receta o suplementos); no tiene alergias
Historial Social
Esta inscrito en una Universidad local, tercer ano.
Vive en un dormitorio de Universidad con 3 companeros
Tiene seguro medico estudiantil
Toma alcohol socialmente, toma hasta 4 bebidas mezclas por ocasion.
Aveces toma alcohol antes de encuentros sexuales
No usa drogas recreacionalmente
Historial Sexual
Se identifica gay pero solo lo a divulgado a amigos y no a su familia
Tiene un promedio de 2 parejas sexuales nuevas (hombres) al mes, a participado en encuentro sexual con 2 mujeres en los ultimos 6 meses.
Se conocen en fiestas o por internet
Participa en sexo oral y sexo anal pasivo y activo.
No use condon consistentement, especialmente cuando toma alcohol.
No tiene historial de infecciones transmitidas sexualmente
Historial Medico de Familia
Padre: Hipertension
Madre: Diabetes tipo 2
44
44
