# Supplementary material for: HIV Pre-exposure Prophylaxis Education for Clinicians Caring for Spanish-Speaking Men Who Have Sex With Men (MSM)
Source: MedEdPORTAL. 2021 Mar 18;17:11110. doi: 10.15766/mep_2374-8265.11110 (PMC8015640; doi:10.15766/mep_2374-8265.11110)
Supplement: Supplementary file 1 — Spanish PPT Presentation.pptxEnglish PPT Presentation.pptxSpanish Audio-Guided PPT Video Presentation.pptxEnglish Audio-Guided PPT Video Presentation.pptxDiscussion Guide.docxPatient-Physician Video.mp4Spanish Transcript of Patient-Physician Video.docxEnglish Transcript of Patient-Physician Video.docxPreworkshop Evaluation Form.docxPostworkshop Evaluation Form.docx [file mep_2374-8265.11110-s001.zip › D. English Audio-Guided PPT Video Presentation.pptx]

## Slide 1
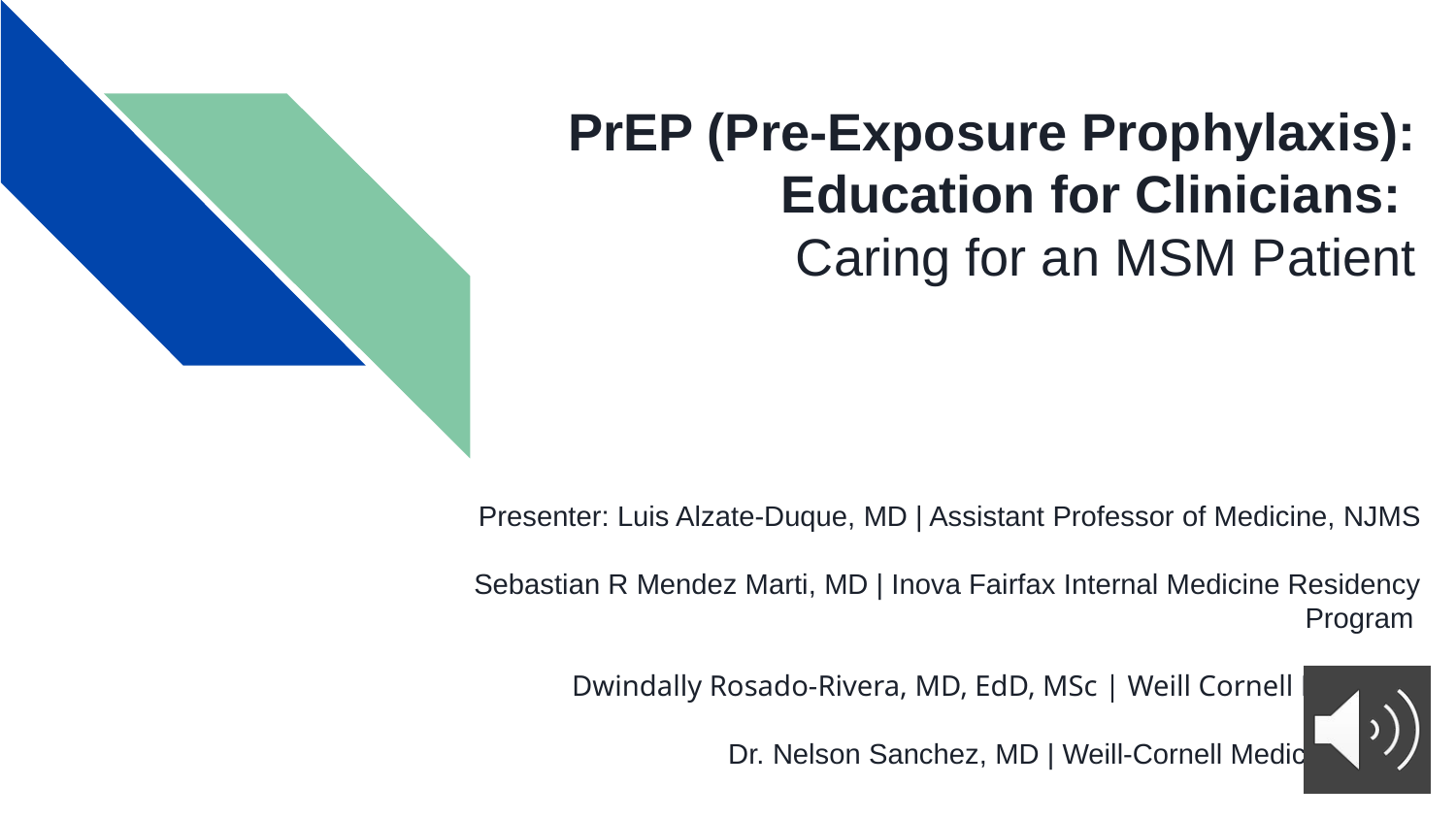

# PrEP (Pre-Exposure Prophylaxis): Education for Clinicians: Caring for an MSM Patient
Presenter: Luis Alzate-Duque, MD | Assistant Professor of Medicine, NJMS
Sebastian R Mendez Marti, MD | Inova Fairfax Internal Medicine Residency Program
Dwindally Rosado-Rivera, MD, EdD, MSc | Weill Cornell Medicine
Dr. Nelson Sanchez, MD | Weill-Cornell Medical Center
1

## Slide 2
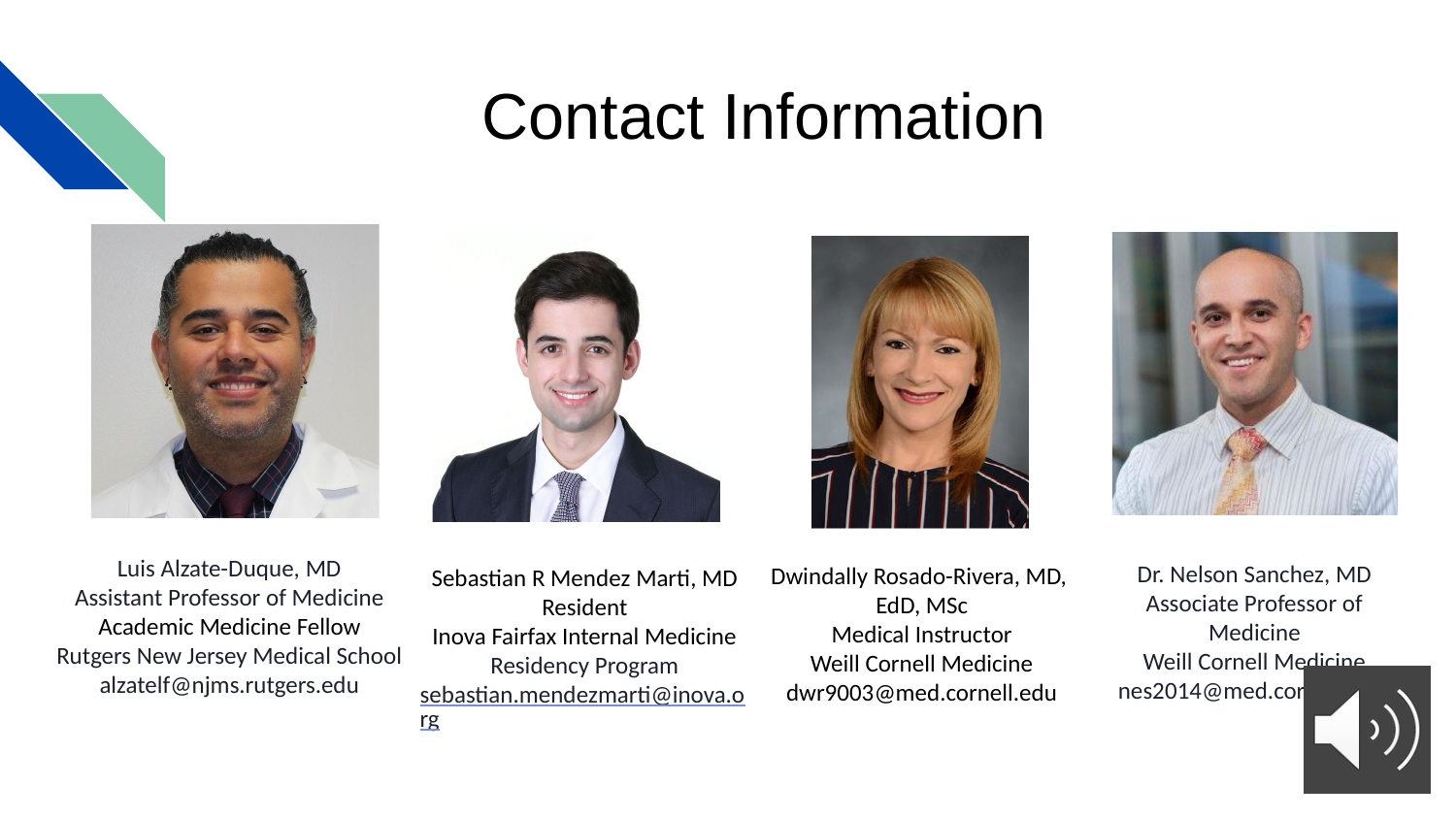

Contact Information
Dr. Nelson Sanchez, MD
Associate Professor of Medicine
Weill Cornell Medicine
nes2014@med.cornell.edu
Sebastian R Mendez Marti, MD
Resident
Inova Fairfax Internal Medicine Residency Program
sebastian.mendezmarti@inova.org
Luis Alzate-Duque, MD
Assistant Professor of Medicine
Academic Medicine Fellow
Rutgers New Jersey Medical School
alzatelf@njms.rutgers.edu
Dwindally Rosado-Rivera, MD,
EdD, MSc
Medical Instructor
Weill Cornell Medicine
dwr9003@med.cornell.edu
2

## Slide 3
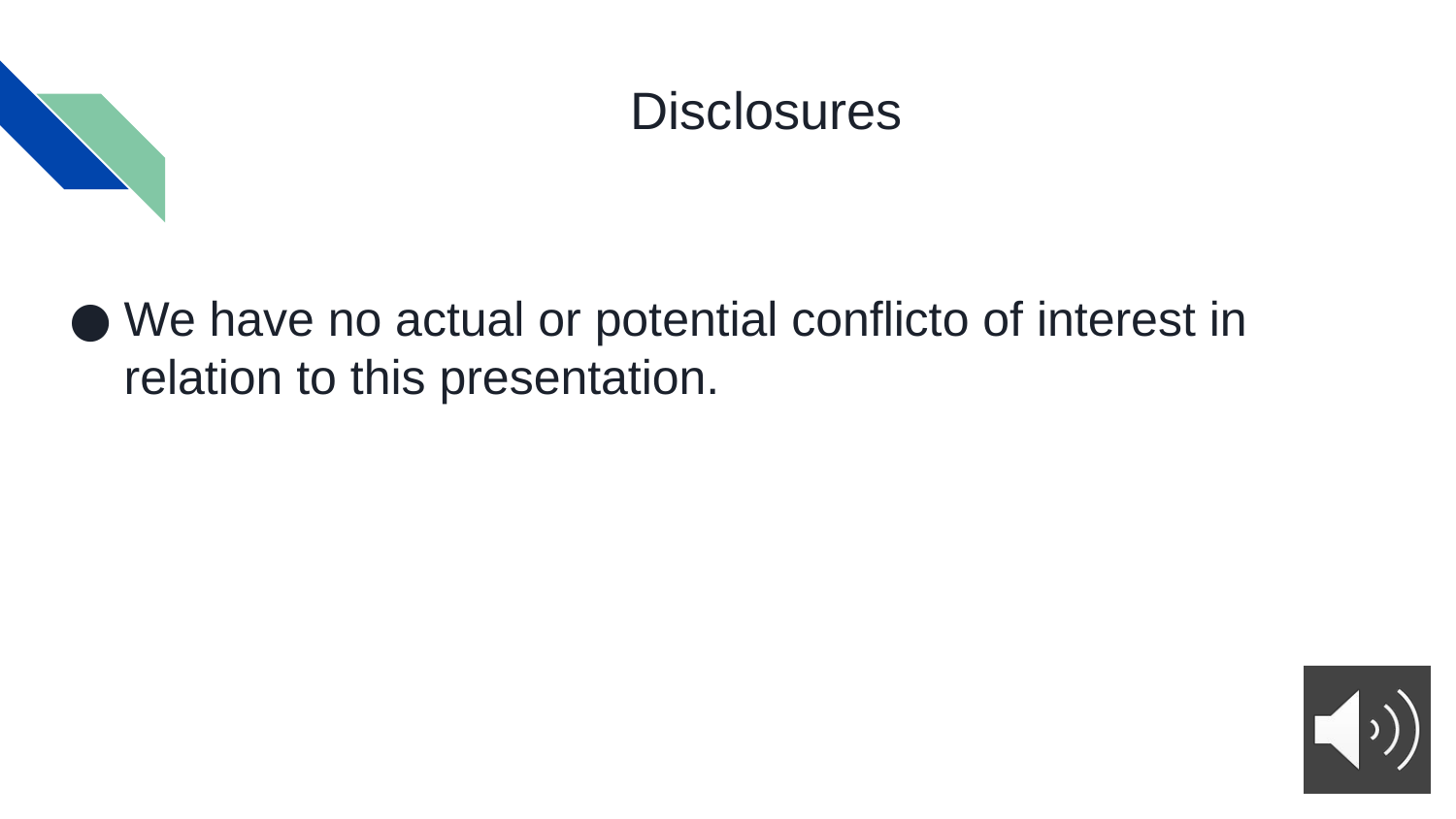

# Disclosures
We have no actual or potential conflicto of interest in relation to this presentation.
3

## Slide 4
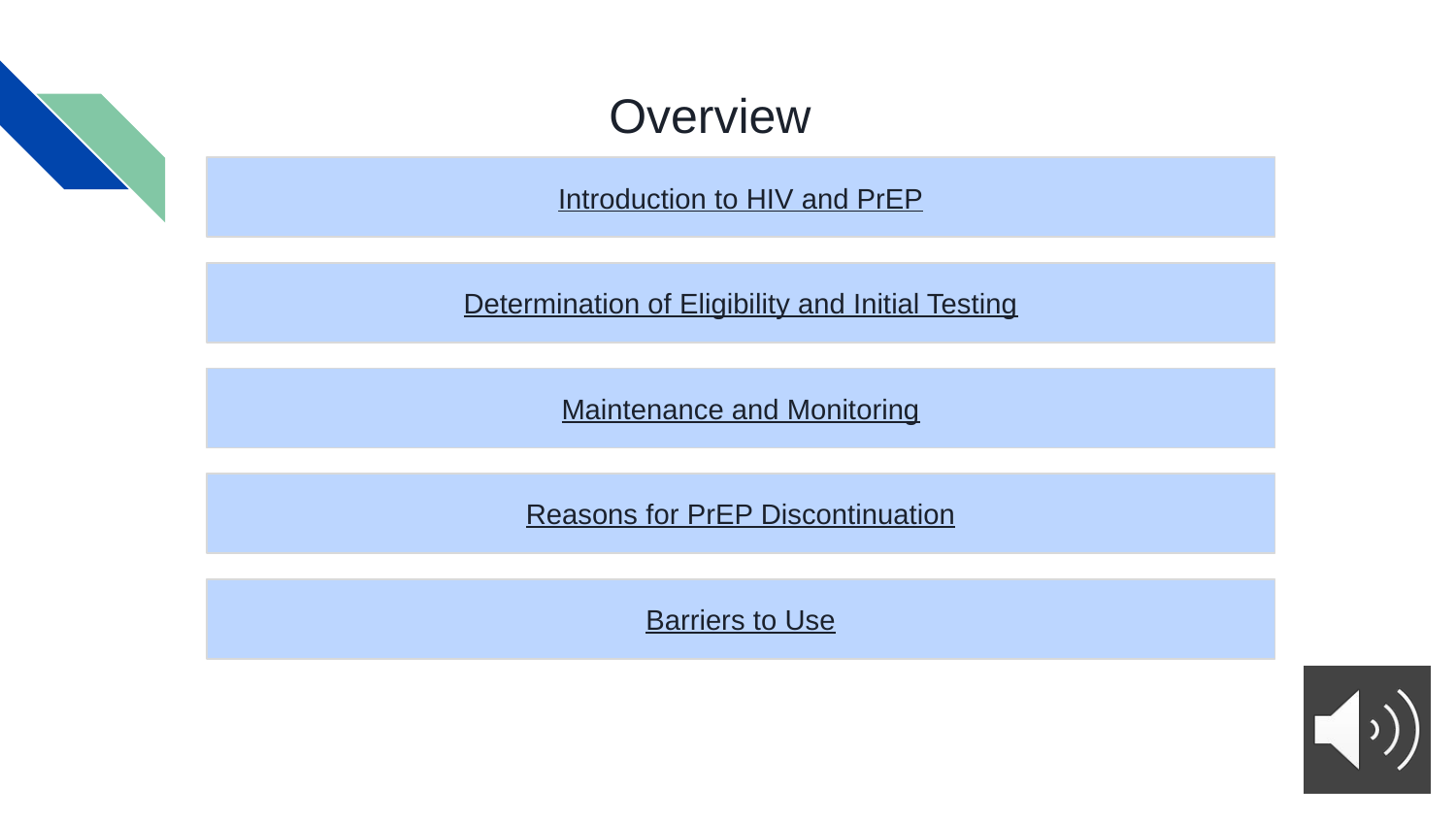

# Overview
Introduction to HIV and PrEP
Determination of Eligibility and Initial Testing
Maintenance and Monitoring
Reasons for PrEP Discontinuation
Barriers to Use
4

## Slide 5
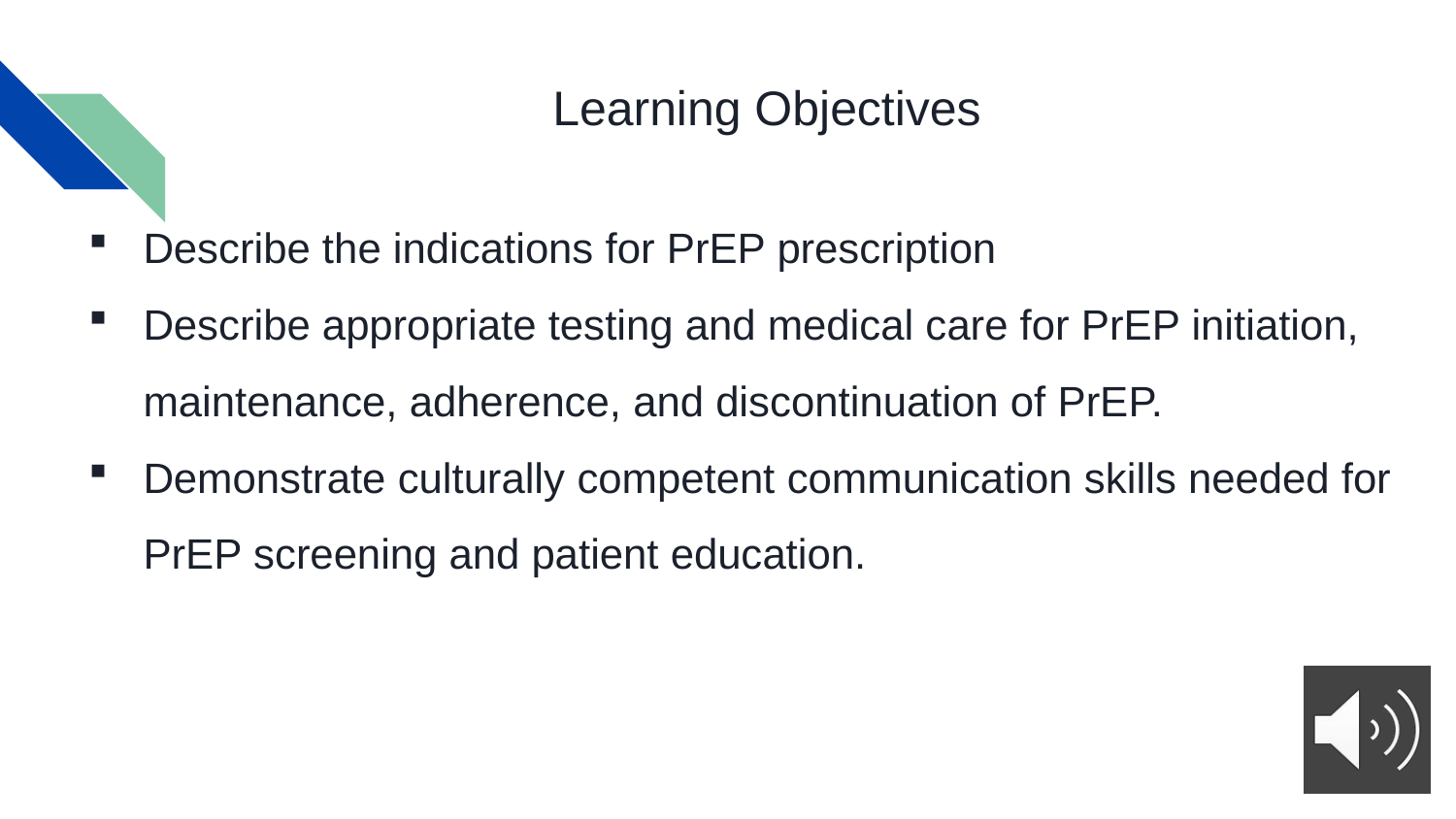

# Learning Objectives
Describe the indications for PrEP prescription
Describe appropriate testing and medical care for PrEP initiation, maintenance, adherence, and discontinuation of PrEP.
Demonstrate culturally competent communication skills needed for PrEP screening and patient education.
5

## Slide 6
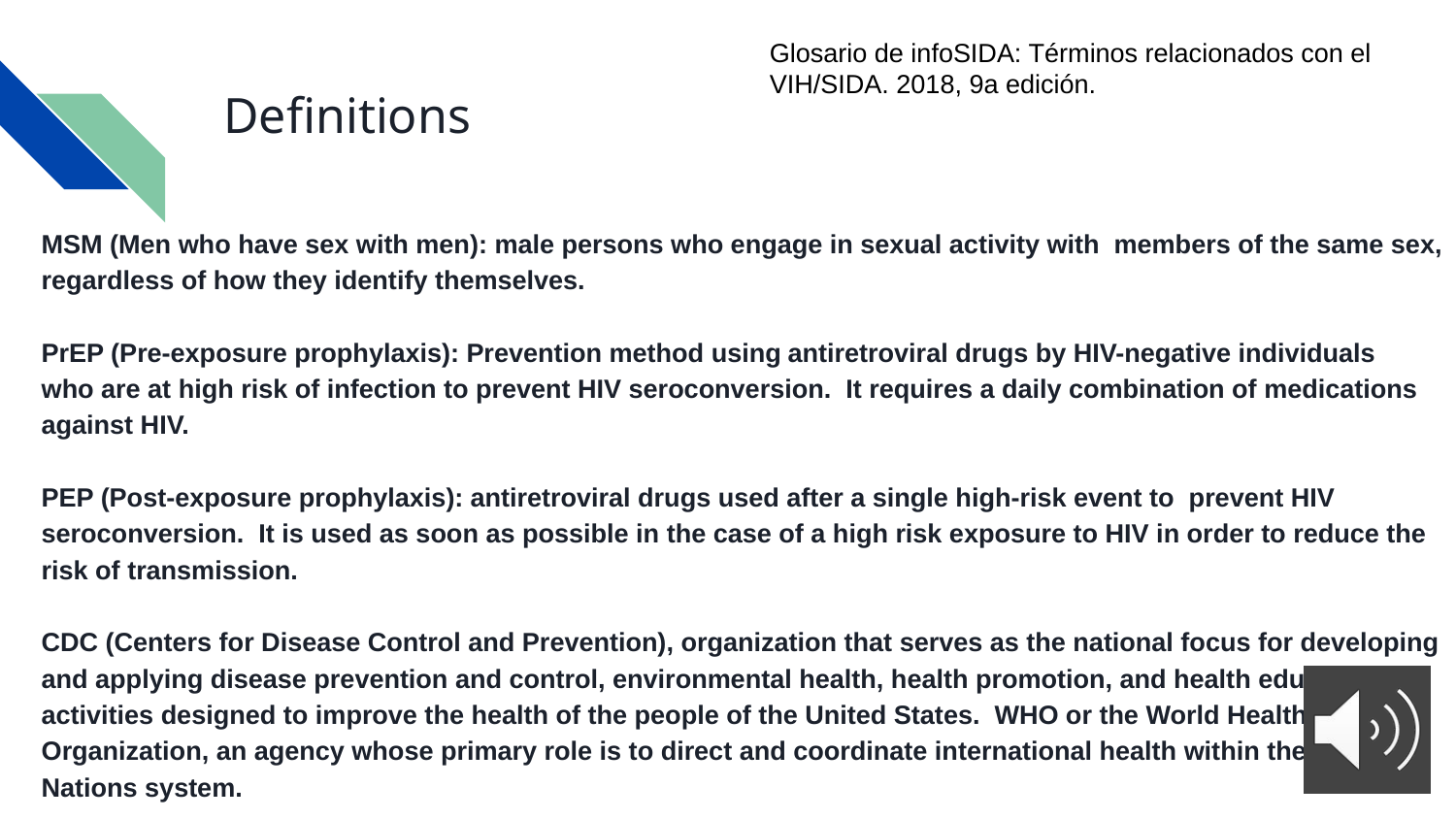

Glosario de infoSIDA: Términos relacionados con el VIH/SIDA. 2018, 9a edición.
# Definitions
MSM (Men who have sex with men): male persons who engage in sexual activity with members of the same sex, regardless of how they identify themselves.
PrEP (Pre-exposure prophylaxis): Prevention method using antiretroviral drugs by HIV-negative individuals who are at high risk of infection to prevent HIV seroconversion. It requires a daily combination of medications against HIV.
PEP (Post-exposure prophylaxis): antiretroviral drugs used after a single high-risk event to prevent HIV seroconversion. It is used as soon as possible in the case of a high risk exposure to HIV in order to reduce the risk of transmission.
CDC (Centers for Disease Control and Prevention), organization that serves as the national focus for developing and applying disease prevention and control, environmental health, health promotion, and health education activities designed to improve the health of the people of the United States. WHO or the World Health Organization, an agency whose primary role is to direct and coordinate international health within the United Nations system.
6

## Slide 7
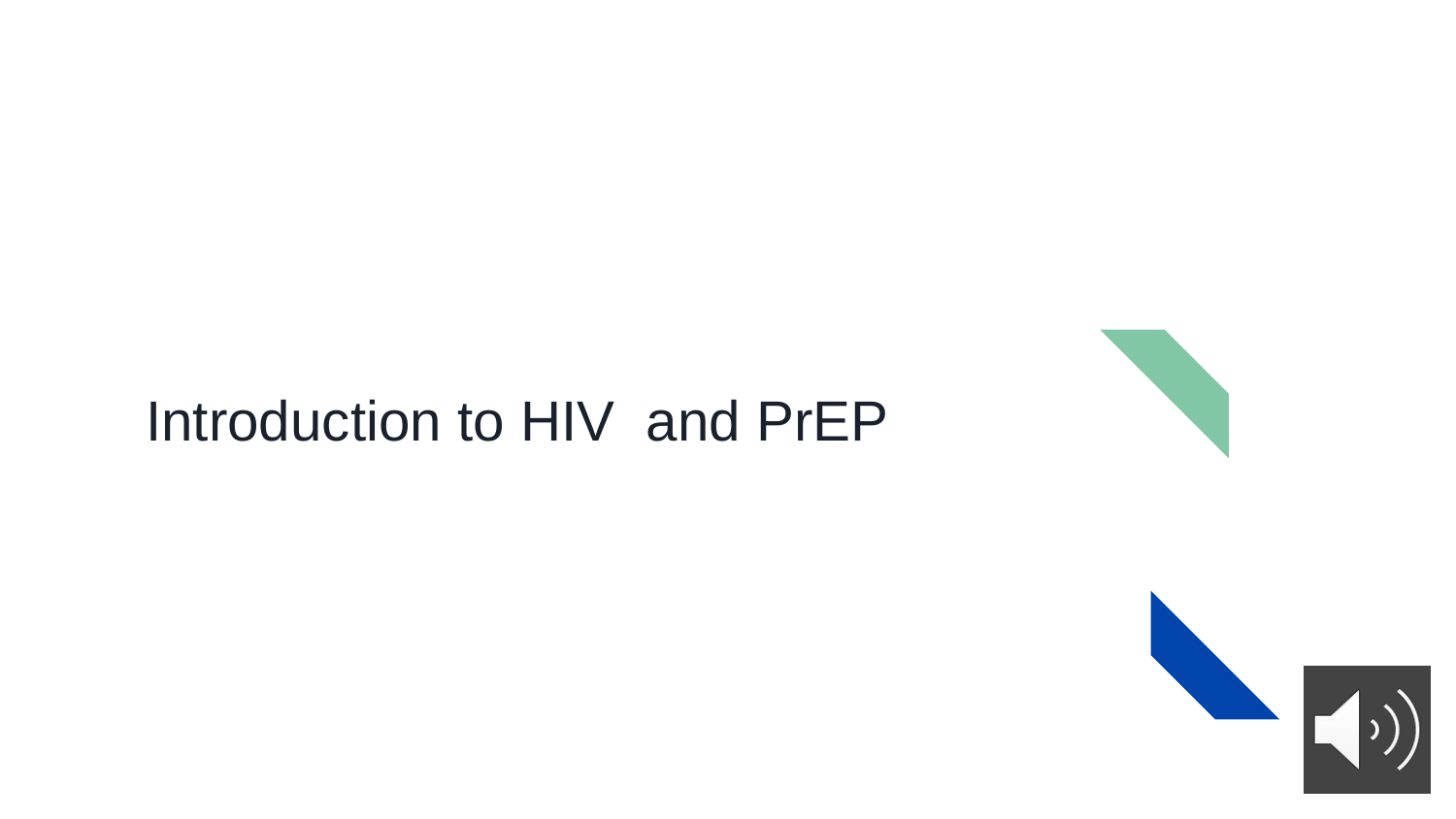

# Introduction to HIV and PrEP
7

## Slide 8
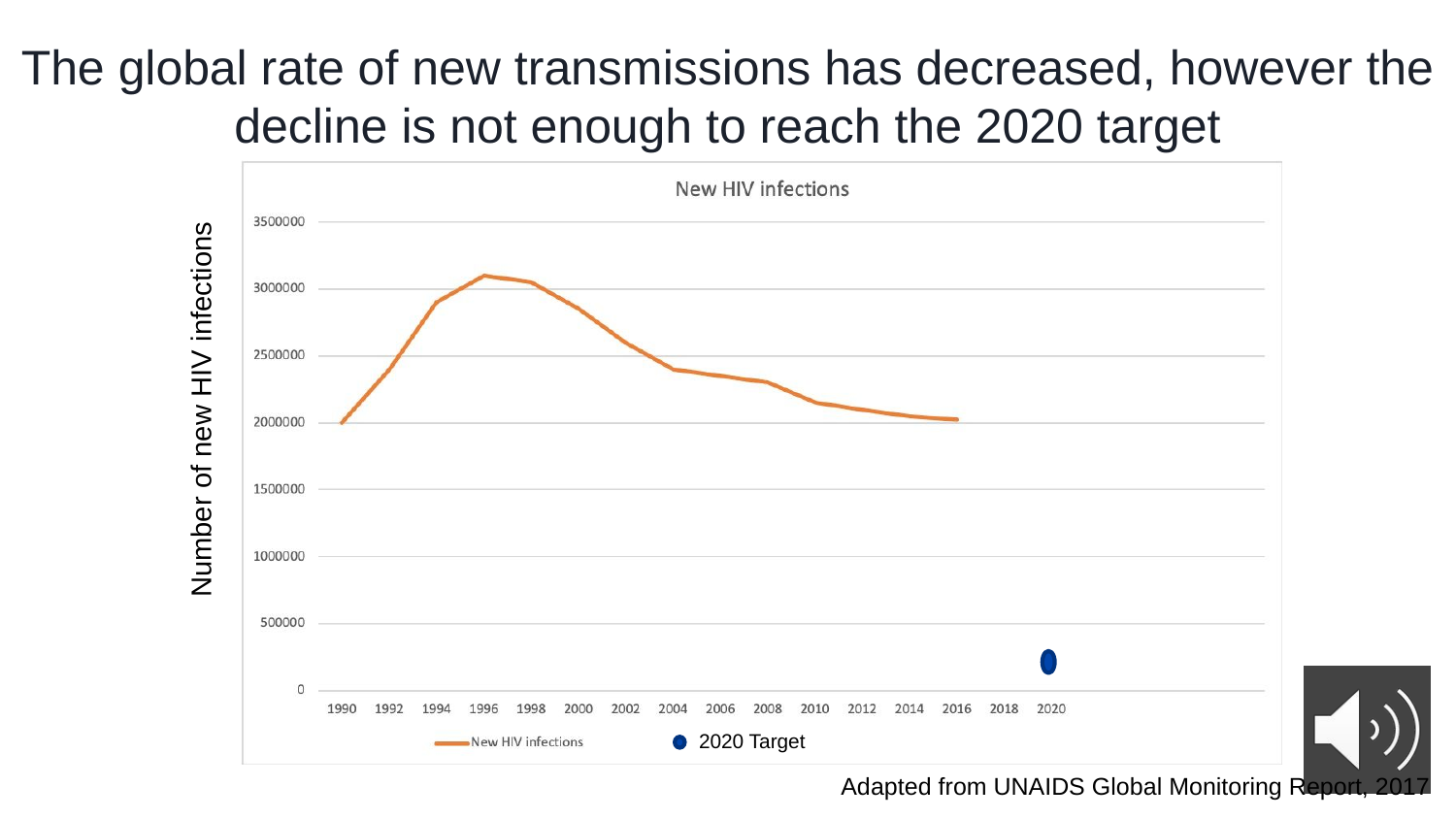

# The global rate of new transmissions has decreased, however the decline is not enough to reach the 2020 target
Number of new HIV infections
2020 Target
8
Adapted from UNAIDS Global Monitoring Report, 2017

## Slide 9
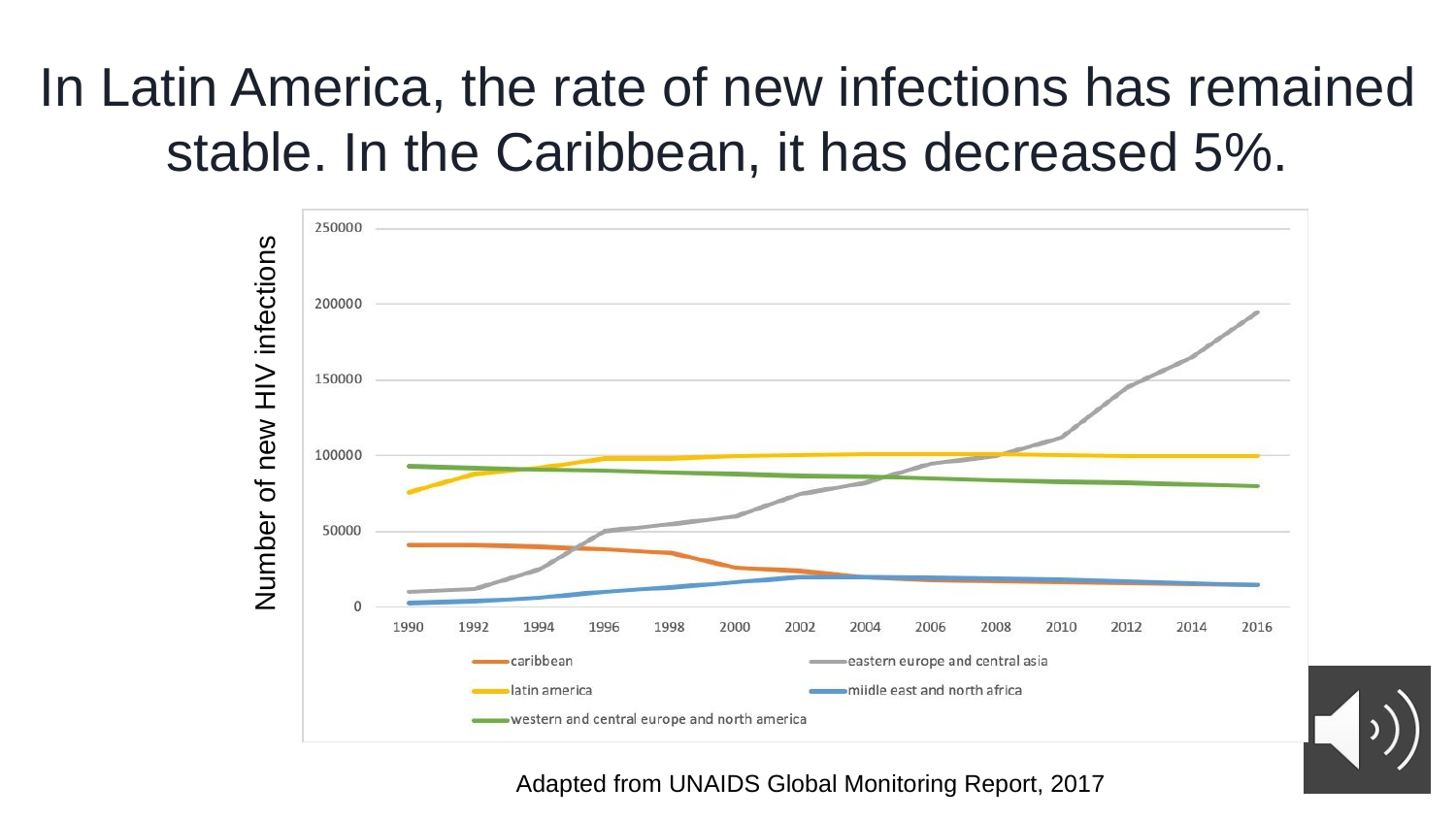

# In Latin America, the rate of new infections has remained stable. In the Caribbean, it has decreased 5%.
Number of new HIV infections
9
Adapted from UNAIDS Global Monitoring Report, 2017

## Slide 10
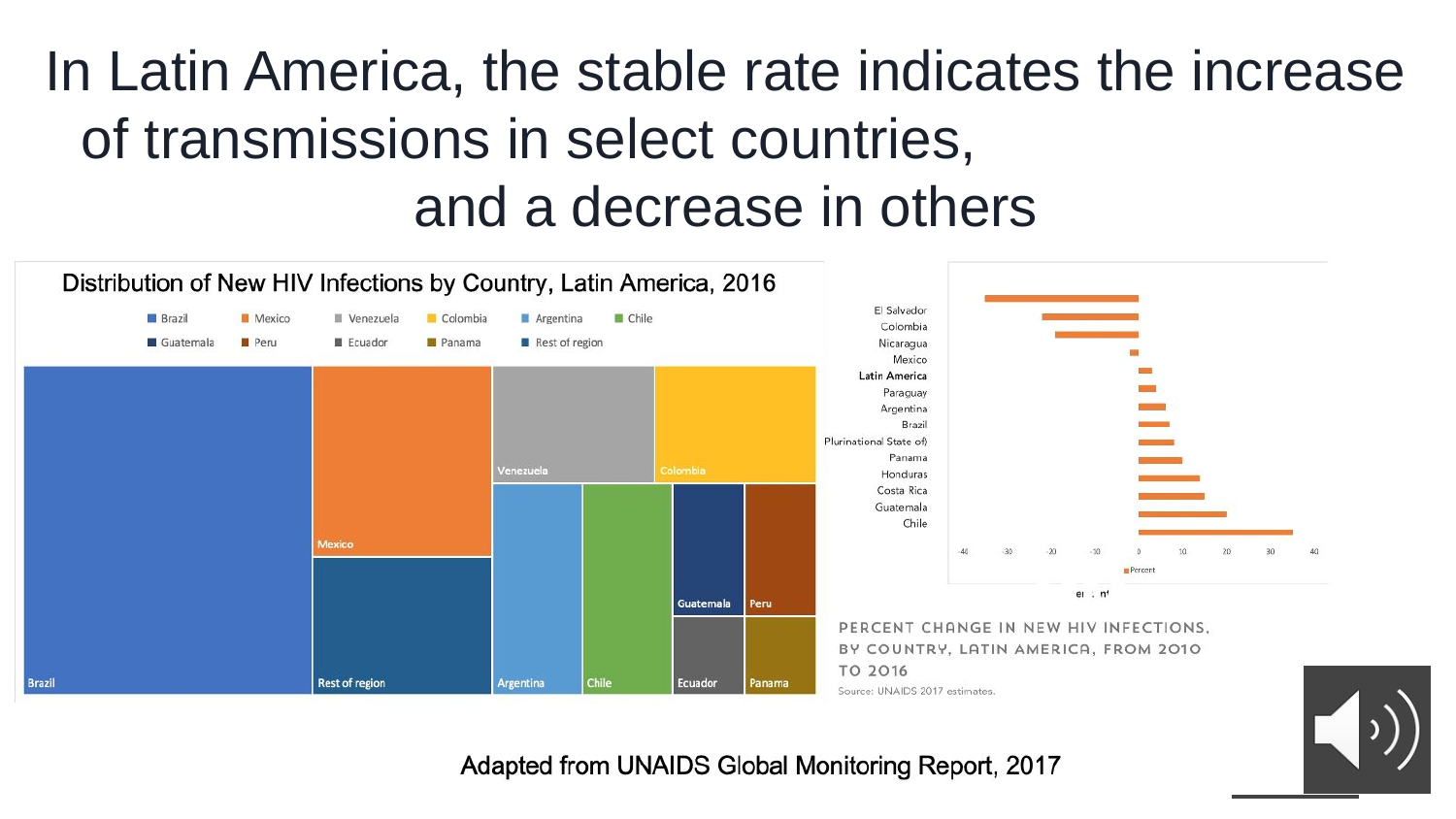

# In Latin America, the stable rate indicates the increase of transmissions in select countries, and a decrease in others
10

## Slide 11
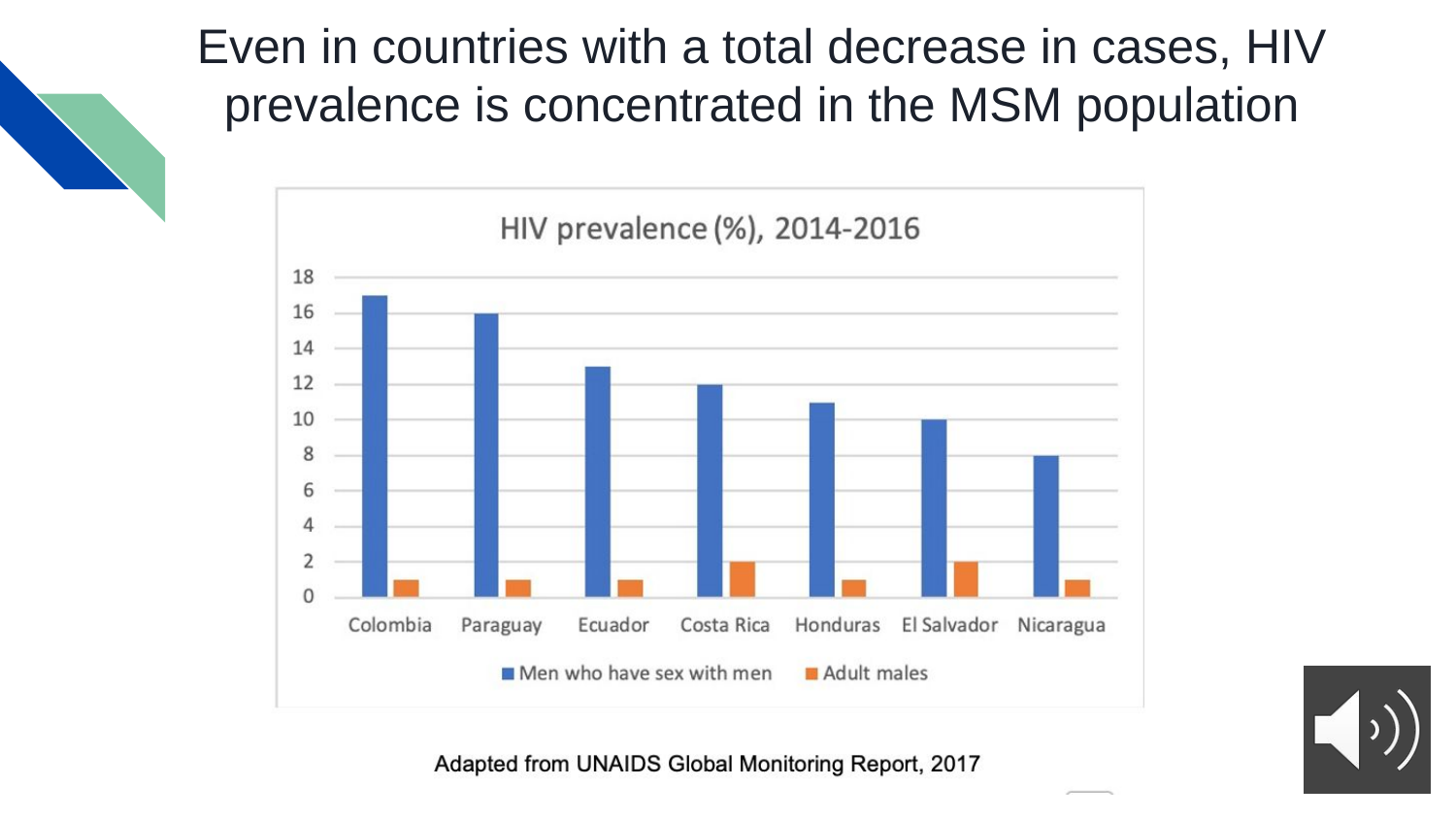

# Even in countries with a total decrease in cases, HIV prevalence is concentrated in the MSM population
7/10
11

## Slide 12
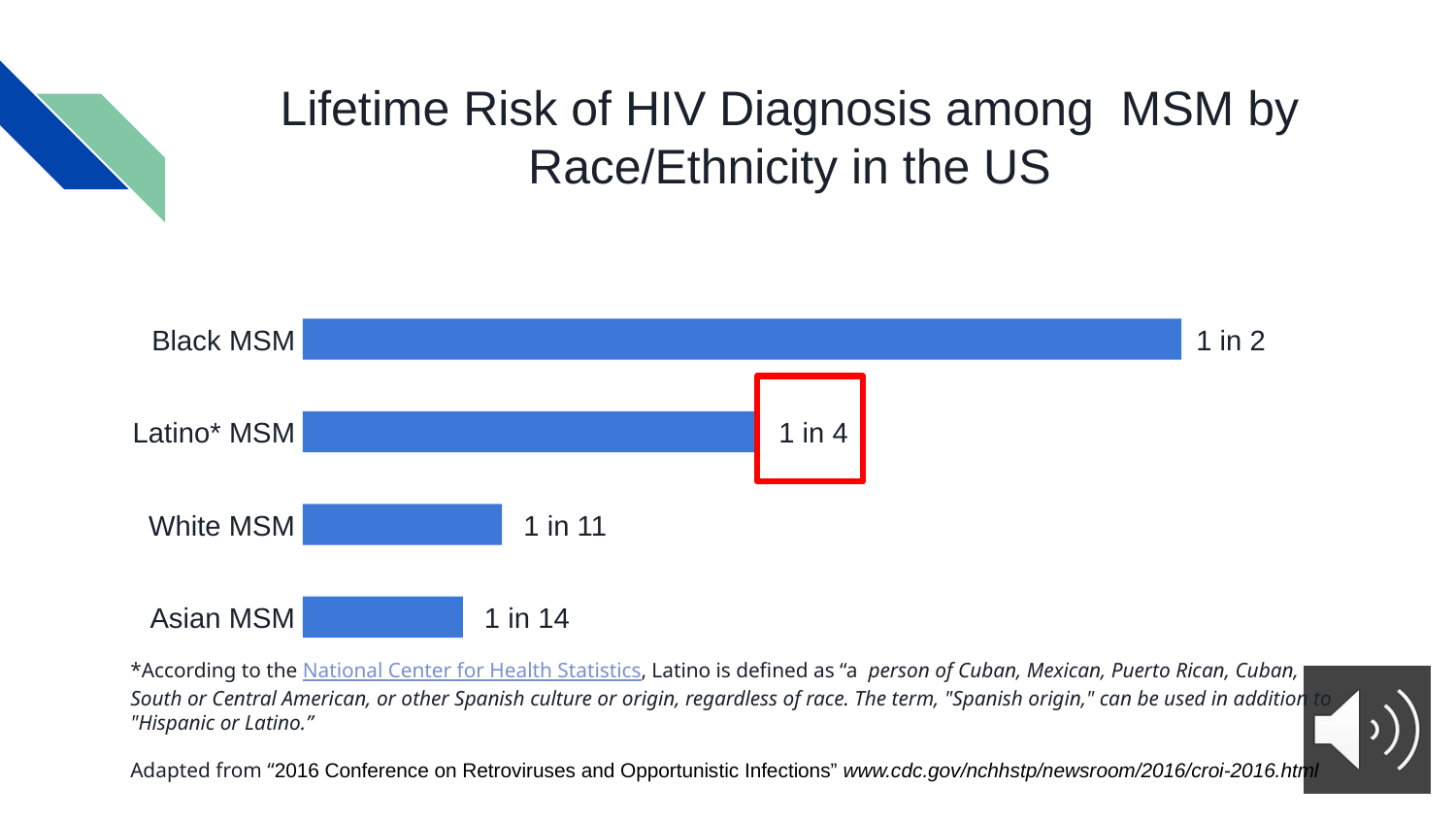

# Lifetime Risk of HIV Diagnosis among MSM by Race/Ethnicity in the US
Black MSM
1 in 2
Latino* MSM
1 in 4
White MSM
1 in 11
Asian MSM
1 in 14
*According to the National Center for Health Statistics, Latino is defined as “a person of Cuban, Mexican, Puerto Rican, Cuban, South or Central American, or other Spanish culture or origin, regardless of race. The term, "Spanish origin," can be used in addition to "Hispanic or Latino.”
Adapted from “2016 Conference on Retroviruses and Opportunistic Infections” www.cdc.gov/nchhstp/newsroom/2016/croi-2016.html
12

## Slide 13
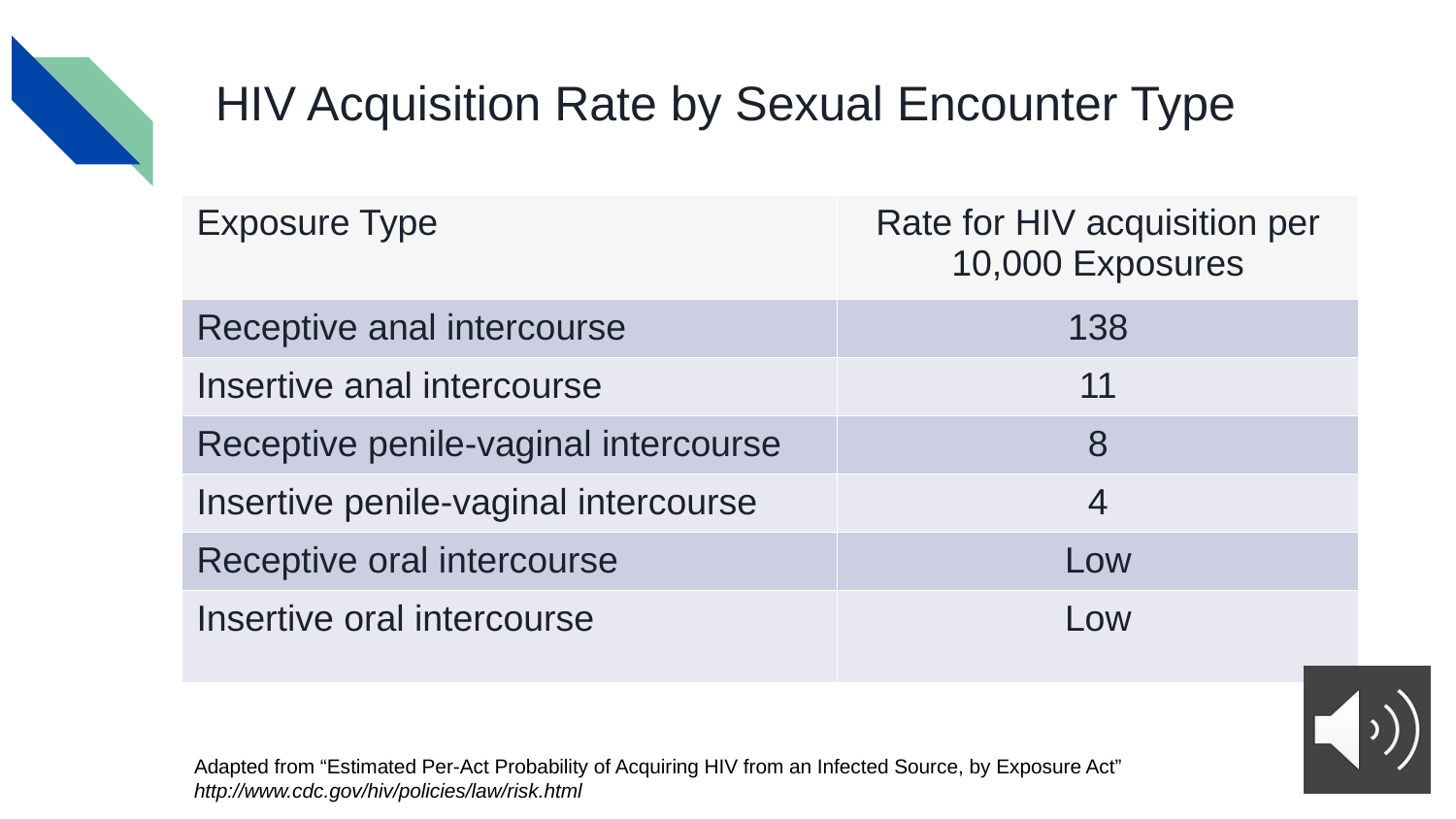

# HIV Acquisition Rate by Sexual Encounter Type
| Exposure Type | Rate for HIV acquisition per 10,000 Exposures |
| --- | --- |
| Receptive anal intercourse | 138 |
| Insertive anal intercourse | 11 |
| Receptive penile-vaginal intercourse | 8 |
| Insertive penile-vaginal intercourse | 4 |
| Receptive oral intercourse | Low |
| Insertive oral intercourse | Low |
Adapted from “Estimated Per-Act Probability of Acquiring HIV from an Infected Source, by Exposure Act” http://www.cdc.gov/hiv/policies/law/risk.html

## Slide 14
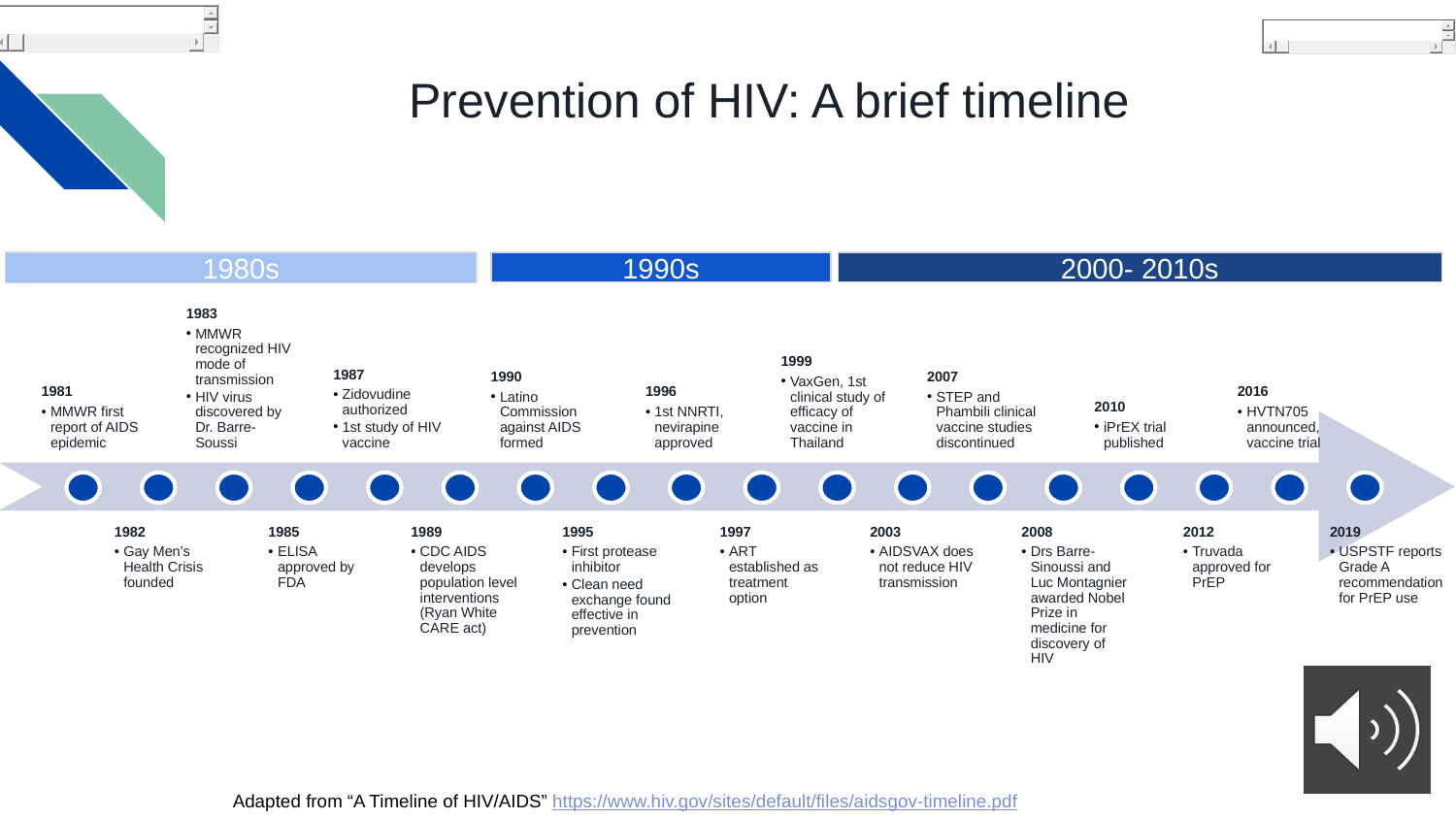

# Prevention of HIV: A brief timeline
1981
MMWR first report of AIDS epidemic
1983
MMWR recognized HIV mode of transmission
HIV virus discovered by Dr. Barre-Soussi
1987
Zidovudine authorized
1st study of HIV vaccine
1990
Latino Commission against AIDS formed
1996
1st NNRTI, nevirapine approved
1999
VaxGen, 1st clinical study of efficacy of vaccine in Thailand
2007
STEP and Phambili clinical vaccine studies discontinued
2010
iPrEX trial published
2016
HVTN705 announced, vaccine trial
1982
Gay Men's Health Crisis founded
1985
ELISA approved by FDA
1989
CDC AIDS develops population level interventions (Ryan White CARE act)
1995
First protease inhibitor
Clean need exchange found effective in prevention
1997
ART established as treatment option
2003
AIDSVAX does not reduce HIV transmission
2008
Drs Barre-Sinoussi and Luc Montagnier awarded Nobel Prize in medicine for discovery of HIV
2012
Truvada approved for PrEP
2019
USPSTF reports Grade A recommendation for PrEP use
1980s
1990s
2000- 2010s
14
Adapted from “A Timeline of HIV/AIDS” https://www.hiv.gov/sites/default/files/aidsgov-timeline.pdf

## Slide 15
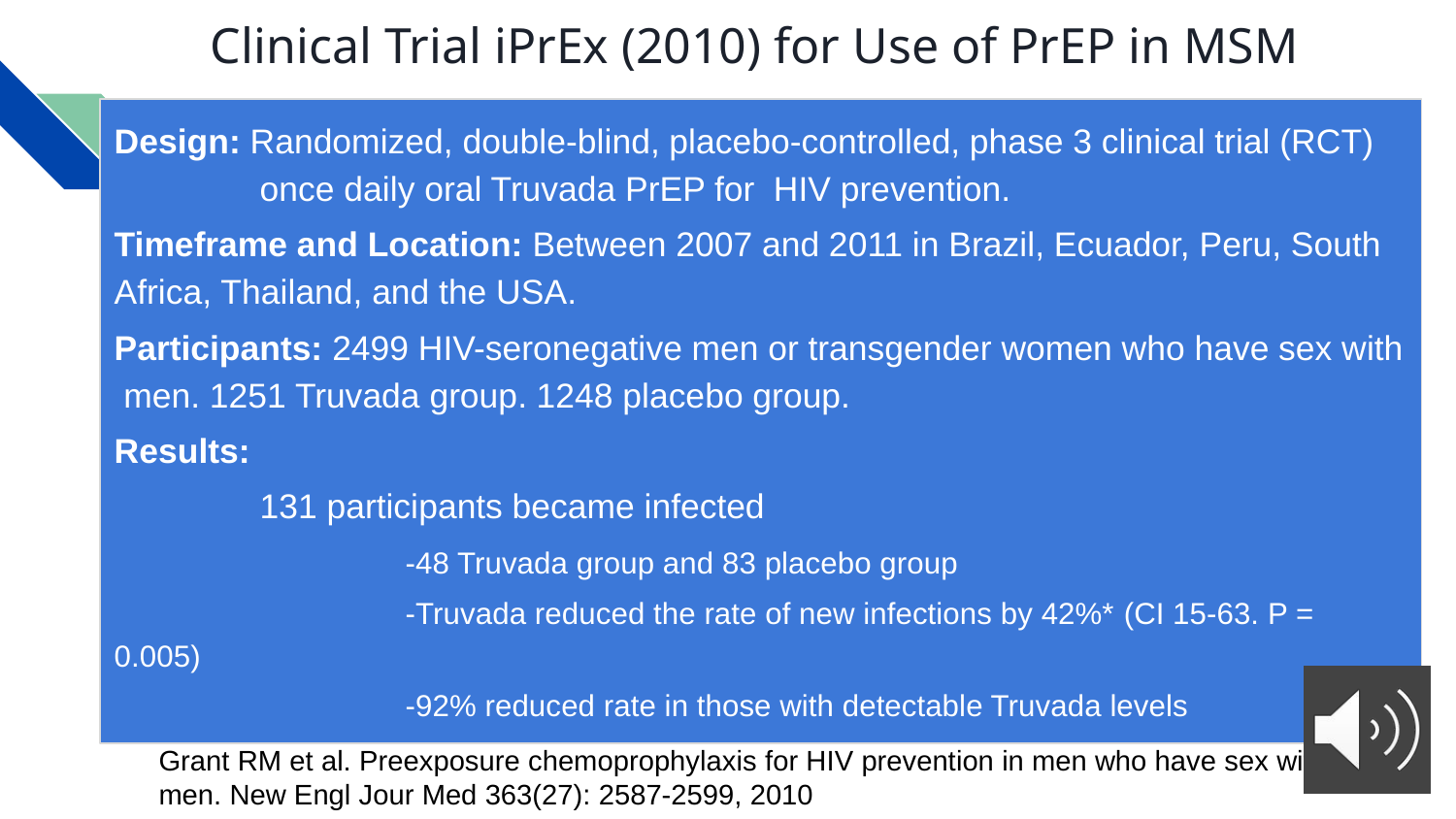

# Clinical Trial iPrEx (2010) for Use of PrEP in MSM
Design: Randomized, double-blind, placebo-controlled, phase 3 clinical trial (RCT)
	once daily oral Truvada PrEP for HIV prevention.
Timeframe and Location: Between 2007 and 2011 in Brazil, Ecuador, Peru, South Africa, Thailand, and the USA.
Participants: 2499 HIV-seronegative men or transgender women who have sex with men. 1251 Truvada group. 1248 placebo group.
Results:
	131 participants became infected
		-48 Truvada group and 83 placebo group
		-Truvada reduced the rate of new infections by 42%* (CI 15-63. P = 0.005)
		-92% reduced rate in those with detectable Truvada levels
Grant RM et al. Preexposure chemoprophylaxis for HIV prevention in men who have sex with men. New Engl Jour Med 363(27): 2587-2599, 2010
15

## Slide 16
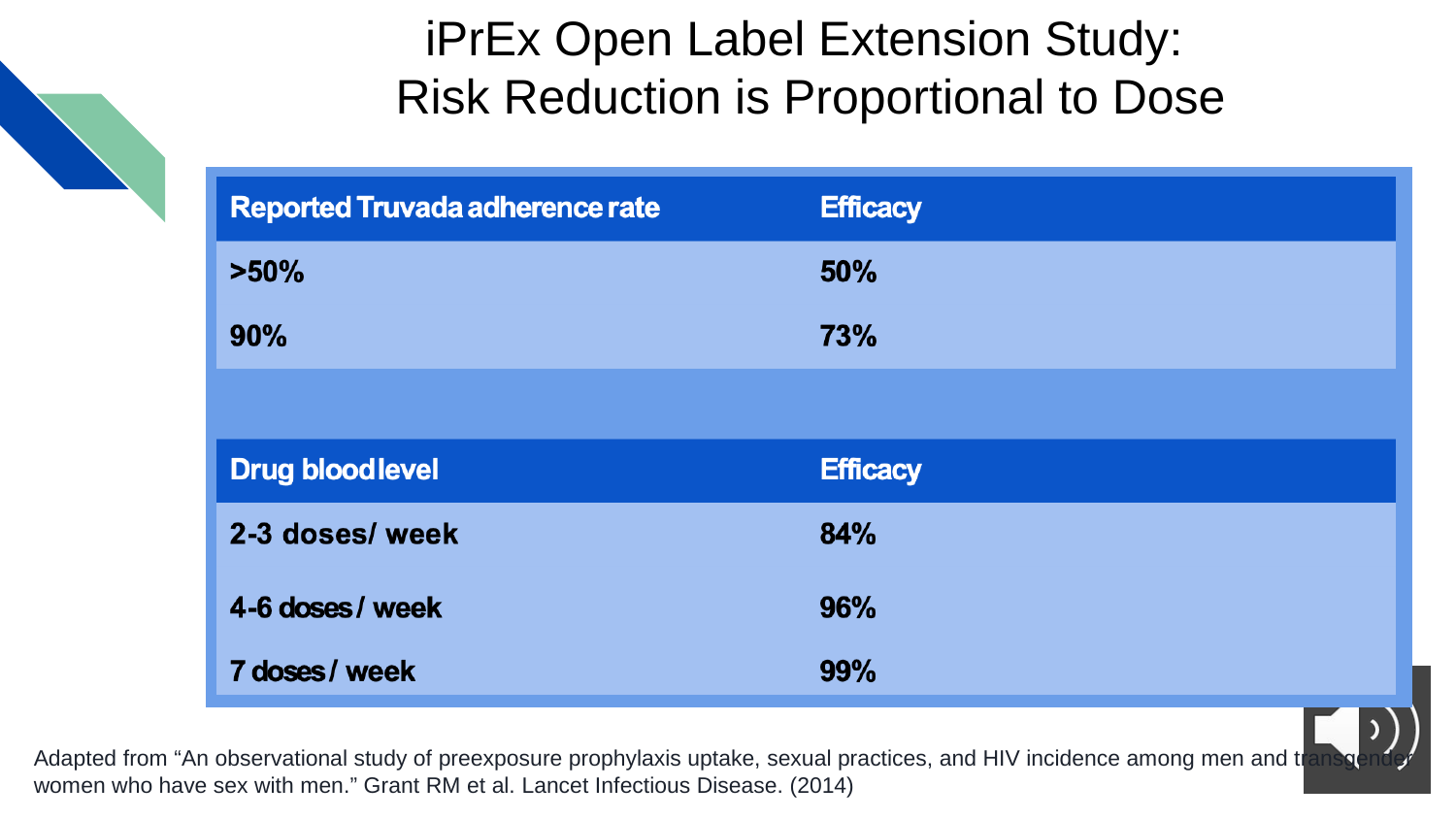

iPrEx Open Label Extension Study:
Risk Reduction is Proportional to Dose
# iPrEx Open Label Extension Study
Placebo
Adapted from “An observational study of preexposure prophylaxis uptake, sexual practices, and HIV incidence among men and transgender women who have sex with men.” Grant RM et al. Lancet Infectious Disease. (2014)
16

## Slide 17
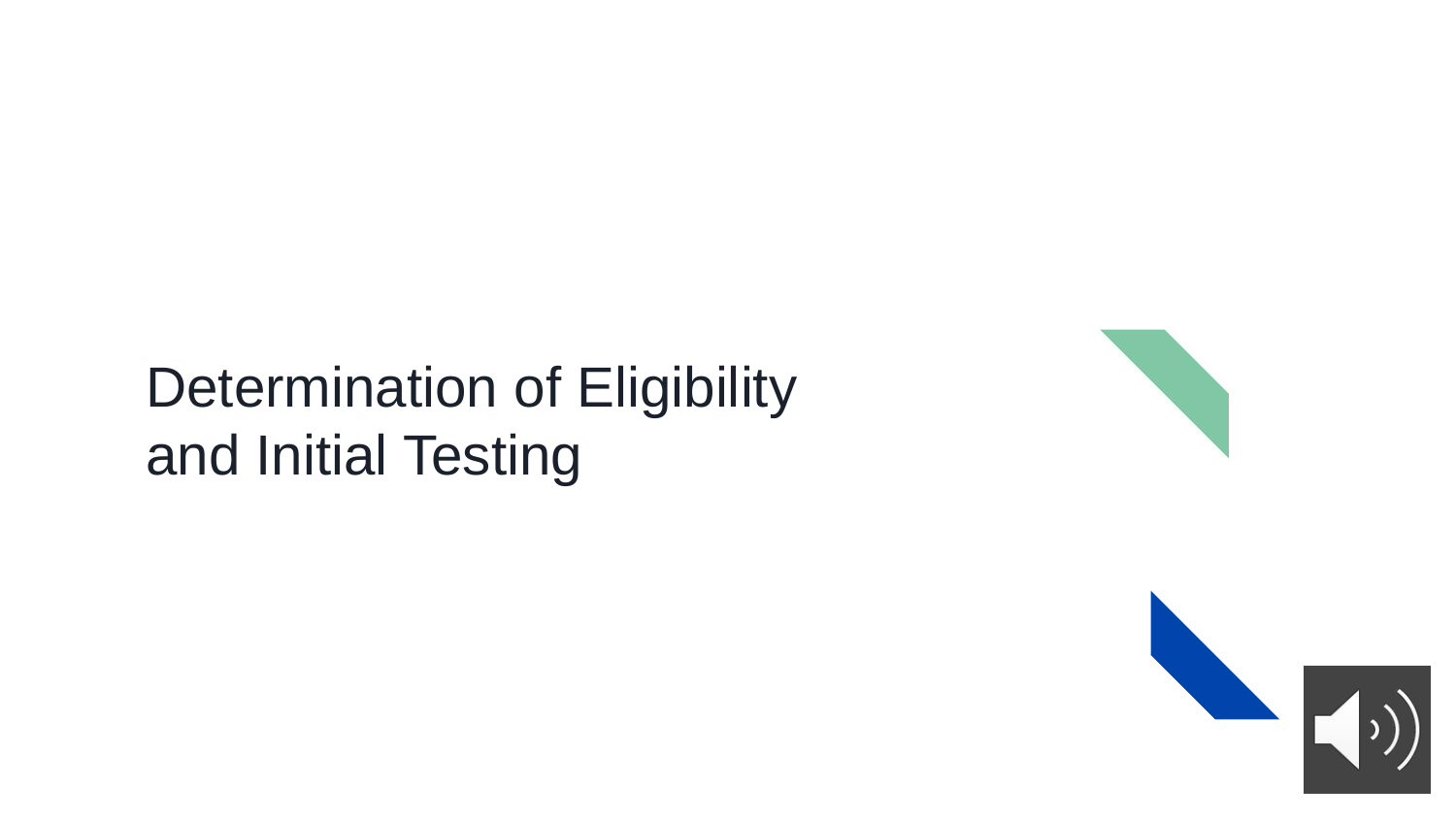

# Determination of Eligibility and Initial Testing
17

## Slide 18
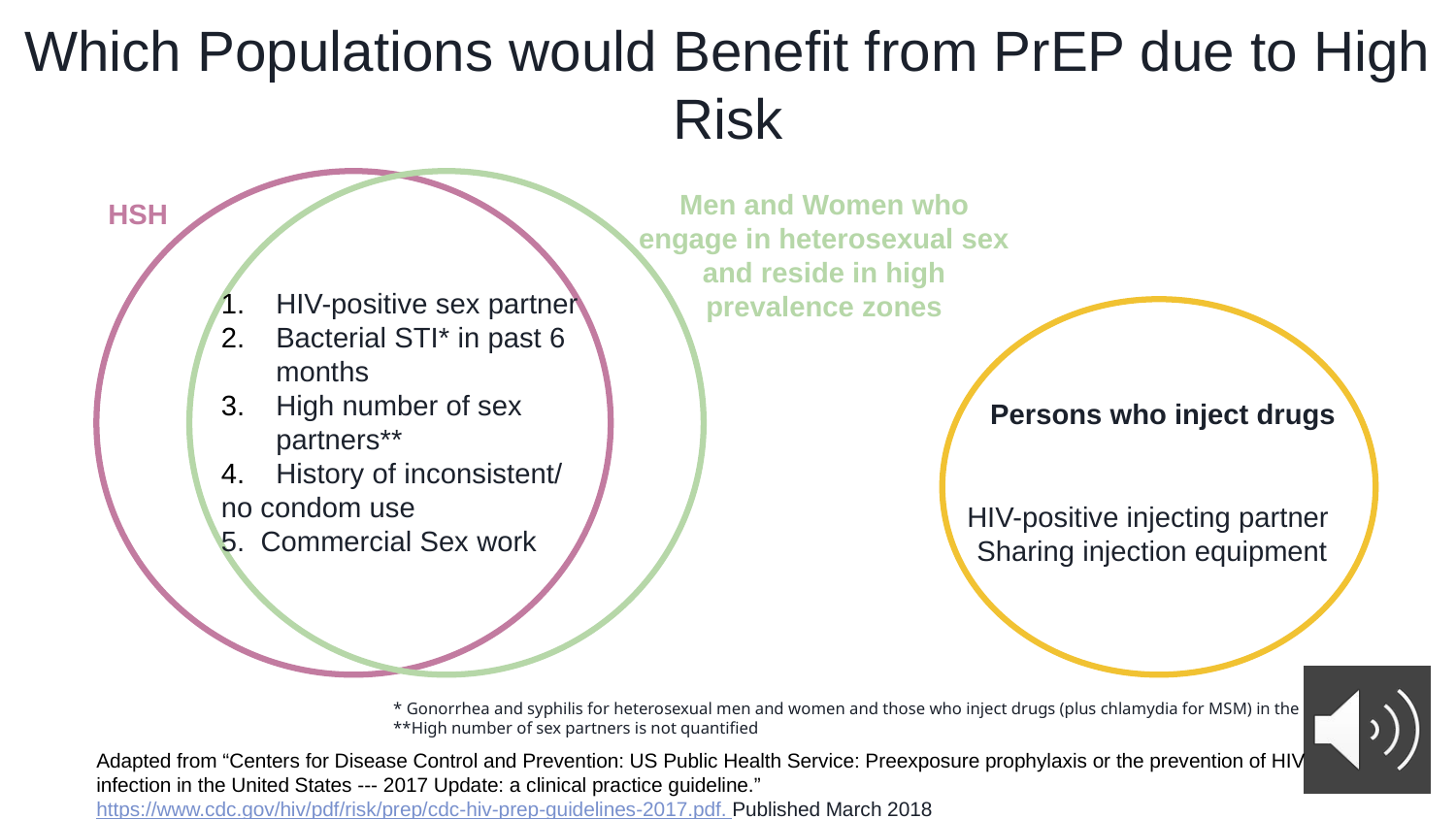

Which Populations would Benefit from PrEP due to High Risk
Men and Women who engage in heterosexual sex and reside in high prevalence zones
HSH
HIV-positive sex partner
Bacterial STI* in past 6 months
High number of sex partners**
History of inconsistent/
no condom use
5. Commercial Sex work
Persons who inject drugs
HIV-positive injecting partner Sharing injection equipment
* Gonorrhea and syphilis for heterosexual men and women and those who inject drugs (plus chlamydia for MSM) in the past 6 months
**High number of sex partners is not quantified
Adapted from “Centers for Disease Control and Prevention: US Public Health Service: Preexposure prophylaxis or the prevention of HIV infection in the United States --- 2017 Update: a clinical practice guideline.” https://www.cdc.gov/hiv/pdf/risk/prep/cdc-hiv-prep-guidelines-2017.pdf. Published March 2018
18

## Slide 19
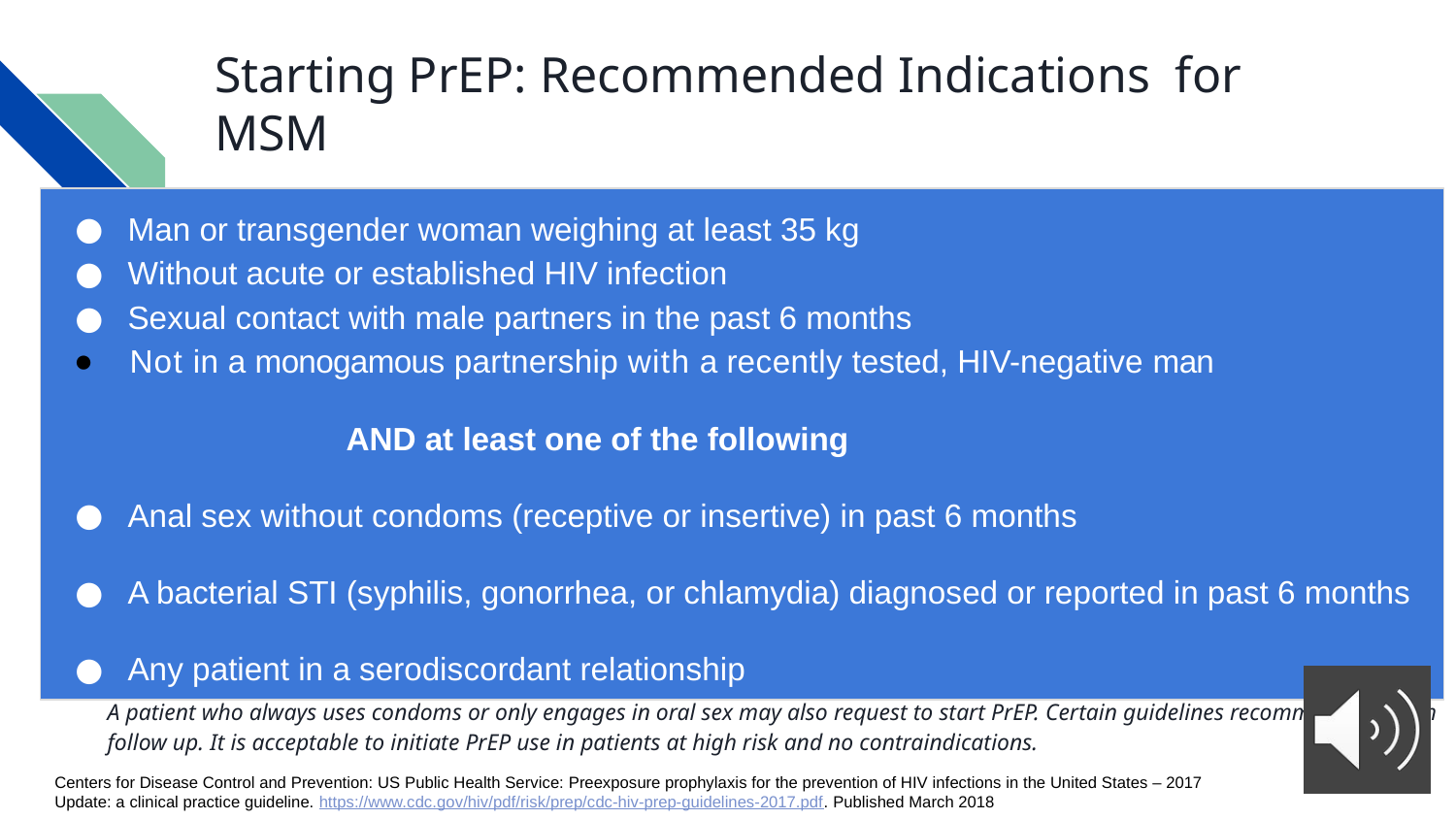

# Starting PrEP: Recommended Indications for MSM
Man or transgender woman weighing at least 35 kg
Without acute or established HIV infection
Sexual contact with male partners in the past 6 months
Not in a monogamous partnership with a recently tested, HIV-negative man
AND at least one of the following
Anal sex without condoms (receptive or insertive) in past 6 months
A bacterial STI (syphilis, gonorrhea, or chlamydia) diagnosed or reported in past 6 months
Any patient in a serodiscordant relationship
A patient who always uses condoms or only engages in oral sex may also request to start PrEP. Certain guidelines recommend a 3 onth follow up. It is acceptable to initiate PrEP use in patients at high risk and no contraindications.
19
Centers for Disease Control and Prevention: US Public Health Service: Preexposure prophylaxis for the prevention of HIV infections in the United States – 2017 Update: a clinical practice guideline. https://www.cdc.gov/hiv/pdf/risk/prep/cdc-hiv-prep-guidelines-2017.pdf. Published March 2018

## Slide 20
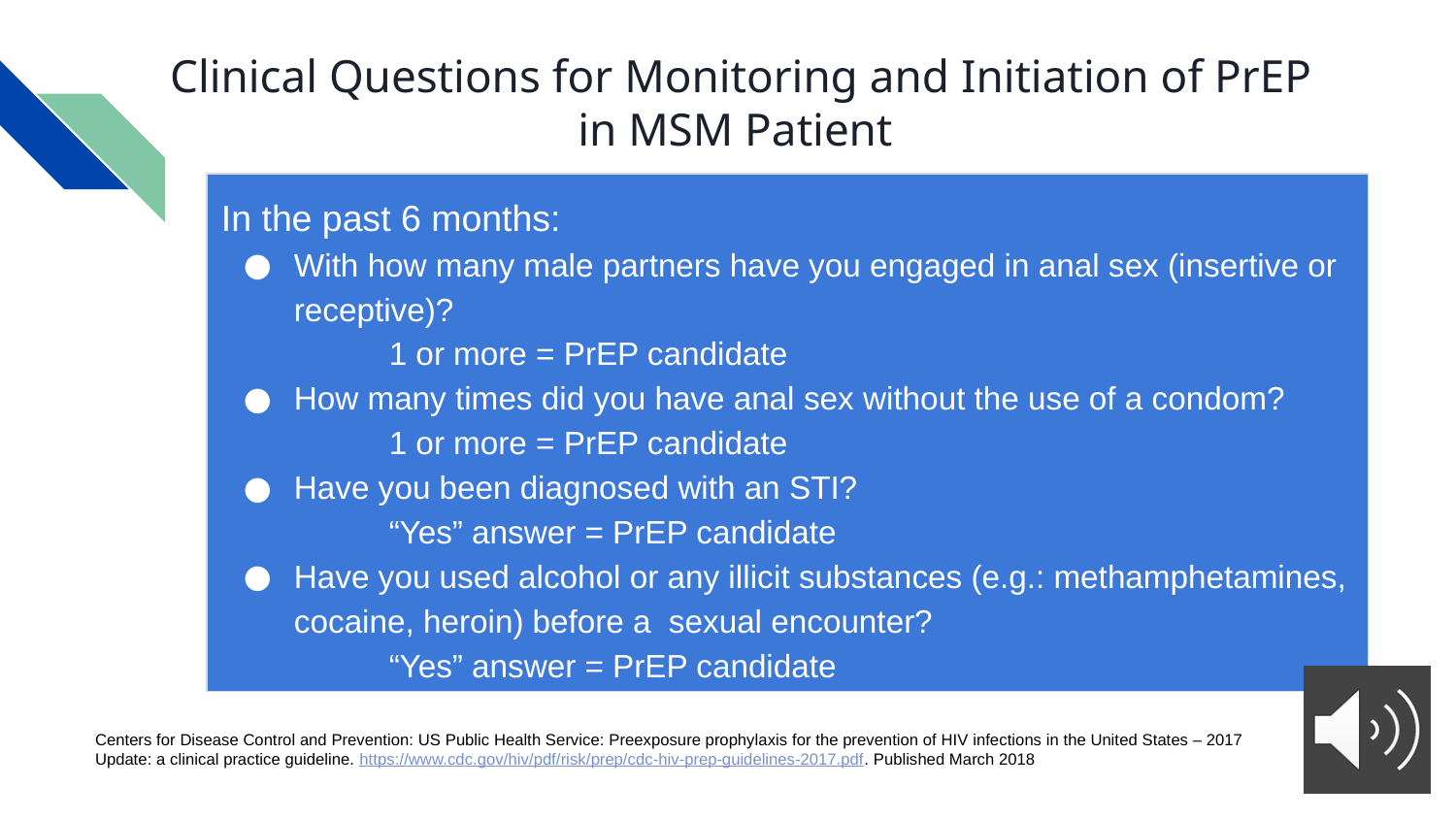

# Clinical Questions for Monitoring and Initiation of PrEP in MSM Patient
In the past 6 months:
With how many male partners have you engaged in anal sex (insertive or receptive)?
	1 or more = PrEP candidate
How many times did you have anal sex without the use of a condom?
	1 or more = PrEP candidate
Have you been diagnosed with an STI?
	“Yes” answer = PrEP candidate
Have you used alcohol or any illicit substances (e.g.: methamphetamines, cocaine, heroin) before a sexual encounter?
	“Yes” answer = PrEP candidate
Centers for Disease Control and Prevention: US Public Health Service: Preexposure prophylaxis for the prevention of HIV infections in the United States – 2017 Update: a clinical practice guideline. https://www.cdc.gov/hiv/pdf/risk/prep/cdc-hiv-prep-guidelines-2017.pdf. Published March 2018
20

## Slide 21
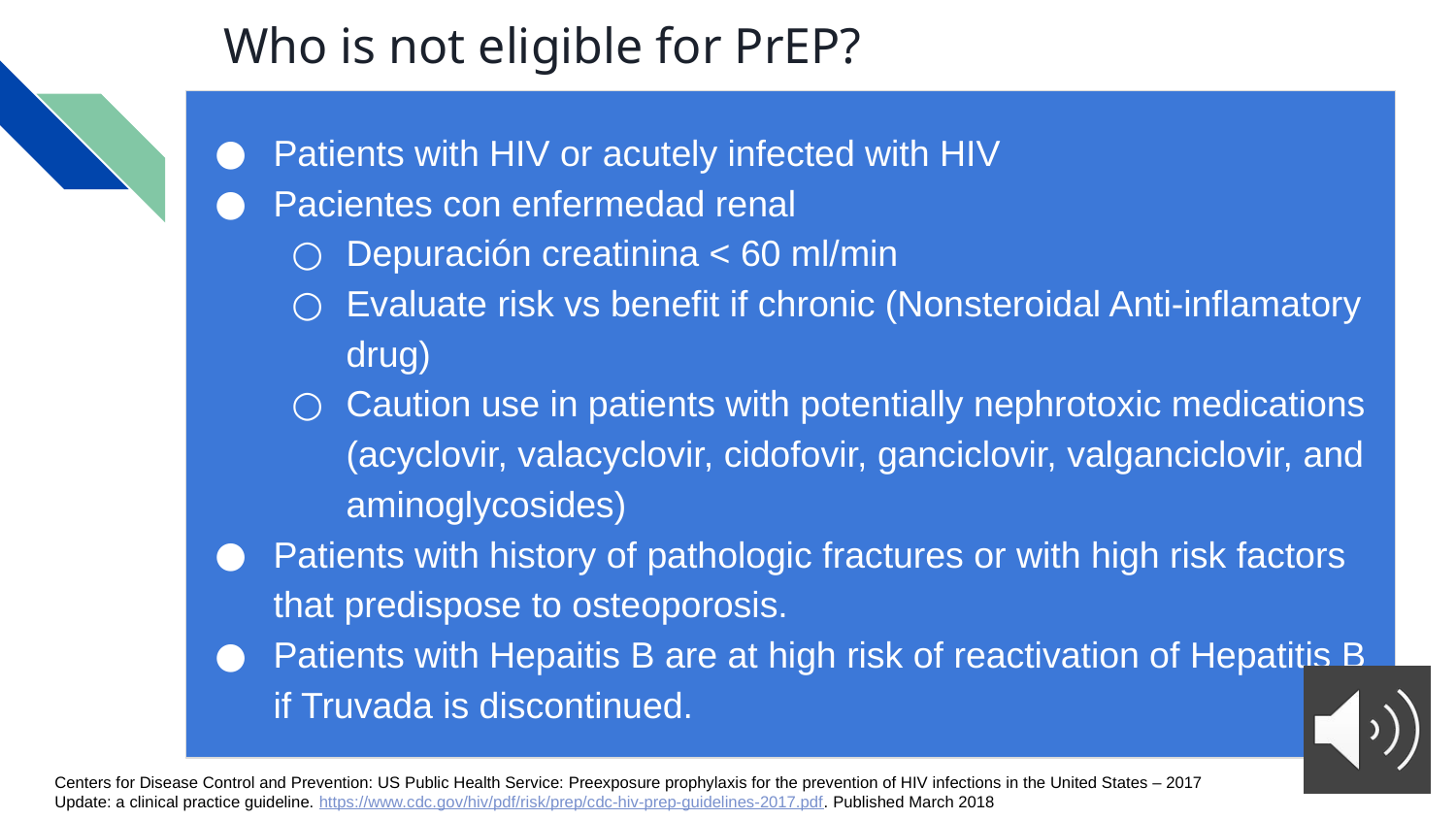

# Who is not eligible for PrEP?
Patients with HIV or acutely infected with HIV
Pacientes con enfermedad renal
Depuración creatinina < 60 ml/min
Evaluate risk vs benefit if chronic (Nonsteroidal Anti-inflamatory drug)
Caution use in patients with potentially nephrotoxic medications (acyclovir, valacyclovir, cidofovir, ganciclovir, valganciclovir, and aminoglycosides)
Patients with history of pathologic fractures or with high risk factors that predispose to osteoporosis.
Patients with Hepaitis B are at high risk of reactivation of Hepatitis B if Truvada is discontinued.
21
Centers for Disease Control and Prevention: US Public Health Service: Preexposure prophylaxis for the prevention of HIV infections in the United States – 2017 Update: a clinical practice guideline. https://www.cdc.gov/hiv/pdf/risk/prep/cdc-hiv-prep-guidelines-2017.pdf. Published March 2018

## Slide 22
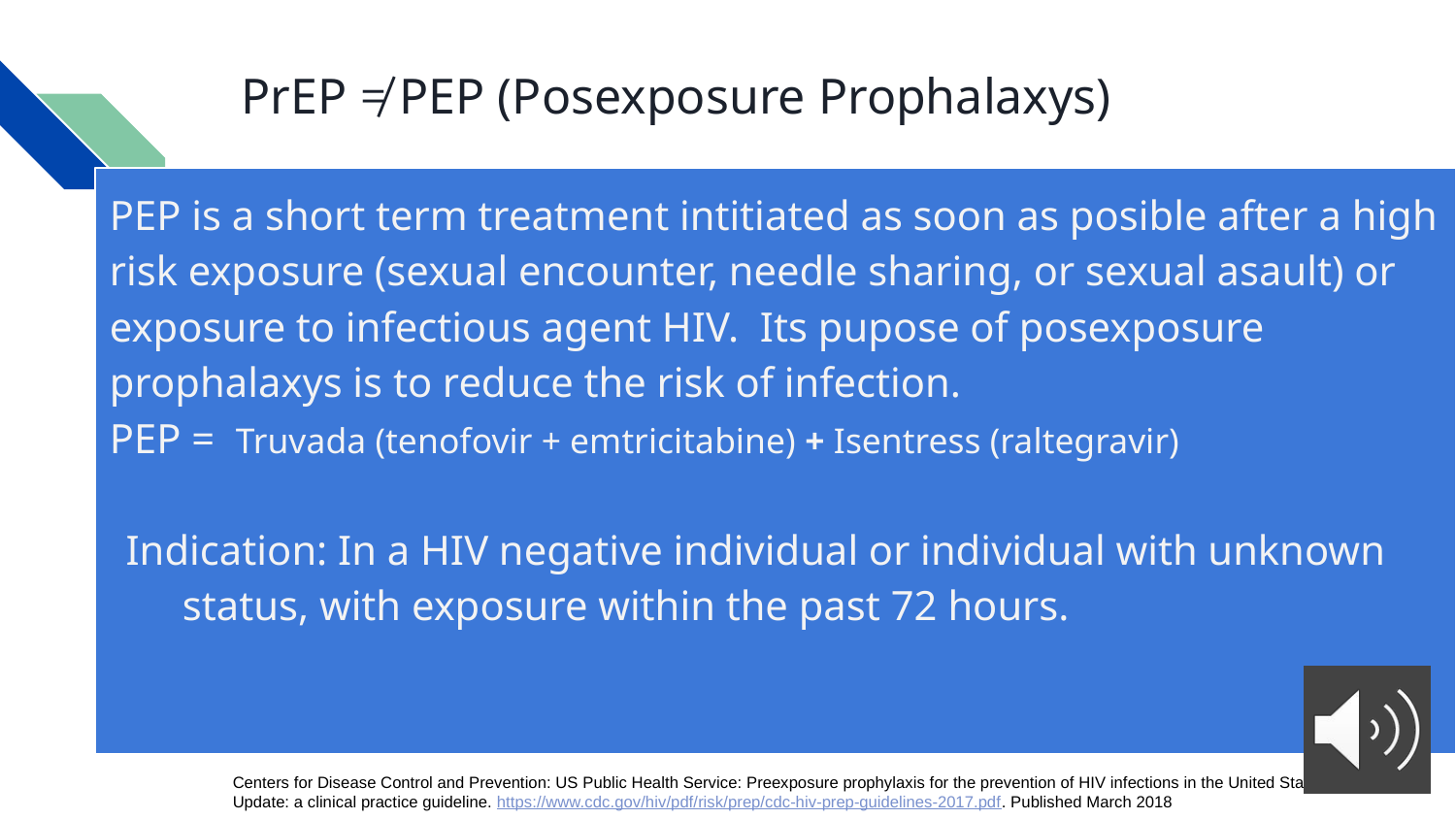

# PrEP ≠ PEP (Posexposure Prophalaxys)
PEP is a short term treatment intitiated as soon as posible after a high risk exposure (sexual encounter, needle sharing, or sexual asault) or exposure to infectious agent HIV. Its pupose of posexposure prophalaxys is to reduce the risk of infection.
PEP = Truvada (tenofovir + emtricitabine) + Isentress (raltegravir)
Indication: In a HIV negative individual or individual with unknown status, with exposure within the past 72 hours.
22
Centers for Disease Control and Prevention: US Public Health Service: Preexposure prophylaxis for the prevention of HIV infections in the United States – 2017 Update: a clinical practice guideline. https://www.cdc.gov/hiv/pdf/risk/prep/cdc-hiv-prep-guidelines-2017.pdf. Published March 2018

## Slide 23
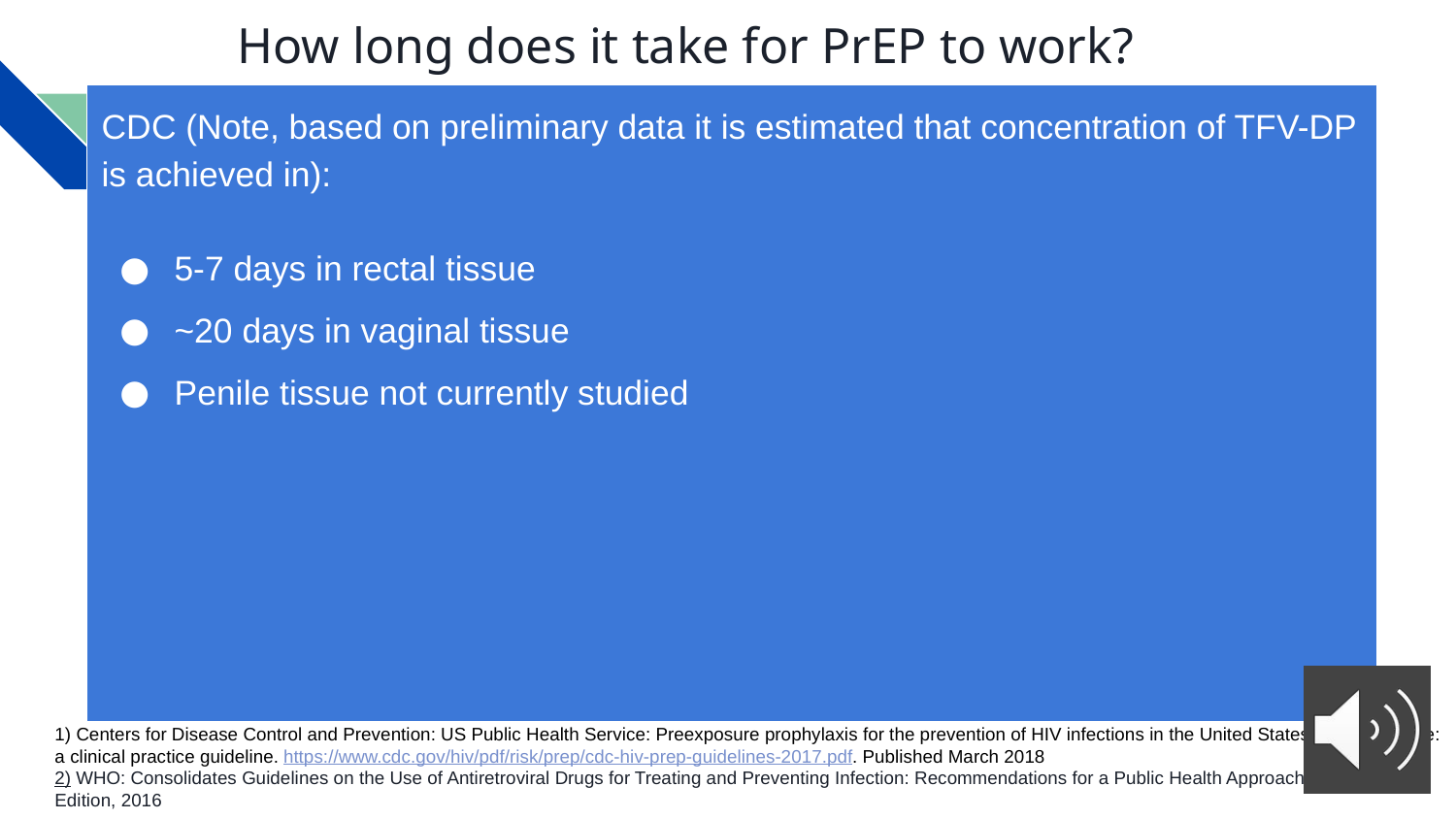

# How long does it take for PrEP to work?
CDC (Note, based on preliminary data it is estimated that concentration of TFV-DP is achieved in):
5-7 days in rectal tissue
~20 days in vaginal tissue
Penile tissue not currently studied
1) Centers for Disease Control and Prevention: US Public Health Service: Preexposure prophylaxis for the prevention of HIV infections in the United States – 2017 Update: a clinical practice guideline. https://www.cdc.gov/hiv/pdf/risk/prep/cdc-hiv-prep-guidelines-2017.pdf. Published March 2018
2) WHO: Consolidates Guidelines on the Use of Antiretroviral Drugs for Treating and Preventing Infection: Recommendations for a Public Health Approach, Second Edition, 2016
23

## Slide 24
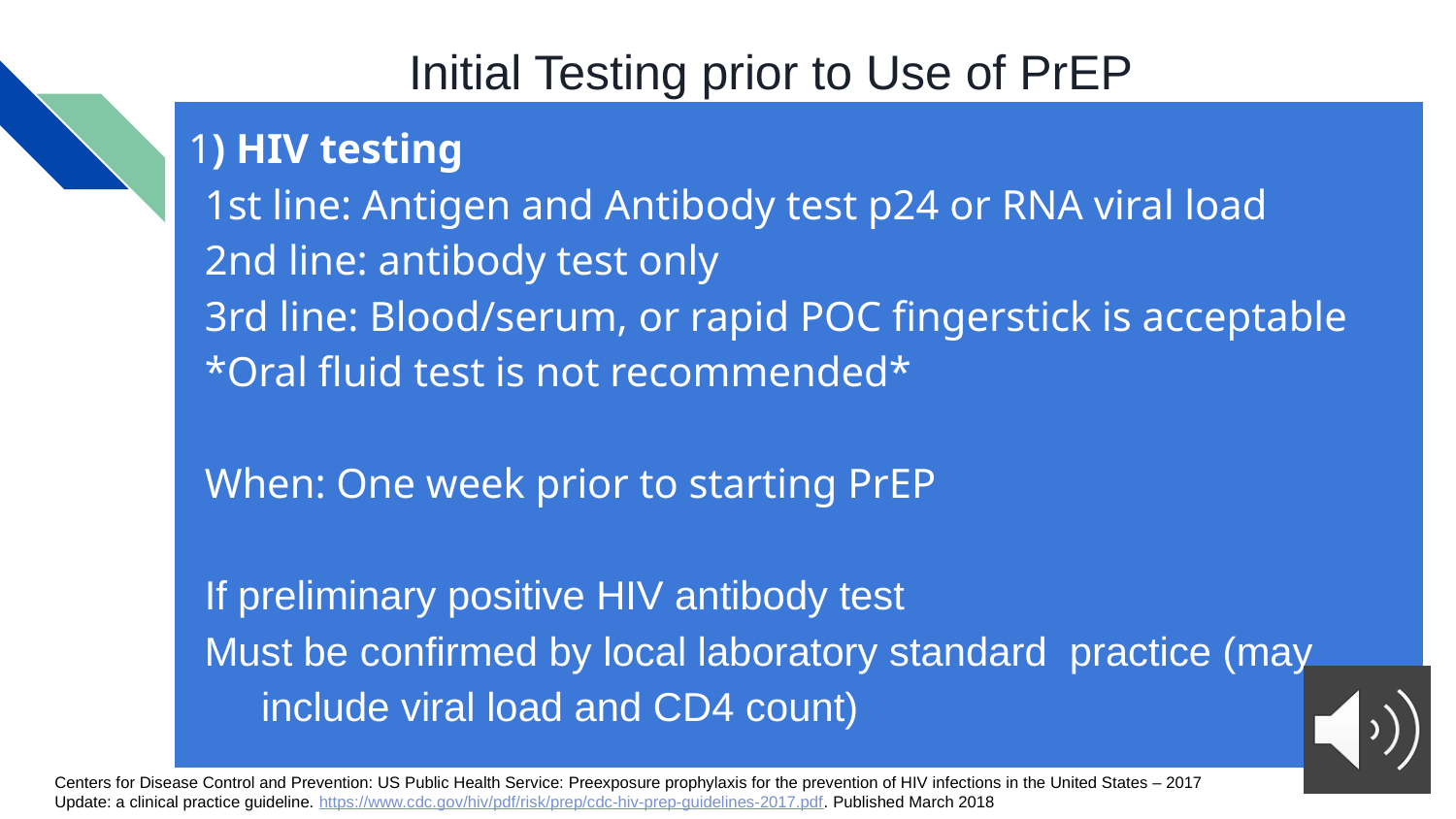

# Initial Testing prior to Use of PrEP
1) HIV testing
1st line: Antigen and Antibody test p24 or RNA viral load
2nd line: antibody test only
3rd line: Blood/serum, or rapid POC fingerstick is acceptable
*Oral fluid test is not recommended*
When: One week prior to starting PrEP
If preliminary positive HIV antibody test
Must be confirmed by local laboratory standard practice (may include viral load and CD4 count)
24
Centers for Disease Control and Prevention: US Public Health Service: Preexposure prophylaxis for the prevention of HIV infections in the United States – 2017 Update: a clinical practice guideline. https://www.cdc.gov/hiv/pdf/risk/prep/cdc-hiv-prep-guidelines-2017.pdf. Published March 2018

## Slide 25
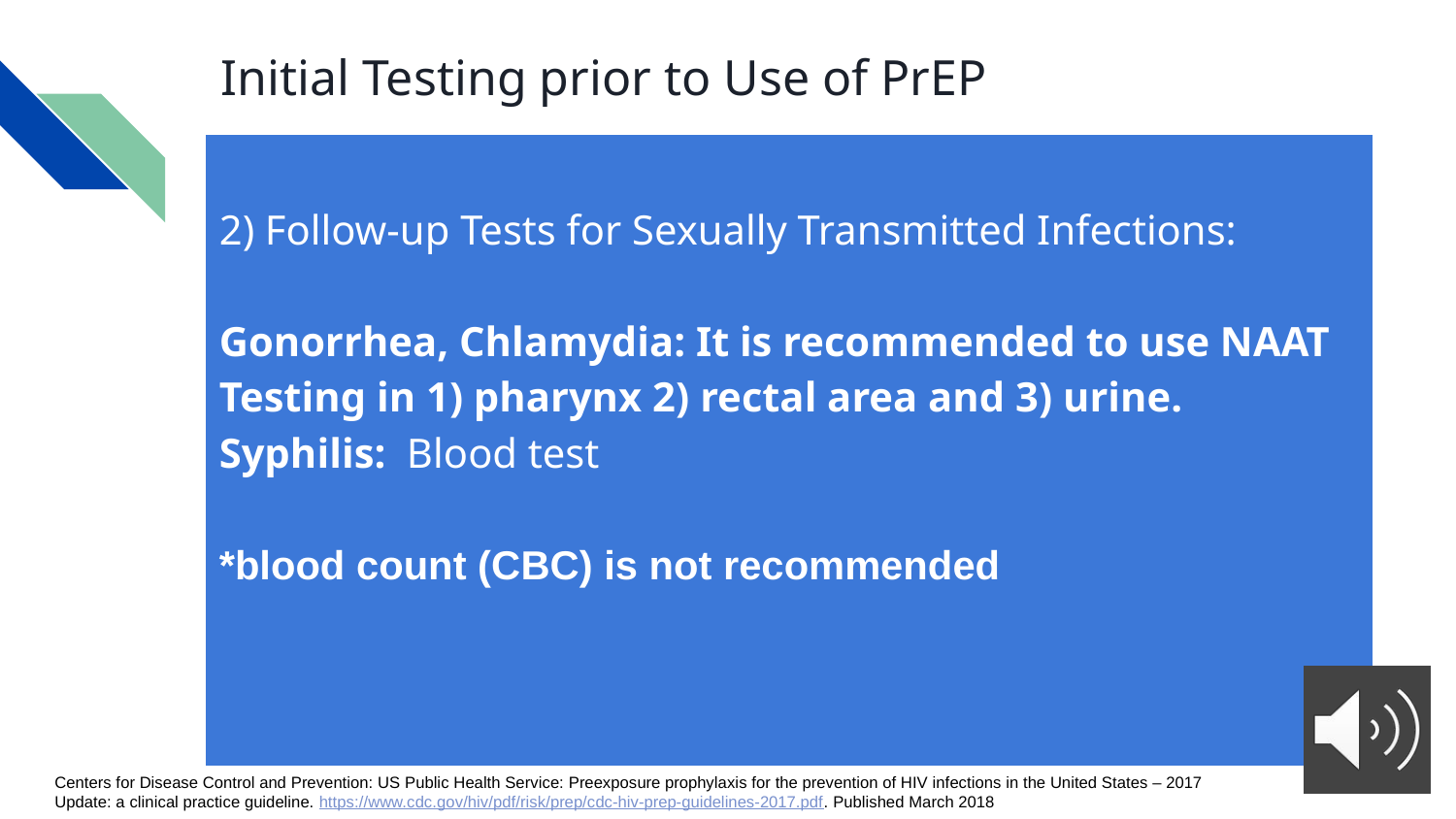

# Initial Testing prior to Use of PrEP
2) Follow-up Tests for Sexually Transmitted Infections:
Gonorrhea, Chlamydia: It is recommended to use NAAT Testing in 1) pharynx 2) rectal area and 3) urine.
Syphilis: Blood test
*blood count (CBC) is not recommended
25
Centers for Disease Control and Prevention: US Public Health Service: Preexposure prophylaxis for the prevention of HIV infections in the United States – 2017 Update: a clinical practice guideline. https://www.cdc.gov/hiv/pdf/risk/prep/cdc-hiv-prep-guidelines-2017.pdf. Published March 2018

## Slide 26
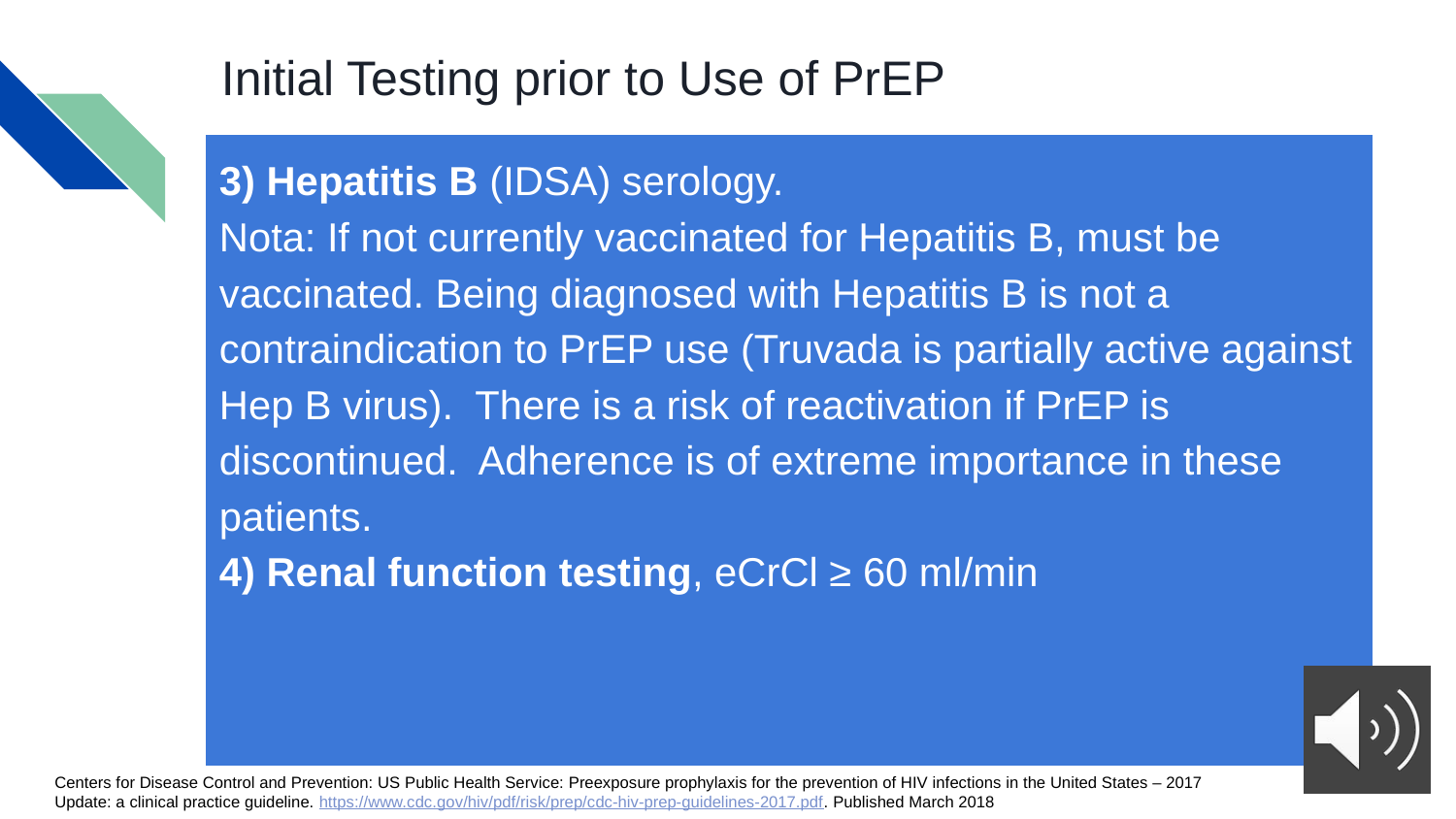

# Initial Testing prior to Use of PrEP
3) Hepatitis B (IDSA) serology.
Nota: If not currently vaccinated for Hepatitis B, must be vaccinated. Being diagnosed with Hepatitis B is not a contraindication to PrEP use (Truvada is partially active against Hep B virus). There is a risk of reactivation if PrEP is discontinued. Adherence is of extreme importance in these patients.
4) Renal function testing, eCrCl ≥ 60 ml/min
26
Centers for Disease Control and Prevention: US Public Health Service: Preexposure prophylaxis for the prevention of HIV infections in the United States – 2017 Update: a clinical practice guideline. https://www.cdc.gov/hiv/pdf/risk/prep/cdc-hiv-prep-guidelines-2017.pdf. Published March 2018

## Slide 27
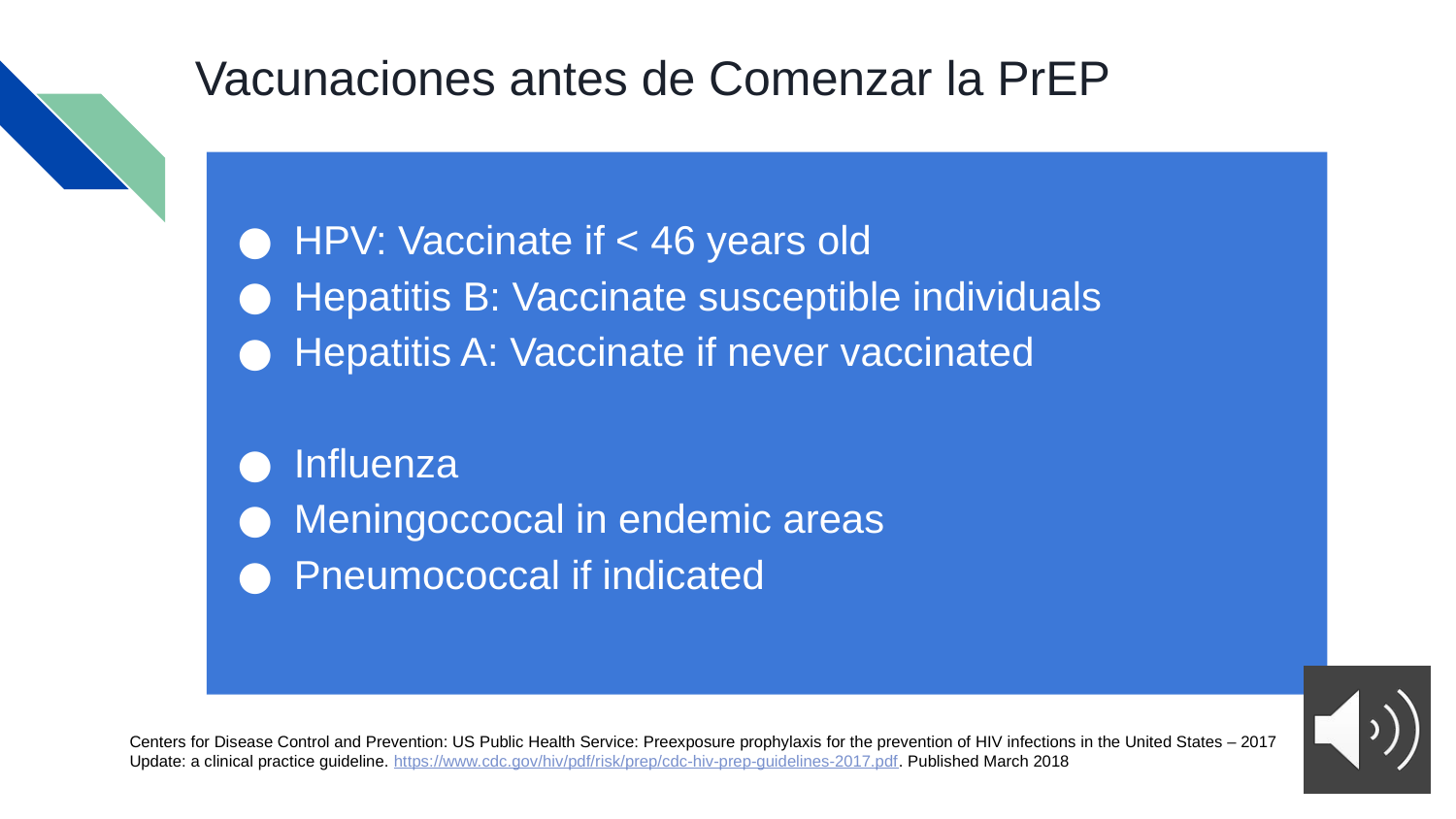

# Vacunaciones antes de Comenzar la PrEP
HPV: Vaccinate if < 46 years old
Hepatitis B: Vaccinate susceptible individuals
Hepatitis A: Vaccinate if never vaccinated
Influenza
Meningoccocal in endemic areas
Pneumococcal if indicated
Centers for Disease Control and Prevention: US Public Health Service: Preexposure prophylaxis for the prevention of HIV infections in the United States – 2017 Update: a clinical practice guideline. https://www.cdc.gov/hiv/pdf/risk/prep/cdc-hiv-prep-guidelines-2017.pdf. Published March 2018
27

## Slide 28
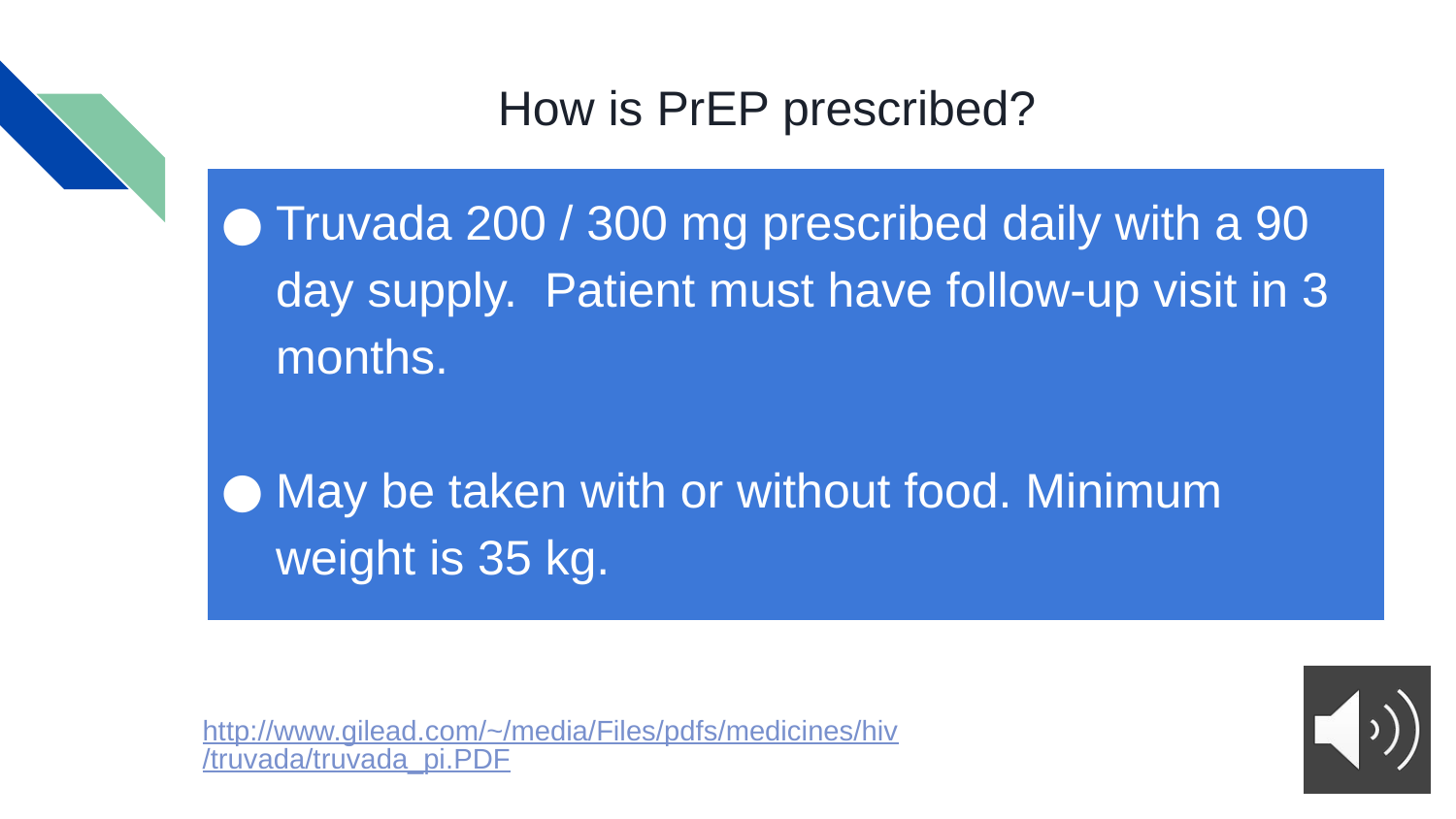

# How is PrEP prescribed?
Truvada 200 / 300 mg prescribed daily with a 90 day supply. Patient must have follow-up visit in 3 months.
May be taken with or without food. Minimum weight is 35 kg.
http://www.gilead.com/~/media/Files/pdfs/medicines/hiv/truvada/truvada_pi.PDF
28

## Slide 29
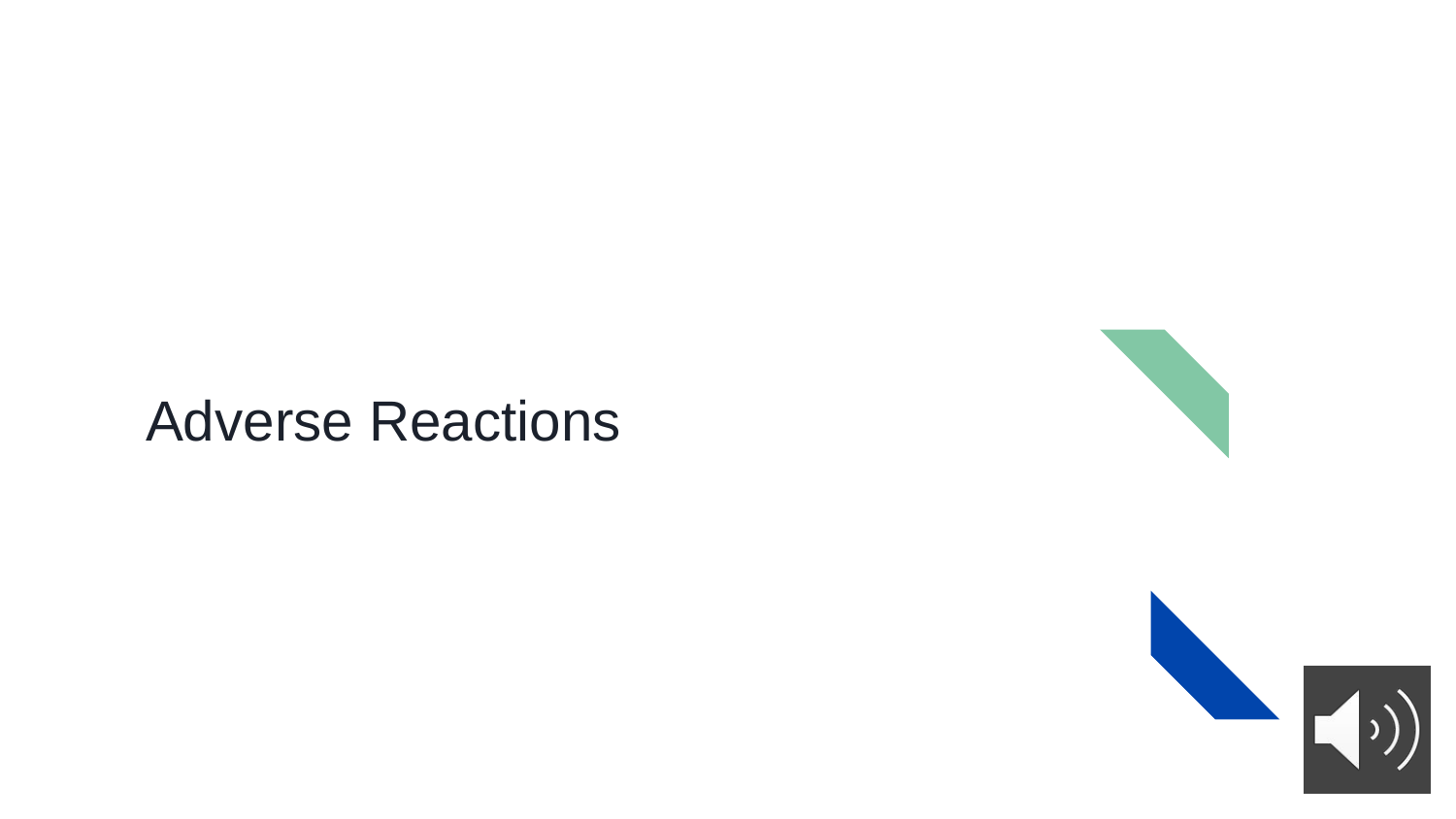

# Adverse Reactions
29

## Slide 30
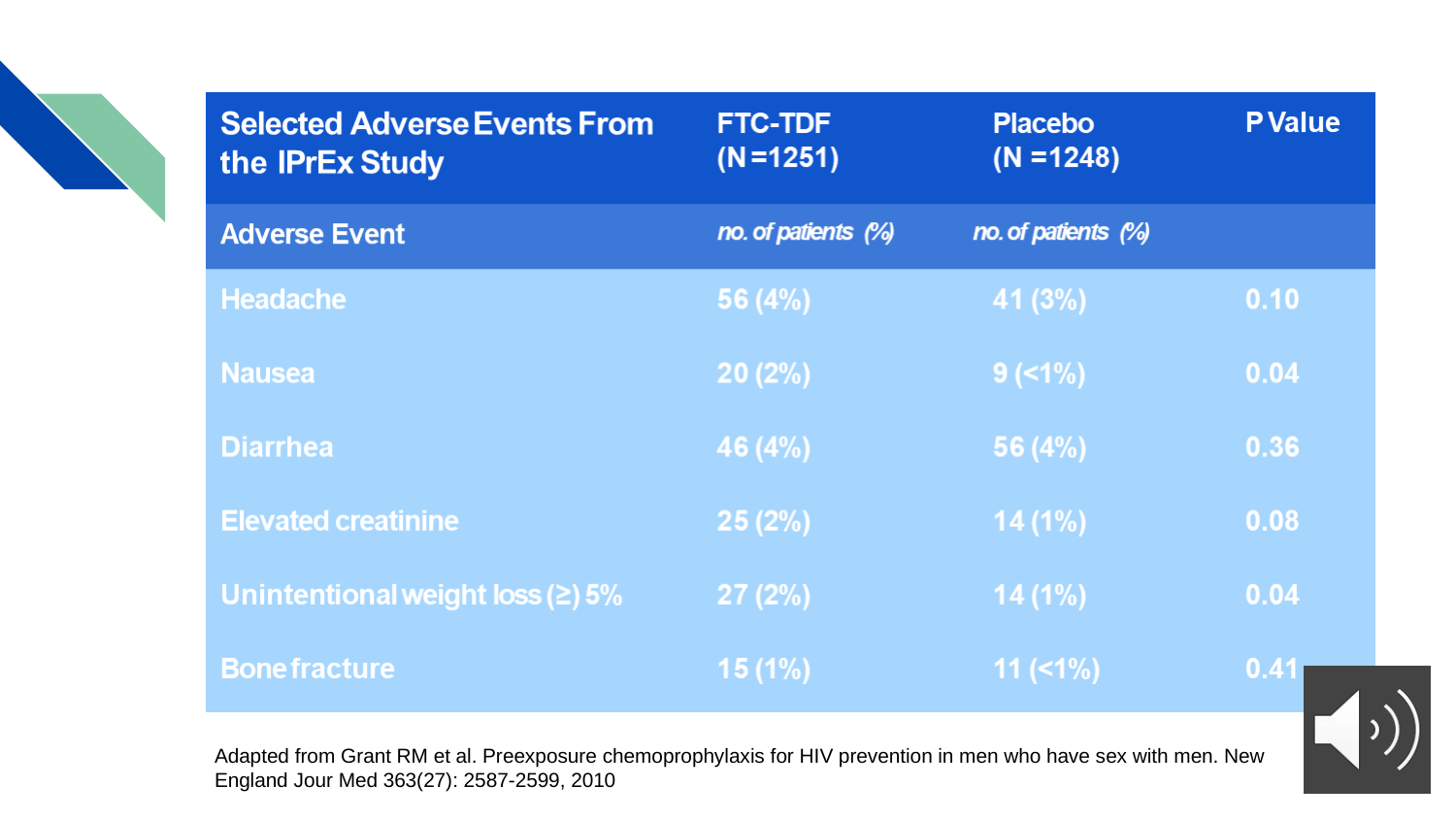

# PrEP Side Effects and Adverse Reactions
Adapted from Grant RM et al. Preexposure chemoprophylaxis for HIV prevention in men who have sex with men. New England Jour Med 363(27): 2587-2599, 2010
30

## Slide 31
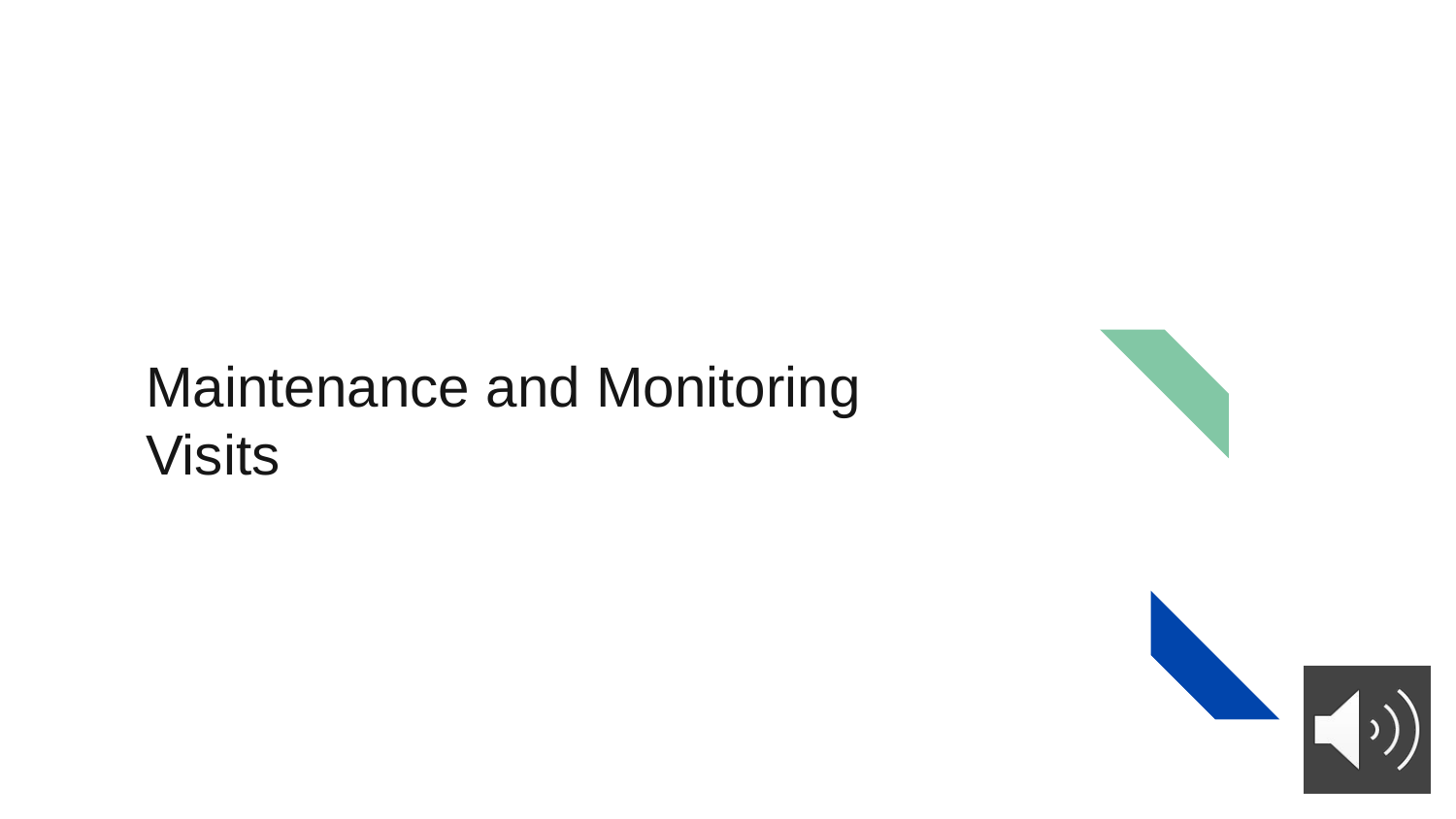

# Maintenance and Monitoring Visits
31

## Slide 32
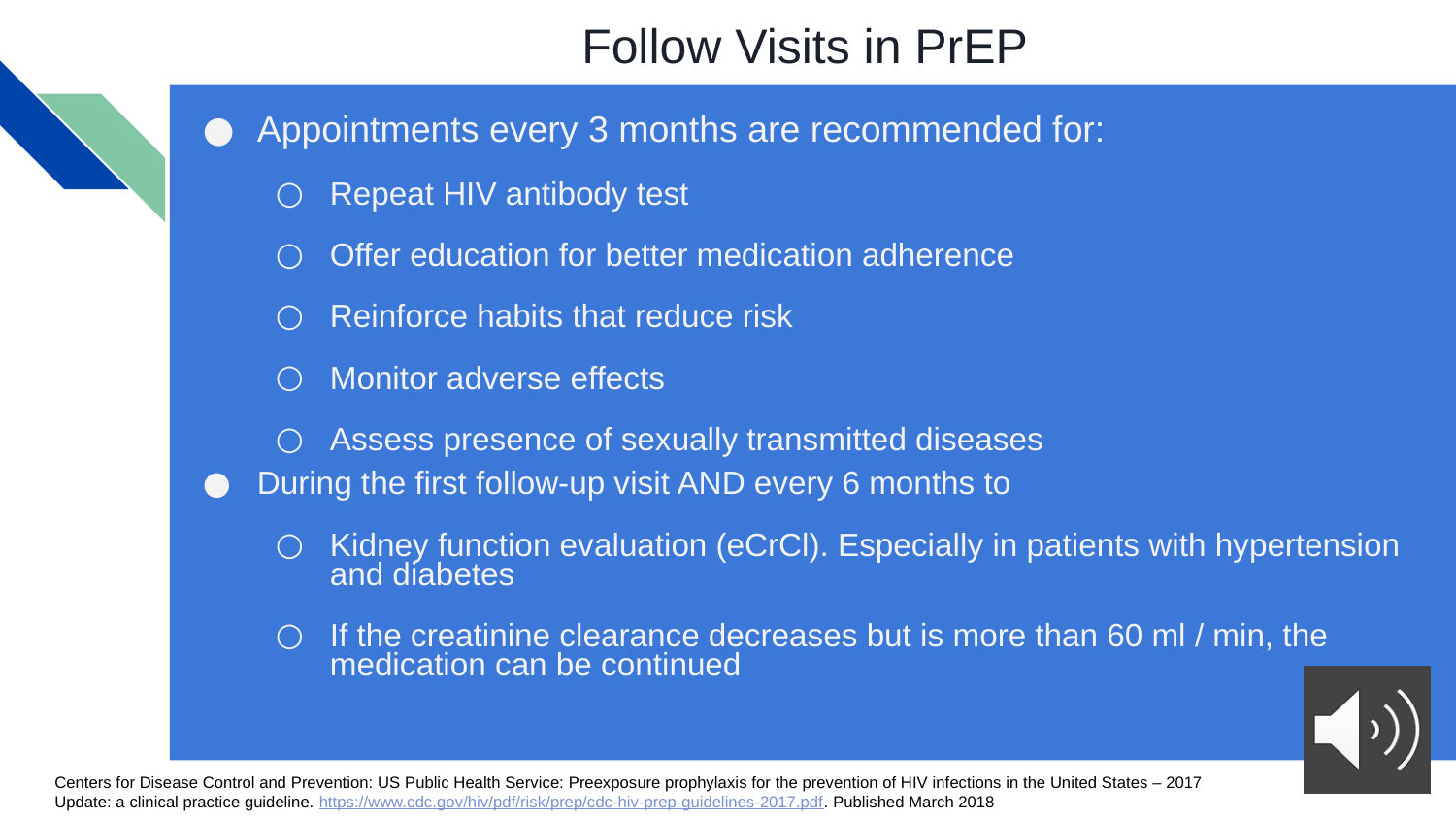

# Follow Visits in PrEP
Appointments every 3 months are recommended for:
Repeat HIV antibody test
Offer education for better medication adherence
Reinforce habits that reduce risk
Monitor adverse effects
Assess presence of sexually transmitted diseases
During the first follow-up visit AND every 6 months to
Kidney function evaluation (eCrCl). Especially in patients with hypertension and diabetes
If the creatinine clearance decreases but is more than 60 ml / min, the medication can be continued
32
Centers for Disease Control and Prevention: US Public Health Service: Preexposure prophylaxis for the prevention of HIV infections in the United States – 2017 Update: a clinical practice guideline. https://www.cdc.gov/hiv/pdf/risk/prep/cdc-hiv-prep-guidelines-2017.pdf. Published March 2018

## Slide 33
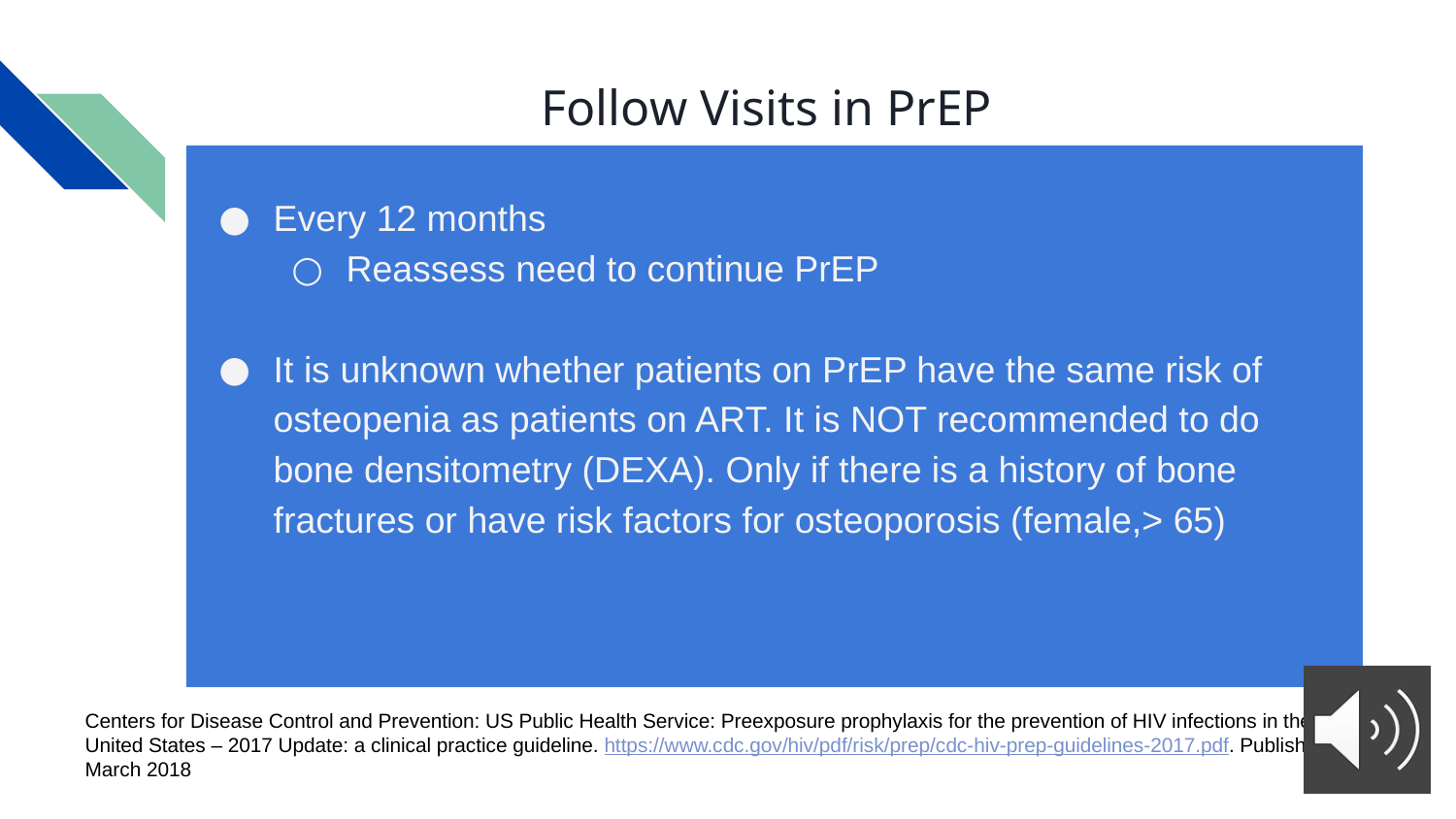

# Follow Visits in PrEP
Every 12 months
Reassess need to continue PrEP
It is unknown whether patients on PrEP have the same risk of osteopenia as patients on ART. It is NOT recommended to do bone densitometry (DEXA). Only if there is a history of bone fractures or have risk factors for osteoporosis (female,> 65)
Centers for Disease Control and Prevention: US Public Health Service: Preexposure prophylaxis for the prevention of HIV infections in the United States – 2017 Update: a clinical practice guideline. https://www.cdc.gov/hiv/pdf/risk/prep/cdc-hiv-prep-guidelines-2017.pdf. Published March 2018
33

## Slide 34
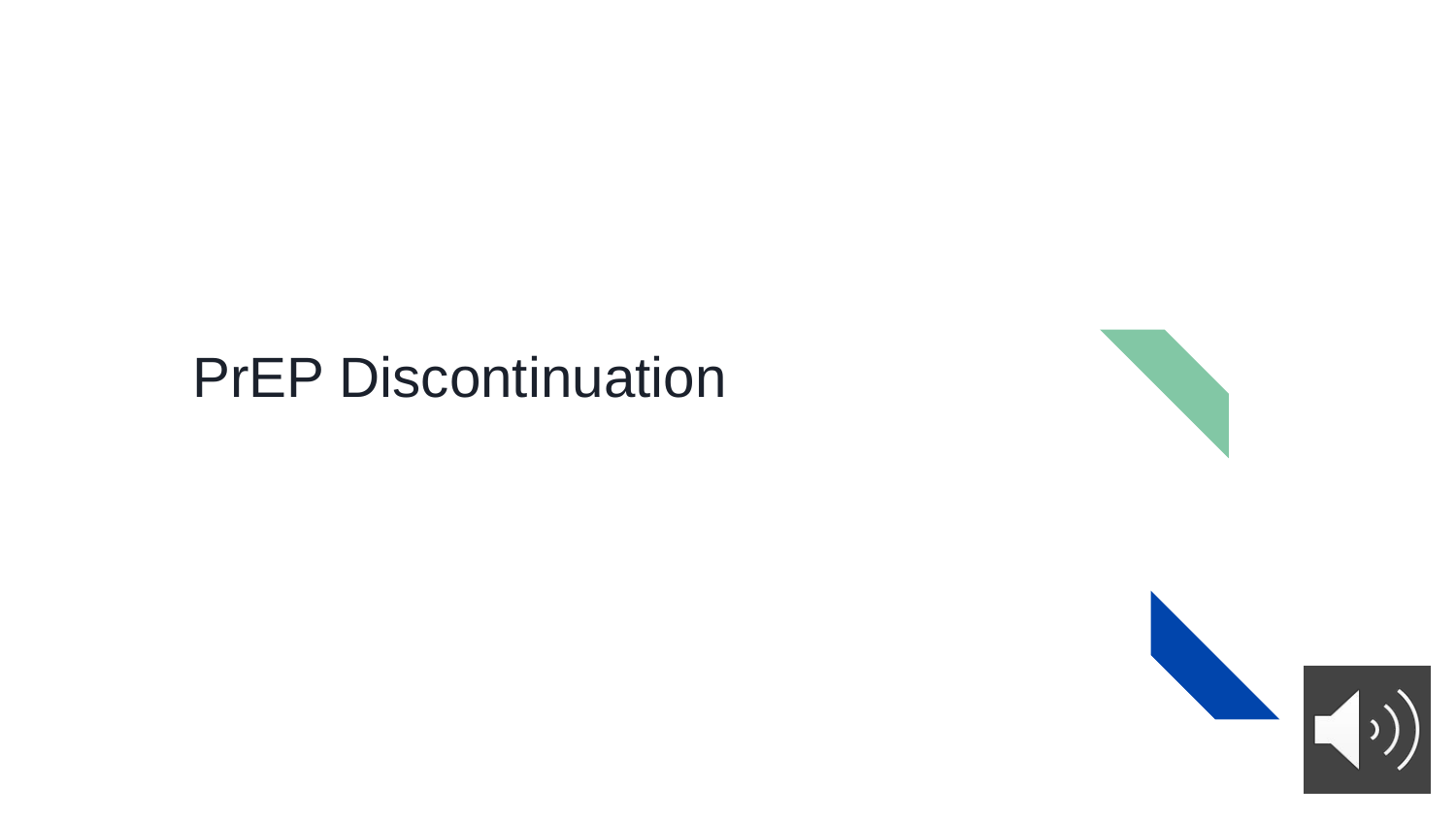

# PrEP Discontinuation
34

## Slide 35
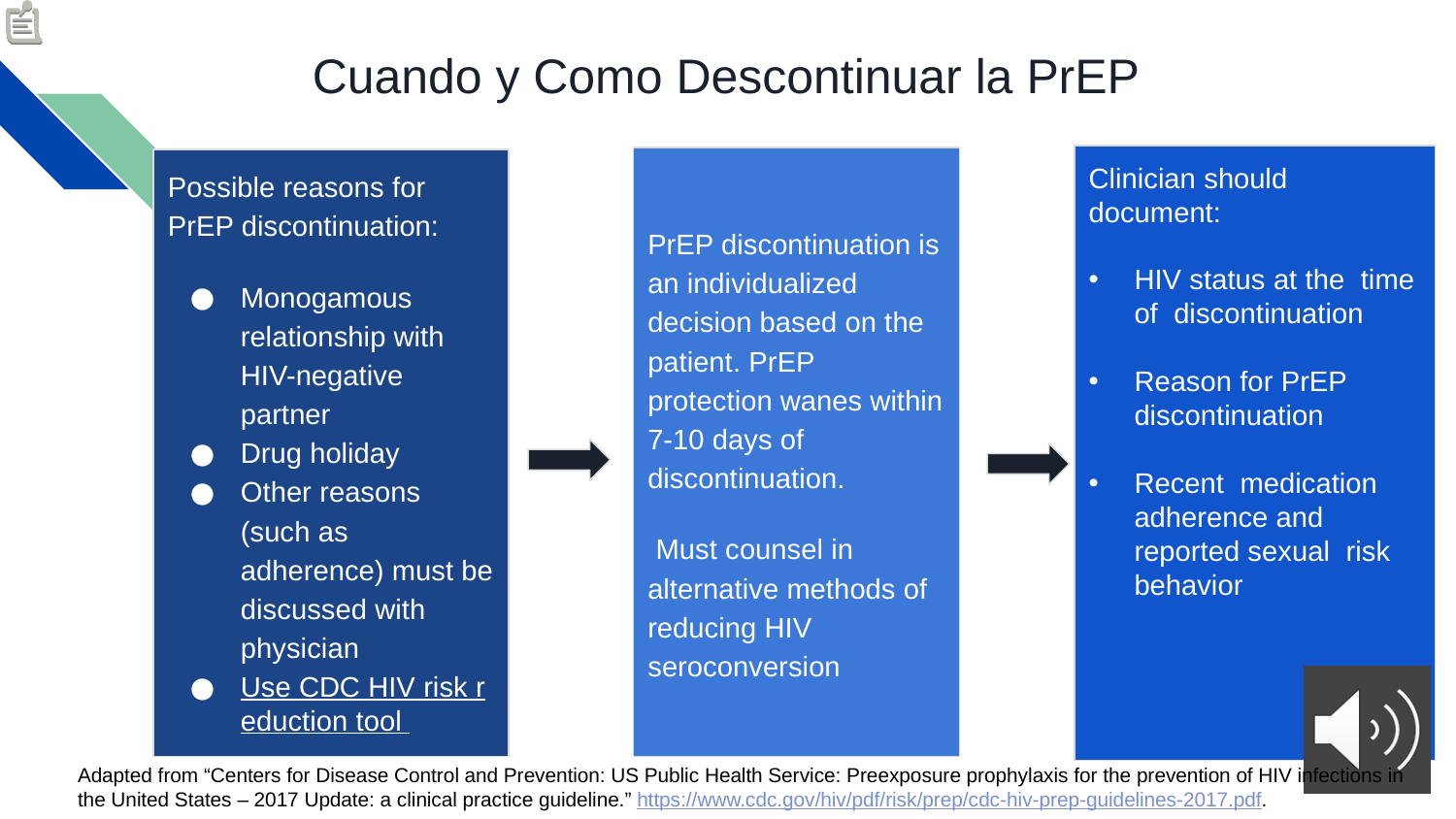

# Cuando y Como Descontinuar la PrEP
Clinician should document:
HIV status at the time of discontinuation
Reason for PrEP discontinuation
Recent medication adherence and reported sexual risk behavior
PrEP discontinuation is an individualized decision based on the patient. PrEP protection wanes within 7-10 days of discontinuation.
 Must counsel in alternative methods of reducing HIV seroconversion
Possible reasons for PrEP discontinuation:
Monogamous relationship with HIV-negative partner
Drug holiday
Other reasons (such as adherence) must be discussed with physician
Use CDC HIV risk reduction tool
35
Adapted from “Centers for Disease Control and Prevention: US Public Health Service: Preexposure prophylaxis for the prevention of HIV infections in the United States – 2017 Update: a clinical practice guideline.” https://www.cdc.gov/hiv/pdf/risk/prep/cdc-hiv-prep-guidelines-2017.pdf.

## Slide 36
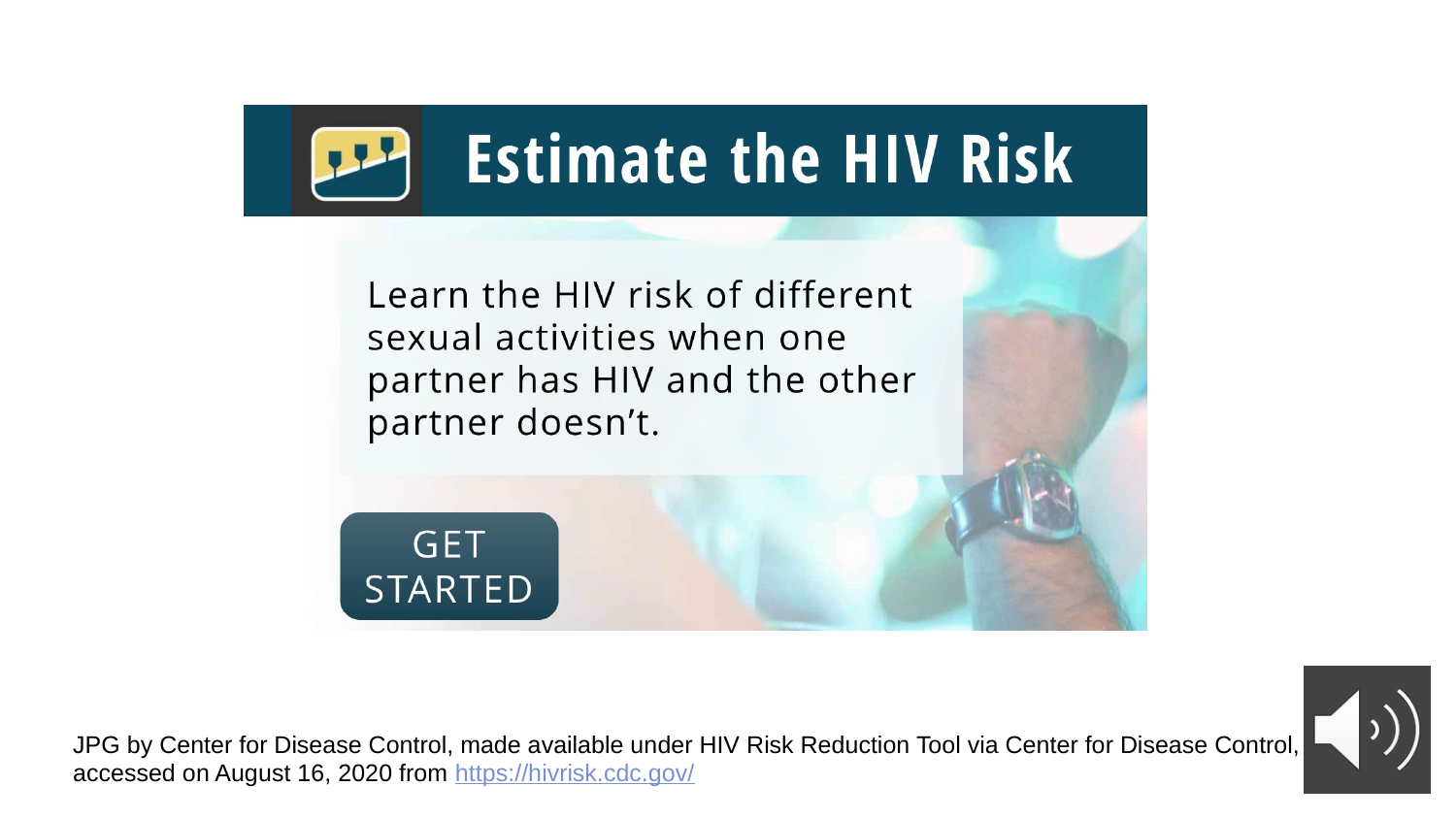

Click Here
JPG by Center for Disease Control, made available under HIV Risk Reduction Tool via Center for Disease Control, accessed on August 16, 2020 from https://hivrisk.cdc.gov/
36

## Slide 37
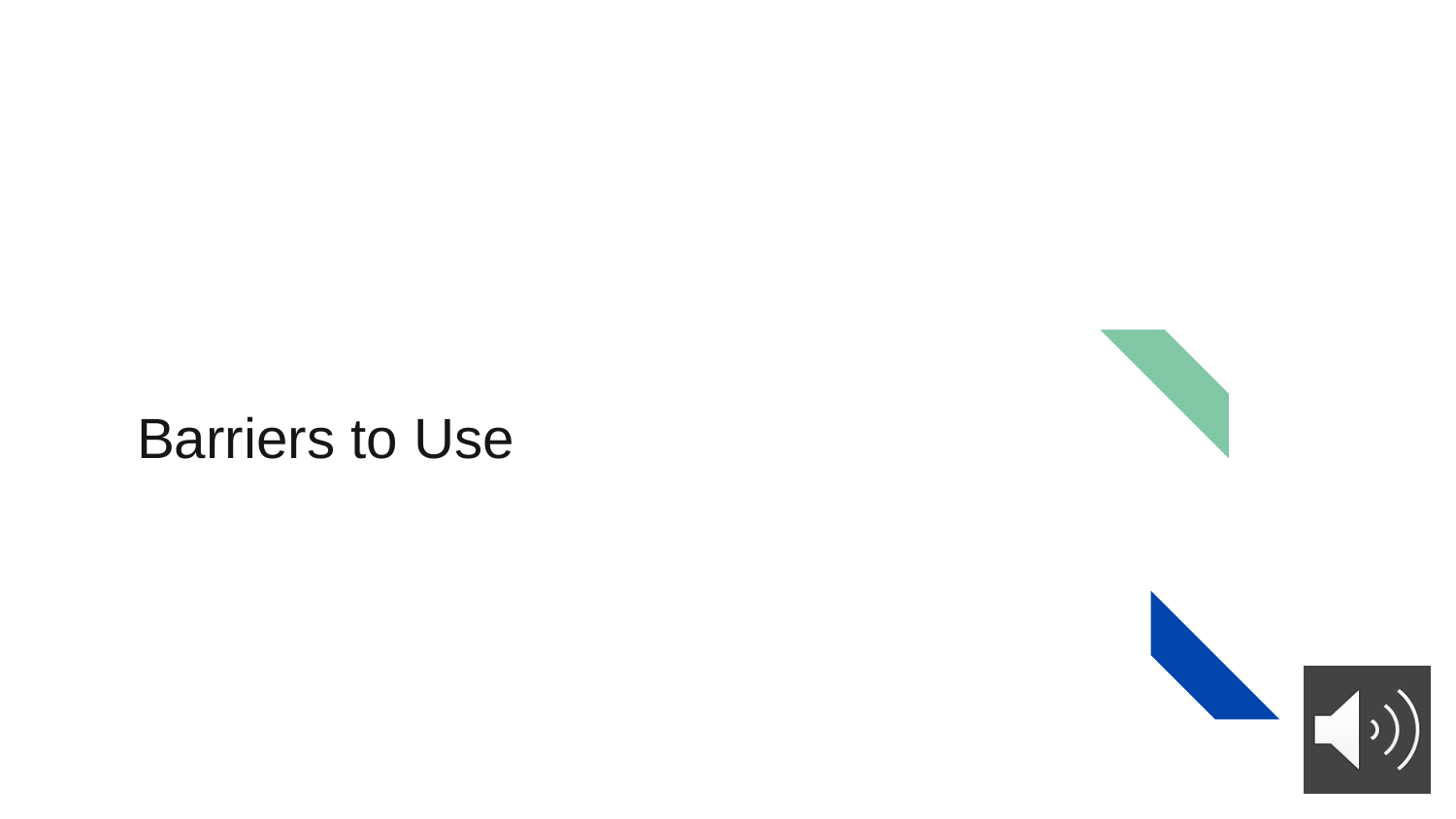

# Barriers to Use
37

## Slide 38
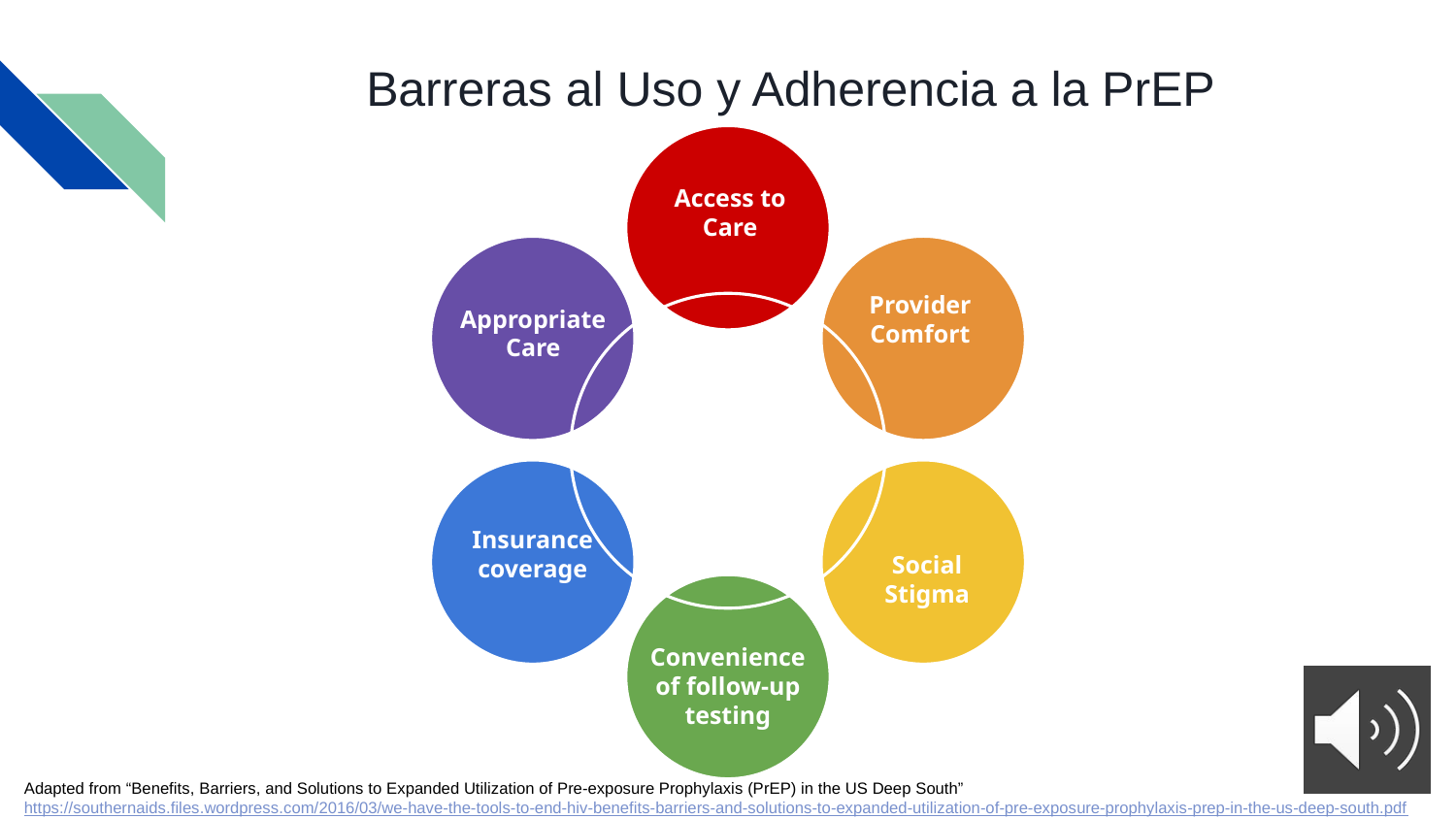

# Barreras al Uso y Adherencia a la PrEP
Access to Care
Provider Comfort
Appropriate Care
Uptake and Adherence to PrEP
Social Stigma
Insurance coverage
Convenience of follow-up testing
38
Adapted from “Benefits, Barriers, and Solutions to Expanded Utilization of Pre-exposure Prophylaxis (PrEP) in the US Deep South” https://southernaids.files.wordpress.com/2016/03/we-have-the-tools-to-end-hiv-benefits-barriers-and-solutions-to-expanded-utilization-of-pre-exposure-prophylaxis-prep-in-the-us-deep-south.pdf

## Slide 39
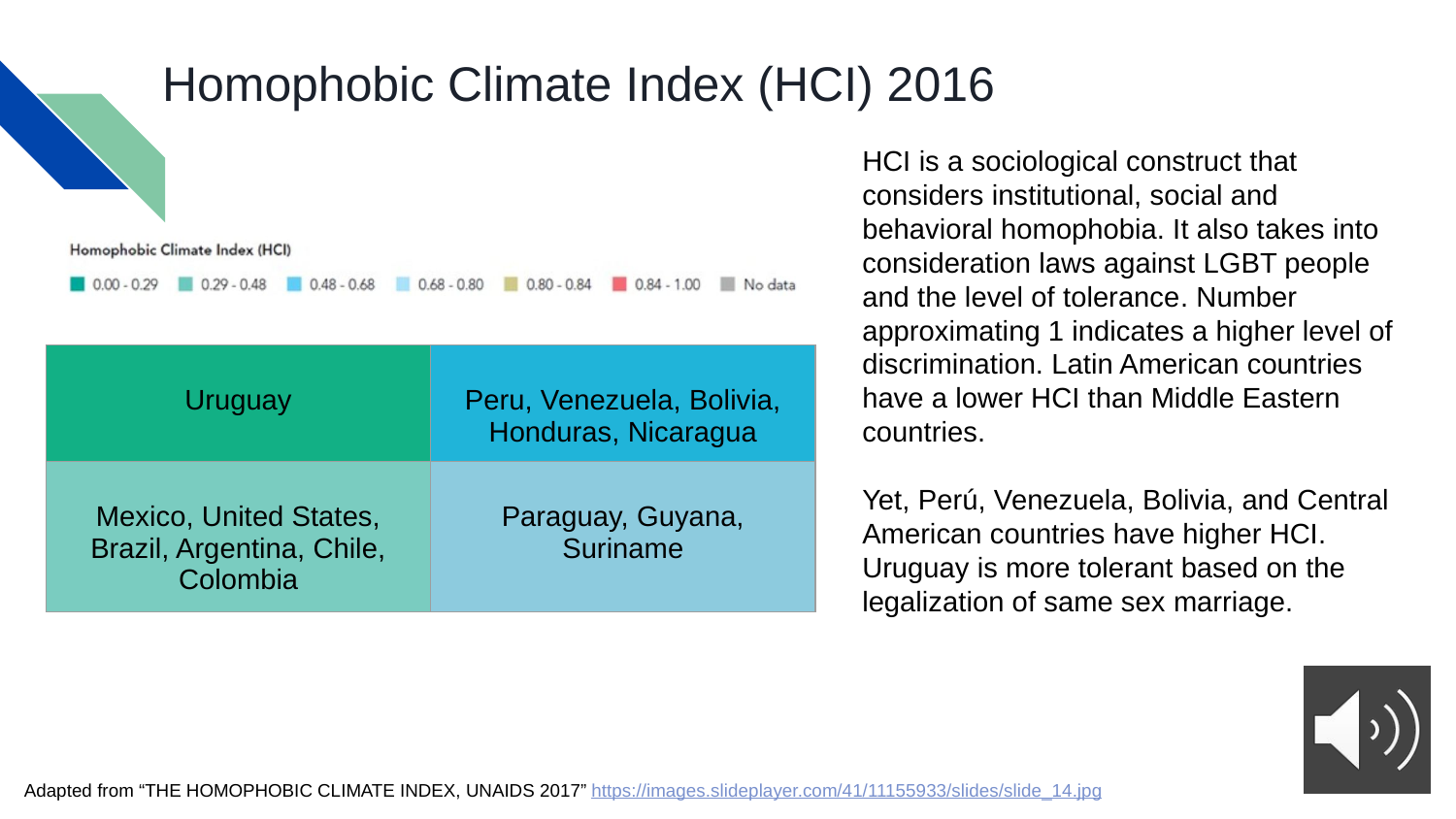

# Homophobic Climate Index (HCI) 2016
HCI is a sociological construct that considers institutional, social and behavioral homophobia. It also takes into consideration laws against LGBT people and the level of tolerance. Number approximating 1 indicates a higher level of discrimination. Latin American countries have a lower HCI than Middle Eastern countries.
Yet, Perú, Venezuela, Bolivia, and Central American countries have higher HCI. Uruguay is more tolerant based on the legalization of same sex marriage.
| Uruguay | Peru, Venezuela, Bolivia, Honduras, Nicaragua |
| --- | --- |
| Mexico, United States, Brazil, Argentina, Chile, Colombia | Paraguay, Guyana, Suriname |
39
Adapted from “THE HOMOPHOBIC CLIMATE INDEX, UNAIDS 2017” https://images.slideplayer.com/41/11155933/slides/slide_14.jpg

## Slide 40
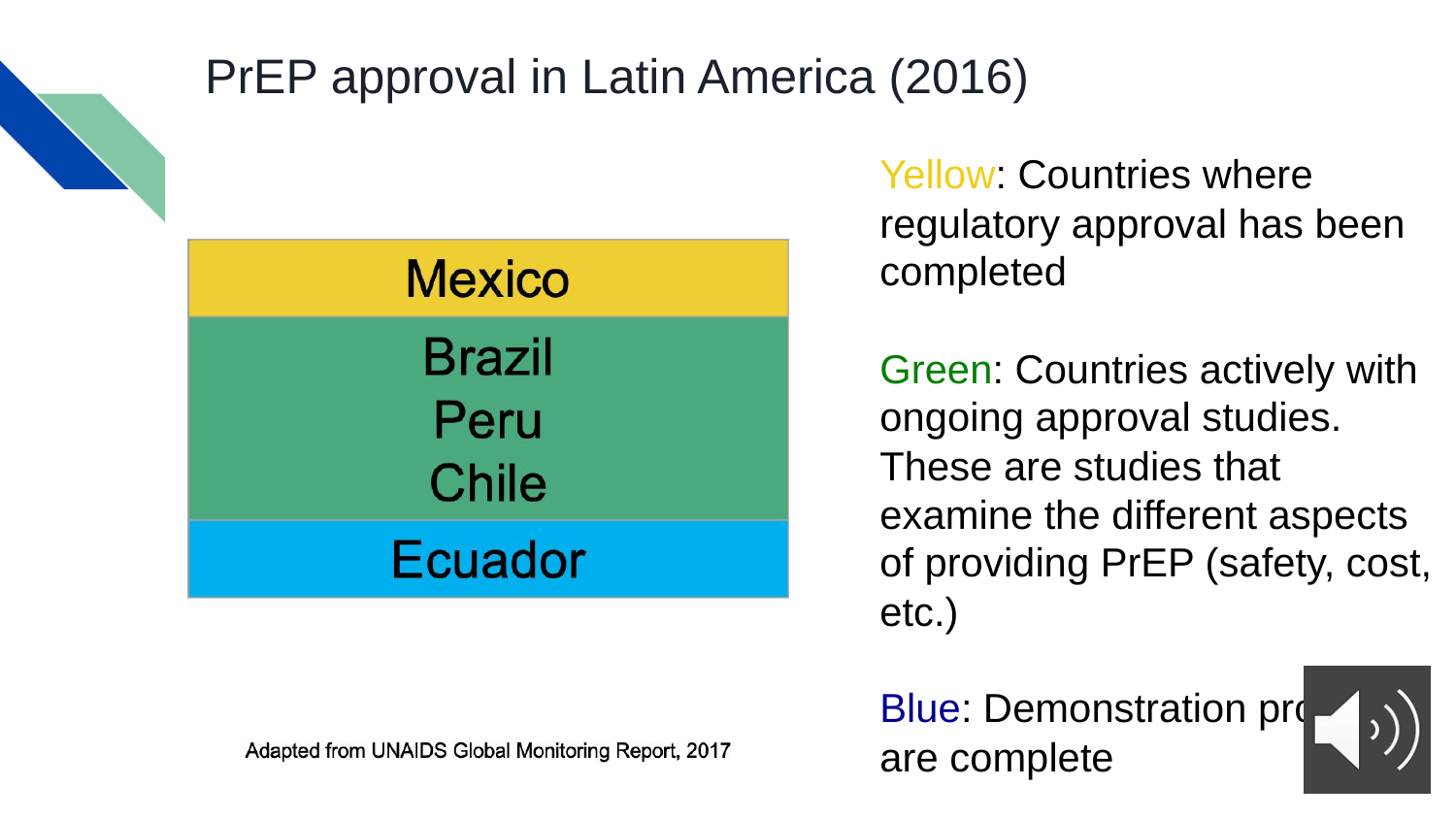

# PrEP approval in Latin America (2016)
Yellow: Countries where regulatory approval has been completed
Green: Countries actively with ongoing approval studies. These are studies that examine the different aspects of providing PrEP (safety, cost, etc.)
Blue: Demonstration projects are complete
40

## Slide 41
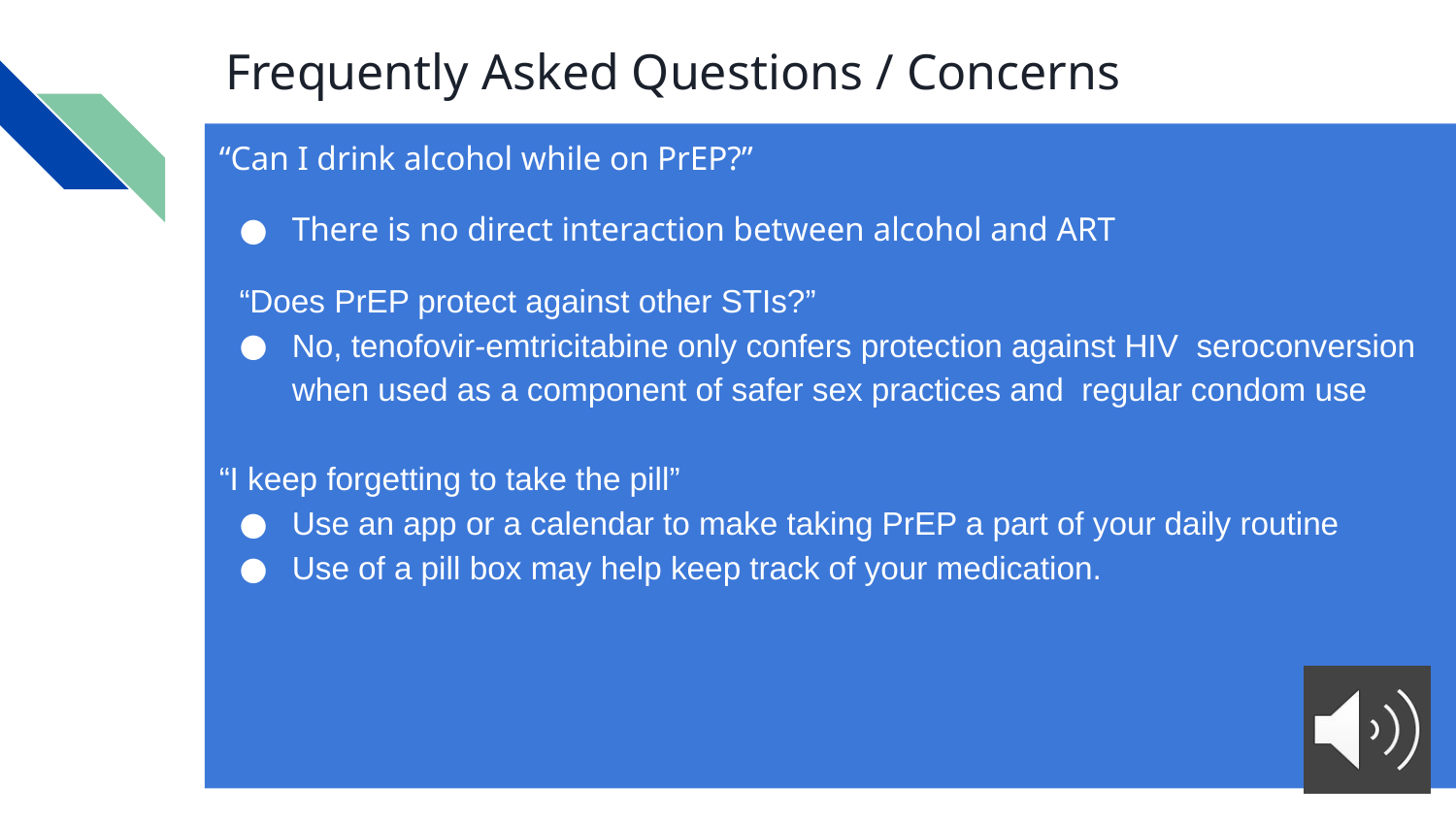

# Frequently Asked Questions / Concerns
“Can I drink alcohol while on PrEP?”
There is no direct interaction between alcohol and ART
“Does PrEP protect against other STIs?”
No, tenofovir-emtricitabine only confers protection against HIV seroconversion when used as a component of safer sex practices and regular condom use
“I keep forgetting to take the pill”
Use an app or a calendar to make taking PrEP a part of your daily routine
Use of a pill box may help keep track of your medication.
41

## Slide 42
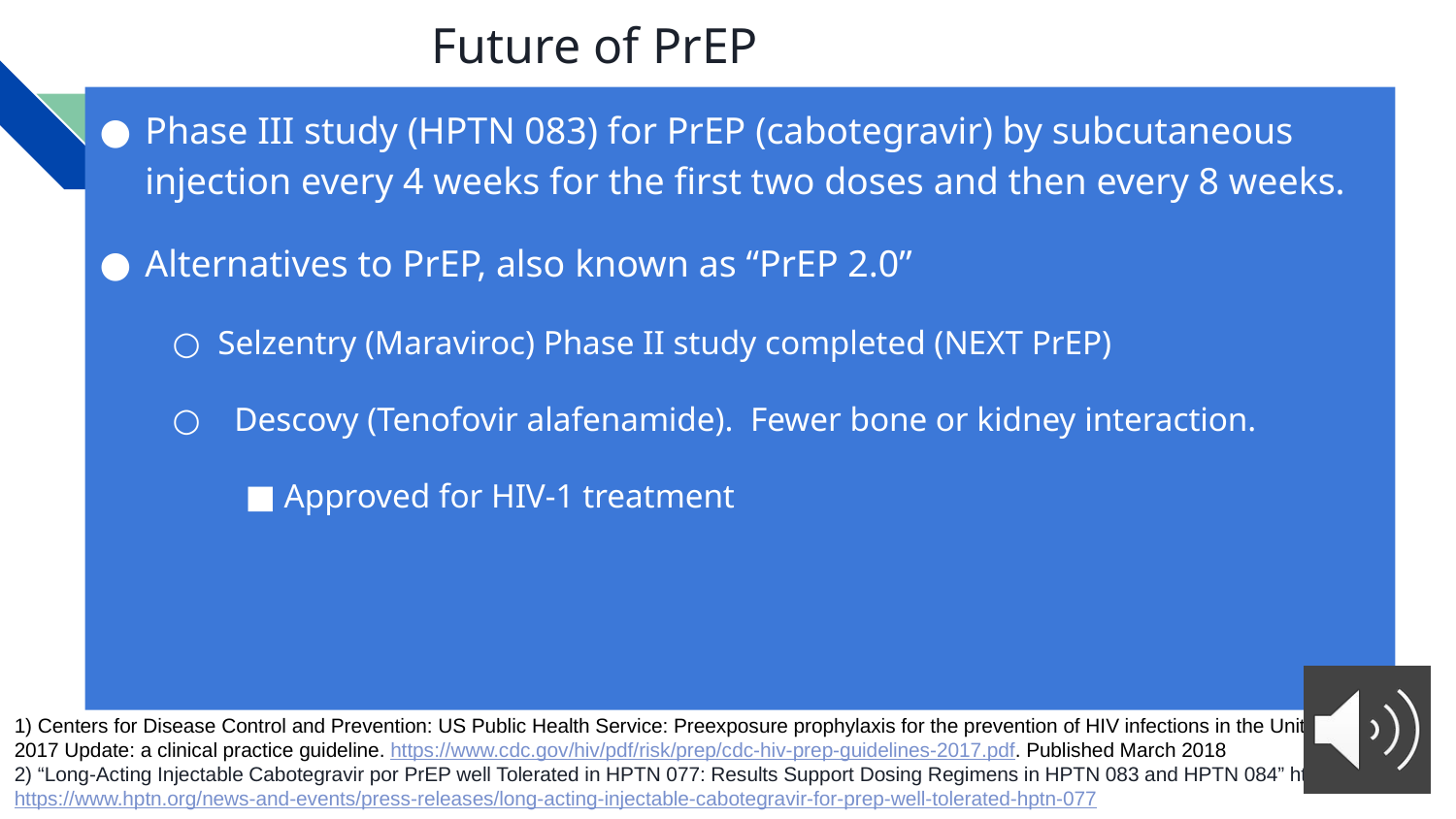

# Future of PrEP
Phase III study (HPTN 083) for PrEP (cabotegravir) by subcutaneous injection every 4 weeks for the first two doses and then every 8 weeks.
Alternatives to PrEP, also known as “PrEP 2.0”
Selzentry (Maraviroc) Phase II study completed (NEXT PrEP)
 Descovy (Tenofovir alafenamide). Fewer bone or kidney interaction.
 Approved for HIV-1 treatment
1) Centers for Disease Control and Prevention: US Public Health Service: Preexposure prophylaxis for the prevention of HIV infections in the United States – 2017 Update: a clinical practice guideline. https://www.cdc.gov/hiv/pdf/risk/prep/cdc-hiv-prep-guidelines-2017.pdf. Published March 2018
2) “Long-Acting Injectable Cabotegravir por PrEP well Tolerated in HPTN 077: Results Support Dosing Regimens in HPTN 083 and HPTN 084” https:https://www.hptn.org/news-and-events/press-releases/long-acting-injectable-cabotegravir-for-prep-well-tolerated-hptn-077
42

## Slide 43
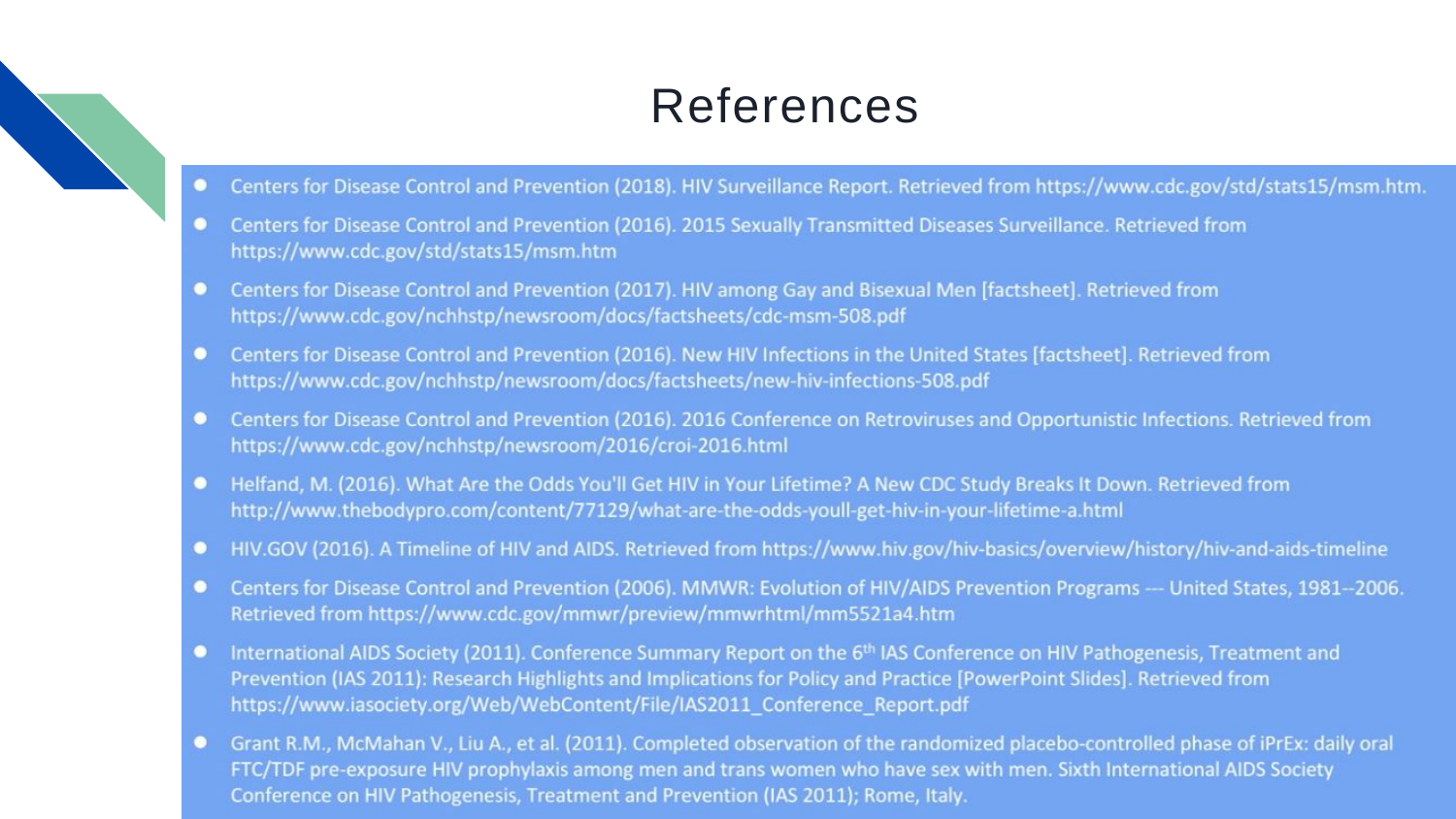

# References
43

## Slide 44
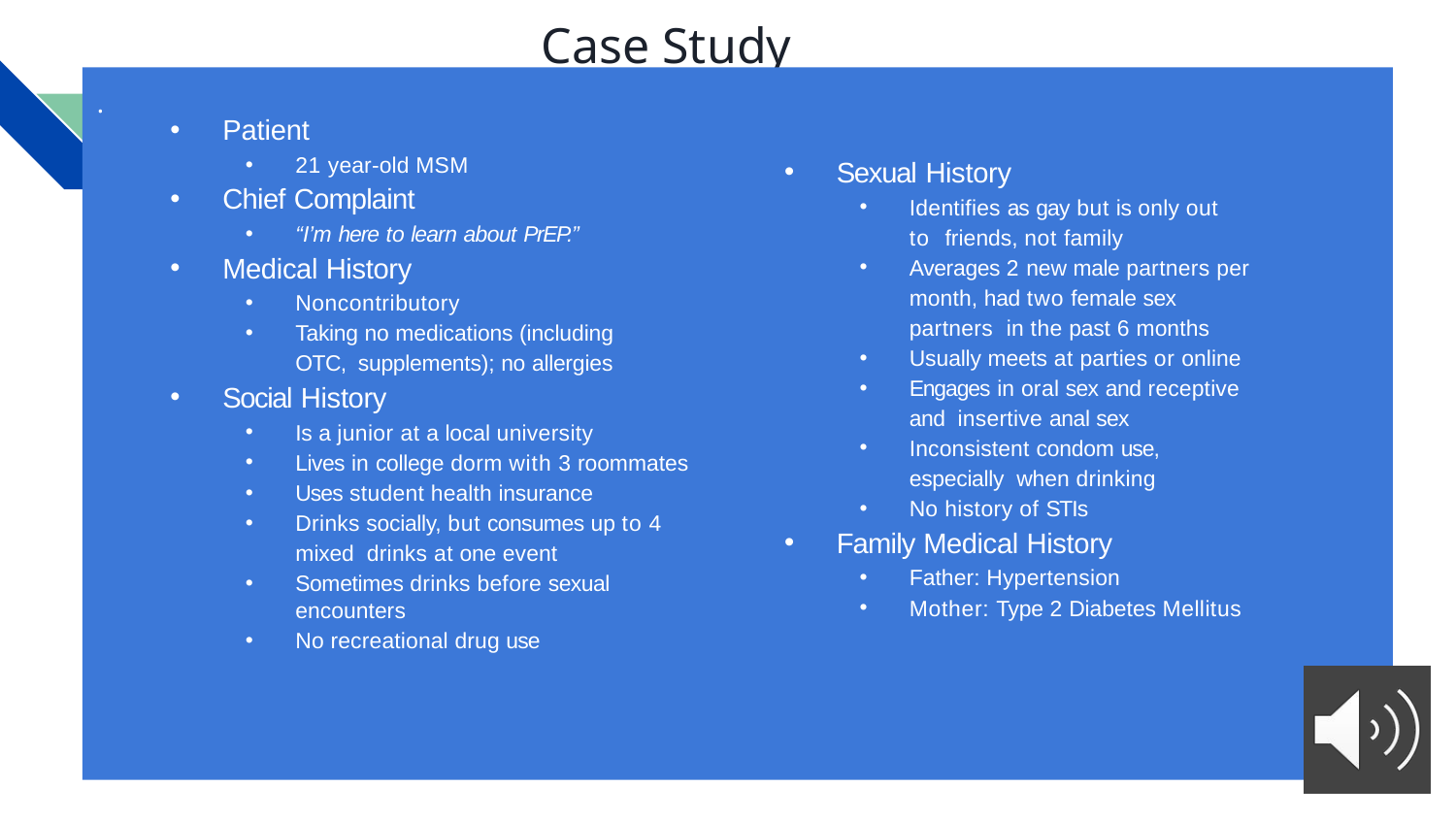

# Case Study
.
Patient
21 year-old MSM
Chief Complaint
“I’m here to learn about PrEP.”
Medical History
Noncontributory
Taking no medications (including OTC, supplements); no allergies
Social History
Is a junior at a local university
Lives in college dorm with 3 roommates
Uses student health insurance
Drinks socially, but consumes up to 4 mixed drinks at one event
Sometimes drinks before sexual encounters
No recreational drug use
Sexual History
Identifies as gay but is only out to friends, not family
Averages 2 new male partners per month, had two female sex partners in the past 6 months
Usually meets at parties or online
Engages in oral sex and receptive and insertive anal sex
Inconsistent condom use, especially when drinking
No history of STIs
Family Medical History
Father: Hypertension
Mother: Type 2 Diabetes Mellitus
44
44
