# Supplementary material for: HIV Pre-exposure Prophylaxis Education for Clinicians Caring for Spanish-Speaking Men Who Have Sex With Men (MSM)
Source: MedEdPORTAL. 2021 Mar 18;17:11110. doi: 10.15766/mep_2374-8265.11110 (PMC8015640; doi:10.15766/mep_2374-8265.11110)
Supplement: Supplementary file 1 — Spanish PPT Presentation.pptxEnglish PPT Presentation.pptxSpanish Audio-Guided PPT Video Presentation.pptxEnglish Audio-Guided PPT Video Presentation.pptxDiscussion Guide.docxPatient-Physician Video.mp4Spanish Transcript of Patient-Physician Video.docxEnglish Transcript of Patient-Physician Video.docxPreworkshop Evaluation Form.docxPostworkshop Evaluation Form.docx [file mep_2374-8265.11110-s001.zip › E. Discussion Guide.docx]

HIV Pre-Exposure Prophylaxis Education for Clinicians Caring for Spanish-Speaking Men Who Have Sex with Men (MSM)

**Facilitator Guide**

**Slide 1:**

Title Slide.

**Slide 2:**

Presenters should introduce themselves

For example: Hello everyone, thank you for joining us for today’s presentation for pre-exposure prophylaxis education for clinicians. My name is _________________ (credentials). Today we will be taking you through an overview of pre-exposure prophylaxis or PrEP, with a specific emphasis in the MSM population.

**Slide 3:**

Disclosure slide

**Slide 4:**

Give a brief overview of the presentation. This workshop has been developed to educate medical providers on how to identify, initiate, survey and maintain patients on PrEP with a focus on MSM communities.

This slide provides a quick overview of today’s topics. First we will go through a quick introduction of HIV and PrEP, including a history of HIV prevention. Next, we will examine the determination for eligibility, and initial testing in patients who may benefit from PrEP. We will then move on to discuss appropriate steps in maintenance, on monitoring of health of patients on PrEP. Then we will discuss potential scenarios in which a patient may discontinue PrEP, plus the appropriate steps that you, as the clinician, must take in order to safely discontinue medication. Lastly we will explore potential barriers to the use of PrEP, including barriers to uptake and adherence, as well as some resources that may help your patients access and pay for PrEP more easily.

**Slide 5:**

Review the learning objectives with the audience

**Slide 6:**

It is important to go over definition early on in the presentation. Here, we bring the audience up to date with accepted acronyms. It is important to emphasize HSH as an acronym with no distinction to sexual identity. We also present the difference between PrEP and PEP. Additionally, it introduced the CDC as the organization where guidelines for PrEP are based from for the purposes of this presentation.

**Slide 7:**

Slide 7 is an introduction slide.

**Slide 8:**

This slide highlight that although the global annual incidence of HIV infections of has decreased, it is not a fast enough decent to reach the 2020 target goal fewer than 500,000 new HIV infections globally. This is equivalent to a 75% reduction since 2010.

UNAIDS 2017, Global AIDS monitoring 2017

**Slide 9:**

In Latin America, the incidence of HIV infections has remained stable, while in the Caribbean it has only decreased by 5%. This highlights the need for intervention due to stagnant rates of incidence.

UNAIDS 2017, Global AIDS monitoring 2017

**Slide 10:**

In Latin American and the Caribbean, overall incidence rate changes are influence by individual country rates. It indicates an increase in incidence such as in Chile, Cuba, and Guatemala and a decrease in incidence such as in El Salvador, Trinidad & Tobago and Colombia.

UNAIDS 2017, Global AIDS monitoring 2017

**Slide 11:**

Despite public health initiatives targeting the MSM community, this group still remains a high incidence cohort for HIV infections. Additionally, we can observe from this slide that the prevalence of HIV in Latin American countries is concentrated in the MSM community. Therefore, continued clinical education is needed to reduce further HIV transmission in this high risk group.

UNAIDS 2017, Global AIDS monitoring 2017

**Slide 12:**

This next slide shows the disparities that continue to exist within the MSM community. We have been unable to close the disparity gap among racial and ethnic groups, most notably within the black and latino communities. A statistic that one may find particularly shocking is that the life-time risk of HIV diagnosis in Latino MSM is 1 in 4.

*“2016 CROI News Release | CDC.” Centers for Disease Control and Prevention, Centers for Disease Control and Prevention, www.cdc.gov/nchhstp/newsroom/2016/croi-2016.html.*

**Slide 13:**

This table highlights the rate of acquiring HIV by sexual encounter. We can see that among anal, penile-vaginal intercourse and oral intercourse, anal intercourse experiences the highest rate of HIV acquisition per 10,000 exposures. Of all, receptive anal intercourse has the highest rate of HIV acquisition per 10,000 exposures.

**Slide 14:**

Depicted here, is a brief timeline of HIV, as well as the result in prevention strategies. These strategies were implemented almost immediately, starting in 1981, when the first cases of immune deficiency were described. During the mid-1980’s, the regular use of condoms was promoted as a means of reducing HIV transmission. This was followed by needle exchange programs in the 1990’s as well as concerted grass-roots efforts from community leaders in minority communities to address HIV prevention. Since the 1980’s, efforts to develop vaccines against HIV have failed to produce an effective immunization strategy. It wasn’t until 2012, that the FDA approved an oral medication for HIV pre-exposure prophalaxys, thanks in large part to a multinational clinical trial known as IPREX. Finally, in 2019 USPSTF issues Grade A recommendation for PrEP & HIV testing.

It is important not to emphasize each point but to highlight some important prevention strategies as well as the introduction of IPREX clinical trial

**Slide 15:**

The IPREX trial, also known as Iniciativa prophalaxys pre-exposicion, was a randomized, double-blind, placebo controlled phase 3 clinical trial, of once daily oral truvada, also known as emtricitabine and tenofivir, for HIV prevention. It occurred between 2007 and 2011 in Brazil, Ecuador, Peru, South Africa, Thailand, and the EEUU. Participants included 2499 HIV-seronegative men or transgender women who have sex with men. 1251 were assigned to the truvada group. 1248 were assigned to the placebo group. Results included 131 participants who became HIV+, 48 in the truvada group and 83 in the placebo group. Overall, truvada reduced the rate of new infections by 42%* and by 92% in those with detectable drug levels, after a modified intention to treat analysis.

*Grant RM et al. Preexposure chemoprophylaxis for HIV prevention in men who have sex with men. New Engl Jour Med 363(27): 2587-2599, 2010*

**Slide 16:**

When looking more closely at risk reduction based on adherence to truvada, which was confirmed by detectable drug levels in patient blood samples, we can see that poor to little compliance with medication explains low risk reduction for certain participants. However, we see that in participants with moderate to excellent compliance, optimal HIV transmission risk reduction was achieved. Although it is not recommended that a patient miss doses, we can see that even missing a few doses can confer some protective ability. If your patient misses one dose periodically, you can reassure them they are still receiving a protective benefit from their PrEP regimen.

**Slide 17:**

Eligibility Determination

**Slide 18:**

Depicted here, is a summary of guidance for PrEP use for the MSM and HSW/M population. Patients who satisfy any of the following criteria may be eligible for PrEP. It should be noted that a high number of sex partners is loosely defined but has been described in some studies as around 5 or more in the past 12 months. However, each individual’s behaviors and practices should be addressed on a case by case basis. Another population who may benefit is injectable drug users, however, for the purposes of this presentation the focus will be on MSMs who do not inject drugs.

**Slide 19:**

Shown here, are recommended indications for MSMs. First being, a man or transgender woman weighing at least 35kg, without any acute or established HIV infection, who has had any male partners in the past 6 months, who is not in a monogamous relationship with a recently tested HIV negative man AND at least one of the following:

Engages in anal sex without condoms, either receptive or insertive, in the past 6 months

Has a history of bacterial STI, including syphilis, gonorrhea, or chlamydia, diagnosed or reported in the past 6 months

Or any patient in a serodiscordant relationship

A patient who always uses condoms or only engages in oral sex may also request to start on PrEP, given that there are no contraindications to initiating therapy. It is ok to prescribe to these patients giving proper counseling on the short and long-term side effects as well as the importance of adherence.

*Centers for Disease Control and Prevention: US Public Health Service: Preexposure prophylaxis for the prevention of HIV infections in the United States – 2017 Update: a clinical practice guideline.* [*https://www.cdc.gov/hiv/pdf/risk/prep/cdc-hiv-prep-guidelines-2017.pdf*](https://www.cdc.gov/hiv/pdf/risk/prep/cdc-hiv-prep-guidelines-2017.pdf)*. Published March 2018*

**Slide 20:**

When starting PrEP, it is important to ask the following screening questions to see if your patient is a candidate:

In the past 6 months:

With how many male partners have you engaged in anal sex, either receptive or insertive? If the answer is 1 or more times, the patient may be a candidate

How many times did you have receptive or insertive anal sex where one of you was not wearing a condom? If the answer is 1 or more times, the patient may be a candidate

Have you been diagnosed with an STI? If the patient answers yes, they may be a PrEP candidate.

Have you used alcohol or any illicit substances, such as methamphetamines, cocaine, heroine, etc, before a sexual encounter? If the patient answers yes, they may also be a PrEP candidate.

*Centers for Disease Control and Prevention: US Public Health Service: Preexposure prophylaxis for the prevention of HIV infections in the United States – 2017 Update: a clinical practice guideline.* [*https://www.cdc.gov/hiv/pdf/risk/prep/cdc-hiv-prep-guidelines-2017.pdf*](https://www.cdc.gov/hiv/pdf/risk/prep/cdc-hiv-prep-guidelines-2017.pdf)*. Published March 2018*

**Slide 21:**

Who is not eligible for PrEP, an HIV positive patient, or one who is acutely infected with HIV. A patient with renal disease which includes an estimated creatinine clearance rate of less than 60ml/min, a patient, who for any reason, is on high doses of NSAIDs, or a patient who is on any nephrotoxic agents, including: acyclovir, valacyclovir, cidofovir, ganciclovir, valganciclovir, and aminoglycosides. Or a patient with a history of pathologic or fragility fractures OR who has significant risk factors for osteoporosis. Of note, Hepatitis B infection is not a contraindication to PrEP use, but PrEP adherence is critical for these patients. It is important that these patients are also co-managed with an infectious disease or hepatic disease specialist.

*Centers for Disease Control and Prevention: US Public Health Service: Preexposure prophylaxis for the prevention of HIV infections in the United States – 2017 Update: a clinical practice guideline.* [*https://www.cdc.gov/hiv/pdf/risk/prep/cdc-hiv-prep-guidelines-2017.pdf*](https://www.cdc.gov/hiv/pdf/risk/prep/cdc-hiv-prep-guidelines-2017.pdf)*. Published March 2018*

**Slide 22:**

What is the difference between PrEP and PEP. PEP is post exposure prophalaxys, it is a treatment to reduce risk of HIV transmission. PEP is tenofovir DF or TDF with emtricitabine and raltegravir or dolutegravir. PEP is used if an individual is HIV-negative or does not know his HIV status and in the last 72 hours:

May have been exposed to HIV during sex

Shared needles and paraphernalia to prepare injectable drugs

Or was sexually assaulted

*Centers for Disease Control and Prevention: US Public Health Service: Preexposure prophylaxis for the prevention of HIV infections in the United States – 2017 Update: a clinical practice guideline.* [*https://www.cdc.gov/hiv/pdf/risk/prep/cdc-hiv-prep-guidelines-2017.pdf*](https://www.cdc.gov/hiv/pdf/risk/prep/cdc-hiv-prep-guidelines-2017.pdf)*. Published March 2018*

**Slide 23:**

How long does it take for PrEP to work. According to the CDC, maximum protection is achieved in rectal tissue in 5 to 7 days and in vaginal tissue in about 20 days. Penile tissue has not been studied.

*Centers for Disease Control and Prevention: US Public Health Service: Preexposure prophylaxis for the prevention of HIV infections in the United States – 2017 Update: a clinical practice guideline.* [*https://www.cdc.gov/hiv/pdf/risk/prep/cdc-hiv-prep-guidelines-2017.pdf*](https://www.cdc.gov/hiv/pdf/risk/prep/cdc-hiv-prep-guidelines-2017.pdf)*. Published March 2018*

*WHO: Consolidates Guidelines on the Use of Antiretroviral Drugs for Treating and Preventing Infection: Recommendations for a Public Health Approach, Second Edition, 2016*

**Slide 24:**

**Initial Testing and vaccinations for PrEP**

HIV testing: HIV testing includes a documented negative anti-body test within the week before initiating, ideally an anti-body antigen test. Blood, serum or rapid point of care fingerstick is acceptable. Oral fluid tests are not sensitive enough. Viral RNA testing to test for acute HIV infection. If the patient tests preliminarily positive on the HIV antibody test, this must be confirmed by a local laboratory standardized practice, which may include viral load and CD4 count.

**Slide 25:**

STI testing, most notably for gonorrhea and chlamydia, will include a nucleic acid amplification test in 3 sites, pharyngeal, rectal and urine specimens.

Test for Syphilis: Prueba de sangre

Liver and kidney testing includes Hepatitis B virus serology, and as noted earlier, hepatitis B vaccination is not a contraindication for PrEP use. Hepatitis B diagnosis does not exclude from PrEP use either. Adherence, again, is more important for these patients. As for kidney function, an estimated creatinine clearance of greater than 60ml/min is ideal.

**Slide 26:**

Liver and kidney testing includes Hepatitis B virus serologies, and as noted earlier, hepatitis B vaccination is not a contraindication for PrEP use. Hepatitis B diagnosis does not exclude from PrEP use either. There is risk of reactivation of the Hepatitis B virus if PrEP is discontinued. Adherence, again, is more important for these patients. As for kidney function, an estimated creatinine clearance of greater than 60ml/min is ideal.

*Centers for Disease Control and Prevention: US Public Health Service: Preexposure prophylaxis for the prevention of HIV infections in the United States – 2017 Update: a clinical practice guideline.* [*https://www.cdc.gov/hiv/pdf/risk/prep/cdc-hiv-prep-guidelines-2017.pdf*](https://www.cdc.gov/hiv/pdf/risk/prep/cdc-hiv-prep-guidelines-2017.pdf)*. Published March 2018*

**Slide 27:**

Vaccine Recommendations:

HPV: vaccinate if your patient is under 46 years old

For hepatitis B, vaccinate if they are susceptible

For hepatitis A, vaccinate if your patient had never been vaccinated before

Other vaccines include, influenza, meningococcus depending on geography and pneumococcus if indicated

*Centers for Disease Control and Prevention: US Public Health Service: Preexposure prophylaxis for the prevention of HIV infections in the United States – 2017 Update: a clinical practice guideline.* [*https://www.cdc.gov/hiv/pdf/risk/prep/cdc-hiv-prep-guidelines-2017.pdf*](https://www.cdc.gov/hiv/pdf/risk/prep/cdc-hiv-prep-guidelines-2017.pdf)*. Published March 2018*

**Slide 28:**

How is PrEP prescribed. Truvada is prescribed as one oral pill daily with a 90 day supply during onboarding. Patients must return at 3 months for their follow-up visit. The following information is given from Gilead: The dosage of TRUVADA in HIV-1 uninfected adults and adolescents weighing at least 35kg is one tablet, containing 200mg of Tenofivir and 300mg of emtricitibine, taken once daily orally with or without food.

<https://www.gilead.com/~/media/Files/pdfs/medicines/hiv/truvada/truvada_pi.PDF>

**Slide 29:**

Adverse reactions

**Slide 30:**

PrEP Side effects and adverse reactions. Shown here are selected adverse reactions from the IPREX study as well as the statistical significance of each. The headaches, nausea and diarrhea usually resolve within the first 2 weeks and you should encourage your patient to continue with the medicine. Again if creatinine is above 60, they can still continue on with the drug. Nausea and unintentional weight loss were the only 2 adverse events that were found to be statistically significant.

**Slide 31:**

Maintenance

**Slide 32:**

Follow up testing for PrEP. Follow up testing at every 3 months should include an HIV test, medication adherence counseling, behavioral risk reduction support, side effect assessment, STI symptom assessment and test for bacterial STIs. At 3 months and every 6 months thereafter, you should also test for renal function. More frequent monitoring is recommended if comorbidities such as hypertension or diabetes mellitus are present. As a reminder, a rise in creatinine is not a reason to withhold if the estimated creatinine clearance rate remains above 60ml/min

**Slide 33:**

Every 12 months, you should reassess whether or not your patient should continue on PrEP. It is unclear if HIV uninfected are as at risk as HIV infected persons treated with the combination ART. DEXA is not recommended for those patients on PrEP, unless there is a history of pathologic or fragility fractures OR the patient also has other risk factors for osteoporosis.

*Centers for Disease Control and Prevention: US Public Health Service: Preexposure prophylaxis for the prevention of HIV infections in the United States – 2017 Update: a clinical practice guideline.* [*https://www.cdc.gov/hiv/pdf/risk/prep/cdc-hiv-prep-guidelines-2017.pdf*](https://www.cdc.gov/hiv/pdf/risk/prep/cdc-hiv-prep-guidelines-2017.pdf)*. Published March 2018*

**Slide 34:**

Discontinuation of PrEP

**Slide 35:**

When and how to discontinue PrEP. Possible scenarios that your patient may offer you include, starting a monogamous relationship with an HIV-negative partner, a drug holiday during which the patient no longer wishes to be on medication, or any other situation to discuss with clinician on a case by case basis. In any case, it is important that you use the CDC’s HIV risk reduction tool to help your patient understand their risk. Safely discontinuing PrEP should be discussed with the patient on a case by case basis. This also includes using alternative methods of reducing HIV seroconversion. At the time, clinicians should document the HIV status, the reason for PrEP discontinuation, and also the patients recent medication adherence and reported sexual risk behavior.

*Centers for Disease Control and Prevention: US Public Health Service: Preexposure prophylaxis for the prevention of HIV infections in the United States – 2017 Update: a clinical practice guideline.* [*https://www.cdc.gov/hiv/pdf/risk/prep/cdc-hiv-prep-guidelines-2017.pdf*](https://www.cdc.gov/hiv/pdf/risk/prep/cdc-hiv-prep-guidelines-2017.pdf)*. Published March 2018*

**Slide 36:**

Show here is a screen shot of the HIV’s risk reduction tool. You can customize your patient including their HIV status as well as their sexual activity. If you’d like more information, please refer to the CDCs HIV risk reduction tool.

*“HIV Risk Reduction Tool | CDC.” Centers for Disease Control and Prevention, Centers for Disease Control and Prevention, wwwn.cdc.gov/hivrisk/.*

**Slide 37:**

Barriers to Use

**Slide 38:**

Barriers to uptake and adherence to PrEP. Uptake and adherence to PrEP is often confounded by many barriers. The first being accessing medical care.

Access, especially in underserved communities, poor access to insurance and to culturally competent clinicians, is often the first barrier to care.

Provider comfort level. It is important that you as a provider, be well versed not only in the indications, mechanisms and side effects of PrEP, but also in how to talk to your patient and to provide them a safe and non-judgmental atmosphere.

Social Stigma. The social stigma against the LGBTQ individuals still exists. Embarrassment sometimes contributes to patients not seeking care.

Convenience of follow up testing. Multiple rounds of follow up testing are needed for PrEP maintenance. This barrier is important for patients who work or who need child care services.

Insurance coverage. Varying levels of coverage exist, so it is important that you talk to your care coordination and social work teams to figure out what is best for your patient and how to cover gaps that may be left if not all of cost is covered by insurance.

Overall financial burden. This can be comprised of other financial barriers, which include, childcare services, missed time from work, transportation issues, parking, etc.

<https://southernaids.files.wordpress.com/2016/03/we-have-the-tools-to-end-hiv-benefits-barriers-and-solutions-to-expanded-utilization-of-pre-exposure-prophylaxis-prep-in-the-us-deep-south.pdf>

**Slide 39:**

The Homophobic Climate Index (HCI) published by UNAIDS in 2017 is a sociological construct that considers institutional, social and behavioral homophobia. It also takes into consideration laws against LGBT people and the level of tolerance. The dark green color indicates less tolerant countries and clearing of color to light green, blue, yellow and red indicate more tolerant countries.

*UNAIDS 2017, Global AIDS monitoring 2017*

**Slide 40:**

Self-explanatory, approval of PrEP in Latin America

**Slide 41:**

Frequently asked questions or concerns.

Can I drink alcohol while on PrEP?

While there is no direct interaction between and antiretroviral drugs, there has been a study that reported an association with alcohol interaction beliefs and adherence to PrEP.

Does PrEP protect against other STIs?

Truvada only confers protection against HIV seroconversion when used as a component of safer sex practices and regular condom use.

“I keep forgetting to take my medication”

It may be useful to use an app or a calendar to make taking PrEP a part of a patient’s daily routine. It may also be advisable to use a pill box to help keep track of medication.

*Kalichman SC and Eaton L Journal of the International AIDS Soceity 2017,* ***20****:21534. http:www.jiasoceity.org/index.php.jias/article/view/21534 doi:10.7448/IAS.20.1.21534*

**Slide 42:**

Self-explanatory

**Slide 43:**

References

**Slide 44:**

We will now show you a scripted clinical encounter for best practices in PrEP prescribing in Spanish. The patient here is a 21-year-old male; whose chief complaint is “estoy aqui para aprender sobre PrEP.” His Medical history is non-contributory. He takes no medications, including no over-the-counter medications and no supplements. He has no allergies.

The social history includes, education as a junior at a local university, who lives in a college dorm with 3 roommates. He uses student health insurance. He drinks socially, but consumes up to 4 mixed drinks at one event. Sometimes drinks before sexual encounters. He does not engage in recreational drug use.

The sexual history includes, identification as gay, but is only out to friends, not family. Averages 2 new male partners per month but has had 2 female sex partners in the past 6 months. Usually meets his partners at parties or online. He engages in oral sex, as well as insertive and receptive anal sex. His condom use is inconsistent, especially when drinking. He has no history of STIs.

His family medical history includes, a father with hypertension and a mother with type II diabetes mellitus.

During this portion of the presentation, the facilitator can select from the following options:

1. Real time demonstration: The facilitator acts out the described clinical encounter with a volunteer. The facilitator can play the role of the provider and demonstrate appropriate clinical communication skills used in assessing risk factors for HIV acquisition and PrEP candidacy. A co-facilitator or participant volunteer can play the role of the patient. Upon completion of the demonstration, the facilitator should ask for audience feedback on recommendations for communication strategies used in assessing risk factors for HIV acquisition and PrEP candidacy. It is important to share with participants that communication skills can vary from clinician to clinician, and it is important to listen to and receive feedback from their professional colleagues and patients.
2. Play videotaped clinical encounter: The facilitator plays the videotaped clinical encounter for the audience (see Appendix D). After watching the video, the facilitator should ask for audience feedback on recommendations for communication strategies used in assessing risk factors for HIV acquisition and PrEP candidacy. They should comment on what they liked or did not like about the communication skills used, and reflect on how the skills demonstrated influence their own clinical practices. It is also important to share with participants that communication skills can vary from clinician to clinician, and it is important to listen to and receive feedback from their professional colleagues and patients. *For example, during the videotape, the provider asks the patient if he is in any romantic relationships (situacion romantica). It is important to point out to the learners that sexual encounters may or may not be secondary to romantic relationships and some patients may not disclose sexual encounters if they are limited to romantic relationships.*

*Other discussion topics:*

- *Motivational interviewing discussing alcohol or drug use and recommendations for using alcohol responsibly and restricting drug use.*
  - *Assess current alcohol use and discuss strategies to mitigate alcohol abuse. Counsel that they should abstain from alcohol prior to sexual encounters to reduce the risk of high-risk sexual activity.*
  - *Assess current drug use and discuss strategies to abstain from drug use prior to sexual encounters.*
- *Motivational interviewing for patients reluctant to start PrEP or hesitant to continue taking it.*
  - *Discuss their reasons for resistance to taking PrEP and address their concerns based on current evidence*
  - *Assess and discuss their current risk-taking behaviors and ways to address them*
  - *If they would benefit from PrEP, advise them that you and the support staff are an available resource for questions and concerns while they take PrEP.*
- *How to discuss discontinuing PrEP when clinically appropriate.*
  - *Inquire about and document the reasons for their interest in discontinuation.*
  - *Ask when was the last time they had a sexual encounter?*
  - *Ask if they are in a monogamous relationship with an HIV-negative partner?*
  - *Document the last time they used PrEP.*
  - *Document their last HIV test*
- *Cultural stigma against Hispanic MSM*
  - *Men who have sex with men traditionally did not identify as gay unless they lived in cities where they were exposed to the North American-European “gay identity.” ^10^*
  - *The literature describes a dichotomy between more “feminine” roles attached with receptive sex while the more “hetero” partner would take the active role. This played into a defined sexual “script” with attached meanings to penetrative and receptive sexual roles, manliness, and sexually.^11^*
  - *In Latin American communities, homosexuality can be deemed “a weakness” or cause for “embarrassment to the family.”^12^*
  - *Older Latinos are predominantly Catholic and their condemnation of homosexuality can suppress expression of sexuality and safer sex practices. ^12^*

**References**

1. UNAIDS 2017, Global AIDS monitoring 2017. Indicatorregistry.unaids.org. https://indicatorregistry.unaids.org/sites/default/files/2017-global-aids-monitoring_en.pdf. Published 2020. Accessed October 16, 2020.

2. 2016 CROI News Release | CDC. Cdc.gov. https://www.cdc.gov/nchhstp/newsroom/2016/croi-2016.html. Published 2020. Accessed October 16, 2020.

3. Grant RM, Lama JR, Anderson PL, et al. Preexposure chemoprophylaxis for HIV prevention in men who have sex with men. *N Engl J Med*. 2010;363(27):2587-2599. doi:10.1056/NEJMoa1011205

4. Centers for Disease Control and Prevention: US Public Health Service: Preexposure prophylaxis for the prevention of HIV infections in the United States – 2017 Update: a clinical practice guideline. . <https://www.cdc.gov/hiv/pdf/risk/prep/cdc-hiv-prep-guidelines-2017.pdf>. Published March 2018. Accessed October 16, 2020.

5. World Health Organization: Consolidated Guidelines on the Use of Antiretroviral Drugs for Treating and Preventing Infection: Recommendations for a Public Health Approach, Second Edition. Published 2016.

6. Gilead.com. https://www.gilead.com/~/media/Files/pdfs/medicines/hiv/truvada/truvada_pi.PDF. Published 2020. Accessed October 16, 2020.

7. HIV Risk Reduction Tool | CDC. HIV Risk Reduction Tool. https://hivrisk.cdc.gov/. Published 2020. Accessed October 16, 2020.

8. Ervin J, McAllaster C. WE HAVE THE TOOLS TO END HIV: Benefits, Barriers, and Solutions to Expanded Utilization of Pre-exposure Prophylaxis (PrEP) in the US Deep South. Southernaids.files.wordpress.com. https://southernaids.files.wordpress.com/2016/03/we-have-the-tools-to-end-hiv-benefits-barriers-and-solutions-to-expanded-utilization-of-pre-exposure-prophylaxis-prep-in-the-us-deep-south.pdf. Published 2020. Accessed October 16, 2020.

9. Kalichman S, Eaton L. Alcohol-antiretroviral interactive toxicity beliefs as a potential barrier to HIV pre-exposure prophylaxis among men who have sex with men. *J Int AIDS Soc*. 2017;20(1):21534. doi:10.7448/ias.20.1.21534

10. Evens E, Lanham M, Santi K, Cooke J, Ridgeway K, Morales G, Parker C, Brennan C, de Bruin M, Desrosiers PC, Diaz X, Drago M, McLean R, Mendizabal M, Davis D, Hershow RB, Dayton R. Experiences of gender-based violence among female sex workers, men who have sex with men, and transgender women in Latin America and the Caribbean: a qualitative study to inform HIV programming. BMC Int Health Hum Rights. 2019 Mar 5;19(1):9. doi: 10.1186/s12914-019-0187-5. PMID: 30832664; PMCID: PMC6399914.

11. Cáceres CF. HIV among gay and other men who have sex with men in Latin America and the Caribbean: a hidden epidemic? AIDS. 2002 Dec;16 Suppl 3:S23-33. doi: 10.1097/00002030-200212003-00005. PMID: 12685922.

12. Brooks RA, Etzel MA, Hinojos E, Henry CL, Perez M. Preventing HIV among Latino and African American gay and bisexual men in a context of HIV-related stigma, discrimination, and homophobia: perspectives of providers. *AIDS Patient Care STDS*. 2005;19(11):737-744. doi:10.1089/apc.2005.19.737
