# Supplementary material for: HIV Pre-exposure Prophylaxis Education for Clinicians Caring for Spanish-Speaking Men Who Have Sex With Men (MSM)
Source: MedEdPORTAL. 2021 Mar 18;17:11110. doi: 10.15766/mep_2374-8265.11110 (PMC8015640; doi:10.15766/mep_2374-8265.11110)
Supplement: Supplementary file 1 — Spanish PPT Presentation.pptxEnglish PPT Presentation.pptxSpanish Audio-Guided PPT Video Presentation.pptxEnglish Audio-Guided PPT Video Presentation.pptxDiscussion Guide.docxPatient-Physician Video.mp4Spanish Transcript of Patient-Physician Video.docxEnglish Transcript of Patient-Physician Video.docxPreworkshop Evaluation Form.docxPostworkshop Evaluation Form.docx [file mep_2374-8265.11110-s001.zip › G. Spanish Transcript of Patient-Physician Video.docx]

**SP Encounter Script (Spanish)**

SP: (knock) Adelante

Doctor: Hola Carlos, sí, yo soy la doctora Rosado, voy a hablar contigo en el día de hoy. ¿Cuéntame, que te trae por aquí?

SP: Bueno Doctora, soy estudiante Universitario estaba caminando por el campus y vi que tenían un kiosko, para, promoviendo la salud para los jóvenes, y me acerque y me hicieron un par de preguntas, y me dijeron que basado en las contestaciones que yo di, que yo, me recomendaron que me hiciera una evaluación formal con un doctor. So por eso estoy aquí

Doctor: No, pues, me alegra mucho que hallas venido. ¿Antes de todo, me gustaría conocer un poco más de ti, mencionas que eres estudiante de la Universidad?

SP: Sí, estoy en mi tercer ano.

Doctor: Okay, qué Bueno. ¿Con quién vives?

SP: Pues, estoy en casa, estoy con mi mama y con papi y con mi hermana menor.

Doctor: ¿Okay, eh, y en cuanto a tu historial medico pasado, tienes alguna… padeces de alguna enfermedad?

SP: No

Doctor: ¿Okay, haz tenido algún procedimiento quirúrgico?

SP: Bueno, me sacaron las amígdalas cuando tenia 8 anos, pero no no, nada mas eso.

Doctor: ¿Y te tomas algún medicamento o suplemento?

SP:No

Doctor: ¿Okay, tienes alergia a algún medicamento o algo?

SP: No

Doctor: ¿Okay, entonces, mencionaste tu familia, mama y papa, y como están de salud?

SP: Pues, están bastante bien. Papi tiene sus lípidos altos y mami tiene diabetes tipo dos, y artritis.

Doctor: Okay, eh…

SP: Mi hermana esta bien de salud también.

Doctor: Que bueno, entonces, te voy a hacer algunas preguntitas que son un poquito mas personales…

SP: Okay

Doctor: … pero te recuerdo que todo aquí es confidencial, excepto en casos de abuso sexual o enfermedades de transmisión sexual que tendría que reportarlas por ley. ¿Okay? ¿Tienes hijos?

SP: No

Doctor: ¿Y cual es tu situación romántica ahora?

SP: Pues, ahora no estoy saliendo con nadie formalmente, la ultima relación formal que tuve fue en Noviembre y nos dejamos.

Doctor: ¿Y desde ese entonces, haz estado con alguien?

SP: Pues e estado con varias gentes, estoy como en una época extraña, estoy explorando mi sexualidad, y sí e estado con varias personas desde Noviembre.

Doctor: ¿Como cuántas personas tu dirías que as estado en estos últimos 6 meses?

SP: Déjame ver, Bueno, yo creo que han sido como 12 en total. Y como 8 han sido hombres.

Doctor: ¿Okay, y como te identificas? ¿Te identificas como gay, bisexual…?

SP: Bueno, yo creo que probablemente soy gay, pero todavía no estoy cómodo en decirlo en la calle. Digamos…

Doctor: ¿Okay, y como tu describirías esas relaciones sexuales que haz tenido? ¿Han sido sexo oral, sexo anal, vaginal?

SP: En realidad, es como la canción, Doctora, de todo un poco.

Doctor: Okay, entiendo, entiendo. Eh, en cuanto a el uso de condones, usas condones consistentemente?

SP: Yo trato que sí, pero le voy a ser sincero, que cuando estoy especialmente saliendo, bebiendo, compartiendo con mis amigos, se me pasa.

Doctor: Okay

SP: Pero, sí, no es todo el tiempo, pero aveces pasa.

Doctor: ¿Y eh, pues, mencionaste el alcohol, alguna otra substancia como cocaine, heroína, crystal meth, marijuana?

SP: Marijuana, de vez en cuando, pero eso es lo único.

Doctor: ¿Okay, este, y alguna vez as sido diagnosticado con alguna enfermedad de transmisión sexual?

SP: Pues, yo no, pero eso es un, otra razón por la cual yo estoy aquí. Uno de mis parejas me contó después, pues, que sí salió positive para gonorrhea y eso me preocupa.

Doctor: Okay, pues, me alegra mucho que estes aquí buscando información, de hecho, basado en las contestaciones que me haz dado, el día de hoy, y pues las que te mandaron aquí, yo entiendo que si, que estas a alto riesgo, a contraer enfermedades de transmisión sexual. Okay, so lo primero que vamos a hacer en el día de hoy, ya que sabemos que estuviste expuesto a la gonorrhea, pues vamos a hacer unas pruebitas de gonorrhea en el día de hoy.

SP: Perfecto

Doctor: Ademas de esas pruebitas, también vamos a hacer unas pruebitas de el VIH, sífilis, este pues y otras enfermedades de transmisión sexual. Aveces ellas vienen juntas.

SP: Sí, sí, sí, haz todo lo que usted piensa es necesario.

Doctor: Okay, muy bien. Ademas de eso, te quisiera dar consejos, aconsejarte en cuanto el consume del alcohol, que de verdad debes limitarlo, pues, aveces cuando uno consume otras substancias, pues, hace que a uno se le olvide el ser cuidadoso y tener practicas sexuales que sean, pues, cuidadosas. Así que, eh, te aconsejaría que limitaras eso. También te aconsejo que uses condones consistentemente.

SP: Si, si.

Doctor: Y, pues, aprovecho la ocasión para también hablarte de un medicamento que tenemos que, eh, se llama la PrEP.

SP: Okay.

Doctor: El nombre también es Truvada. Y básicamente es una droga que es para pre-exposicion de VIH.

SP: OH

Doctor: Y esta droga lo que hace es que, eh, baja significativamente la, el riesgo de contraer el VIH en gente que ya son identificadas como negativas.

SP: ¿Cómo gente como yo?

Doctor: Exactamente.

SP: Okay, Y…

Doctor: Te gustaría saber un poquito mas de esto?

SP: Yo creo que sí, pues, como ejemplo, usted dijo que me la tomo todos los días?

Doctor: Si, tendría que ser algo que te tomas todos los días, el medicamento.

SP: ¿Y, cuales son los efectos secundarios?

Doctor: Pues, los efectos secundarios mas reportados an sido dolores de cabeza, nausea y diarrhea. Estos son unos síntomas que se han reportado, casi siempre, las primeras dos semanas—una o dos semanas y usualmente después se mejoran con el tiempo.

SP: ¿Okay, y lo puedo beber con alcohol? Que yo se que hay algunos medicamentos que no se pueden mezclar

Doctor: Pues, la verdad es que no interacciones con el alcohol, pero, como te mencioné anteriormente, pues, te aconsejo que limites el uso del alcohol por lo menos a dos bebidas en una ocasión.

SP: ¿Y cuándo uno… lo puedo tomar en cualquier hora del día? ¿Tiene que… me lo tengo que tomar con las comidas? ¿Cómo que hay alguna, eh, alguna indicación formal?

Doctor: Pues, fijate, no, no, no. Eh, no es algo que tengas que tomarte con comida, puedes tomártelo con o sin comida. Si decides tomártelo en la mañana o por las noches, lo importante es que busques la manera de recordarte tomártelo diariamente.

SP: Okay, okay. ¿Y también, me protege para el VIH usted me dijo?

Doctor: Si, te protege para el VIH pero no te protege contra otras enfermedades. Así que es bien importante usarlo en conjunto con condones para, pues, disminuir el riesgo de otras enfermedades.

SP: Okay, okay.

Doctor: Okay.

SP: Y ademas de las pruebitas de gonorrhea y esas cosas, hay otra cosa mas que tenemos que hacer para empezar el medicamento?

Doctor: Definitivamente, antes de comenzarte en el medicamento tendría que hacerte unas pruebitas de tu función renal para asegurarnos que esta bien antes de poder comenzarlo. También, eh, tendría que verificar tu estatus de, contra… hacerte pruebitas de hepatitis.

SP: Okay, pues yo creo que me parece bien, Doctora.

Doctor: Ah pues bien.

SP: Estoy interesado.

Doctor: Que bueno, me alegra de que estes interesado, lo que vamos a hacer ahora es que te voy a pasar por el area de la enfermería para que te saquen la, la sangre—tenemos unas pruebitas de sangre y orina. Este, y entonces te voy a dar unas recetas de 90 días.

SP: Ohh

Doctor: ¿Y me gustaría verte en 3 meses para el seguimiento, okay?

SP: Esta bien.

Doctor: Bueno, cuídate mucho, un placer.

SP: ¡Gracias, Doctora!
