# Supplementary material for: HIV Pre-exposure Prophylaxis Education for Clinicians Caring for Spanish-Speaking Men Who Have Sex With Men (MSM)
Source: MedEdPORTAL. 2021 Mar 18;17:11110. doi: 10.15766/mep_2374-8265.11110 (PMC8015640; doi:10.15766/mep_2374-8265.11110)
Supplement: Supplementary file 1 — Spanish PPT Presentation.pptxEnglish PPT Presentation.pptxSpanish Audio-Guided PPT Video Presentation.pptxEnglish Audio-Guided PPT Video Presentation.pptxDiscussion Guide.docxPatient-Physician Video.mp4Spanish Transcript of Patient-Physician Video.docxEnglish Transcript of Patient-Physician Video.docxPreworkshop Evaluation Form.docxPostworkshop Evaluation Form.docx [file mep_2374-8265.11110-s001.zip › H. English Transcript of Patient-Physician Video.docx]

**SP Encounter Script (English Translation)**

SP: (knock): Please come in

Doctor: Hello Carlos, my name is Dr Rosado, and I am the doctor taking care of you today. Please tell me what brings you in.

SP: Well, I am a university student. I was walking on campus and saw a booth promoting healthy habits for young adults. I walked over there. They asked me several questions, and recommended I seek a formal evaluation with a Doctor based on my answers to these questions. And that’s why I am here now.

Doctor: I am very happy you have come here. Before we get started, I would like to know a little more about you. You mentioned you are a university student?

SP: Yes I’m a junior (3rd year).

Doctor: Good. And with whom do you live with?

SP: I am living at home with my mom, dad, and little sister.

Doctor: Ok. In terms of your medical history, do you have any medical conditions?

SP: No

Doctor: Any surgeries?

SP: I had my tonsils removed when I was 8, but nothing else.

Doctor: Do you take any medications or supplements?

SP: No

Doctor: Any allergies?

SP: No

Doctor: Well then, you mentioned your family, mom and dad. How is their health?

SP: Overall pretty good. Dad has high cholesterol, and Mom and Type II diabetes and arthritis.

Doctor: Ok, and…

SP: (interrupts): My sister is healthy

Doctor: Excellent. No I am going to ask several more sensitive questions…

SP: Ok

Doctor: Let me remind you everything that we discuss here is completely confidential, except in cases of sexual abuse or sexually transmitted infections that I must report by law. Ok? Do you have any children?

SP: No

Doctor: Are you in a relationship?

SP: I’m not going out with anyone formally right now. My last relationship was in November, and we have since broken up.

Doctor: Since then, have you been sexually active?

SP: Yes, I have been sexually active since November. I have been with several people. I am exploring my sexuality.

Doctor: How many people have you been sexually active with in the past 6 months?

SP: I think about 12 in total. At least the last 8 have been men.

Doctor: Cool. How do you identify yourself sexually? Are you gay, bisexual, or have another term that you use?

SP: Well, I’m probably gay, but I’m not comfortable saying that in public yet.

Doctor: Ok, how would you describe your sexual activities? Do you have oral sex, anal sex, vaginal sex, etc?

SP: A little bit of everything.

Doctor: I understand. Do you use condoms consistently?

SP: I try to, but I’m going to be honest. If I’m out with my friends, drinking, sometimes I forget.

Doctor: Ok

SP: But yes, not all the time, but sometimes I forget

Doctor: You mentioned drinking. Do you use any other substance such as cocaine, heroin, crystal meth, or marijuana?

SP: I use marijuana every once in a while, but that’s the only one.

Doctor: Ok. Have you ever been diagnosed with a sexually transmitted infection (STI)?

SP: I haven’t, but that’s why I’m here. One of my partners told me he tested positive for gonorrhea and that concerned me.

Doctor: Ok, I am glad you are concerned. Based on the information you told me, and the fact that the booth sent you here, I understand you are high risk to contract a sexually transmitted infection (STI). The first thing we will do is test you for gonorrhea given you may have been exposed.

SP: Perfect

Doctor: We will also test you for HIV, syphilis, and other STIs. Sometimes these diseases run together.

SP: Yes, yes. I will do everything you think is necessary.

Doctor: Ok, very good. I also wanted to counsel you on a few things. First, let’s talk about your alcohol intake. Sometimes when you mix alcohol with other substances, you forget to be careful and use careful sexual practices. So, I advise you to limit your alcohol intake. I also strongly suggest you use condoms consistently.

SP: Yes, yes.

Doctor: And, taking advantage of our meeting, I wanted to share some information regarding a medication called PrEP.

SP: Ok

Doctor: It is also called Truvada. It is a drug used to prevent acquiring HIV.

SP: Oh

Doctor: Yes, it reduces the risk significantly of acquiring HIV in people who are HIV negative.

SP: In people like me?

Doctor: Exactly

SP: OK, and…

Doctor: Would you like to know more about this?

SP: I think so. Would you take this drug every day?

Doctor: Yes, you would take this medication every day.

SP: And, what are the side effects?

Doctor: The most commonly reported side effects are headaches, nausea, and diarrhea. These tend to occur in first two weeks of starting the medication. They usually get better with time.

SP: Ok, can I drink alcohol while I am taking this medication? I know there are medications you can’t take with alcohol.

Doctor: The truth is there is no data to suggest it interacts with alcohol. But as I mentioned before, I suggest your alcohol intake to two drinks per occasion.

SP: Can I take the medication any time of day? Do I have to take it with meals? Are there any formal instructions?

Doctor: No, no, nothing like that. You can take the medications with out without food. It does not really matter if you take in the morning or at night, the important thing is remembering to take it daily.

SP: Ok, ok. So it protects me from HIV?

Doctor: Yes, it protects you from HIV but does not protect you from acquiring other diseases. Therefore it is very important to use the medication in conjunction with condoms to reduce the risk of acquiring other infections.

SP: Ok, ok.

Doctor: Ok.

SP: And besides the gonorrhea test etc, is there anything else we have to do before starting the medication?

Doctor: Definitely, before initiating the medication we have to run several additional tests, such as measuring your renal function. We also have to test you for hepatitis.

SP: Ok, that sounds good Doctor.

Doctor: Great.

SP: I’m interested.

Doctor: Thats great, I am glad you are interested. Now I’ll have you go the nurses’ station to draw your blood and collect a urine sample. Then I will give you 90 day prescription for the medication.

SP: Ohh

Doctor: And I would like to see you in three months for the follow up visit.

SP: Sounds good.

Doctor: Take care, it was nice to meet you.

SP: Thank you Doctor!
