# Supplementary material for: HIV Pre-exposure Prophylaxis Education for Clinicians Caring for Spanish-Speaking Men Who Have Sex With Men (MSM)
Source: MedEdPORTAL. 2021 Mar 18;17:11110. doi: 10.15766/mep_2374-8265.11110 (PMC8015640; doi:10.15766/mep_2374-8265.11110)
Supplement: Supplementary file 1 — Spanish PPT Presentation.pptxEnglish PPT Presentation.pptxSpanish Audio-Guided PPT Video Presentation.pptxEnglish Audio-Guided PPT Video Presentation.pptxDiscussion Guide.docxPatient-Physician Video.mp4Spanish Transcript of Patient-Physician Video.docxEnglish Transcript of Patient-Physician Video.docxPreworkshop Evaluation Form.docxPostworkshop Evaluation Form.docx [file mep_2374-8265.11110-s001.zip › I. Preworkshop Evaluation Form.docx]

**PrEP Education for Clinicians - Pretest**

- 1. **This quiz serves to assess one's familiarity on PrEP. Please give yourself an unique ID:***

_________________________

- 1. **How much confidence do you have in your ability to describe approaches to prevent HIV transmission?**

*Mark only one oval.*

0 1 2 3 4

No Confidence Complete Confidence

- 1. **How much confidence do you have in your ability to list the indications to prescribe PrEP?**

*Mark only one oval.*

0 1 2 3 4

No Confidence Complete Confidence

- 1. **How much confidence do you have in your ability to order the appropriate laboratory tests prior to starting patients on PrEP?**

*Mark only one oval.*

0 1 2 3 4

No Confidence Complete Confidence

- 1. **How much confidence do you have in your ability to** describe common side effects associated with the use of PrEP

*Mark only one oval.*

0 1 2 3 4

No Confidence Complete Confidence

- 1. **How much confidence do you have in your ability to** identify barriers to PrEP adherence?

*Mark only one oval.*

0 1 2 3 4

No Confidence Complete Confidence

- 1. **How much confidence do you have in your ability to** describe proper medical follow-up for patients using PrEP?

*Mark only one oval.*

0 1 2 3 4

No Confidence Complete Confidence

- 1. **How much confidence do you have in your ability to** identify indications to discontinue PrEP?

*Mark only one oval.*

0 1 2 3 4

No Confidence Complete Confidence

- **Please select your current professional role**

*Mark only one oval.*

Medical Student Resident or Fellow

Academic faculty (MD, DO, NP, PA, other) Clinician in private practice (MD, DO, NP, PA) Other
